# Dichloro Butenediamides as Irreversible Site‐Selective Protein Conjugation Reagent

**DOI:** 10.1002/anie.202108791

**Published:** 2021-09-29

**Authors:** Victor Laserna, Daniel Abegg, Cláudia F. Afonso, Esther M. Martin, Alexander Adibekian, Peter Ravn, Francisco Corzana, Gonçalo J. L. Bernardes

**Affiliations:** ^1^ Yusuf Hamied Department of Chemistry University of Cambridge Lensfield Road CB2 1EW Cambridge UK; ^2^ Department of Chemistry The Scripps Research Institute 130 Scripps Way Jupiter Fl 33458 USA; ^3^ Instituto de Medicina Molecular João Lobo Antunes Faculdade de Medicina Universidade de Lisboa Avenida Professor Egas Moniz 1649-028 Lisboa Portugal; ^4^ AstraZeneca R&D BioPharmaceuticals Unit|Antibody Discovery & Protein Engineering (ADPE), Milstein Building Granta Park Cambridge CB21 6GH UK; ^5^ Department of Biotherapeutic Discovery H. Lundbeck A/S Ottiliavej 9 2500 Valby Denmark; ^6^ Departamento de Química Centro de Investigación en Síntesis Química Universidad de La Rioja 26006 Logroño Spain

**Keywords:** bioconjugation, cysteine, irreversibility, maleimides, Michael addition

## Abstract

We describe maleic‐acid derivatives as robust cysteine‐selective reagents for protein labelling with comparable kinetics and superior stability relative to maleimides. Diamide and amido‐ester derivatives proved to be efficient protein‐labelling species with a common mechanism in which a spontaneous cyclization occurs upon addition to cysteine. Introduction of chlorine atoms in their structures triggers ring hydrolysis or further conjugation with adjacent residues, which results in conjugates that are completely resistant to retro‐Michael reactions in the presence of biological thiols and human plasma. By controlling the microenvironment of the reactive site, we can control selectivity towards the hydrolytic pathway, forming homogeneous conjugates. The method is applicable to several scaffolds and enables conjugation of different payloads. The synthetic accessibility of these reagents and the mild conditions required for fast and complete conjugation together with the superior stability of the conjugates make this strategy an important alternative to maleimides in bioconjugation.

## Graphical abstract

Maleic acid derivatives are studied as cysteine‐selective reagents for protein labelling with comparable kinetics and superior stability to existing reagents. A novel mechanism is described to readily access stable chloro‐maleamic acid. The mild conditions, fast kinetics, and irreversible conjugation make this method an appealing alternative to existing cysteine‐targeted methods.

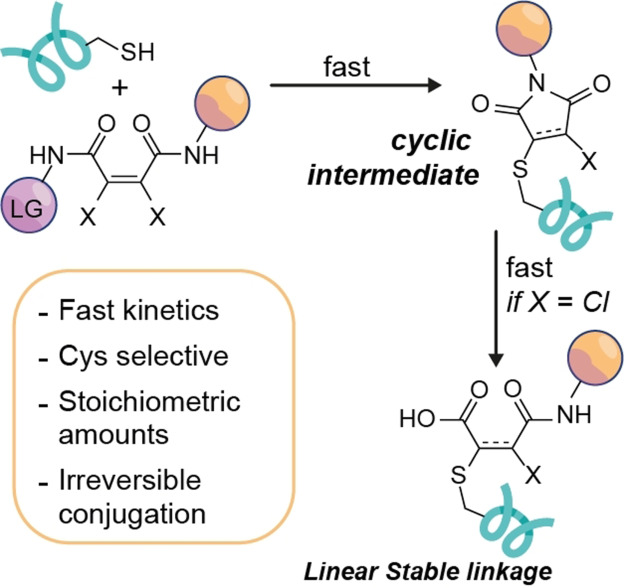

## Introduction

Over the last decade protein site‐selective conjugation has become a thriving research field.^[1]^ A plethora of methods have been reported for the construction of protein conjugates with a wide array of applications in chemical biology and medicine.^[2]^ Cysteine^[3]^ (Cys) and lysine^[4]^ (Lys) remain the main target residues in protein modification although recently, methodologies that target alternative residues, such as aspartic/glutamic acid,^[5]^ tryptophan,^[6]^ methionine,^[7]^ serine,^[8]^ or tyrosine,^[9]^ have been described. Cys‐targeting methods are particularly ubiquitous, as they yield structurally homogeneous conjugates in proteins that contain native^[10]^ or genetically engineered free Cys.^[11]^ Multiple strategies have been reported for Cys‐selective conjugation, usually based on alkylation^[12]^/arylation^[13]^ reagents or Michael acceptors.^[14]^ Michael acceptors, particularly maleimides, remain the most commonly used reagent for the construction of conjugates for biological applications^[15]^ because of their associated fast reaction kinetics. In fact, a number of Food and Drug Administration‐approved conjugates, such as antibody–drug conjugates (ADCs) Brentuximab vedotin^[15]^ and Trastuzumab emtansine^[16]^ or PEGylated conjugate Certolizumab pegol,^[17]^ contain a thio‐succinimide adduct derived from maleimide conjugation.^[18]^

However, it is well known that thio‐succinimide adducts can undergo fast and uncontrolled disruptive cleavage by thiol‐exchange in plasma, which ultimately compromises the safety and efficacy of the conjugate.^[19]^ Considerable efforts have been reported on how to increase the stability of maleimide‐based constructs.^[20]^ The most common approach consists of hydrolyzing the thio‐succinimide linkage to form a stable, linear thio‐succinamic acid species.^[21]^ Different strategies to control the hydrolysis kinetics have been described. For instance, the addition of electron‐withdrawing groups in the payload,^[22]^ tuning the amino‐acid environment near the inserted Cys,^[21a]^ or mild ultrasonication^[23]^ can accelerate thio‐succinimide hydrolysis. Alternatively, maleimides with different leaving groups attached to the vinylic bond or similar structures have been reported to form conjugates with higher stability based on maleimide or maleamic acid linkages.^[24]^

There are also acyclic reagents, such as benzoyl acrylic reagents[^11^, ^25^] or fumarate derivatives.^[26]^ They produce conjugates with linear linkages, which can present enhanced stability in plasma relative to maleimides.[^11^, ^25^] Despite these advances, most of these strategies fail to combine fast conjugation kinetics along with the formation of stable conjugates, which is required for biological applications, and where maleimides remain the first choice. The challenge remains to find a general, simple alternative for Cys bioconjugation with comparable kinetics and synthetic accessibility to maleimides, but complete linkage stability.

**Scheme 1:**
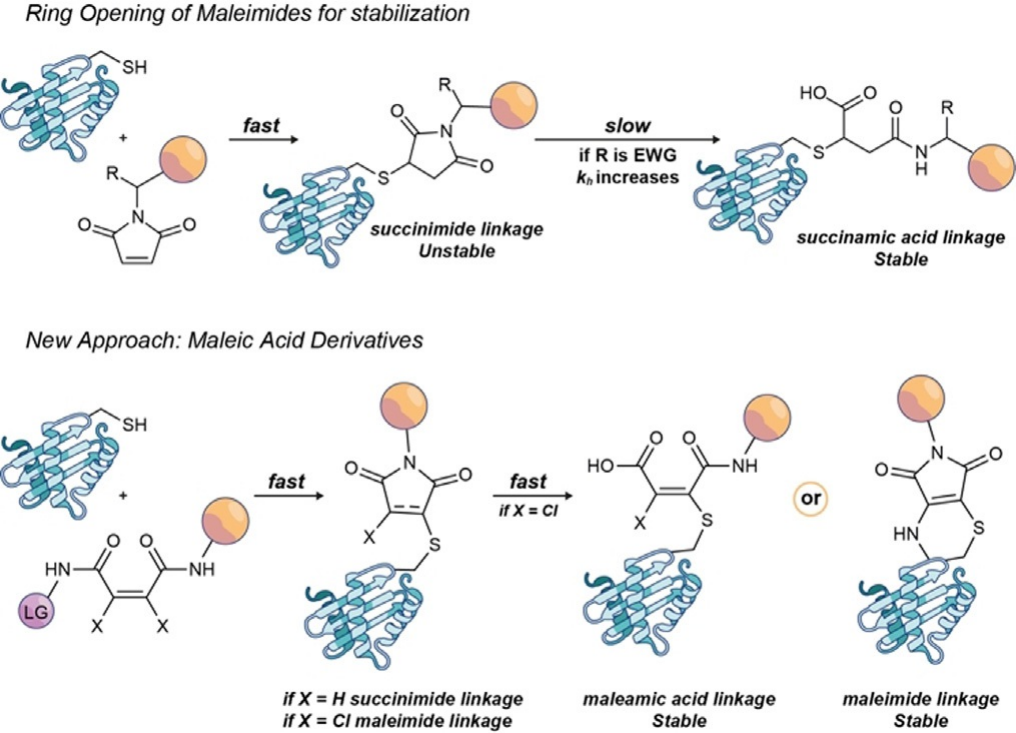
Synthesis of conjugates with linear succinamic acid linkages.

We hereby report on diamide and amido ester derivatives from maleic acid as new, efficient reagents for Cys‐selective protein conjugation. These reagents undergo fast Michael addition reactions with free Cys‐containing proteins in stoichiometric amounts under physiological conditions. Conjugation occurs through an unprecedented mechanism in which, upon Cys addition, spontaneous cyclization is promoted by attack of an amide moiety to the distal carbonyl group to form a succinimide link (Scheme 1). By expanding this approach to 2,3‐dichloro butenediamides, we can selectively target free Cys residues that form chloro‐maleimide intermediate linkages, which quickly undergo hydrolysis. The final conjugates are fully resistant to thiol‐exchange and cleavage in the presence of biological thiols and human plasma. Through this approach an array of free Cys‐containing proteins could be irreversibly tagged, such as ubiquitin (Ub), human serum albumin (HSA), or different antibody fragments, with different tags including azide or alkyne functionalities. The favorable kinetics, stability, and stoichiometry of the conjugation makes a flexible platform to access complex constructs, such as ADCs.

## Results and Discussion

With the goal of finding alternative approaches to access homogeneous conjugates with fully stable linkages in free Cys‐containing proteins, we set out to investigate maleic acid derivatives with labile functionalities, which we envisioned could undergo fast Michael addition with thiols under physiological conditions and, upon conjugation, would hydrolyze to form succinamic acid structures or equally stable analogues.

**Figure 1:**
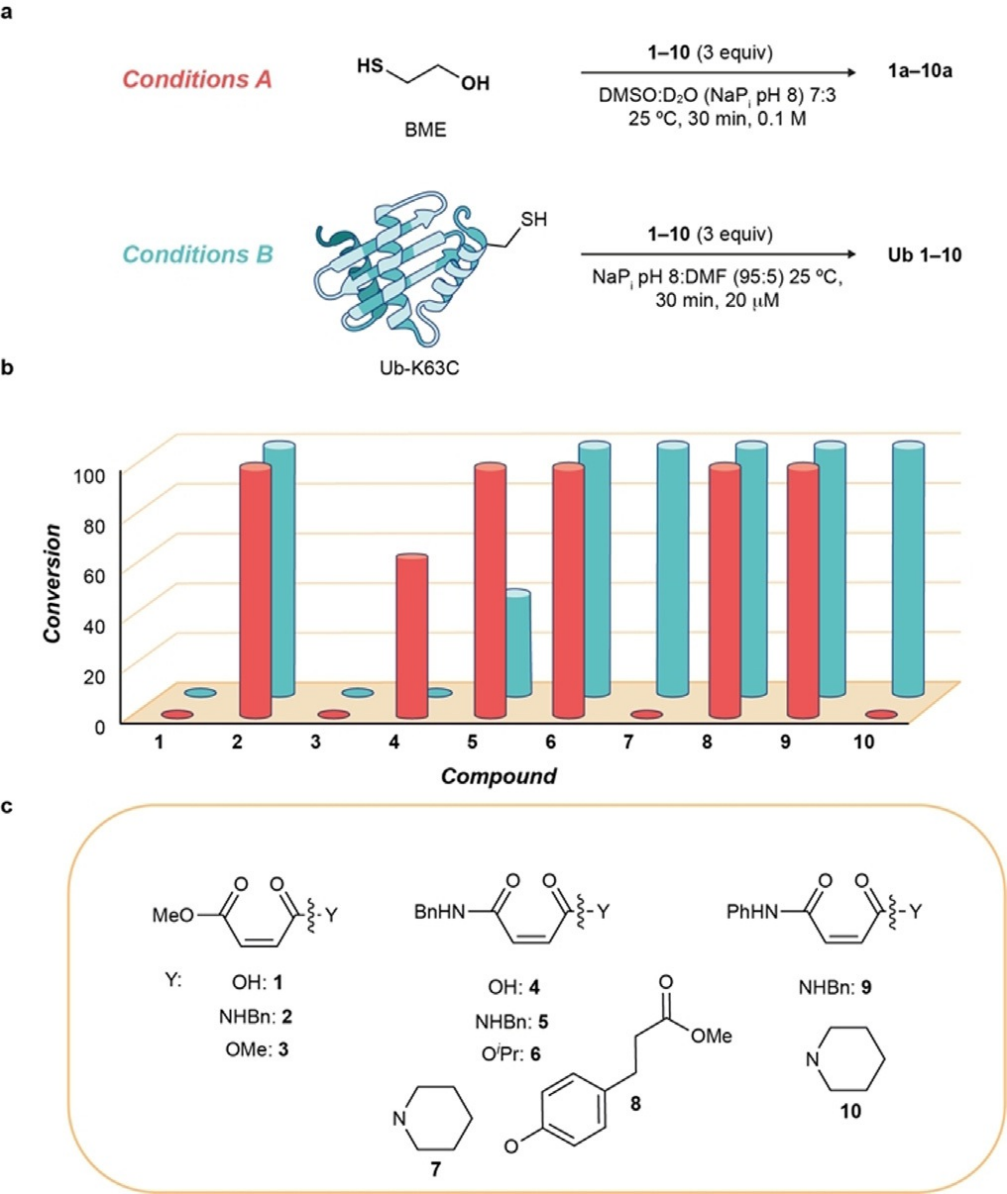
Small molecule and Ub reaction with different maleic acid derivatives. a) Small molecule and protein reaction conditions; b) Conversions observed using **conditions A** or **B**; c) Structures of reagents **1**—**10**. DMF=dimethyl formamide.

The reactivity of β‐mercaptoethanol (BME) was evaluated with different maleic acid derivatives (**1**–**10**, Figure 1 **, Conditions A**, Figures S3–S6) to assess their reactivity as Michael acceptors. Having determined the reactivity of **1**–**10** in small molecule experiments, we now exposed these reagents to the presence of a free Cys‐containing protein (Figure 1 **, Conditions B**, Figures S25–S31). As a model protein we chose Ub, which was engineered with a reactive Cys at position 63 (K63C). Surprisingly, the reactivity of the different species varied depending on the setup. Some species showed high reactivity in small molecule reactions but no protein conjugation reactivity, whereas others proved to be very efficient protein conjugation reagents but were unreactive in a small molecule setup. In successful conjugations, Cys‐selectivity was confirmed after digestion by MS/MS studies (Figure S82, S83) and by using Ellman's reagent as a chemical control (Figure S32).

**Scheme 2:**
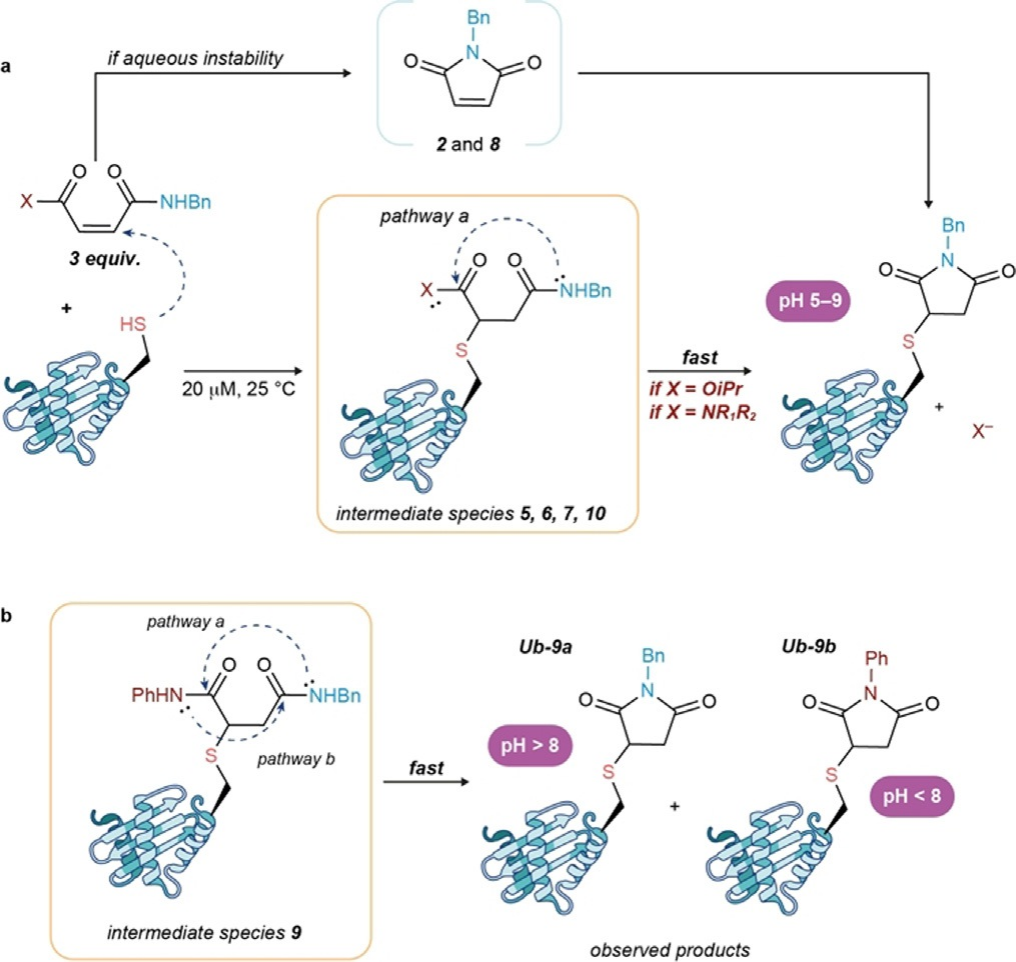
Regioselectivity of the cyclization step with compound **9**.

Mass analysis of the protein conjugates formed revealed a unique conjugate with a mass of 8754 Da (protein mass + 188 Da) for all the examples. Interestingly, the mass of the newly formed conjugate did not correspond to the addition of the full reagent. Instead, we observed the formation of a thio‐succinimide linkage. The origin of such a linkage is through intramolecular attack of the amide to the distal carbonyl group (Scheme 2 a), which can happen prior to or after Cys conjugation. Similar cyclizations are known although never triggered by aqueous solution or thiol conjugation.^[27]^ Furthermore, the cyclization step seems to play a key role in the viability of the conjugation, because only compounds with at least one secondary amide, capable of driving such a cyclization, underwent conjugation. To gain better mechanistic insight, compounds **2**, and **5**–**10** were stirred for 2 h at 25 °C in DMSO/D_2_O (NaP_i_ pH 8, 50 mm) to determine if the cyclization was induced as a result of the pH of the solvent or the thiol addition. Amido esters **2** and **8** cyclized spontaneously in the presence of D_2_O pH 8 to form a maleimide species (Figures S1,S2). However, this was not the general behavior for amido esters; compound **6** together with diamides **5**, **7**, **9**, and **10** was completely stable under these conditions. This aqueous stability supports the idea that thiol addition in the protein environment is responsible for triggering the cyclization. By using diamide **10** and Ub as a model, the reaction showed great tolerance for pH [5–9, Figures S33–S37)] and temperature [25–37 °C (Figures S31 and S36)].

When a reagent with two different secondary amide groups, such as **9**, is conjugated, a regioselectivity issue arises. The presence of two secondary amide moieties allows for two possible cyclizations, each driven by one of the amides (Scheme 2 b); each pathway leads to a different conjugate: **9 a** or **9 b**. The preferred pathway could be controlled by the pH at which the reaction was done. Under slightly acidic conditions, pH 6–7, the aryl amide group‐directed cyclization was favored, whereas under basic conditions, pH 8–9, the opposite selectivity was observed (Figures S38, S39).

Another appealing aspect of this conjugation reaction is the controlled release of small molecules in a particular microenvironment of the protein sequence. Upon cyclization, an amine (diamides) or alcohol (amidoesters) molecule is released, which could open new approaches for monitoring reactions (release of fluorophores), Cys‐targeted decaging of payloads or site‐directed bifunctionalization of proteins. We are currently investigating this directed release in our laboratories and will report it in due course.

The thio‐succinimide linkages obtained are known to hydrolyze over time under physiological conditions to give a linear and stable N‐substituted thio‐succinamic acid linkage, but the kinetics of hydrolysis is extremely slow and often yields mixtures of conjugates. Harsh hydrolyzing conditions, such as high pH and temperature, could ultimately lead to the complete hydrolysis to succinamic acid linkages, but such conditions are not compatible or desirable with proteins because they can result in unfolding and loss of functional properties. To address this problem, we focused on accelerating the hydrolysis of the linkages created by the conjugation of maleic acid diamides.

**Figure 2:**
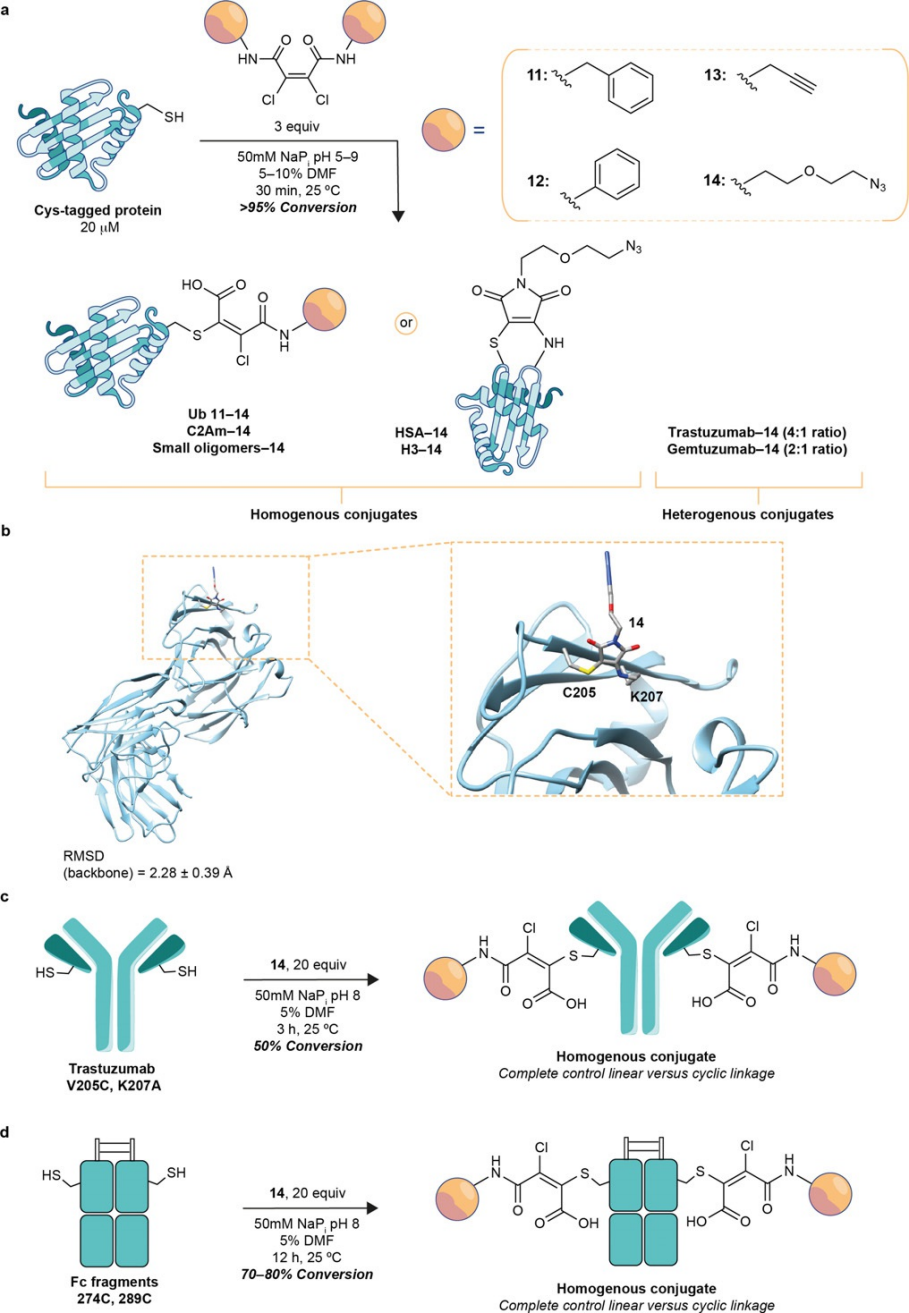
a) Conjugation reaction of reagents **11**–**14** with different Cys‐containing proteins. b) Representative snapshot derived from 0.5 μs MD simulations on conjugate HER2‐**14**. The RMSD value of the protein, relative to the first frame, is also shown. c) Conjugation reaction of Trastuzumab V205C, K207A with **14**. d) Conjugation reaction of Fc fragments 274C and 289C with **14**.

Aqueous instability of dichloro maleimides^[24c]^ suggests that hydrolysis kinetics of maleimides can be exponentially increased by the introduction of halogens into the ring. This led us to think that, by introducing chlorine substituents into the alkene scaffold of maleic acid diamides, the stability of the compounds and their reactivity would not be affected, although now instead of obtaining conjugates with succinimide linkages, chloro‐maleimide linkages would be generated, which would quickly hydrolyze to stable linear chloro maleamic acid species. By following reported procedures^[28]^ we synthesized symmetrical dichloro butenediamides (**11**–**14**, Figure 2 a) and, to our delight, their reactivity still included a cyclization step which yielded a cyclic chloro‐maleimide linkage with enhanced hydrolysis kinetics. Addition of **11** (3 equiv) to a solution of Ub resulted in complete conjugation (Figure 2) after 30 min at 25 °C. The cyclic intermediate was observed if the reaction mixture was analyzed shortly after addition of the reagent (Figure S71). No trace of cyclic maleimide linkage and only linear chloro maleic acid linkage was noted after 30 min at different temperatures (25–37 °C) and pH [pH 5–9 (Figures S67–S70)], which further supports the hypothesis of rapid hydrolysis of the linkage. The mechanism associated with this conjugation is similar to that of dibromo maleimides^[23]^ in that the linkages obtained are similar, but an additional cyclization step and much more favorable hydrolysis kinetics make this approach advantageous to obtain homogeneous, stable maleamic acid linkages.

The generality of the method was demonstrated by conjugation of **7** and **14** to alternative free Cys‐containing proteins. Under similar conditions, HSA, the N‐terminal domain of phage repressor R434 and a camelian nanobody targeted at amyloid precursors (HET nanobody)^[29]^ were effectively conjugated (Figures S40–S42) to **7**. In all examples a Michael addition/cyclization mechanism to yield a succinimide was observed, which supports the generality of the mechanism in different protein sequence microenvironments. Analysis of the circular dichroism (CD) profiles of the conjugates relative to native proteins confirmed that overall the tertiary structure of the proteins was maintained throughout conjugation (Figures S85, S86).

An array of dichloro butenediamide analogues with different functionalities were synthesized (compounds **11**–**14**). Alyphatic **11** and aromatic **12** amide species could be accessed as well as amides with an incorporated alkyne **13** or azide **14** functionality. These new functionalities were incorporated into the protein structure to enable further functionalization through copper(I)‐catalyzed alkyne–azide cycloadditon. Compounds **12**–**14** showed similar reactivity to **11** upon reaction with Ub to form stable, linear chloro‐maleamic acid linkages (Figures S44–S46). However, when compound **14** was tested with different proteins, upon conjugation–cyclization, different reactivity was observed for some of these proteins. In Ub, C2Am^[30]^ or peptide ASCATN the expected sequential Michael addition–cyclization–hydrolysis occurs to give a final conjugate with a chloro‐maleamic acid linkage. When the scope is expanded to proteins like HSA, H3K4C, or the full‐length IgG antibody Gemtuzumab, an alternative pathway consisting of a subsequent Michael addition of a proximal nucleophilic residue occurs, instead of the hydrolysis step (Figure 2 b). This alternative pathway forms cyclic maleimide linkages with two residues on the protein. Both linkages cannot undergo retro‐Michael addition and form stable conjugates, which was demonstrated by analyzing the stability of **Ub‐14**; the conjugate remained stable in buffer, and no degradation was observed after 48 h at 37 °C in the presence of glutathione (1 mm) or in human plasma (Figures S73, S74).

The enhanced plasma stability displayed by the conjugates formed from the reaction of Cys with reagents **11**–**14** has potential for the construction of stable ADCs. However, because two alternative linkages can be formed, this could constitute an issue regarding homogeneity of the final construct. Next, we analyzed the ratio between cyclic and linear conjugates using LCMS. The conjugate derived from Gemtuzumab V205C showed a mixture of species, [ratio 2:1 cyclic vs. linear (Figure S53)], but all others formed selectively one of the two potential linkages. Conjugation of HER2‐targeted IgG Trastuzumab V205C with **14** led to a similar result (ratio 4:1, Figure S54).

To explain this result at the atomic level, we performed molecular dynamics (MD) simulations on the Fab fragment of Trastuzumab conjugated to compound **14** (Figures 2 b and S87) and examined the distance from all lysine residues to the reactive carbon (C−Cl). According to these calculations, lysine at position 207 had the smallest average distance and was presumably the residue that directed the second nucleophilic attack to form the cyclic derivative. The simulations indicated also that the incorporation of this cyclic derivative into Fab does not significantly disrupt its structure (Figure 2 b). The involvement of K207 in the formation of the cyclic linkage was further confirmed by MS/MS studies on Trastuzamab V205C‐**14** (Figure S84). By mutating lysine 207 into an alanine, the new Trastuzumab V205C+K207A was conjugated to **14** and this time complete selectivity to the linear linkage was obtained (Figure S55). This result supported the idea that nearby lysine residues were the driving factors for the formation of the cyclic linkage, allowing us to predict the final linkage formed and if mixtures were to arise, avoid the formation of the cyclic species by introducing cysteines in the appropriate place or mutating the problematic lysine residues. We found, however, that mutation of lysine 207 in Trastuzumab V205+K207A reduces the nucleophilicity of cysteine 205 and only approx. 50 % conjugation is achieved under the previously described conditions (Figure S55).

To find suitable positions to genetically encode a reactive Cys that allow complete conversion, yet selective formation of the stable linear linkage, we produced Fc fragments of an IgG with Cys insertions at different positions (239iC, 268C, 274C, 289C and 442C). Under identical reaction conditions with **14**, the Fc fragments with 274C and 289C reacted completely and selectively formed the linear linkage (Figures S56–S60). These simple maleic acid derivatives enable installation of modifications on antibodies at specific sites to form homogeneous and stable antibody conjugates.

To compare the kinetics of conjugation between maleic acids and maleimides, an equimolar mixture of **11** and its analogous maleimide reagent were conjugated to Fc fragment 289iC. Under the same conditions, **11** and maleimide showed comparable conjugation kinetics as a 1:1 mixture of Fc‐**11** and Fc‐maleimide constructs was obtained (Figure S81). However, when we compared the stability of the constructs in the presence of GSH (1 mm) after 66 h at 37 °C, the constructs (trastuzumab‐**11**, Fc 274iC‐**11**, Fc 289iC‐**11**) conjugated to **11** showed higher stability than the corresponding species conjugated with maleimide (Figures S75–S80). While the conjugates formed with reagent **11** remain intact in the presence of a thiol, constructs built using maleimides form a mixture of hydrolyzed maleimide and unconjugated antibody. These data show a key advantage of our reagents to build stable conjugates and illustrate how enhanced hydrolysis kinetics of the cyclic linkages allows the formation of fully stable species.

## Conclusion

We describe the protein conjugation reactivity of a series of maleic acid derivatives and identify amido esters and diamides as viable conjugation reagents to produce succinimide linkages through a new stepwise mechanism. The strategy was optimized to use dichloro butenediamides, which promote a further hydrolysis step to form completely stable linkages to proteins. This latter species shows comparable conjugation kinetics relative to maleimides yet benefits from superior stability. The general mechanism described composes a Michael addition followed by spontaneous cyclization to form cyclic linkages similar to those obtained by conjugation to maleimides. Introduction of Cl atoms into the cyclic linkages promotes subsequent hydrolysis, leading to fully stable linkages. Nearby nucleophilic residues can promote an alternative mechanism where a second nucleophilic attack leads to the formation of a cyclic linkage, but this pathway can be controlled and depleted if undesired conjugate mixtures are obtained. The approach allows for the creation of conjugates with azide/alkyne moieties and opens up possibilities to create tailored conjugation for a number of relevant proteins, including nanobodies or IgGs relevant for the formation of ADCs.

## Conflict of interest

The authors declare no conflict of interest.

## Supporting information

As a service to our authors and readers, this journal provides supporting information supplied by the authors. Such materials are peer reviewed and may be re‐organized for online delivery, but are not copy‐edited or typeset. Technical support issues arising from supporting information (other than missing files) should be addressed to the authors.

Supporting Information
