# Supplementary material for: Dichloro Butenediamides as Irreversible Site‐Selective Protein Conjugation Reagent
Source: Angew Chem Int Ed Engl. 2021 Sep 29;60(44):23750–5. doi: 10.1002/anie.202108791 (PMC8596790; doi:10.1002/anie.202108791)
Supplement: Supplementary file 1 — Supporting Information [file ANIE-60-23750-s001.pdf]

Angewandte Chemie
Eine Zeitschrift der Gesellschaft Deutscher Chemiker

Supporting Information

Dichloro Butenediamides as Irreversible Site-Selective Protein Conjugation Reagent

Victor Laserna, Daniel Abegg, Cláudia F. Afonso, Esther M. Martin, Alexander Adibekian, Peter Ravn, Francisco Corzana, and Gonçalo J. L. Bernardes**

anie_202108791_sm_miscellaneous_information.pdf

Table of Contents

1.	General Experimental Information	S3
2.	Experimental Procedures	S4
3.	Small Molecule Experiments	S12
4.	Protein Conjugation Experiments	S19
5.	Stability Studies	S110
6.	Competition Experiments	S124
7.	LC-MS/MS Studies	S126
8.	CD Studies	S128
9.	Molecular Dynamic (MD) Simulations	S129
10.	References	S130
11.	^{1}H and ^{13}C NMR Spectra	S132

1. General Experimental Information

All solvents and chemicals were used as received. Column chromatography was carried out using Merck Geduran Si 60 (40-63 μm) silica gel. Analytical thin layer chromatography was carried out using Merck TLC Silica Gel 60 F_{254} aluminium-backed plates. Components were visualised using combinations of ultra-violet lights and potassium permanganate.

Proton magnetic resonance spectra (^{1}H NMR) were recorded at 400 or 600 MHz on a Bruker Avance spectrometer and are reported as follows: chemical shift δ in ppm (number of protons, multiplicity, coupling constant J in Hz, assignment). The solvent used was deuterated chloroform unless stated otherwise. Residual protic solvent was used as the internal reference, setting $CDCl_3$ to δ 7.26. The following abbreviations are used: s, singlet; d, doublet; t, triplet; q, quartet; m, mulitplet; br, broad or a combination of these. Carbon magnetic resonance spectra (^{13}C NMR) were recorded at 100 or 150 MHz on a Bruker Avance spectrometer using deuterated chloroform and using the central reference of $CDCl_3$ to δ 77.0 as the internal standard. Mass Spectrometry data were collected on either TOF or magnetic sector analysers at the Department of Chemistry, University of Cambridge. The ionization method is reported in the experimental data. LC-MS data was collected using a Waters Acquity UPLC system. Starting materials, including dimethyl fumarate were purchased from commercial suppliers (Merck, Fluorochem and Alfa Aesar) and used without further purification.

2. Experimental Procedures

Synthesis of (Z)-4-methoxy-4-oxobut-2-enoic acid 1

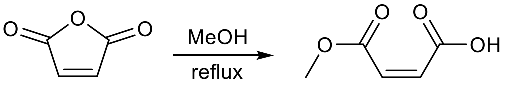

Maleic Anhydride (10 mmol, 980 mg) was dissolved into 50 mL of MeOH and reluxed for 3 h. The consumption of maleic anhydride was monitored by TLC and when no starting material was observed, the solvent was removed in vacuo. No further purification was necessary.

Compound **1**[1] was obtained as a colourless oil in 97 % yield (1.26 g).

^{1}**H NMR** (600 MHz, Chloroform-*d*) δ 6.43 (d, *J* = 12.6 Hz, 1H), 6.39 (d, *J* = 12.6 Hz, 1H), 3.88 (s, 3H). ^{13}**C NMR** (151 MHz, $CDCl_3$) δ 167.7, 165.5, 135.0, 129.8, 53.4.

Synthesis of Amido Maleic Acid

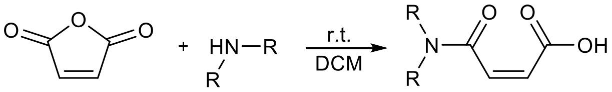

Maleic Anhydride (10 mmol, 980 mg) was dissolved into 50 mL of DCM and stirred at room temperature. Once it was completely dissolved, 1 equiv. of the corresponding amine was added dropwise into the solution. After the addition was completed, the mixture was stirred at room temperature until total consumption of anhydride was observed by TLC (30 min – 1h). The solvent was removed under vacuum and the product was recrystalized in DCM/hexane.

(Z)-4-(benzylamino)-4-oxobut-2-enoic acid **4**

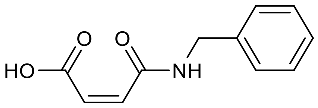

Compound **4**[2] was obtained as a white precipitate in 94 % yield (1.93 g).

^{1}**H NMR** (600 MHz, DMSO) *δ* 14.57 (s, 1H), 9.40 (t, J = 5.9 Hz, 1H), 7.46 – 7.12 (m, 5H), 6.43 (d, J = 12.4 Hz, 1H), 6.26 (d, J = 12.4 Hz, 1H), 4.39 (d, J = 5.9 Hz, 2H). ^{13}**C NMR** (151 MHz, DMSO) *δ* 166.2, 165.6, 138.4, 131.9, 128.88, 128.1, 127.63, 118.5, 43.1.

(Z)-4-oxo-4-(phenylamino)but-2-enoic acid

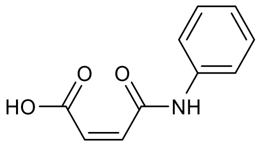

(Z)-4-oxo-4-(phenylamino)but-2-enoic acid[2] was obtained as a white precipitate in 91 % yield (1.74 g).

^{1}H NMR (400 MHz, DMSO-d_6) δ 10.37 (s, 1H), 7.64 – 7.57 (m, 2H), 7.31 (t, J = 7.9 Hz, 2H), 7.07 (t, J = 7.3 Hz, 1H), 6.46 (d, J = 12.1 Hz, 1H), 6.29 (d, J = 12.0 Hz, 1H). **^{13}C NMR** (101 MHz, DMSO) δ 167.3, 163.7, 139.0, 132.2, 130.9, 129.3, 124.3, 120.0.

Synthesis of Amido Maleates

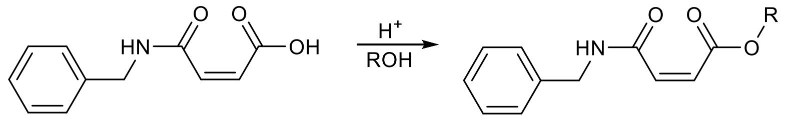

Compound **4** (205 mg, 1 mmol) was dissolved in 10 mL of the corresponding alcohol (ethanol or isopropanol). 5 drops of concentrated HCl were added into the solution and then stirred overnight. The solvent was removed under vacuum and the product was purified by column chromatography (AcOEt:hexane; 1:1).

methyl (Z)-4-(benzylamino)-4-oxobut-2-enoate **2**

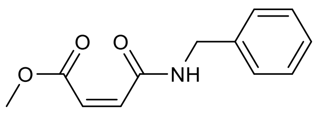

Compound **2**[2] was obtained as a yellow oil in 83 % yield (182 mg).

^{1}H NMR (400 MHz, Chloroform-d) δ 8.48 (br s, 1H), 7.40 – 7.29 (m, 5H), 6.40 (d, J = 13.1, 1H), 6.17 (dd, J = 13.1, 1H), 4.56 (d, J = 5.7 Hz, 2H), 3.78 (s, 3H). **^{13}C NMR** (101 MHz, $CDCl_3$) δ 166.61, 163.88, 138.42, 137.8, 128.7, 127.9, 127.5, 125.1, 52.4, 43.8.

Isopropyl (Z)-4-(benzylamino)-4-oxobut-2-enoate **6**

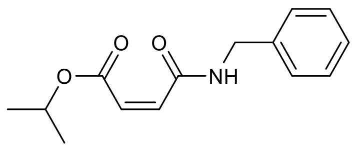

Compound **6**[2] was obtained as a white solid in 69 % yield (170 mg).

^{1}H NMR (400 MHz, Chloroform-*d*) δ 8.78 (br s, 1H), 7.35 (d, *J* = 2.8 Hz, 5H), 6.34 (d, *J* = 13.1 Hz, 1H), 6.13 (d, *J* = 13.1 Hz, 1H), 5.08 (p, *J* = 6.3 Hz, 1H), 4.55 (d, *J* = 5.7 Hz, 2H), 1.30 (d, *J* = 6.3 Hz, 6H). **^{13}C NMR** (101 MHz, $CDCl_3$) δ 165.8, 164.0, 138.2, 128.7, 128.2, 127.8, 127.4, 126.0, 69.5, 43.7, 21.6.

4-(3-methoxy-3-oxopropyl)phenyl (Z)-4-(benzylamino)-4-oxobut-2-enoate **8**

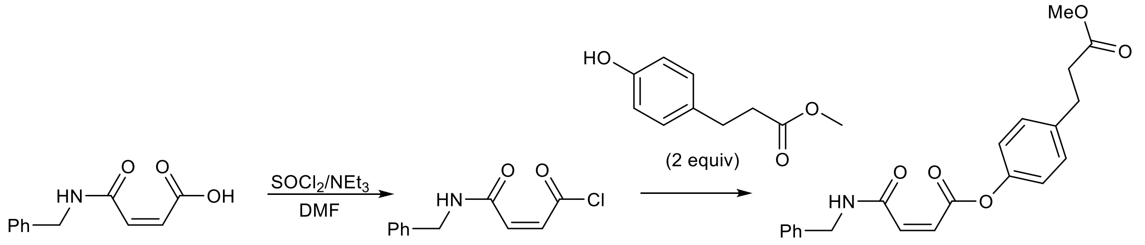

Compound **4** (205 mg, 1 mmol) was dissolved in 10 mL of DMF, the solution was cooled down to 0 ºC on an ice bath before 3 equiv of NEt_3 were added into the solution. Once a clear solution was obtained, $SOCl_2$ was added dropwise into the mixture and stirred for 30 min. After this time 2 equiv of methyl 3-(4-hydroxyphenyl)propanoate (360mg, 2 mmol) were added and the mixture was allowed to warm up to room temperature and stirred for 24 h. Once complete consumption of starting material was observed by TLC, 20 mL AcOEt were added, and the solution was washed with 2 x 20 mL of HCl 0.1M and 2 x 20 mL of distilled water. The organic fraction was collected the solvent was removed under vacuum and the product was purified by column chromatography (AcOEt: hexane; 3:2).

Compound **8** was obtained as a white solid in 58 % yield (212 mg).

^{1}H NMR (400 MHz, Chloroform-*d*) δ 8.00 (br s, 1H), 7.31 (m, 5H), 7.25 (d, *J* = 8.1 Hz, 2H), 7.07 (d, *J* = 8.1 Hz, 2H), 6.51 (d, *J* = 12.7 Hz, 1H), 6.37 (d, *J* = 12.7 Hz, 1H), 4.53 (d, *J* = 5.7 Hz, 2H), 3.70 (s, 3H), 2.98 (t, *J* = 7.8 Hz, 2H), 2.65 (t, *J*

= 7.7 Hz, 2H). **^{13}C NMR** (101 MHz, $CDCl_3$) δ 173.1, 164.8, 163.75, 148.5, 139.0, 138.8, 137.6, 129.4, 128.7, 127.9, 127.5, 125.0, 121.3, 51.68, 43.8, 35.5, 30.3. **HRMS** Found 390.13107 $[C_{21}H_{21}NO_5Na]^+$ calculated 390.1315.

Synthesis of Z-Butenodiamides

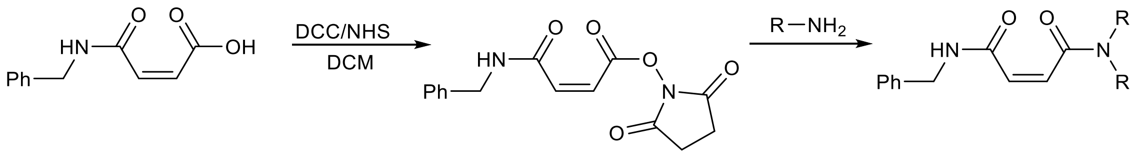

Compound **4** (205 mg, 1 mmol) and N-hydroxy succinimide (115 mg, 1 mmol, 1 equiv) were dissolved in 10 mL of DCM. Once a clear solution was obtained, N,N'-dicyclohexylcarbodiimide (206 mg, 1 mmol, 1 equiv) was added into the mixture and stirred for 30 min. A white precipitate appeared which was filtered out. The corresponding amine (1.5 mmol, 1.5 equiv) was added to the filtrate and the mixture was stirred for 3 h at room temperature. After this time the solvent was removed under vacuum and the product was purified by column chromatography (AcOEt:hexane; 1:1).

N1,N4-dibenzylmaleamide **5**

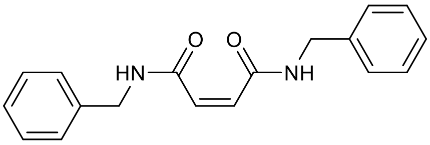

Compound **5**[3] was obtained as a white solid in 81 % yield (238 mg).

^{1}H NMR (400 MHz, Chloroform-d) *δ* 7.37 – 7.30 (m, 10H), 6.15 (s, 2H), 4.49 (d, J = 5.8 Hz, 4H). **^{13}C NMR** (101 MHz, $CDCl_3$) *δ* 164.7, 137.6, 132.6, 128.7, 127.8, 127.5, 43.8.

(Z)-N-benzyl-4-oxo-4-(piperidin-1-yl)but-2-enamide **7**

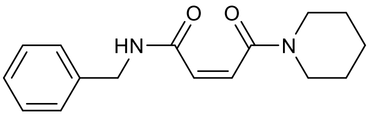

Compound **7** was obtained as a white solid in 57 % yield (154 mg).

^{1}H NMR (400 MHz, Chloroform-d) δ 8.27 – 8.08 (br s, 1H), 7.33 – 7.28 (m, 5H), 6.39 (d, J = 12.8 Hz, 1H), 6.12 (d, J = 12.8 Hz, 1H), 4.49 (d, J = 5.8 Hz, 2H), 3.57 – 3.50 (m, 2H), 3.40 (m, 2H), 1.63 (m, 2H), 1.57 – 1.45 (m, 4H). **^{13}C NMR** (101 MHz, $CDCl_3$) δ 165.6, 164.9, 138.2, 130.4, 130.2, 128.6, 127.7, 127.3, 47.7, 43.4, 42.5, 26.3, 25.3, 24.3. **HRMS** Found 295.14175 $[C_{16}H_{20}N_2O_2Na]^+$ calculated 295.14138.

N1-benzyl-N4-phenylmaleamide **9**

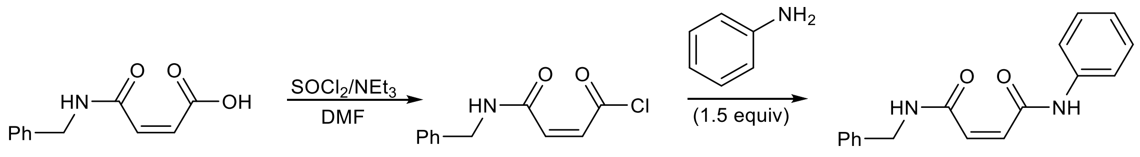

Compound **4** (205 mg, 1 mmol) was dissolved in 10 mL of DMF, the solution was cooled down to 0 ºC on an ice bath and 3 equiv of NEt_3 were added. Once a clear solution was obtained, $SOCl_2$ was added into the mixture and stirred for 30 min. After this time 1.5 equiv of aniline (140 mg, 1.5 mmol) were introduced and the mixture was allowed to warm up to room temperature and stirred for 24 h. After this time, 20 mL of AcOEt were added and the solution was washed with 2x20 mL of HCl 0.1M and 2 x 20 mL of distilled water. The organic fraction was collected, the solvent was removed under vacuum and the product was purified by column chromatography (AcOEt:hexane; 1:1).

Compound **9** was obtained as a white solid in 77 % yield (215 mg).

^{1}H NMR (400 MHz, Chloroform-*d*) δ 7.85 (br s, 1H), 7.67 (d, *J* = 8.0 Hz, 1H), 7.51 – 7.28 (m, 7H), 7.12 (t, *J* = 7.4 Hz, 1H), 6.23 (d, *J* = 13.5 Hz, 1H), 6.14 (d, *J* = 13.5 Hz, 1H), 4.52 (d, *J* = 5.7 Hz, 2H). **^{13}C NMR** (101 MHz, $CDCl_3$) δ 165.2, 162.5, 138.1, 137.2, 136.4, 130.2, 128.9, 128.8, 127.9, 127.8, 124.5, 120.3, 44.0. **HRMS** Found 303.1105 $[C_{17}H_{16}N_2O_2Na]^+$ calculated 303.1153.

(Z)-4-oxo-N-phenyl-4-(piperidin-1-yl)but-2-enamide **10**

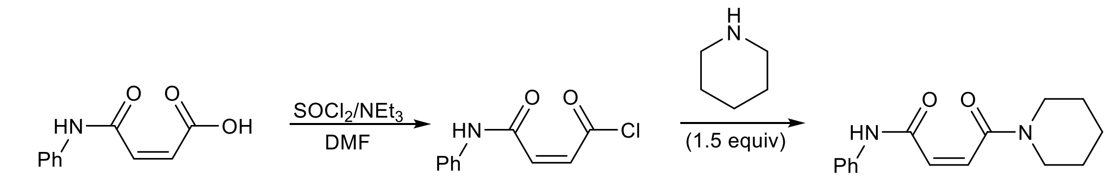

(Z)-4-oxo-4-(phenylamino)but-2-enoic acid (192mg, 1 mmol) was dissolved in 10 mL of DMF, the solution was cooled down to 0 ºC on an ice bath and 3 equiv

of NEt_3 where added into the solution. Once a clear solution was obtained, $SOCl_2$ was added into the mixture and stirred for 30 min. After this time, 1.5 equiv of piperidine (128 mg, 148 μL 1.5 mmol) were added to the mixture, it was then allowed to warm up to room temperature and stirred for 24 h. Once complete consumption of starting material was observed by TLC, 20 mL of AcOEt were added and the solution was washed with 2 x 20 mL of HCl 0.1M and 2 x 20 mL of distilled water. The organic fraction was collected, the solvent was removed under vacuum and the product was purified by column chromatography (AcOEt:hexane; 1:1).
Compound **10** was obtained as a white solid in 71 % yield (183 mg).

^{1}H NMR (400 MHz, DMSO-d_6) δ 10.19 (br s, 1H), 7.65 – 7.56 (m, 2H), 7.30 (dd, J = 8.5, 7.3 Hz, 2H), 7.04 (t, J = 7.3 Hz, 1H), 6.52 (d, J = 11.8 Hz, 1H), 6.17 (d, J = 11.8 Hz, 1H), 3.44 (t, J = 5.5 Hz, 2H), 3.27 (d, J = 5.6 Hz, 2H), 1.60 – 1.53 (m, 2H), 1.47 (m, 4H). **^{13}C NMR** (101 MHz, DMSO) δ 166.2, 162.7, 139.3, 136.4, 129.2, 125.7, 124.0, 119.6, 47.0, 41.6, 33.8, 25.8, 25.3, 24.6. **HRMS** Found 281.12649 $[C_{15}H_{18}N_2O_2Na]^+$ calculated 281.12607.

Synthesis of dichloro Z-Butenodiamides (**11–14**)

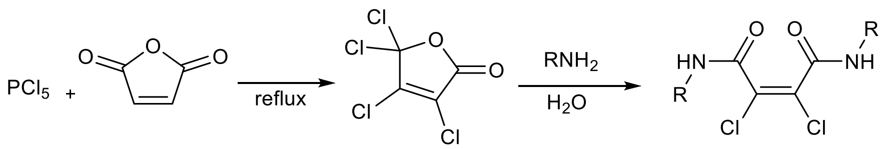

3,4,5,5-tetrachlorofuran-2(5H)-one and dichloro diamides 11-14 were obtained following literature procedures.[4] PCl_5 (80 mmol) was mixed with maleic anhydride (20 mmol) in a 250 mL round bottom flask. The mixture was heated until it melted, and a uniform yellow solution was obtained. This mixture was refluxed overnight. After this time the yellow oil was cooled down and dissolved in 50 mL ethyl acetate and washed with a saturated solution of $NaHCO_3$ (3 x 50 mL). The organic layer was then passed through a silica pad and concentrated in vacuo. Pure compound was obtained by distillation.

3,4,5,5-tetrachlorofuran-2(5H)-one (1 mmol) was dissolved in 3 mL of water and 4 equiv of the corresponding amine were added into the solution. Dichloro maleamides were obtained as brown precipitate which were separated by filtration and recrystallized in mixtures of hexane:DCM, 8:2 or MeOH:AcOEt, 1:9.

3,4,5,5-tetrachlorofuran-2(5H)-one

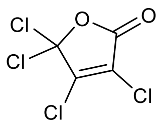

3,4,5,5-tetrachlorofuran-2(5H)-one was obtained as a colourless oil in 87% yield (3450 mg).

^{13}C NMR (101 MHz, $CDCl_3$) δ 158.6, 152.6, 122.9, 104.3.
N1,N4-dibenzyl-2,3-dichloromaleamide **11**

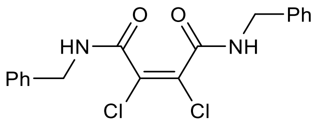

Compound **11** was obtained as a yellow solid in 60 % yield (217 mg).

^{1}H NMR (400 MHz, DMSO-d_6) *δ* 9.04 (t, J = 6.1 Hz, 2H), 7.29 (m, 10H), 4.31 (d, J = 6.0 Hz, 4H). **^{13}C NMR** (101 MHz, DMSO) *δ* 161.3, 138.9, 130.2, 128.7, 127.8, 127.4, 43.2. **HRMS** Found 363.0669 $[C_{18}H_{17}N_2O_2Cl_2]^+$ calculated 363.0667.

2,3-dichloro-N1,N4-diphenylmaleamide **12**

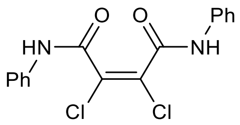

Compound **12** was obtained as a yellow solid in 49 % yield (164 mg).

^{1}H NMR (700 MHz, DMSO-d_6) δ 10.57 (s, 2H), 7.56 (d, *J* = 8.1 Hz, 4H), 7.32 (t, *J* = 7.8 Hz, 5H), 7.12 (t, *J* = 7.5 Hz, 2H). **^{13}C NMR** (176 MHz, DMSO) δ 159.7, 138.3, 130.8, 129.3, 125.0, 120.8. **HRMS** Found 357.0175 $[C_{16}H_{12}N_2O_2Cl_2Na]^+$ calculated 357.0174.

2,3-dichloro-N1,N4-di(prop-2-yn-1-yl) maleamide **13**

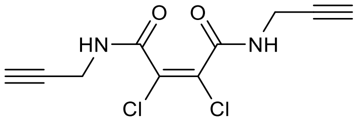

Compound **13** was obtained as a white solid in 43 % yield (111 mg).

^{1}H NMR (400 MHz, Chloroform-*d*) δ 6.37 (br s, 2H), 4.14 (m, 4H), 2.30 (t, *J* = 2.5 Hz, 2H). **^{13}C NMR** (101 MHz, $CDCl_3$) δ 160.7, 130.9, 78.0, 72.7, 30.1. **HRMS** Found 280.9869 $[C_{10}H_8N_2O_2Cl_2Na]^+$ calculated 280.9861.

N1,N4-bis(2-(2-azidoethoxy)ethyl)-2,3-dichloromaleamide **14**

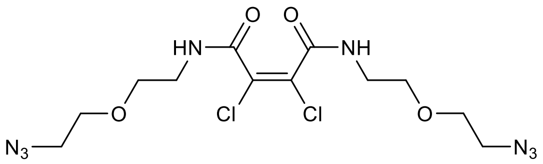

Compound **14** was obtained as a colourless oil in 18 % yield (73 mg).

^{1}H NMR (400 MHz, Chloroform-*d*) δ 3.83 (t, *J* = 5.4 Hz, 4H), 3.69 (t, *J* = 5.5 Hz, 4H), 3.66 – 3.59 (m, 4H), 3.36 – 3.26 (m, 4H). **^{13}C NMR** (101 MHz, $CDCl_3$) δ 163.0, 133.3, 69.9, 67.5, 50.6, 38.6. **HRMS** Found 409.0884 $[C_{12}H_{19}Cl_2N_8O_4]^+$ calculated 409.0891.

3. Small Molecule Experiments

Aqueous Stability Assessment of 1–10

A 0.1 M stock solution of compounds **1–10** was prepared in DMSO-d$_6$. Then in an NMR tube 0.1 mL each solution was diluted with another 0.4 mL of DMSO-d$_6$. 0.2 mL of D_2O (NaP$_i$ pH 8, 50 mM) was added and the mixture was sonicated until a clear solution was obtained. The NMR tube was kept at 25 ºC for 24 h. Once the time was over the solution was analysed by NMR. The stability was assessed by comparing the crude mixtures obtained to the original compounds dissolved in DMSO-d$_6$ and identifying possible subproduct formation.

Compounds **2** and **8** proved to be unstable under these conditions, as benzyl maleimide and methanol (**2**) or the corresponding phenol (**8**) could be detected. The rest proved to be stable under the same conditions and only starting material was detected after this time.

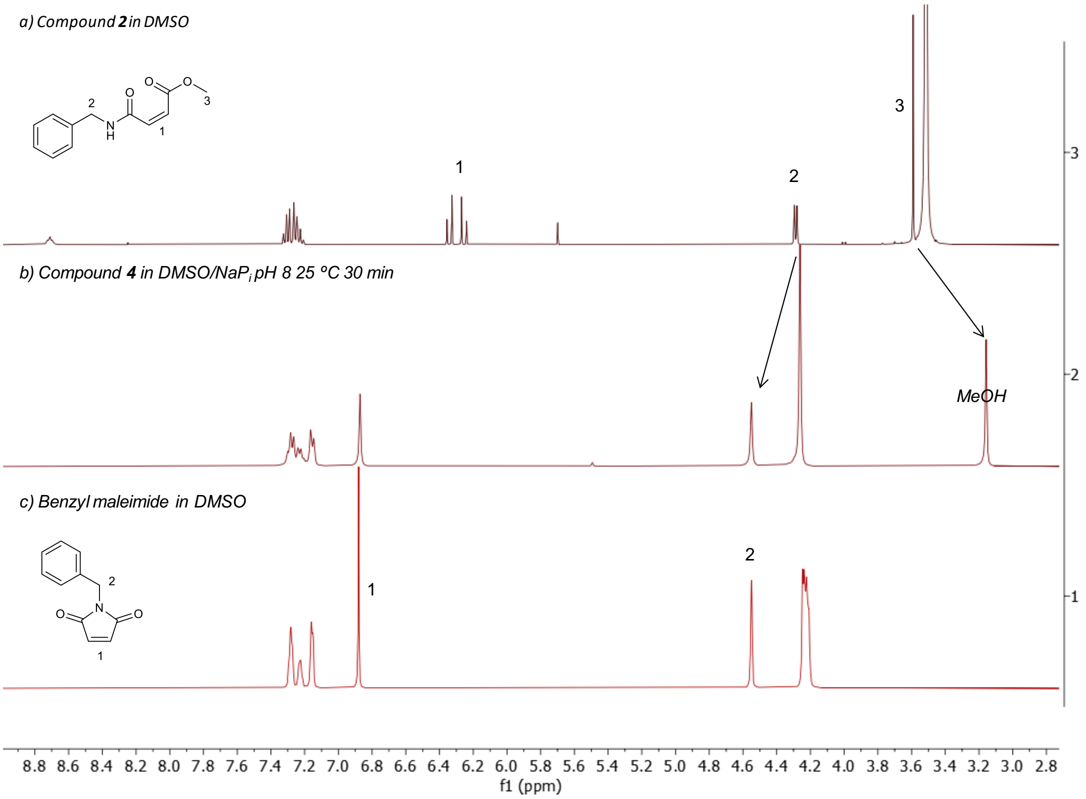

Figure S1. NMR stability study of compound **2**.

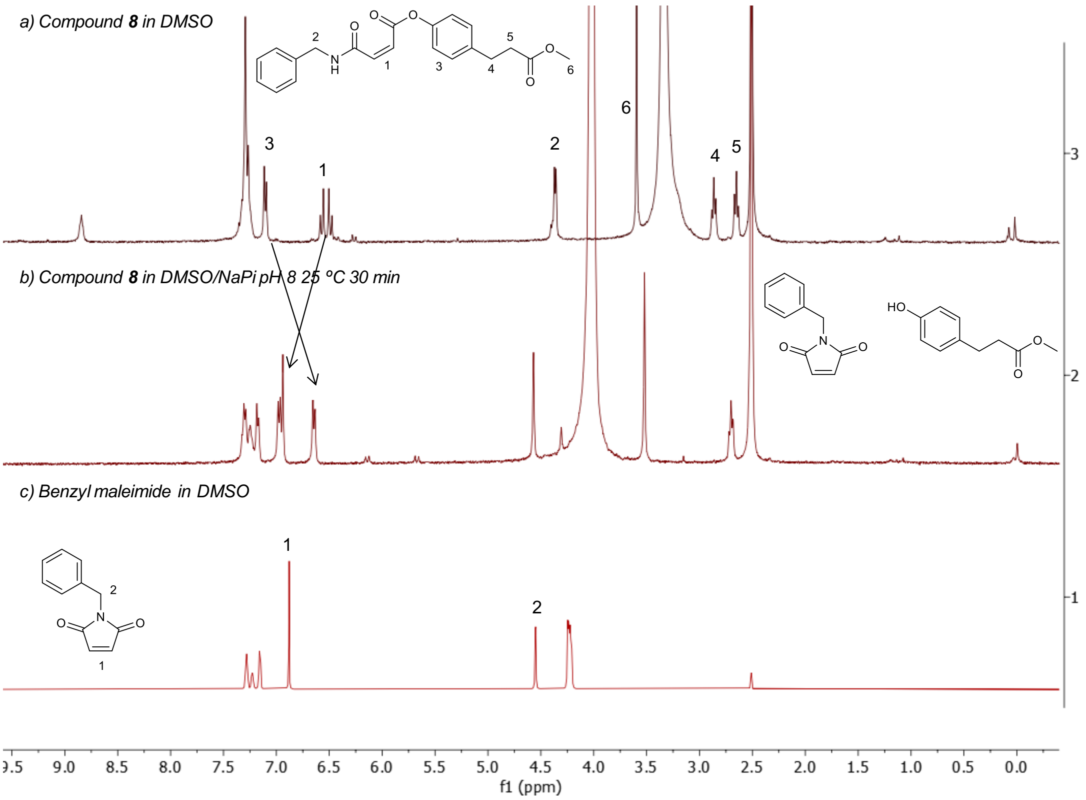

Figure S2. NMR stability study of compound **8**.

Reaction of Compounds 1–10 with BME

A 0.1 M stock solution of compounds **1–10** was prepared in DMSO-d$_6$. Then in an NMR tube 0.1 mL each solution was diluted with another 0.4 mL of DMSO-d$_6$. 0.2 mL of D_2O (NaP$_i$ pH 8, 50 mM) was added and the mixture was sonicated until a clear solution was obtained. 0.1 mL of β-mercaptoethanol (BME) 0.2 M stock solution was then added to the previously prepared solution and kept at 25 ºC for another 30 min. Once the time was over the mixture was analysed by NMR.

BME shows 2 triplets at 2.50 and 3.49 ppm. Dimer formation through oxidation of the thiol to a disulfide species was also observed, 2 triplets at 2.75 and 3.63 ppm (blue circles on spectra). The competing dimer formation is why excess of thiol was added.

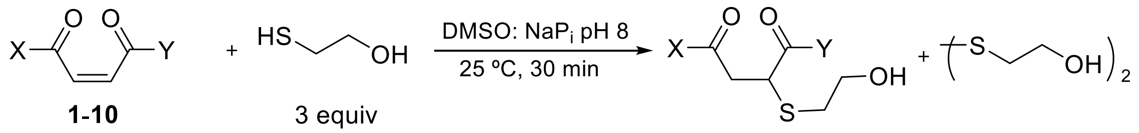

1-10 3 equiv BME **1-10**

Compound	Conversion to BME 1–10 (%)
**1**	0
**2**	99
**3**	0
**4**	50
**5**	99
**6**	99
**7**	0
**8**	99
**9**	99
**10**	0

Table S1. Table showing the conversion of **1–10** to BME **1–10** under the described conditions.

No Michael addition was observed for compounds **1**, **3**, **7** and **10**. The rest of the compounds underwent a Michael addition to form the corresponding thioether. Compounds **2** and **8** also showed complete addition but as the maleimide proved to be formed first in the presence of water and the addition of thiols to maleimides is well documented, it is not included here.

Reaction of BME with compound **4**

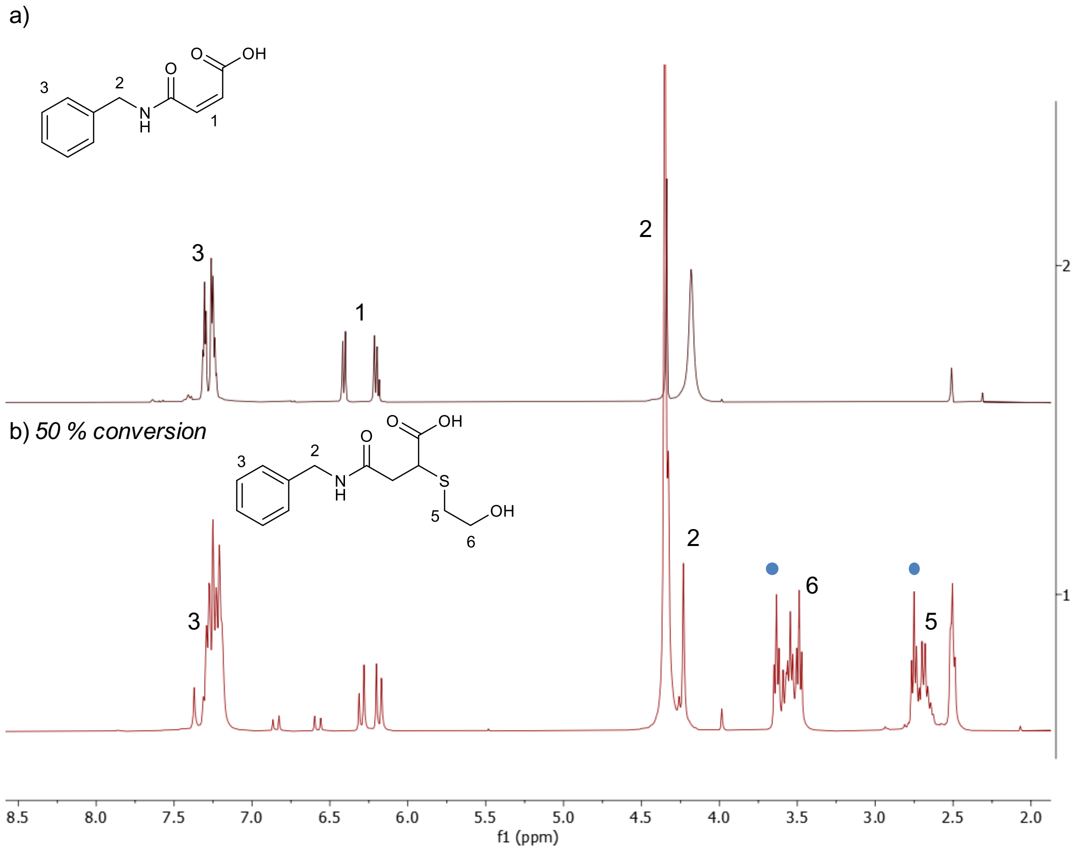

Figure S3. NMR stability study of the reaction of **4** with BME. **a**) Before BME addition; **b**) after addition of BME.

Reaction of BME with compound **5**

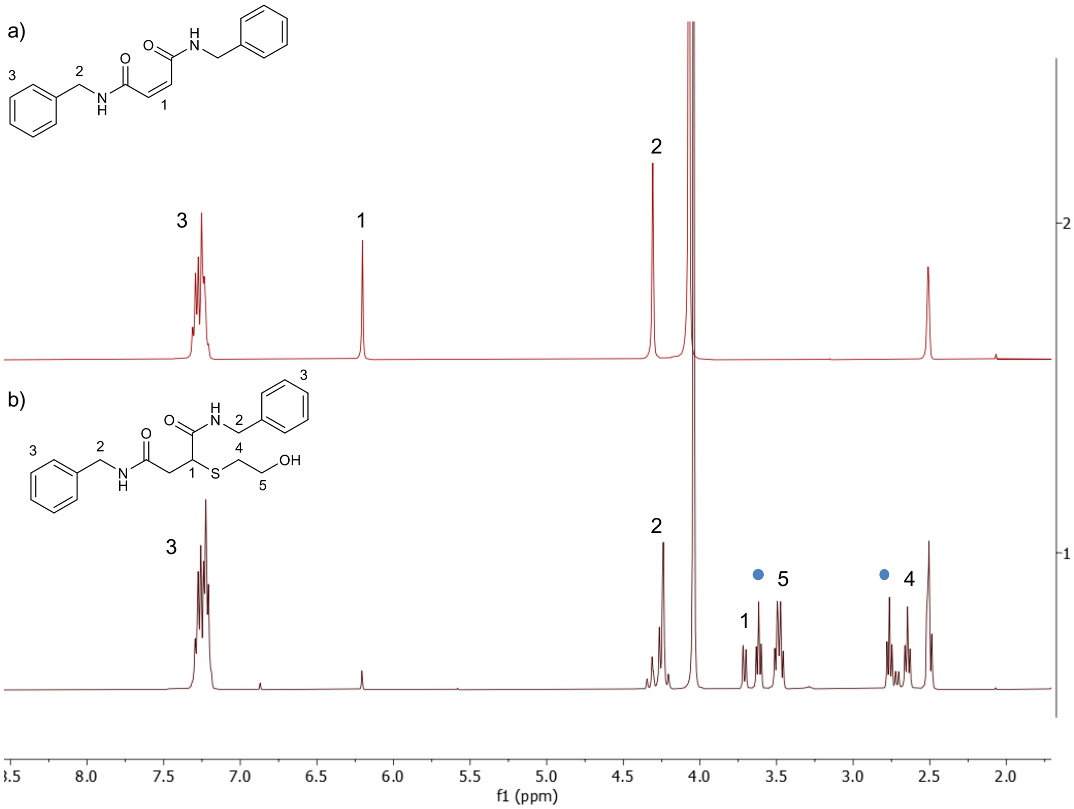

Figure S4. NMR stability study of the reaction of **5** with BME. **a**) Before BME addition; **b**) after addition of BME.

Reaction of BME with compound **6**

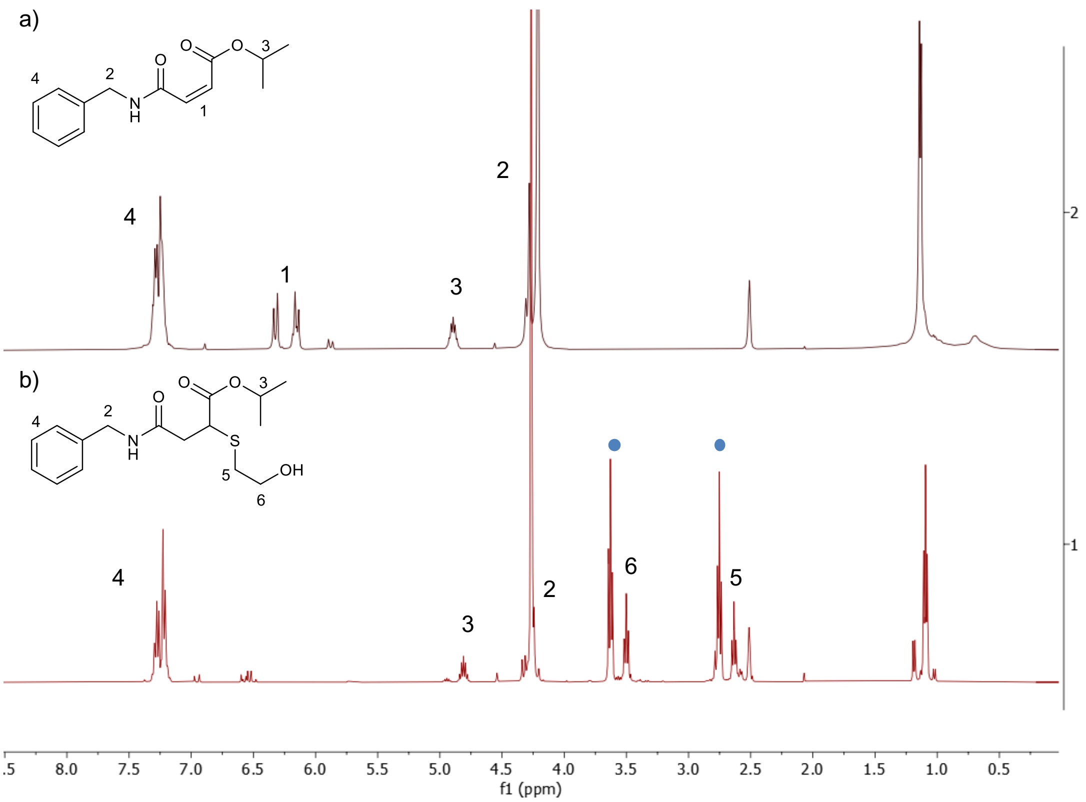

Figure S5. NMR stability study of the reaction of **6** with BME. **a**) Before BME addition; **b**) after addition of BME.

Reaction of BME with compound **9**

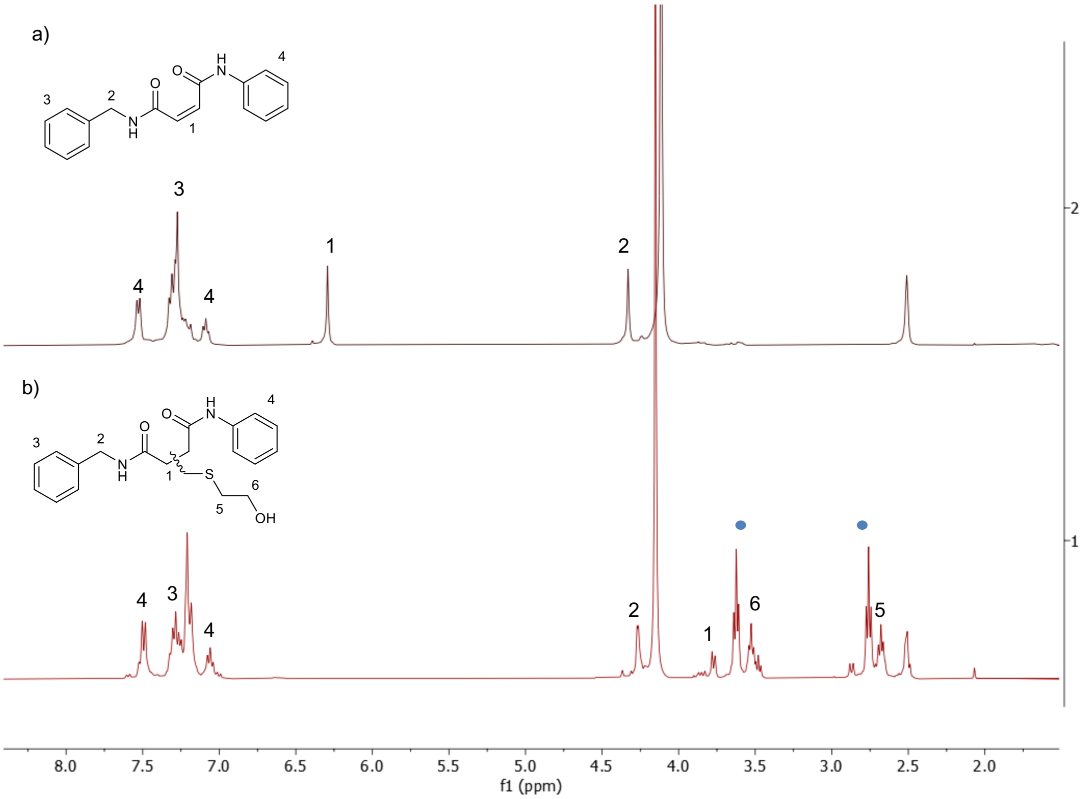

Figure S6. NMR stability study of the reaction of **9** with BME. **a**) Before BME addition; **b**) after addition of BME.

4. Protein Conjugation Experiments

LC–MS was performed on a Waters SQ Detector 2 mass spectrometer coupled to an Acquity UPLC system using an Acquity UPLC BEH C4 column (130Å, 1.7 µm, 2.1 mm X 50 mm). Solvents A, water with 0.1% formic acid and B, 71% acetonitrile, 29% water and 0.075% formic acid were used as the mobile phase at a flow rate of 0.2 mL min^{-1}. The gradient was programmed as follows: 100% A for 2 min, then 100% A to 100% B in 9 min, then 100% B for 5 min, and after that 100% A for 4 min. The electrospray source was operated with a capillary voltage of 3.0 kV and a cone voltage of 30 V. Nitrogen was used as the desolvation gas at a total flow of 800 L h^{-1}. m/z values of positively and negatively charged ions were measured in the mass analyzer, which was scanned between m/z 100–2000 for the generation of mass spectra. The major peak(s) were selected for integration and analyzed using MassLynx software (v. 4.1 from Waters).

C2Am was provided by Dr. A. Neves and Prof. K. Brindle;[5] Ubiquitin–K63C was expressed and purified as previously described;[6] HET nanobody was a gift from the Vendruscolo group,[7] HSA was kindly provided by Albumedix; R434 was expressed following reported procedures;[8] H3K4C was expressed as described below; and Gemtuzumab, Trastuzumab and Fc fragment antibodies were kindly donated by AstraZeneca.

Note: All antibodies and Fc fragments need to be decapped using a reducing agent before cysteine conjugation.

Decapping protocol: TCEP solution (10 mM, 20 equiv) was added and the protein solution and stirred for 1 h at 25 ºC. After this the small molecules were removed from the solution using a desalting column and a 10 mM solution of dehydroascorbic acid (20 equiv) in DMF was added, the solution is then stirred at 25 ºC for 3 h. After this time, the small molecules are removed using desalting Zeba columns.

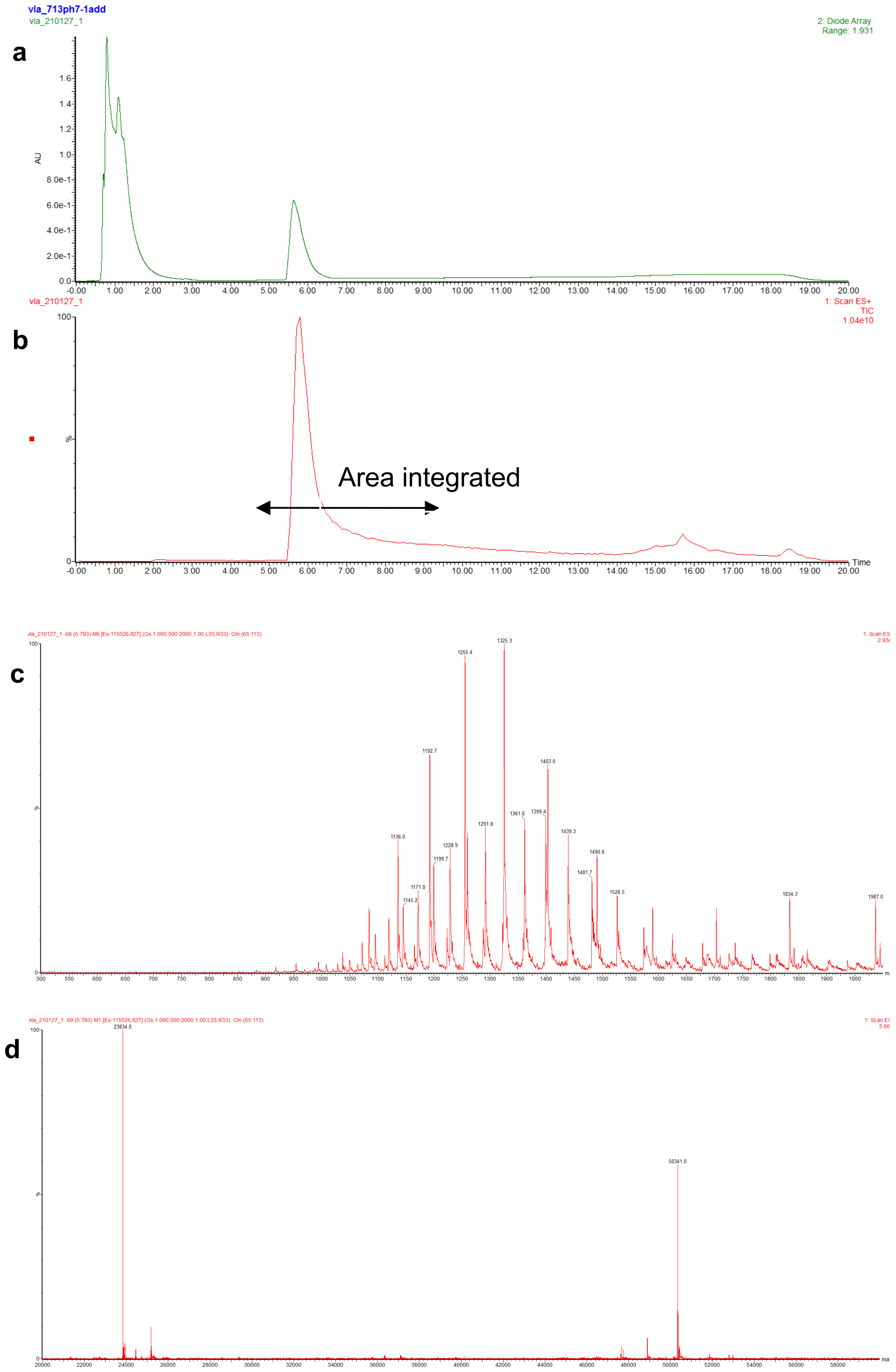

Figure S7. A typical analysis of a conjugation reaction by LC–MS is described for the reaction of Gemtuzumab with TCEP. The main peak in the UV trace (**a**) and the total ion chromatogram (**b**) is integrated, obtaining the combined ion series (**c**) from which one can obtain a deconvoluted spectra (**d**). Identical analyses were carried out for all the conjugation reactions performed in this work. UV traces have not been included in the conjugation experiments as no

separation can be seen between different species so no information can be obtained from it.

Free Cys Containing Proteins and Peptides

Ubiquitin–K63C

Sequence:
SAQIFVKTLTGKTITLEVEPSDTIENVKAKIQDKEGIPPDQQRLIFAGKQLEDGR
TLSDYNIQCESTLHLVLRLRGG

Calculated Isotopically Averaged Molecular Weight = 8565 Da

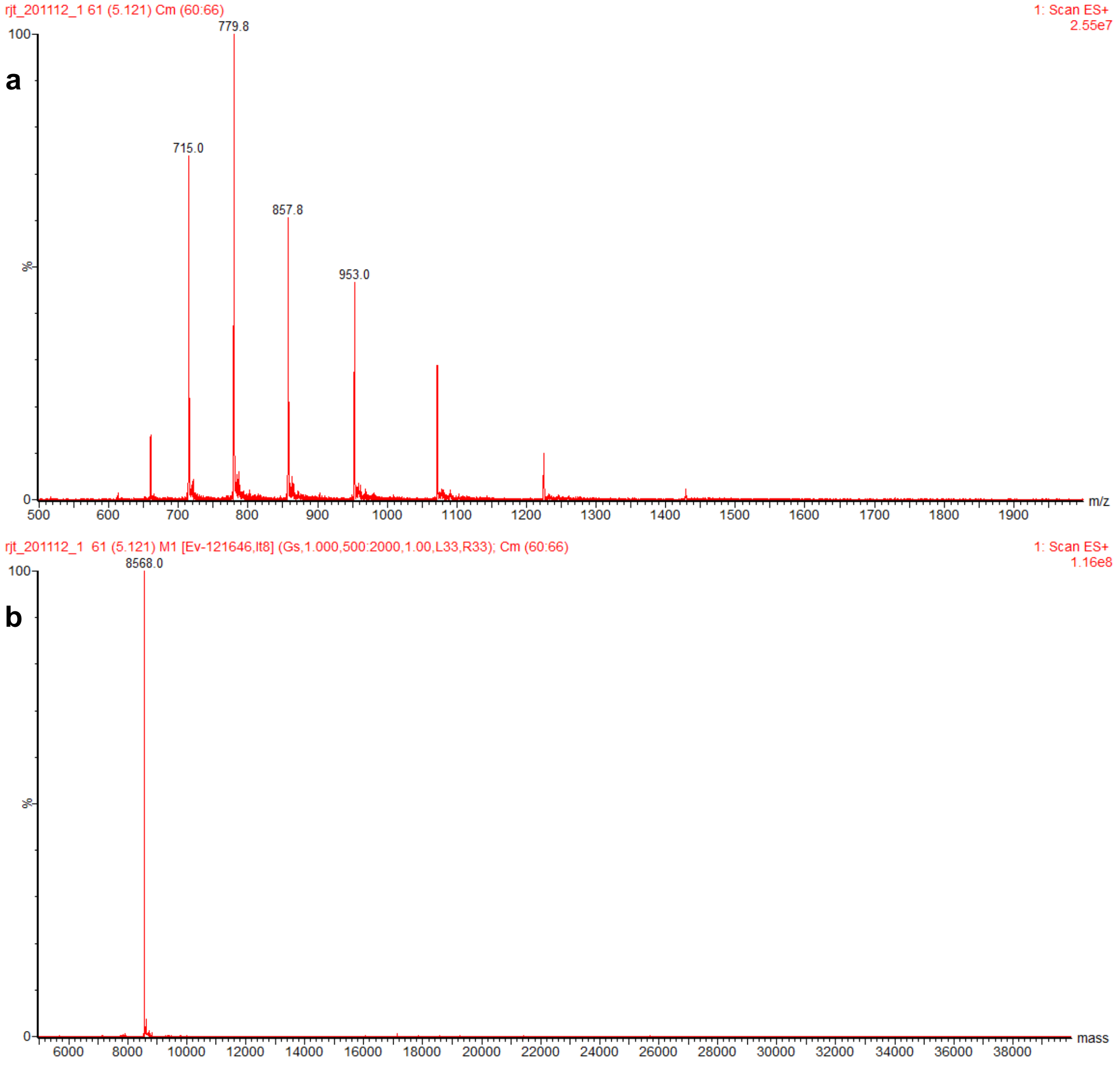

Figure S8. LC–MS spectra of Ub-K63C; **a**) ion series and **b**) deconvoluted spectrum.

Human Serum Albumin (HSA)

Sequence:
DAHKSEVAHRFKDLGEENFKALVLIAFAQYLQQCPFEDHVKLVNEVTEFAKTC
VADESAENCDKSLHTLFGDKLCTVATLRETYGEMADCCAKQEPERNECFLQH
KDDNPNLPRLVRPEVDVMCTAFHDNEETFLKKYLYEIARRHPYFYAPELLFFA
KRYKAAFTECCQAADKAACLLPKLDELRDEGKASSAKQRLKCASLQKFGERA
FKAWAVARLSQRFPKAEFAEVSKLVTDLTKVHTECCHGDLLECADDRADLAK
YICENQDSISSKLKECCEKPLLEKSHCIAEVENDEMPADLPSLAADFVESKDVC
KNYAEAKDVFLGMFLYEYARRHPDYSVVLLLRLAKTYETTLEKCCAAADPHEC
YAKVFDEFKPLVEEPQNLIKQNCELFEQLGEYKFQNALLVRYTKKVPQVSTPT
LVEVSRNLGKVGSKCCKHPEAKRMPCAEDYLSVVLNQLCVLHEKTPVSDRVT
KCCTESLVNRRPCFSALEVDETYVPKEFNAETFTFHADICTLSEKERQIKKQTA
LVELVKHKPKATKEQLKAVMDDFAAFVEKCCKADDKETCFAEEGKKLVAASQ
AALGL

Calculated Isotopically Averaged Molecular Weight = 66445 Da

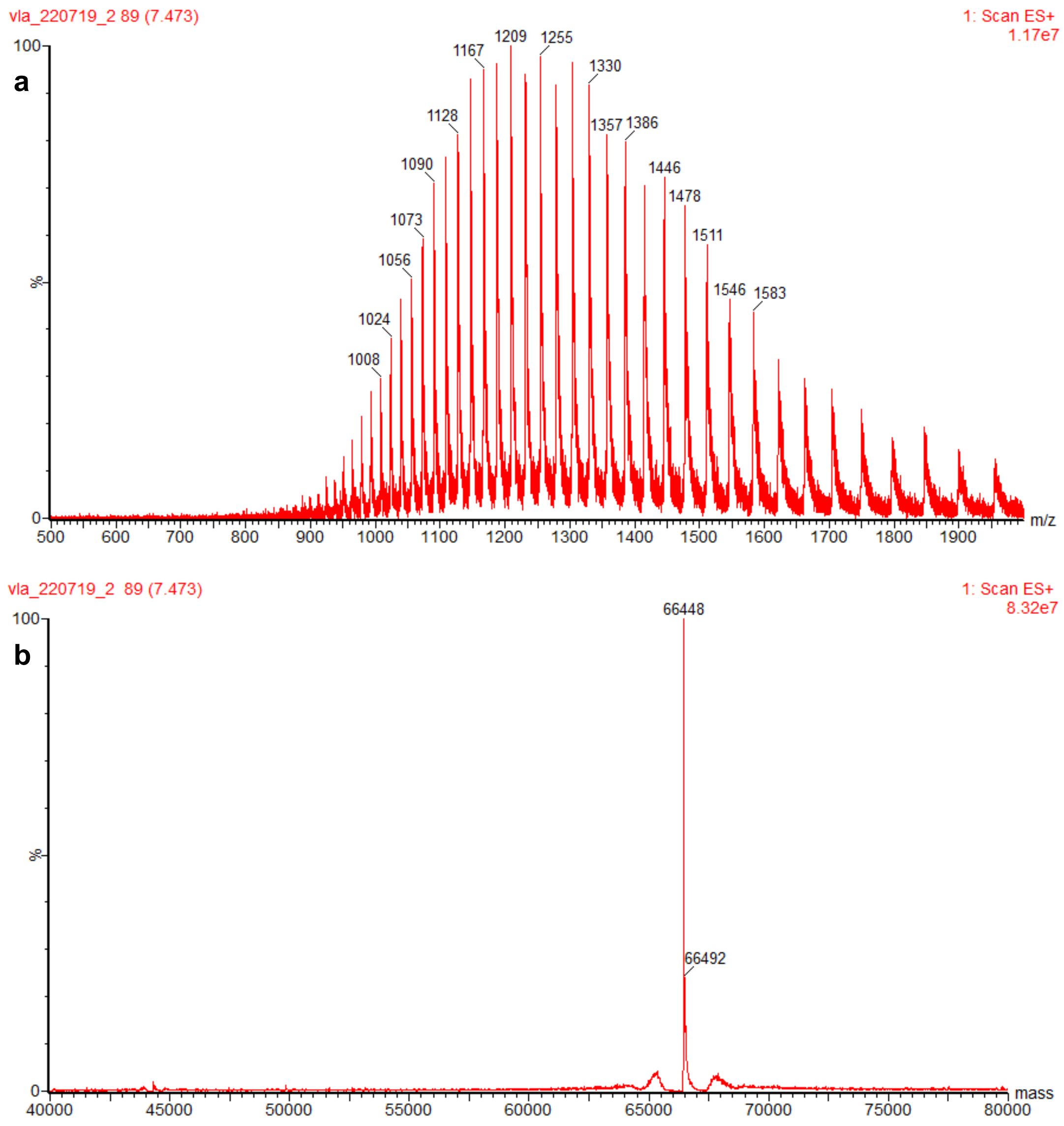

Figure S9. LC–MS spectra of HSA; **a**) ion series and **b**) deconvoluted spectrum.

C2Am–Cys95

Sequence:
GSPGISGGGGGILDSMVEKLGKLQYSLDYDFQNNQLLVGIIQAAELPALDMGG
TSDPYVKVFLLPDKKKKFETKVHRKTLNPVFNEQFTFKVPYCELGGKTLVMAV
YDFDRFSKHDIIGEFKVPMNTVDFGHVTEEWRDLQSAEK

Calculated Isotopically Averaged Molecular Weight = 16222 Da

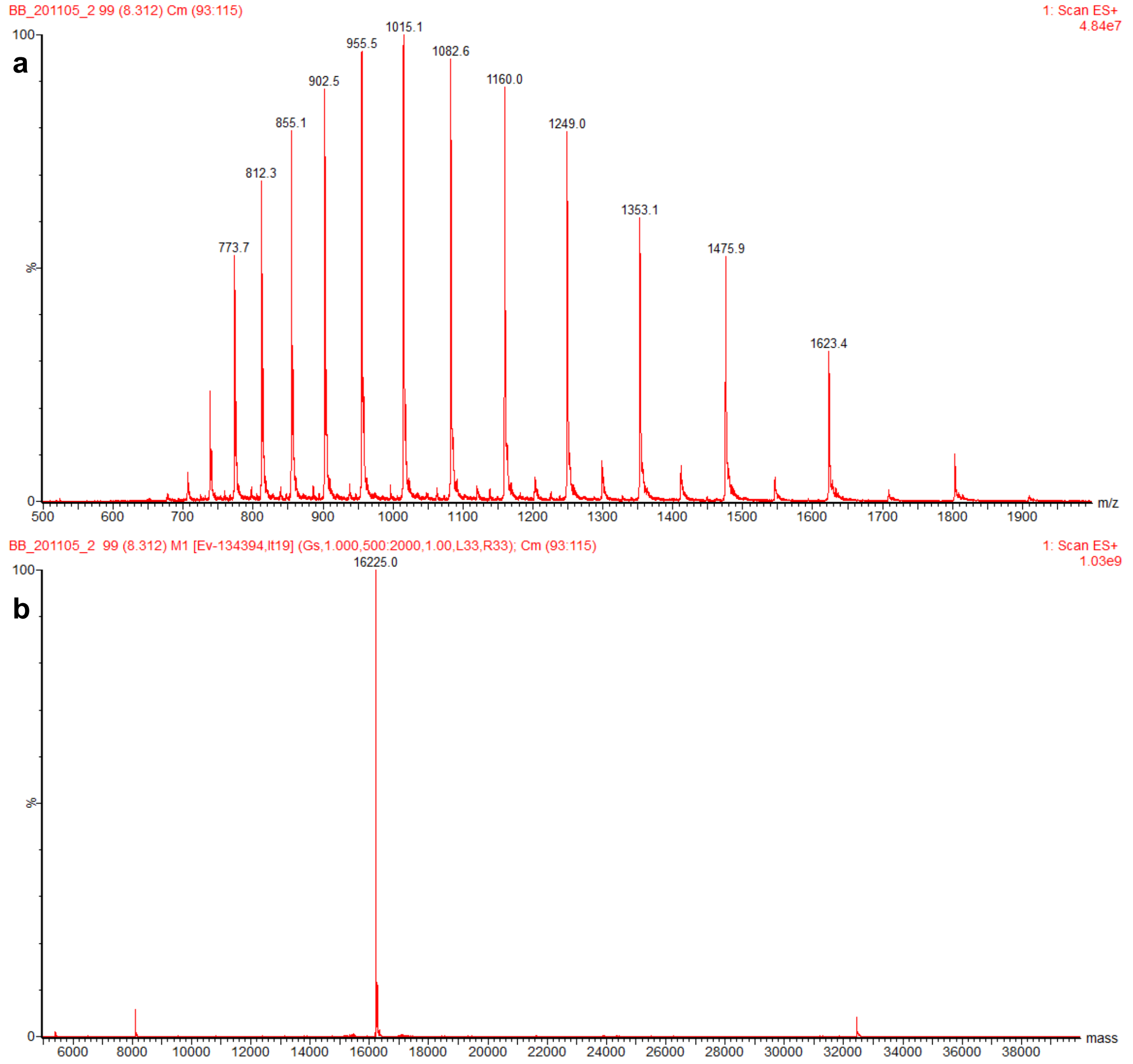

Figure S10. LC–MS spectra of C2Am; **a**) ion series and **b**) deconvoluted spectrum.

R434 G26C

Sequence:
MSISSRVKSKRIQLGLNQAELAQKVCTTQQSIEQLENGKTKRPRFLPELASALG
VSVDWLLNGTSDSNVR

Calculated Isotopically Averaged Molecular Weight = 8010 Da

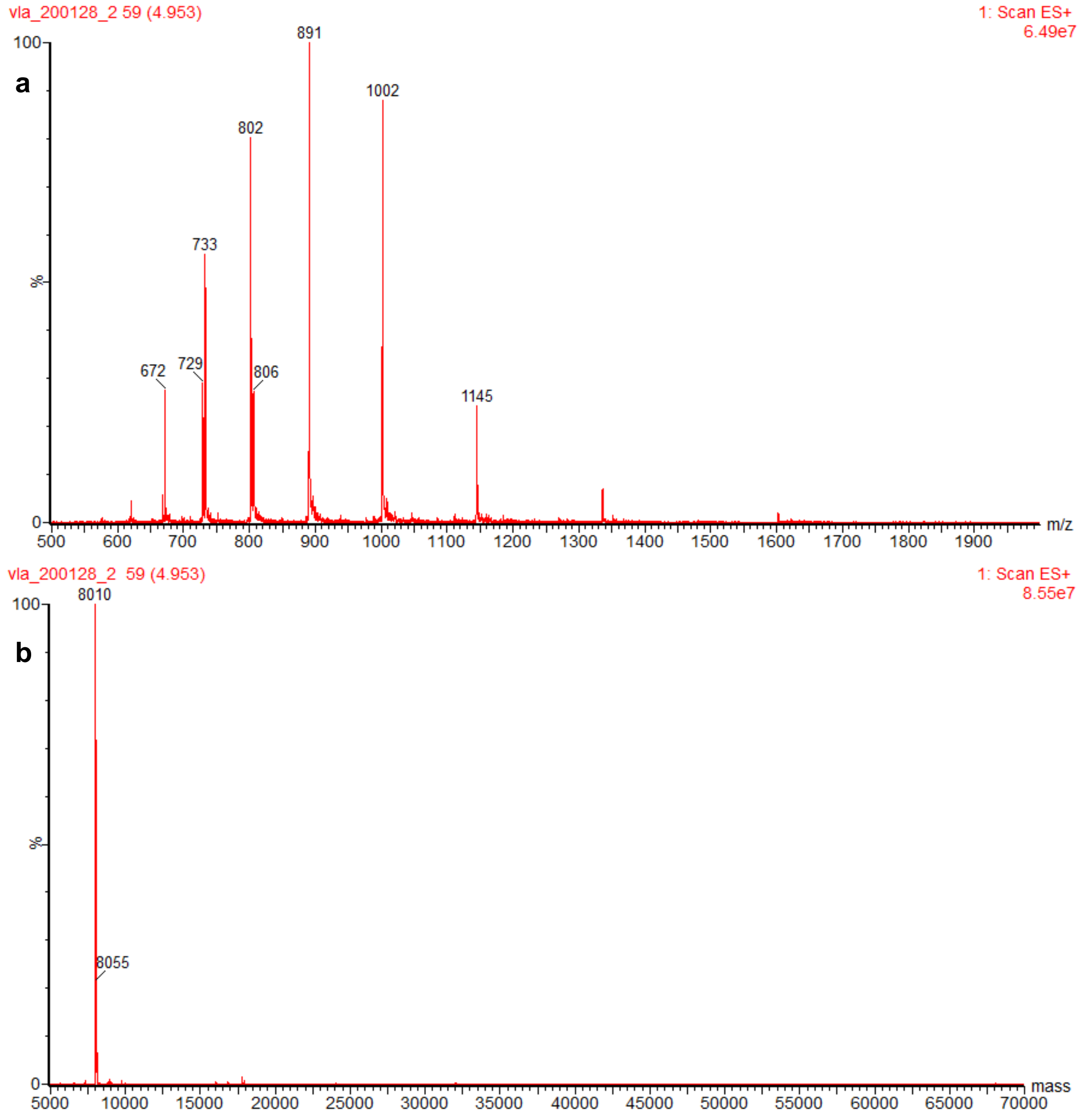

Figure S11. LC–MS spectra of R434; **a**) ion series and **b**) deconvoluted spectrum.

Nanobody HET

Sequence:
MRGSHHHHHHGMASMTGGQQMGRDLYDDDDKDPKLEVQLVESGGGLVQP
GGSLRLSCAASGFNIKDTYIGWVRRAPGKGKEWVASIYPTNGYTRYADSVKG
RFTISADTSKNTAYLQMNSLRAEDTAVYYCAAGSH**C**TLTLREEEAAAWGQGT
LVTVSSGT

Calculated Isotopically Averaged Molecular Weight = 17401 Da

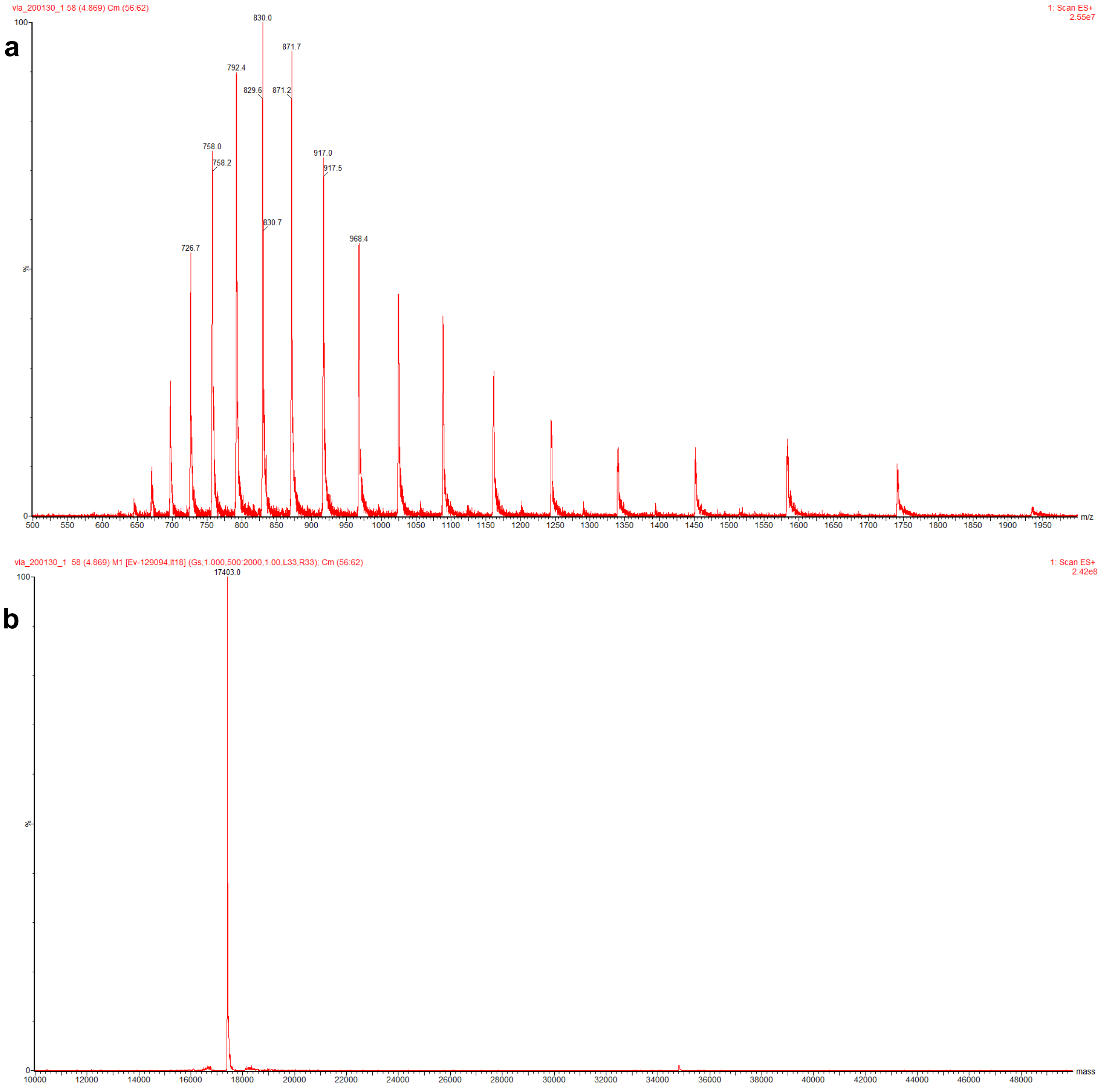

Figure S12. LC–MS spectra of HET; **a**) ion series and **b**) deconvoluted spectrum.

Histone 3 K4C (H3K4C)

Recombinant expression and purification of mutant histones from inclusion bodies: The pET-3a plasmid encoding histone H3 (C110A) from *Xenopus laevis* was the kind gift of Professor Ernest Laue, Department of Biochemistry, University of Cambridge. The K4C mutant was constructed by site-directed mutagenesis using the NZYMutagenesis kit (NZYTech) according to the manufacturer's instructions. Primers were obtained from Sigma and the mutation was confirmed by sequencing. The following primer sequences were used to introduce the K4C mutation in the pET-3a-H3 plasmid by PCR:

5'- ATGGCCCGTACCTGCCAGACCGCCCGT-3'
5'- ACGGGCGGTCTGGCAGGTACGGGCCAT-3'

The mutant histone protein H3K4C was recombinantly expressed in *E. coli BL21(DE3)* cells following bacterial transformation with its expression plasmid. Cells were grown at 37 ºC and 220 rpm in LB containing carbenicillin (100 µg/mL) until the optical density reached 0.5–0.7. Protein expression was then induced with 1 mM IPTG for 2 h at 37 ºC and 220 rpm. Cells were harvested by centrifugation at 7000 rpm and 4 ºC and stored at –80 ºC. Protein expression was confirmed by SDS-PAGE.

Histones were purified by anion and cation exchange chromatography in tandem, essentially as described by Klinker *et al.*[9] Briefly, the bacterial pellet was resuspended in SAU buffer (40 mM NaOAc pH 5.2, 7 M urea, 10 mM lysine, 1 mM EDTA pH 8.0, 5 mM β-mercaptoethanol) containing 200 mM NaCl, protease inhibitors and DNase. Defined buffer conditions were achieved by directly adding to the pellet DNase I (Roche) and protease inhibitors (Roche) in powder, followed by the addition of 10x SAU buffer (400 mM NaOAc pH 5.2, 10 mM EDTA pH 8.0, 100 mM lysine), β-mercaptoethanol and NaCl to a final concentration of 5 mM and 200 mM, respectively. Once the cells were resuspended, urea was added to a concentration of 7M and the suspension filled up to its final volume with water. All steps during lysis were performed on ice.

The suspension was sonicated on ice with an amplitude of 20% using 20 pulses of 15 seconds, each followed by a pause of 30 seconds. The extract was cleared by centrifugation at 40000g and 4 ºC for 30 min. The resulting supernatant was filtered and loaded onto a HiTrap Q HP column (GE Healthcare, 5 ml) stacked on top of a HiTrap SP HP column (GE Healthcare, 1 ml) pre-equilibrated with SAU-200 and connected to an FLPC system (ÄKTA, GE Healthcare). When the extract had passed completely through the Q column, the latter was removed from the FPLC system and the SP column washed with 200 mM NaCl for several column volumes. Histones were then

eluted with a NaCl gradient. Histone-containing fractions were pooled, buffer-exchanged to Tris (15 mM, pH 7.5) with Amicon Ultra-15 mL centrifugal filter units (Merck Millipore) and lyophilized.

Sequence:
ARTCQTARKSTGGKAPRKQLATKAARKSAPATGGVKKPHRYRPGTVALREIR
RYQKSTELLIRKLPFQRLVREIAQDFKTDLRFQSSAVMALQEASEAYLVGLFED
TNLAAIHAKRVTIMPKDIQLARRIRGERA

Calculated Isotopically Averaged Molecular Weight = 15216 Da

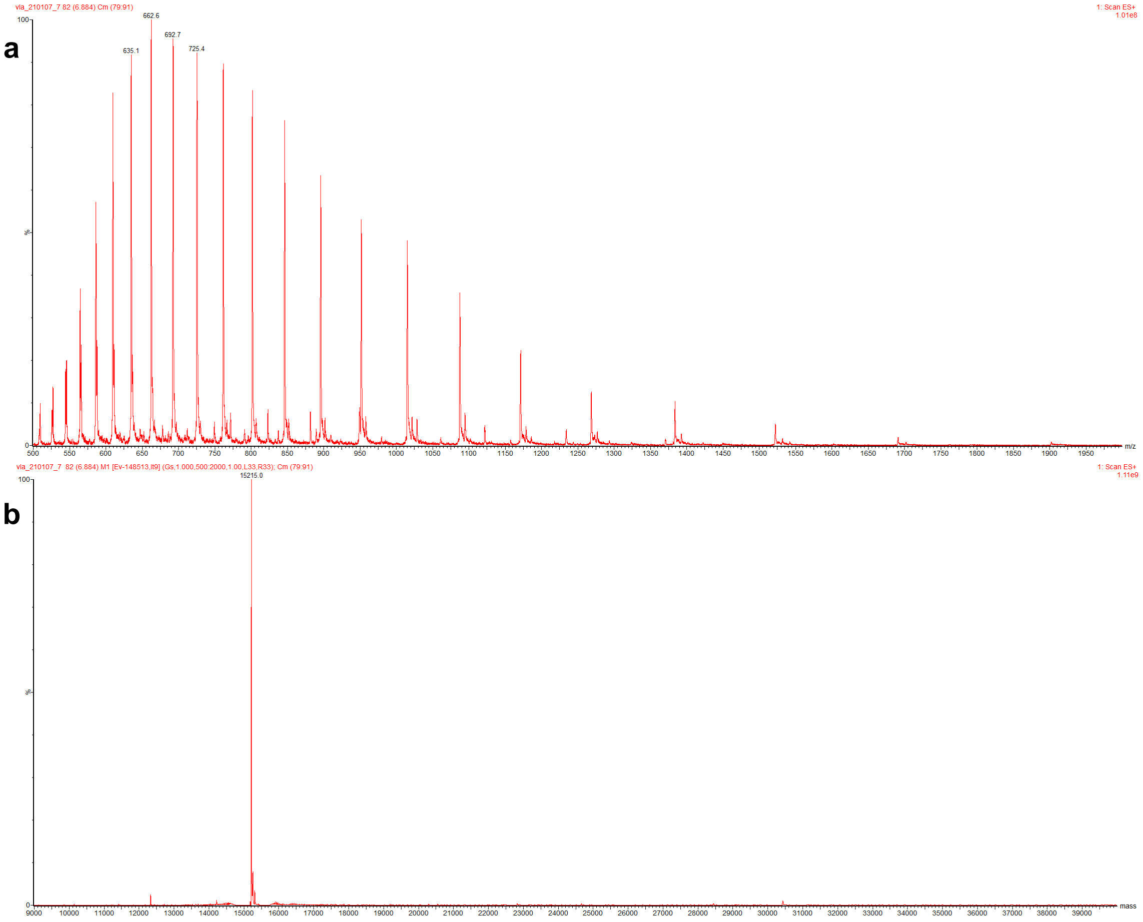

Figure S13. LC–MS spectra of H3K4C; **a**) ion series and **b**) deconvoluted spectrum.

Gemtuzumab V205C

LC–MS was measured after treating protein with 10 equiv of TCEP at 25 ºC for 1 h.
Calculated Isotopically Averaged Molecular Weight Light Chain = 23829 Da, Heavy chain 50349 Da

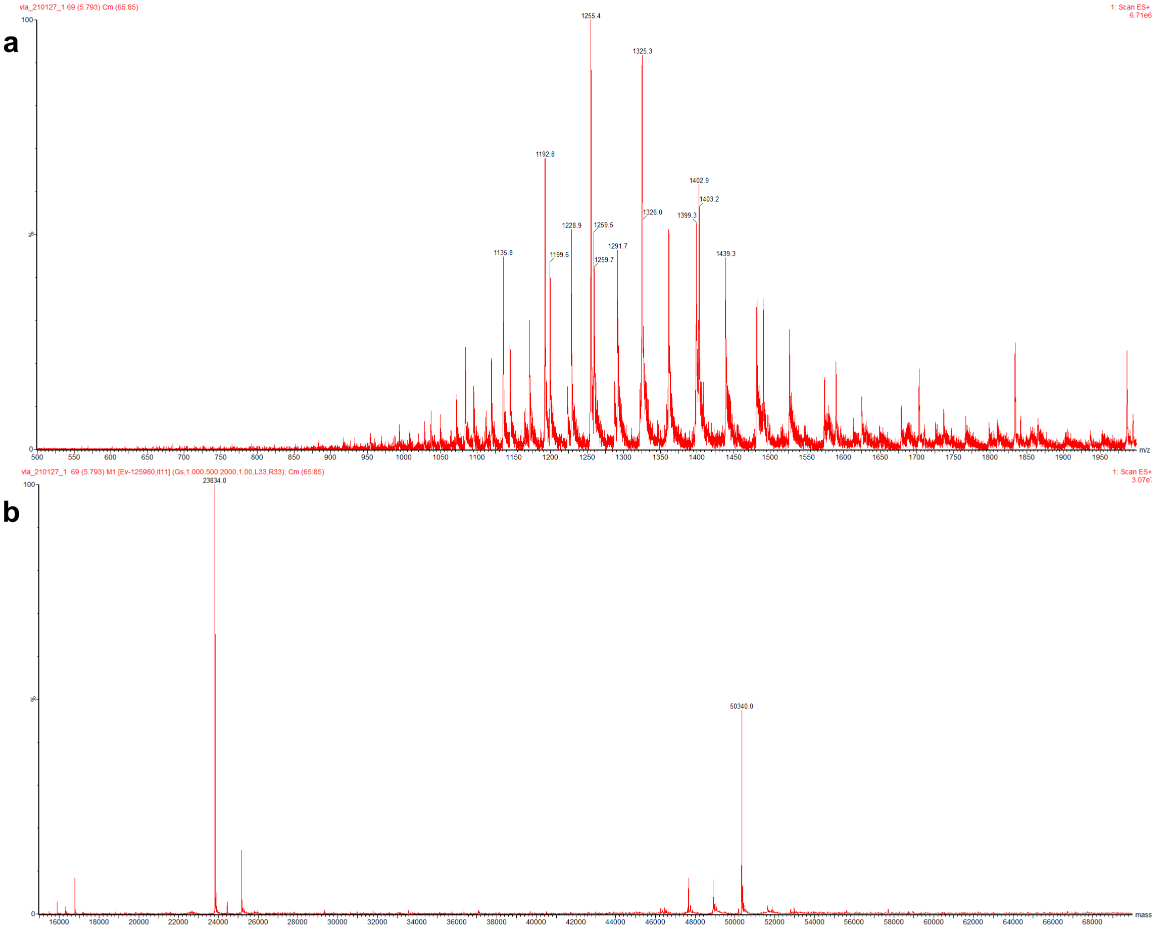

Figure S14. LC–MS spectra of reduced Gemtuzumab V205C; **a**) ion series and b) deconvoluted spectrum.

ASCATN

Calculated Isotopically Averaged Molecular Weight: 602 Da

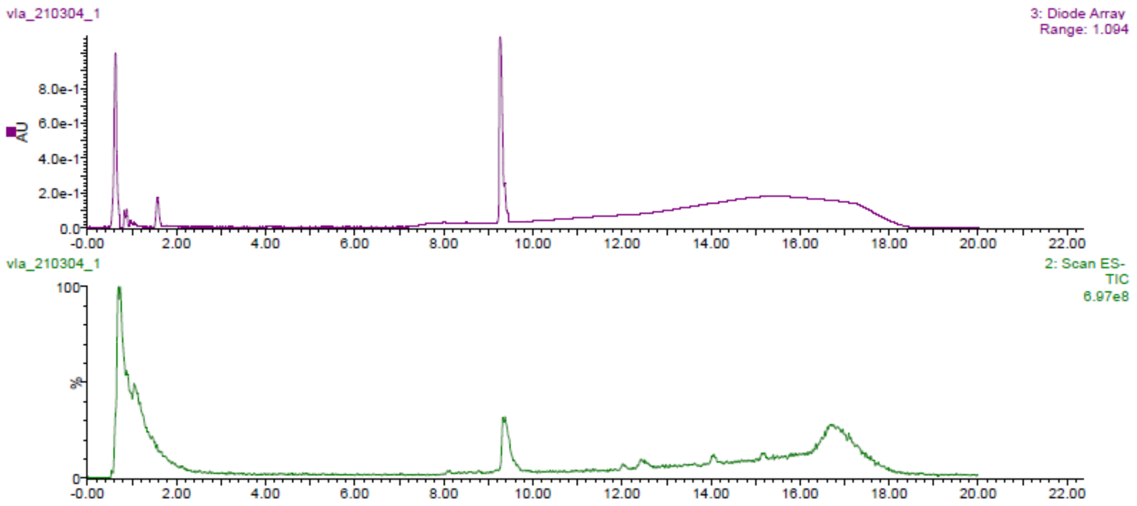

Figure S15. LC–MS UV trace and negative ion scan of ASCATN.

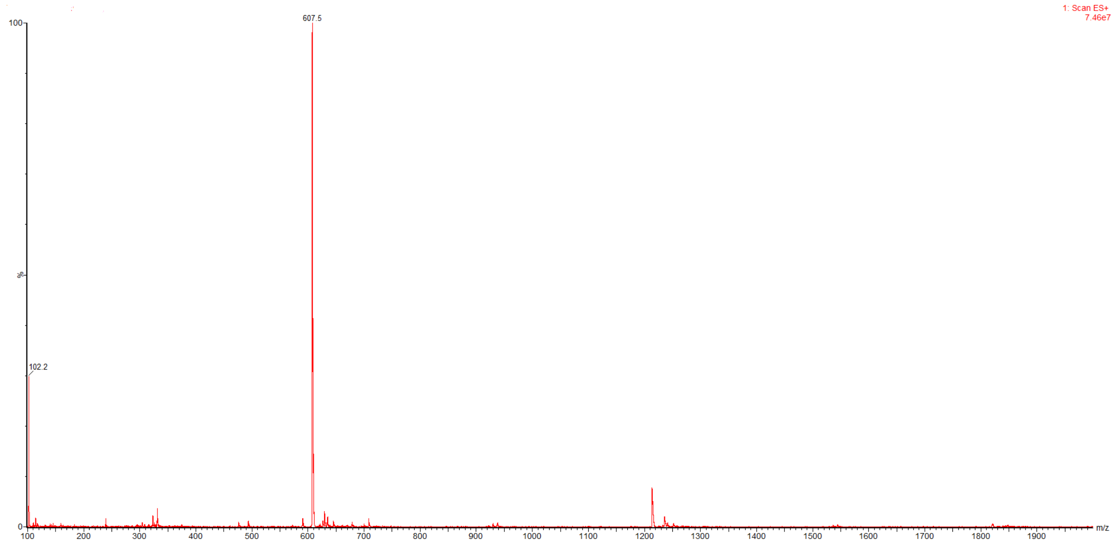

Figure S16. Ion series of main peak at 9.4 min of ASCATN.

Trastuzumab V205C

LC–MS was measured after treating protein with 10 equiv of TCEP at 25 ºC for 1 h.

Calculated Isotopically Averaged Molecular Weight Light Chain = 23445 Da, Heavy chain 50603 Da

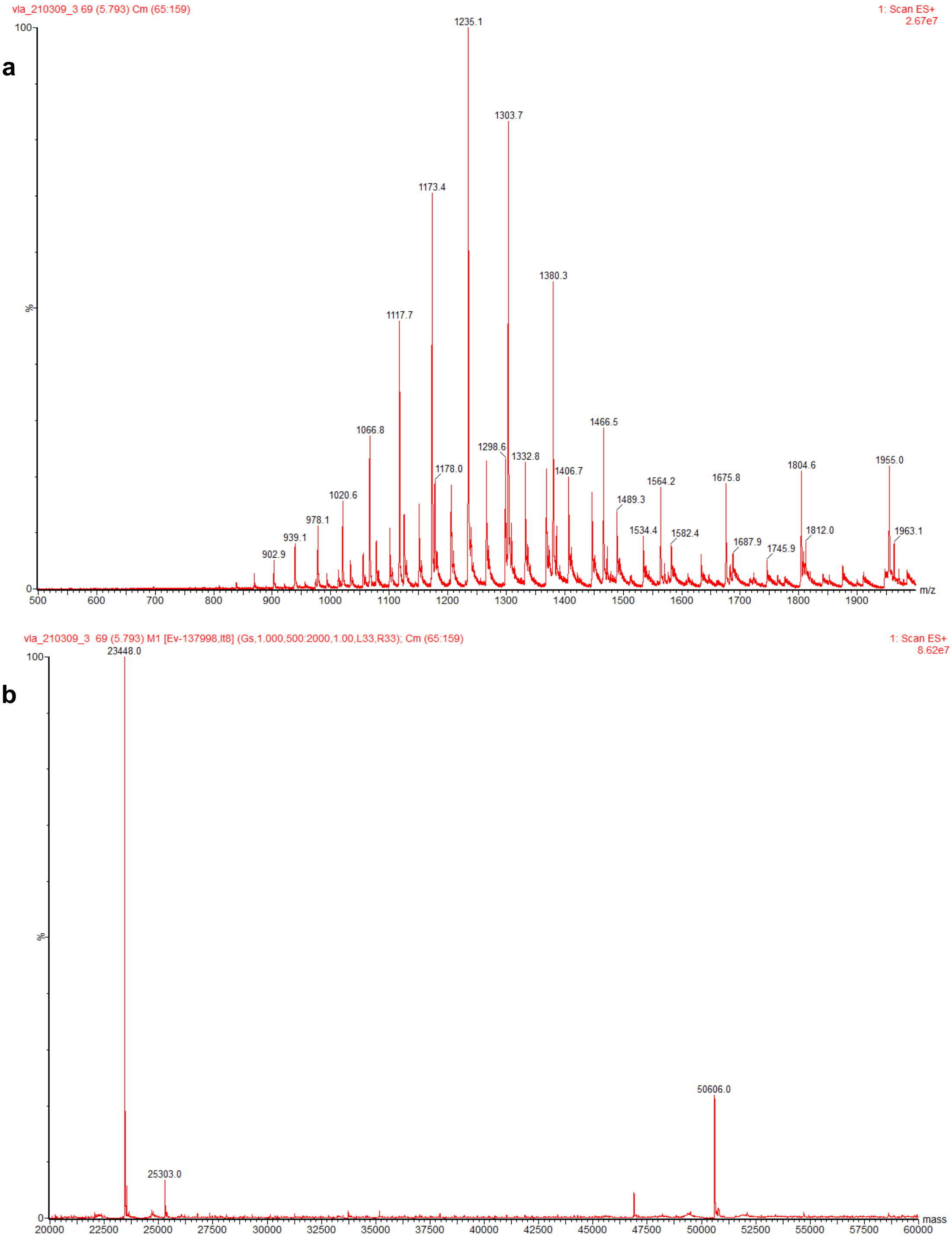

Figure S17. LC–MS spectra of reduced Trastuzumab V205C; **a**) ion series and **b**) deconvoluted spectrum.

Trastuzumab V205C K207A

LC–MS was measured after treating protein with 10 equiv of TCEP at 25 °C for 1 h.

Calculated Isotopically Averaged Molecular Weight Light Chain = 23390 Da, Heavy chain 50631

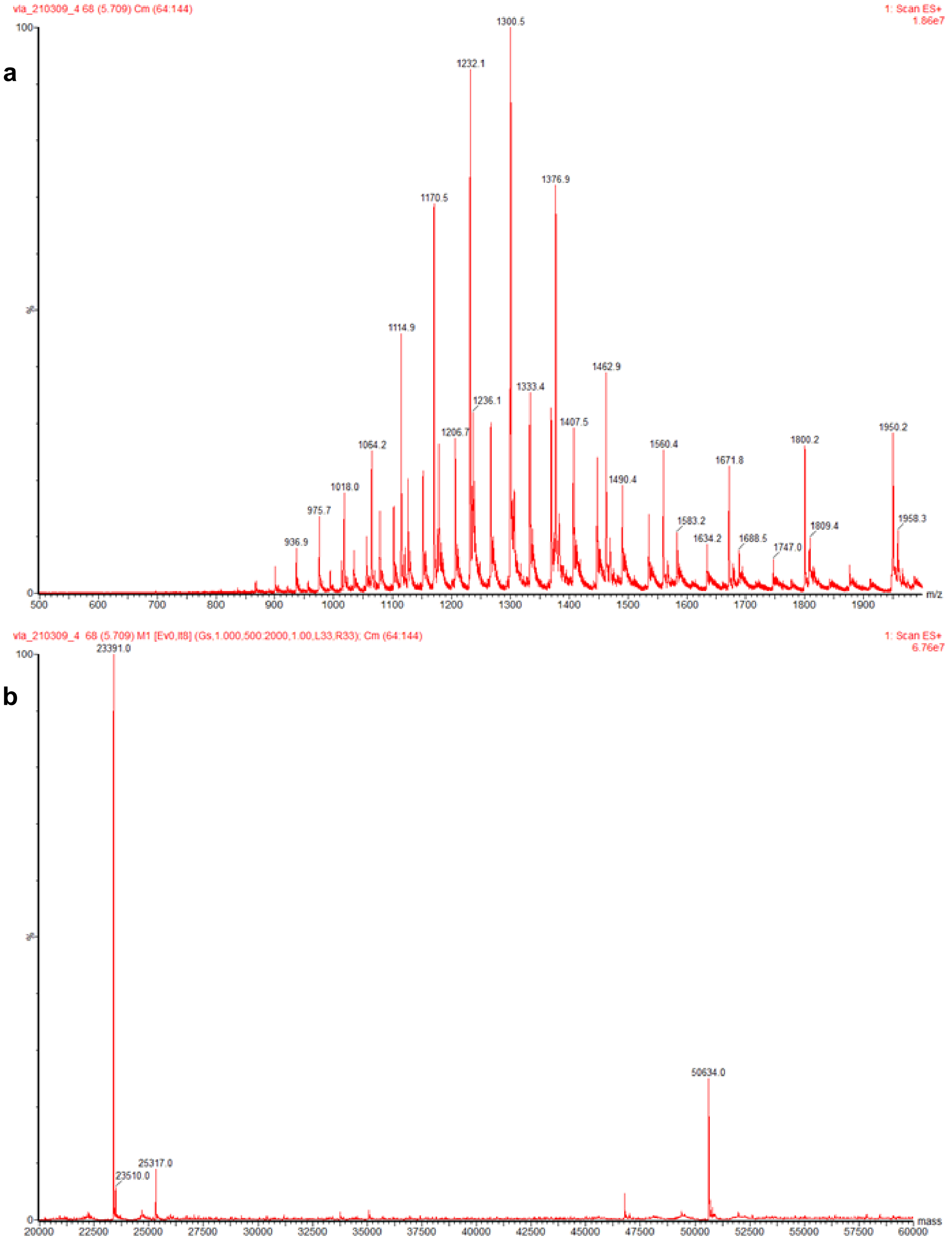

Figure S18. LC–MS spectra of reduced Trastuzumab V205C K207C; **a**) ion series and **b**) deconvoluted spectrum.

Fc 239iC

LC–MS was measured after deglycosilating protein with a Gibco PNG-ase F deglycosylation kit and treating protein with 10 equiv of TCEP at 25 ºC for 1 h. For all Fc fragments an additional peak +948 is also observed which corresponds to an *O*-glycosylation which the kit does not remove.

Sequence:
THTCPPCPAPEFEGGPSCVFLFPPKPKDTLMISRTPEVTCVVVDVSHEDPEVK
FNWYVDGVEVHNAKTKPREEQYNSTYRVVSVLTVLHQDWLNGKEYKCKVSN
KALPASIEKTISKAKGQPREPQVYTLPPSREEMTKNQVSLTCLVKGFYPSDIAV
EWESNGQPENNYKTTPPVLDSDGSFFLYSKLTVDKSRWQQGNVFSCSVMHE
ALHNHYTQKSLSLSPG

Calculated Isotopically Averaged Molecular Weight: 25350 Da

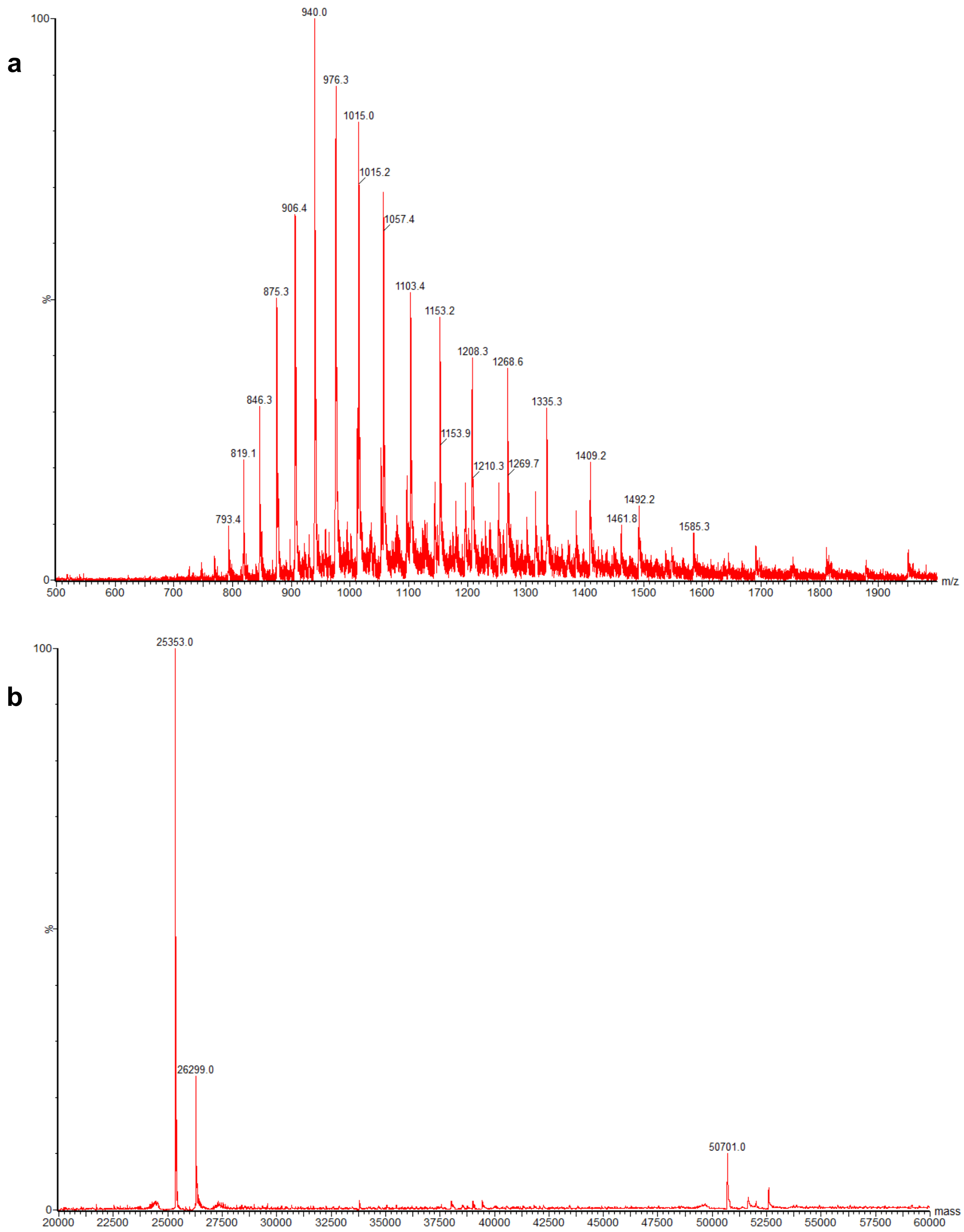

Figure S19. LC–MS spectra of reduced Fc 239iC; **a**) ion series and **b**) deconvoluted spectrum.

Fc 268C

Sequence:
THTCPPCPAPEFEGGPSVFLFPPKPKDTLMISRTPEVTCVVVDVSCEDPEVKF
NWYVDGVEVHNAKTKPREEQYNSTYRVVSVLTVLHQDWLNGKEYKCKVSNK
ALPASIEKTISKAKGQPREPQVYTLPPSREEMTKNQVSLTCLVKGFYPSDIAVE
WESNGQPENNYKTTPPVLDSDGSFFLYSKLTVDKSRWQQGNVFSCSVMHEA
LHNHYTQKSLSLSPG

Calculated Isotopically Averaged Molecular Weight: 25213 Da

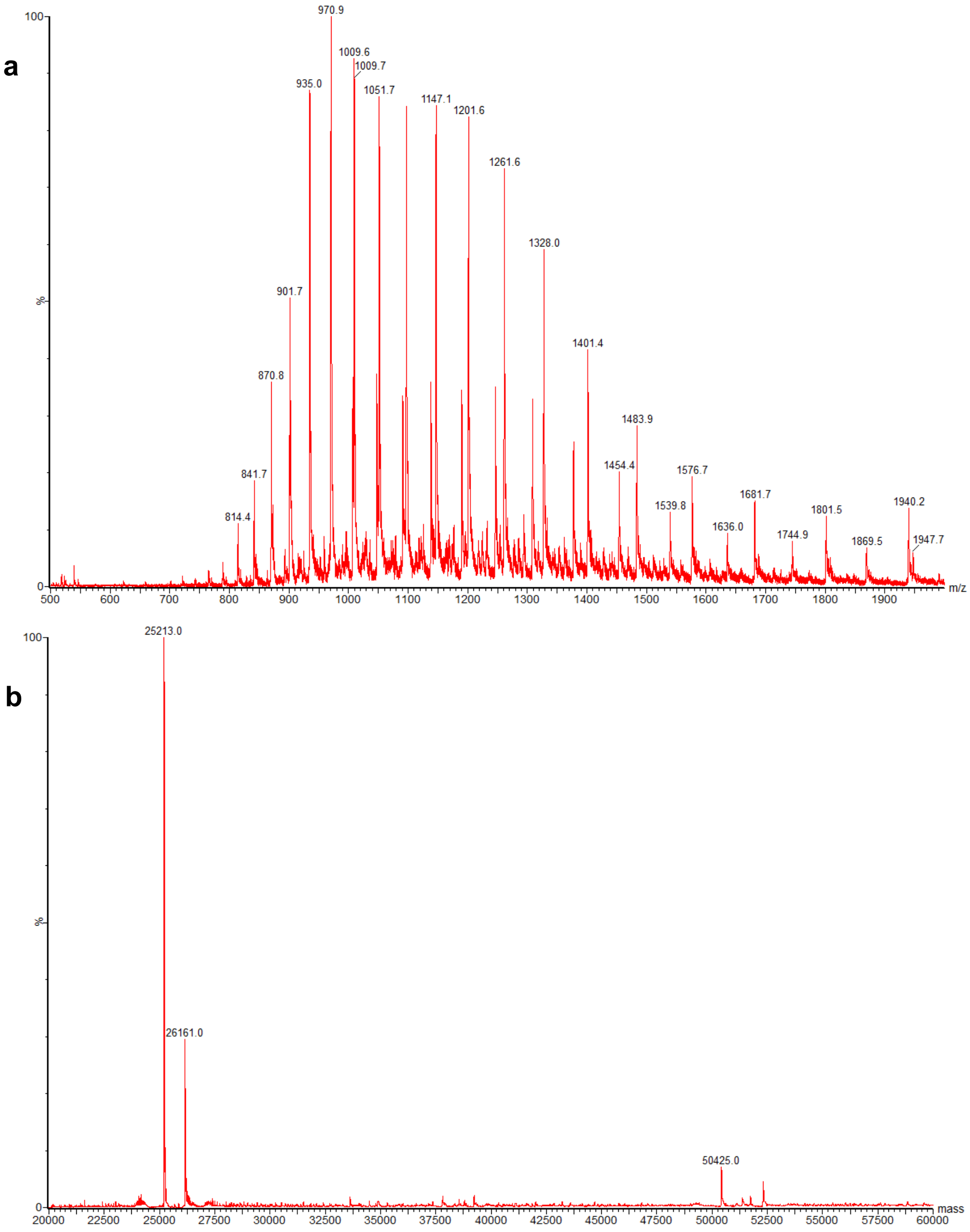

Figure S20. LC–MS spectra of reduced Fc 268C; **a**) ion series and **b**) deconvoluted spectrum.

Fc 274C

Sequence:
THTCPPCPAPEFEGGPSVFLFPPKPKDTLMISRTPEVTCVVVDVSHEDPEVCF
NWYVDGVEVHNAKTKPREEQYNSTYRVVSVLTVLHQDWLNGKEYKCKVSNK
ALPASIEKTISKAKGQPREPQVYTLPPSREEMTKNQVSLTCLVKGFYPSDIAVE
WESNGQPENNYKTTPPVLDSDGSFFLYSKLTVDKSRWQQGNVFSCSVMHEA
LHNHYTQKSLSLSPG

Calculated Isotopically Averaged Molecular Weight: 25222 Da

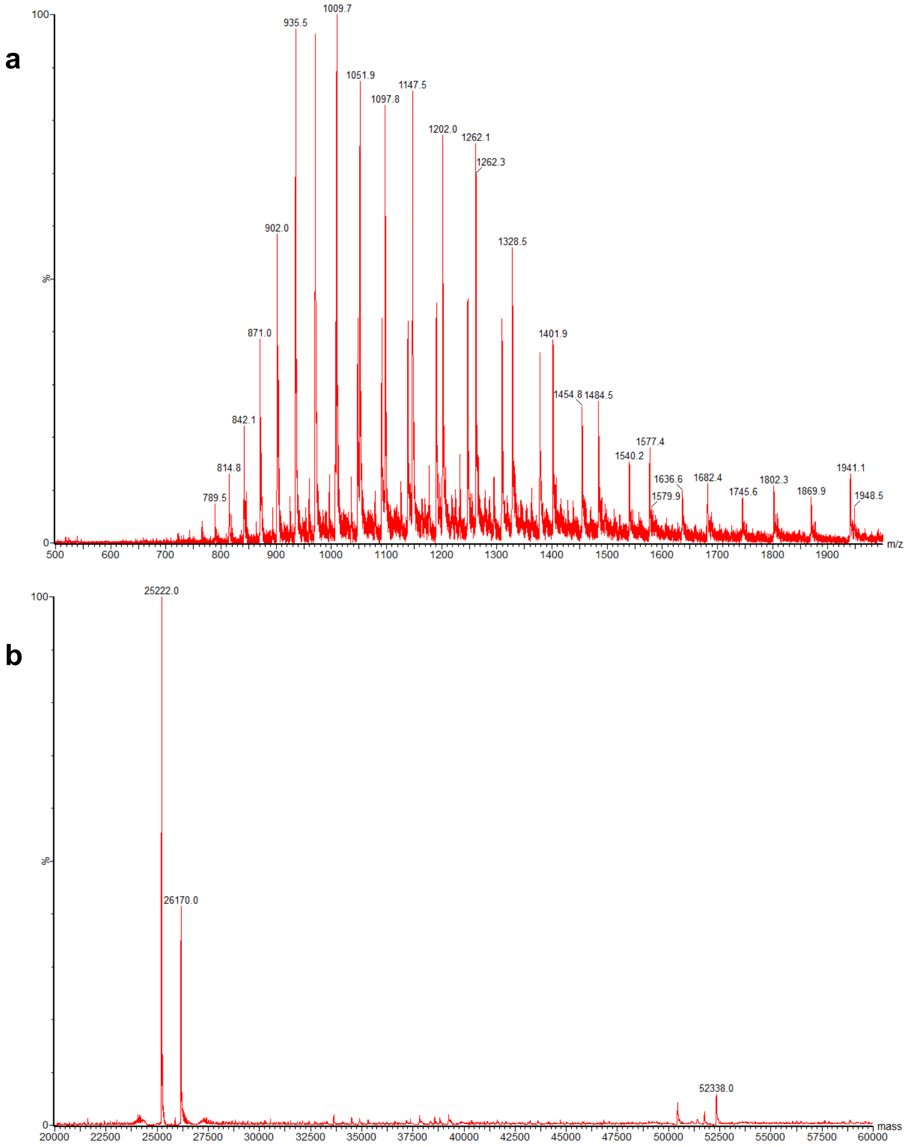

Figure S21. LC–MS spectra of reduced Fc 274C; **a**) ion series and **b**) deconvoluted spectrum.

Fc 289C

Sequence:
THTCPPCPAPEFEGGPSVFLFPPKPKDTLMISRTPEVTCVVVDVSHEDPEVKF
NWYVDGVEVHNAKCKPREEQYNSTYRVVSVLTVLHQDWLNGKEYKCKVSNK
ALPASIEKTISKAKGQPREPQVYTLPPSREEMTKNQVSLTCLVKGFYPSDIAVE
WESNGQPENNYKTTPPVLDSDGSFFLYSKLTVDKSRWQQGNVFSCSVMHEA
LHNHYTQKSLSLSPG

Calculated Isotopically Averaged Molecular Weight: 25249 Da

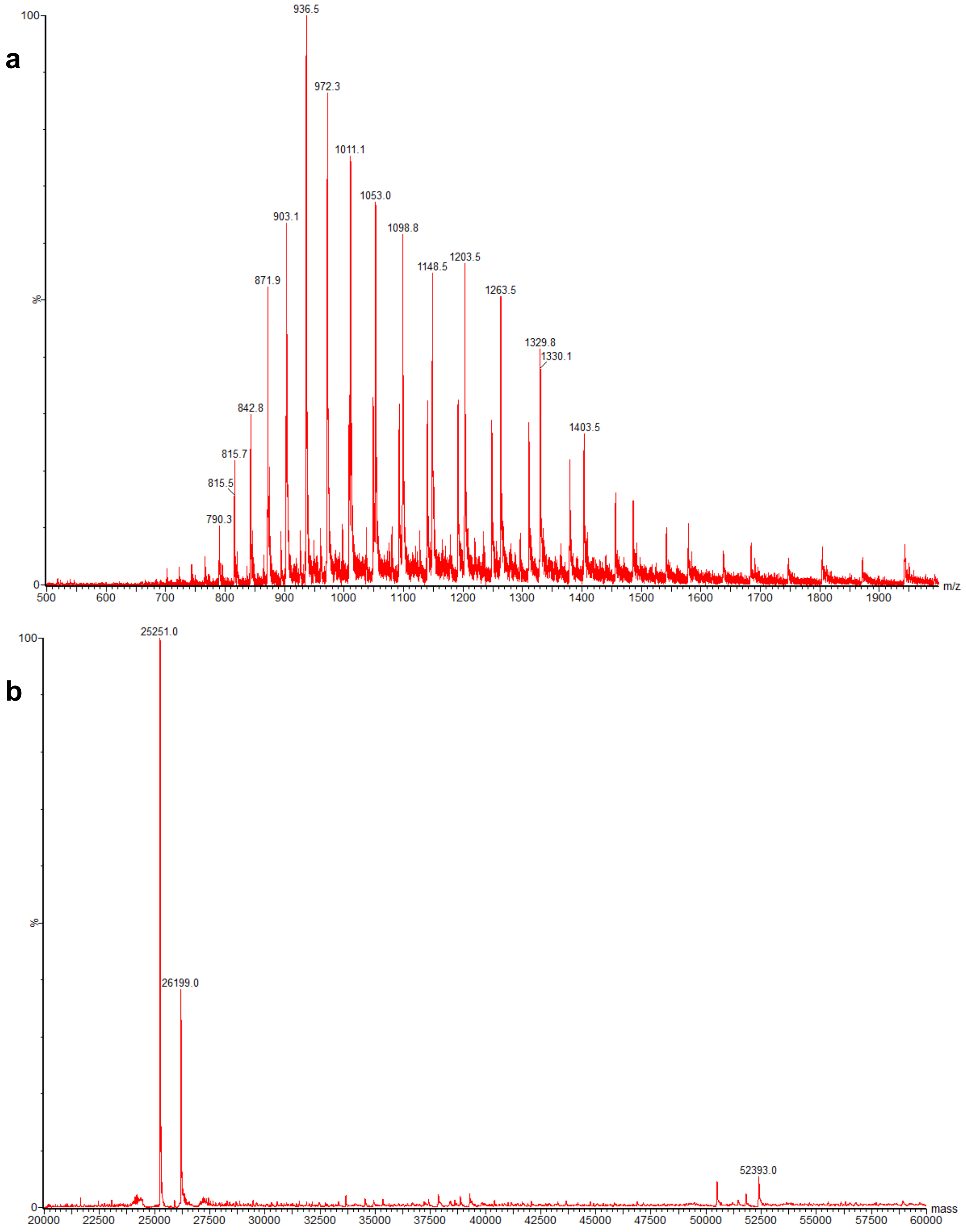

Figure S22. LC–MS spectra of reduced Fc 289C; **a**) ion series and **b**) deconvoluted spectrum.

Fc 442C

Sequence:
THTCPPCPAPEFEGGPSVFLFPPKPKDTLMISRTPEVTCVVVDVSHEDPEVKF
NWYVDGVEVHNAKTKPREEQYNSTYRVVSVLTVLHQDWLNGKEYKCKVSNK
ALPASIEKTISKAKGQPREPQVYTLPPSREEMTKNQVSLTCLVKGFYPSDIAVE
WESNGQPENNYKTTPPVLDSDGSFFLYSKLTVDKSRWQQGNVFSCSVMHEA
LHNHYTQKSLCLSPG

Calculated Isotopically Averaged Molecular Weight: 25263 Da

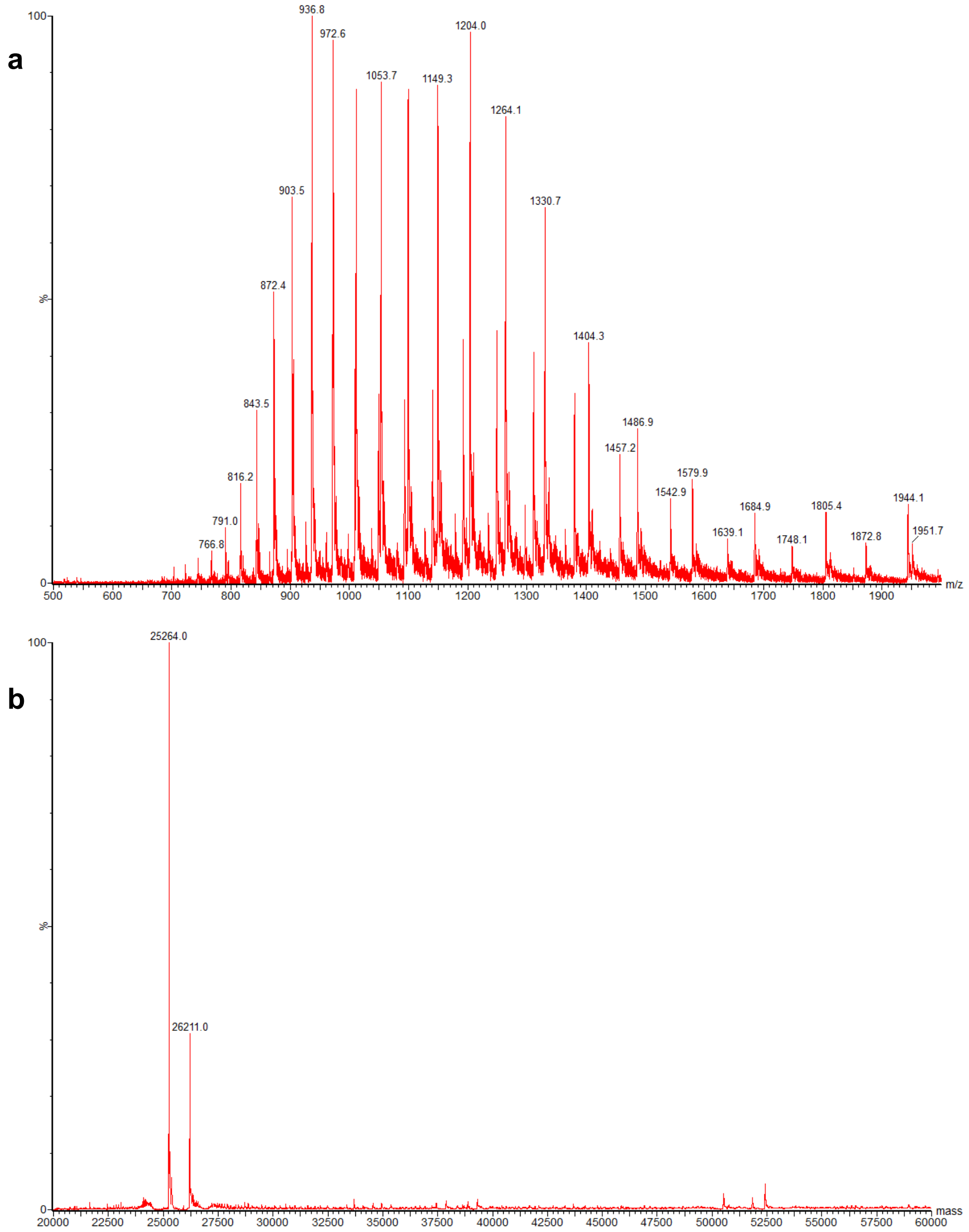

Figure S23. LC–MS spectra of reduced Fc 442C; **a**) ion series and **b**) deconvoluted spectrum.

Ub-K63C Conjugation Reactions with 1–10

Conjugation reactions were performed at 20 μM. 4.4 μL of a stock solution of Ub-K63C (90 μM) was added to an eppendorf containing 15.6 μL of NaP_i buffer (pH 8.0, 50 mM). The resulting mixture was vortexed, and afterwards a 1 mM solution of **1–10** (1.2 μL, 3 equiv) in DMF was added. The reaction mixture was then shaken for 30 min at 25 ºC. After this time, a 10 μL aliquot of the reaction mixture was analysed by LC–MS.

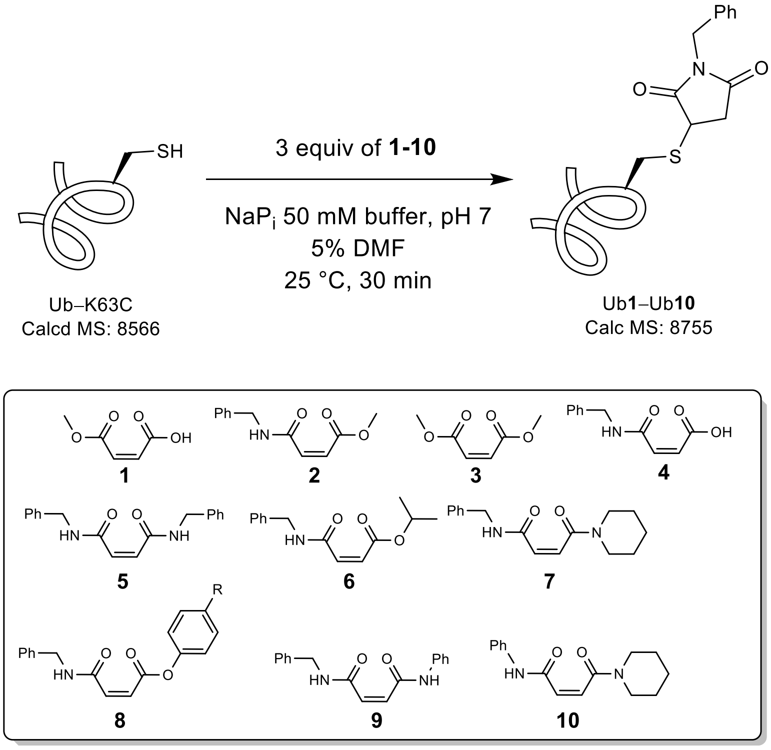

Figure S24. Ub-K63C conjugation with compounds **1–10**.

Compound	Conversion to Ub 1–Ub 10 (%)
**1**	0
**2**	99
**3**	0
**4**	0
**5**	40
**6**	99
**7**	99
**8**	99
**9**	99
**10**	99

Table S2: Table showing the conjugation conversion of **1–10** with Ub under the described conditions. No conjugation was observed with compounds **1**, **3** and **4** under the conditions tried, only native protein and traces of dimer were observed. For the other compounds, complete conjugation to a single species could be observed, except for **5** where a mixture was observed.

Conjugation Reaction Between Ub-K63C and **2**

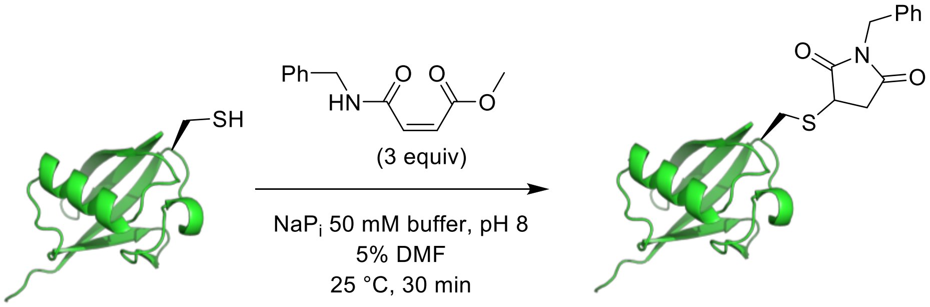

Ub K63C
Calcd Mass: 8567 Da

Ub–**2**
Calcd Mass: 8755 Da

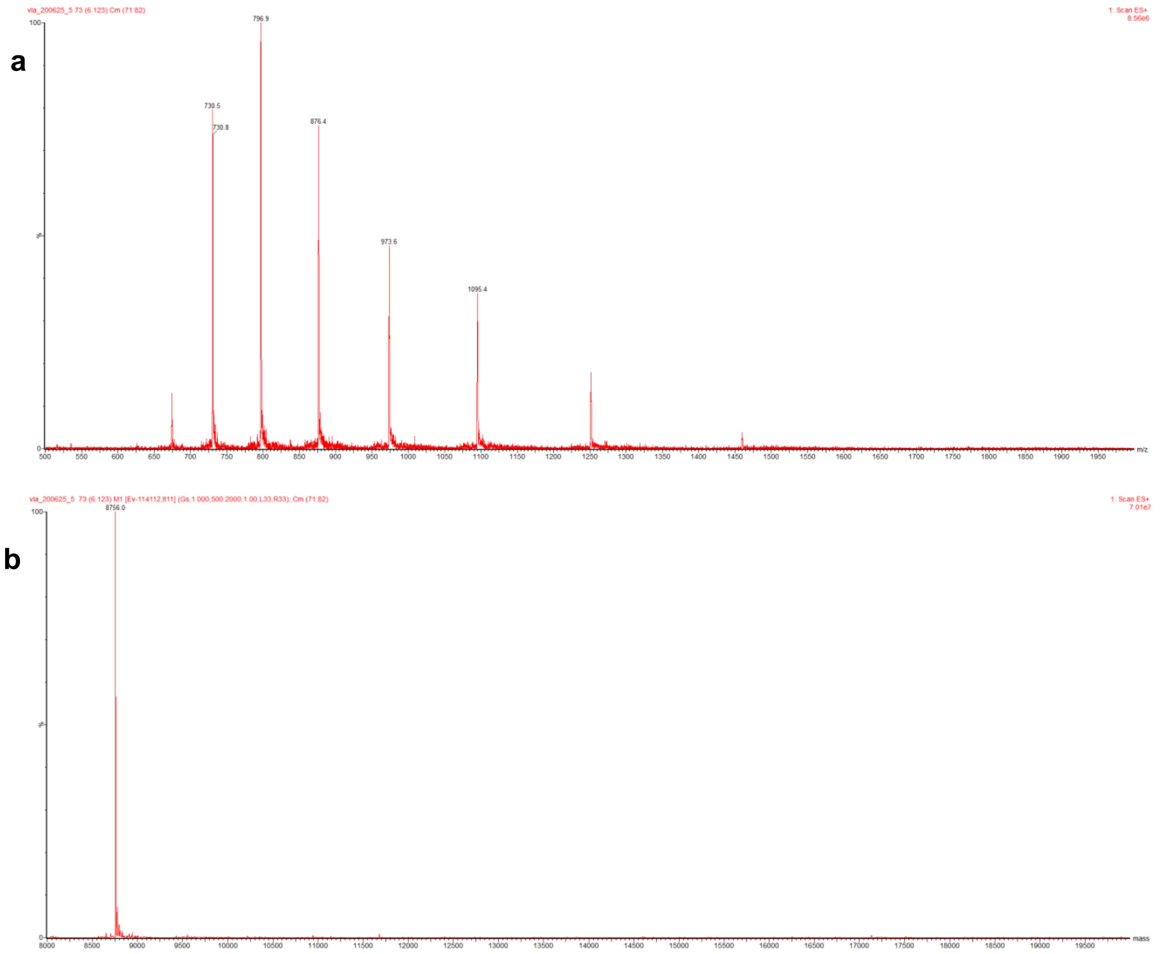

Figure S25. LC–MS spectra of Ub-**2**; **a**) ion series and **b**) deconvoluted spectrum.

Conjugation Reaction Between Ub-K63C and **5**

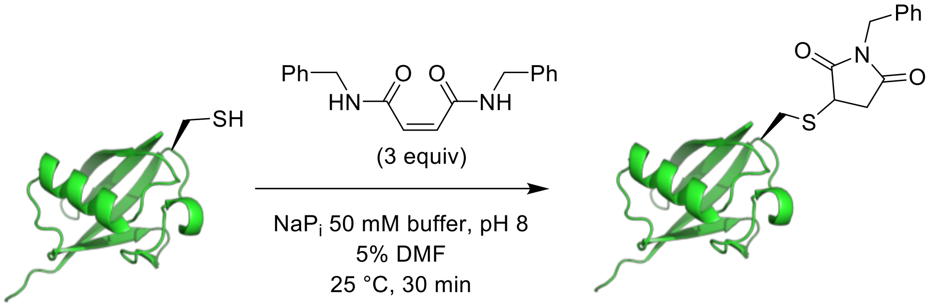

Ub K63C
Calcd Mass: 8567 Da

Ub–**5**
Calcd Mass: 8755 Da

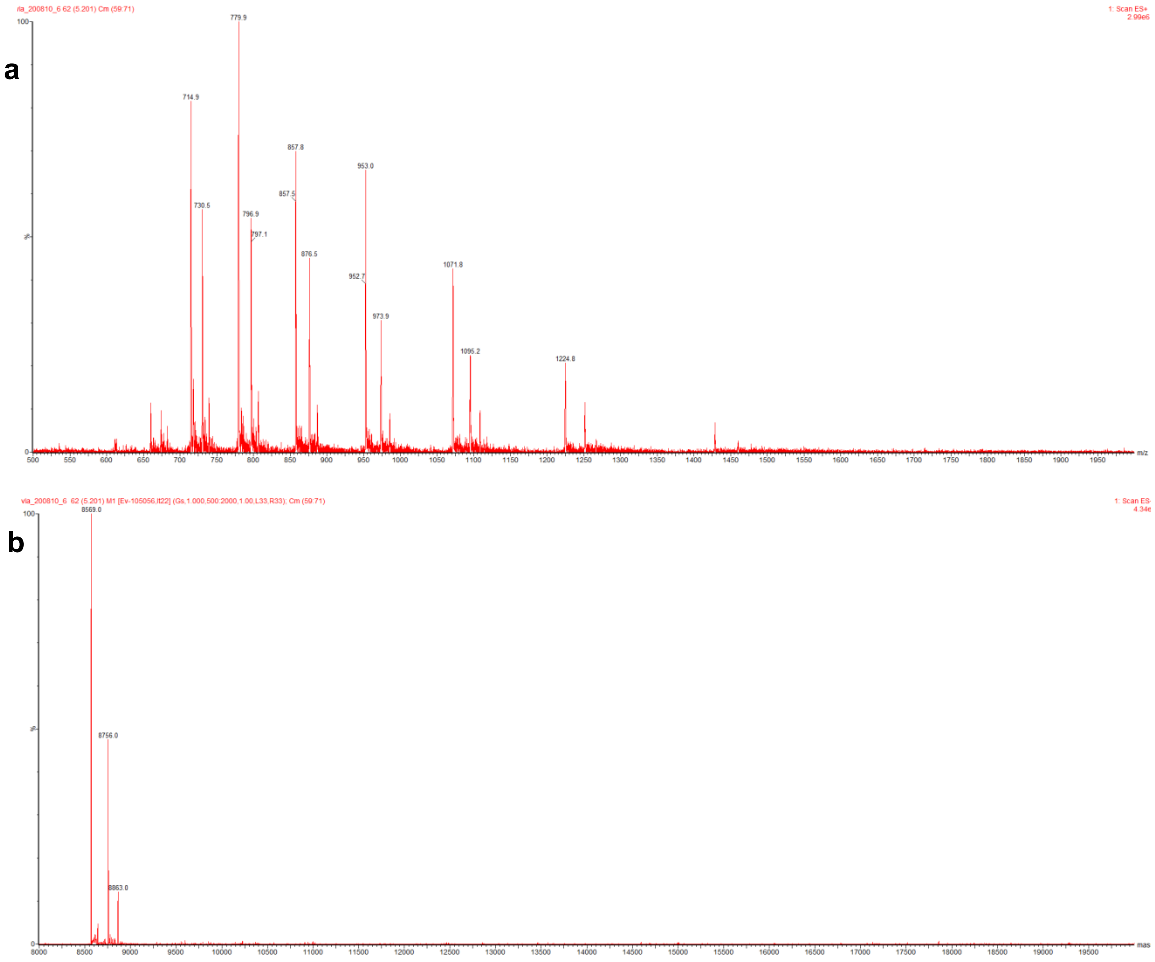

Figure S26. LC–MS spectra of Ub-**5**; **a**) ion series and **b**) deconvoluted spectrum.

Only partial conversion can be observed for this reaction. Also traces of m/z: 8863 Da can be observed which corresponds to the complete addition of the molecule before cyclization.

Conjugation Reaction Between Ub-K63C and **6**

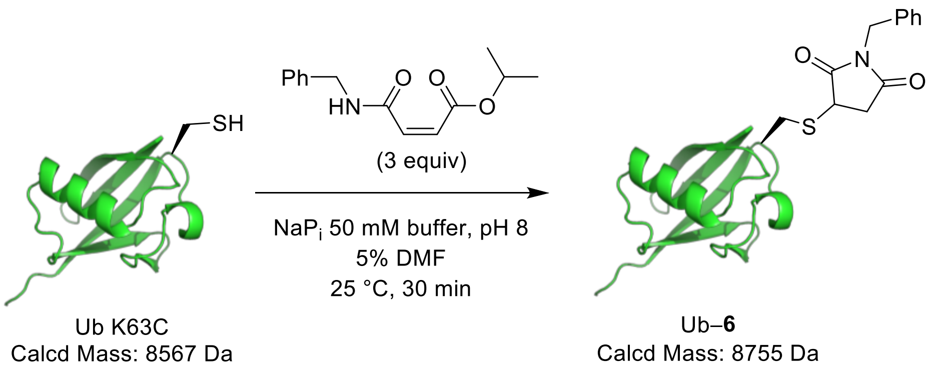

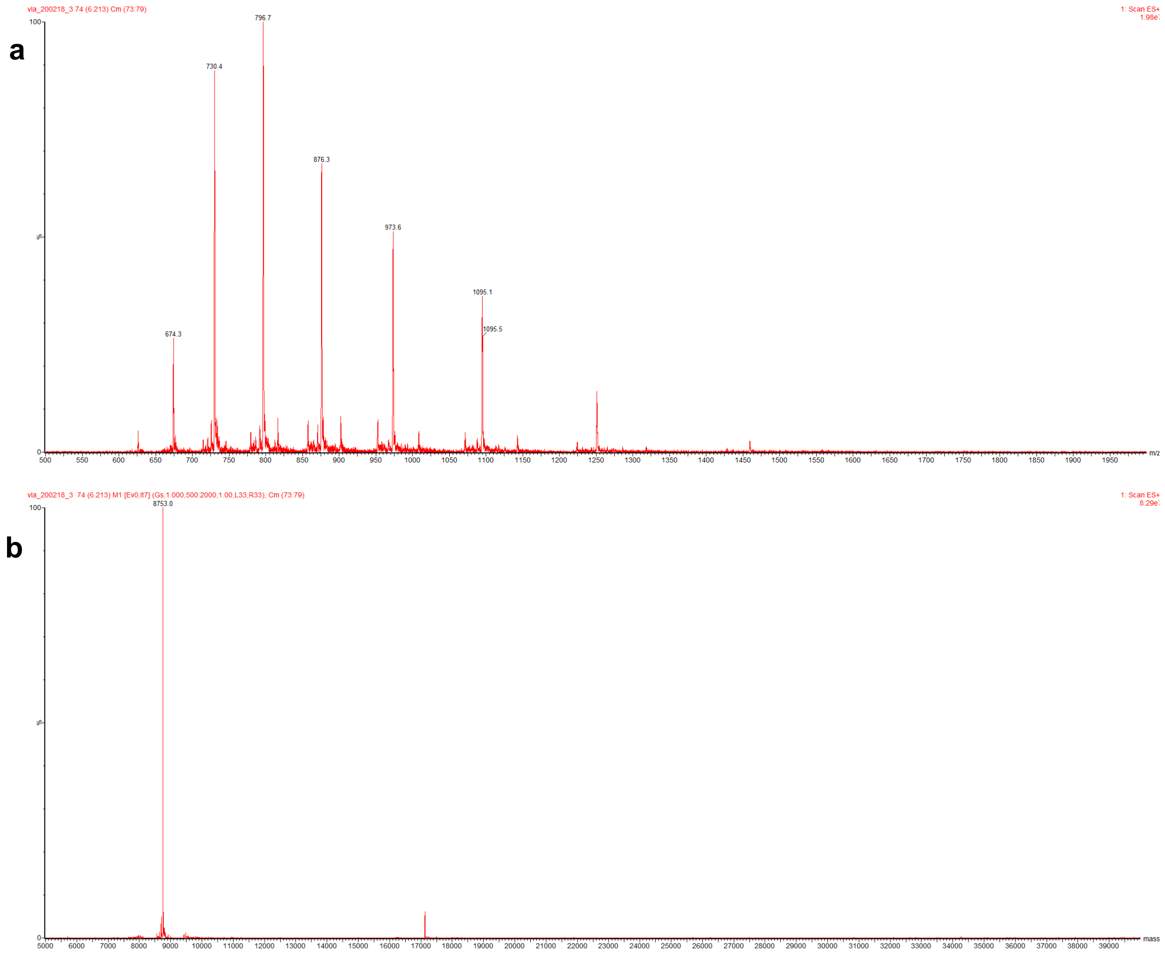

Figure S27. LC–MS spectra of Ub-**6**; **a**) ion series and **b**) deconvoluted spectrum.

Conjugation Reaction Between Ub-K63C and **7**

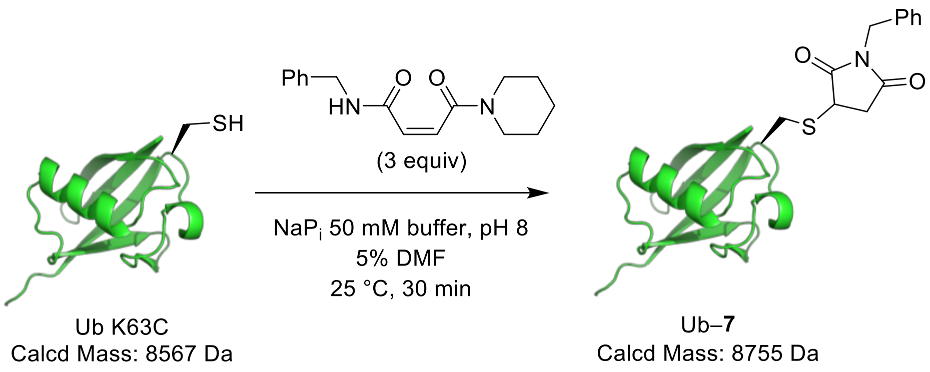

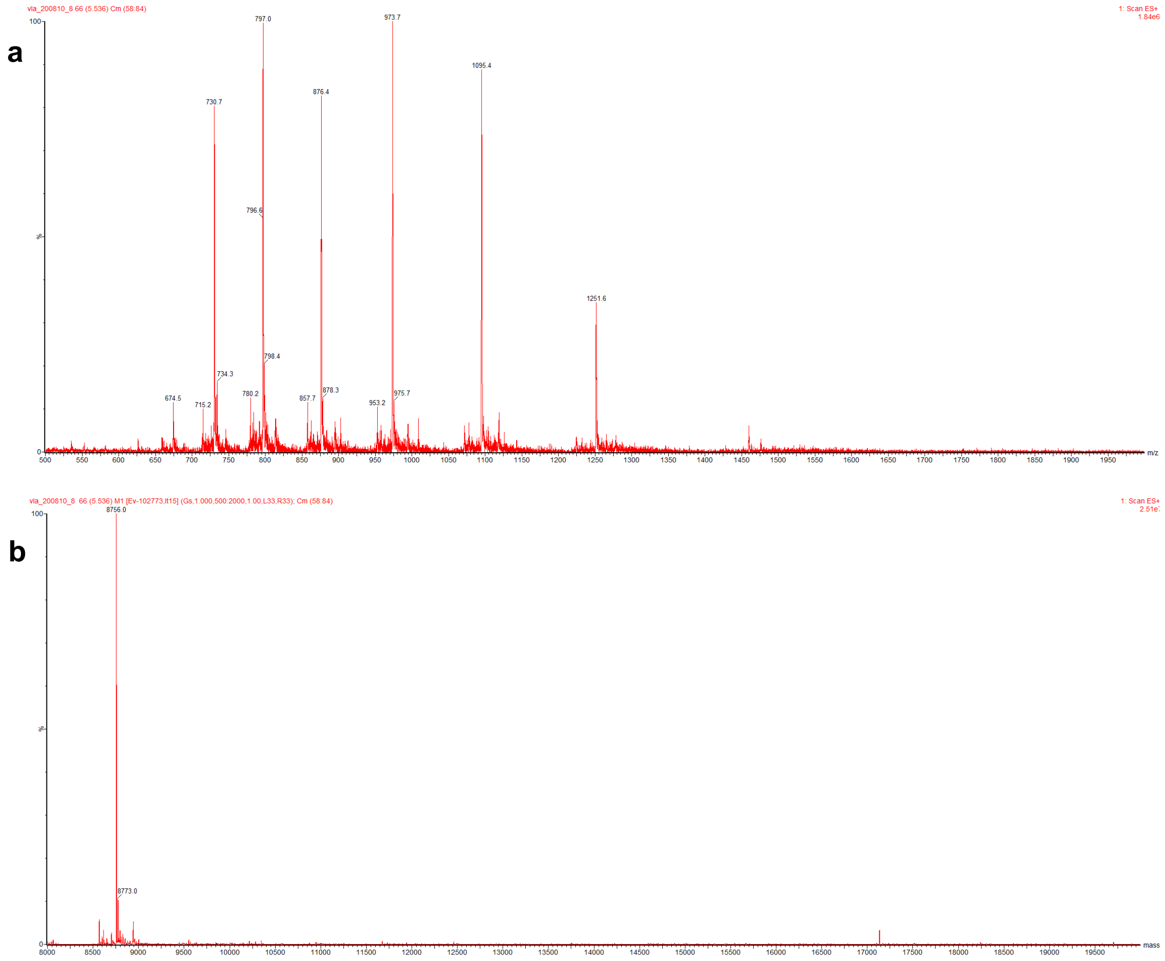

Figure S28. LC–MS spectra of Ub-**7**; **a**) ion series and **b**) deconvoluted spectrum.

Conjugation Reaction Between Ub-K63C and **8**

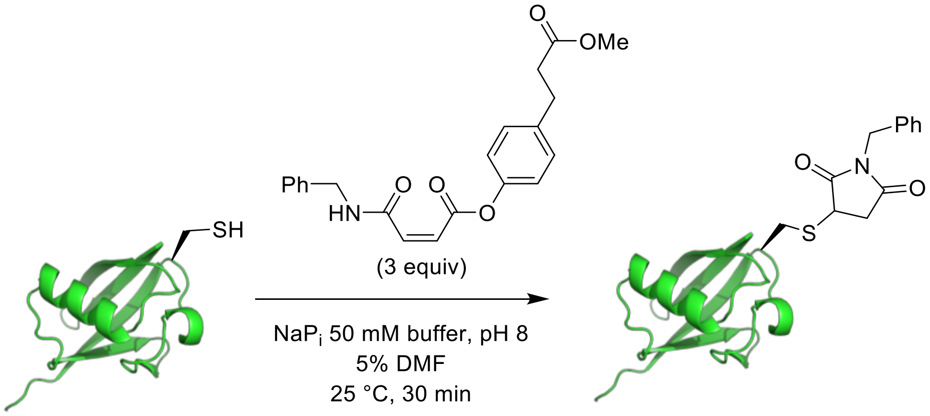

Ub K63C
Calcd Mass: 8567 Da

Ub–**8**
Calcd Mass: 8755 Da

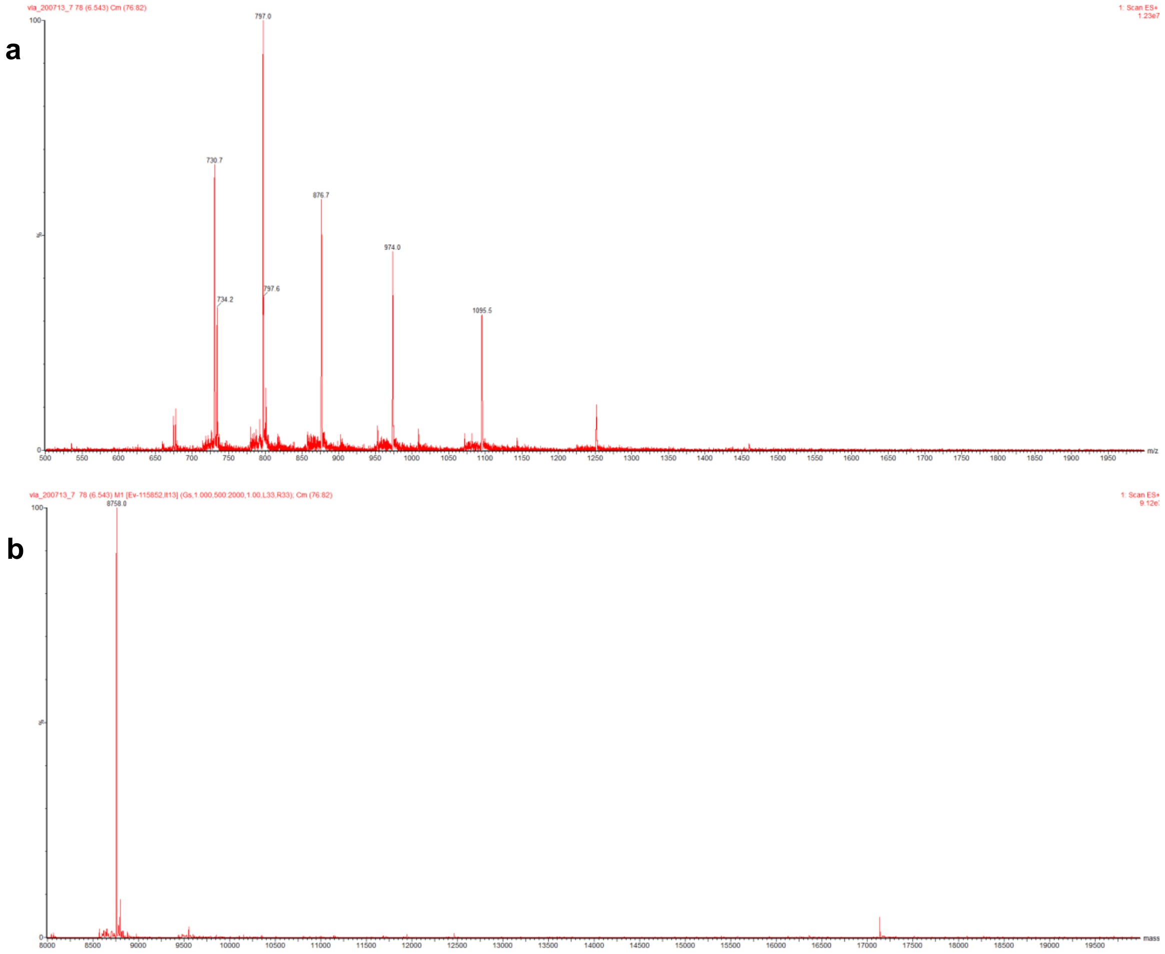

Figure S29. LC–MS spectra of Ub-**8**; **a**) ion series and **b**) deconvoluted spectrum.

Conjugation Reaction Between Ub-K63C and **9**

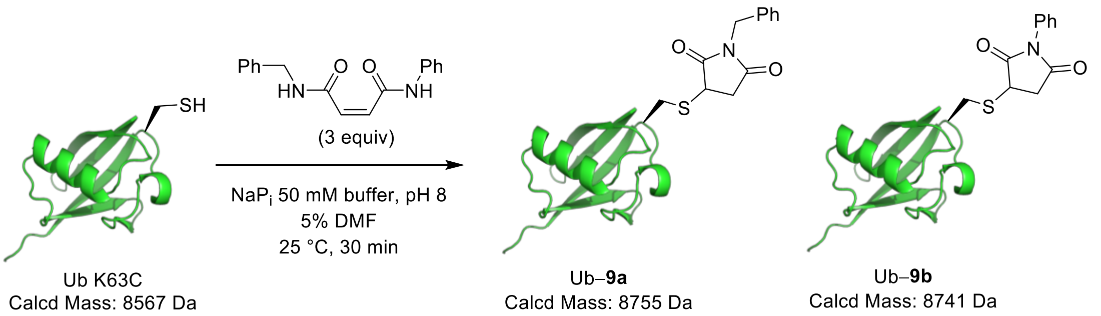

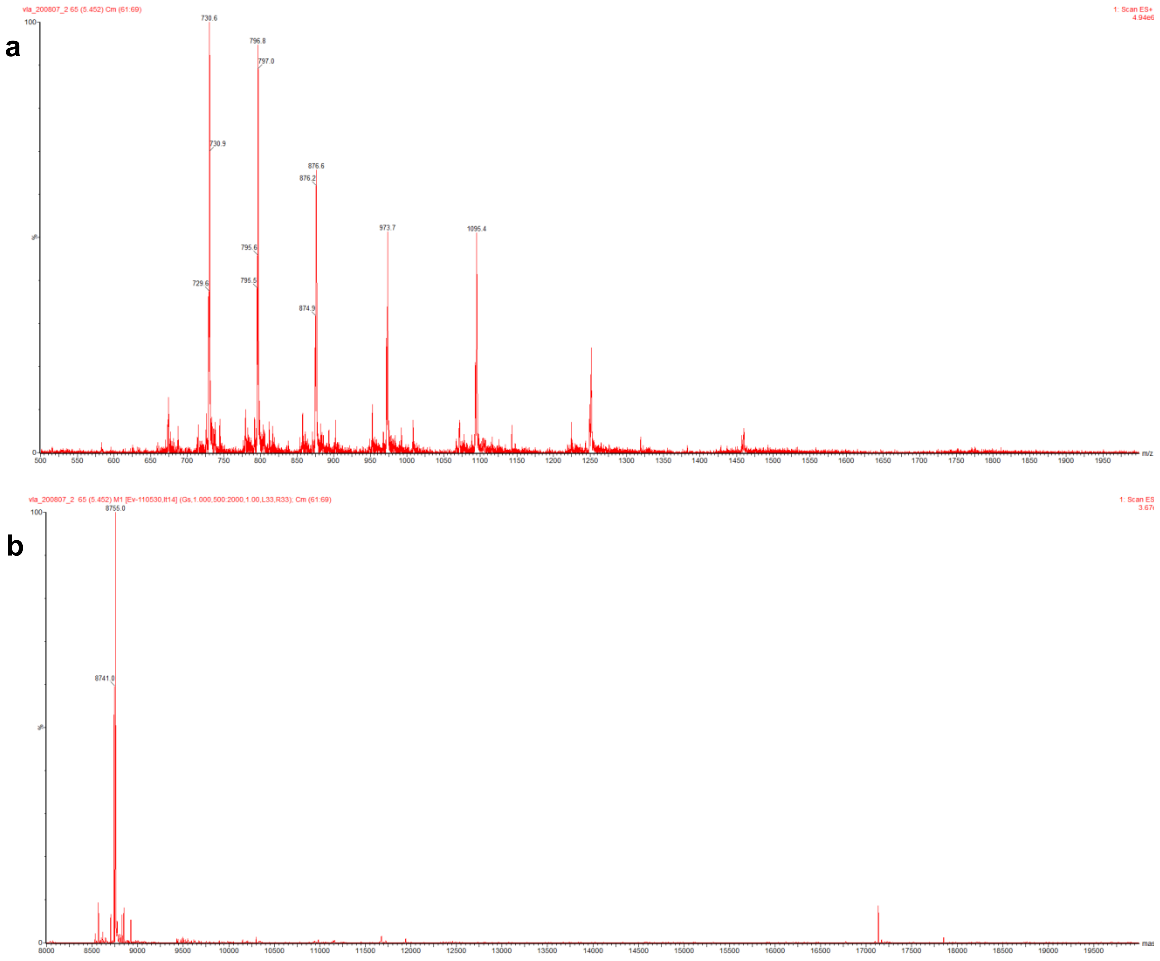

Figure S30. LC–MS spectra of Ub-**9**; **a**) ion series and **b**) deconvoluted spectrum.

Conjugation Reaction Between Ub-K63C and **10**

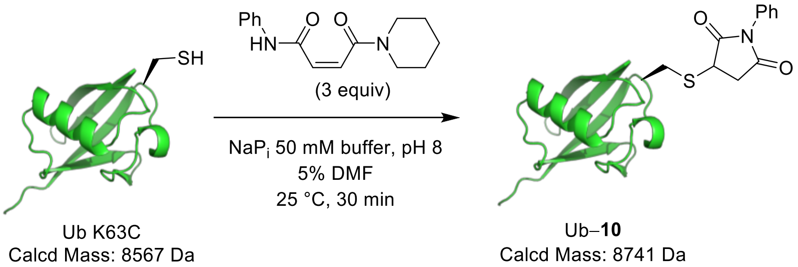

a

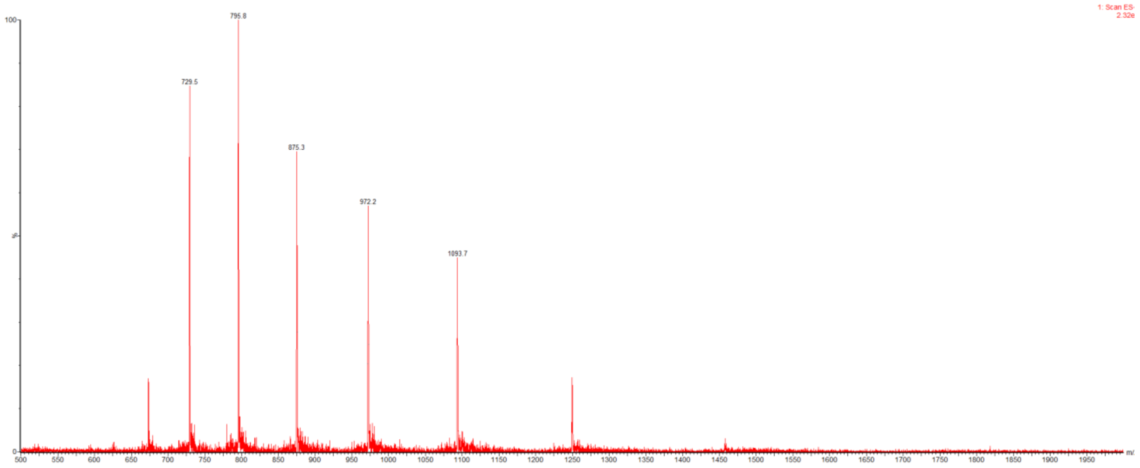

b

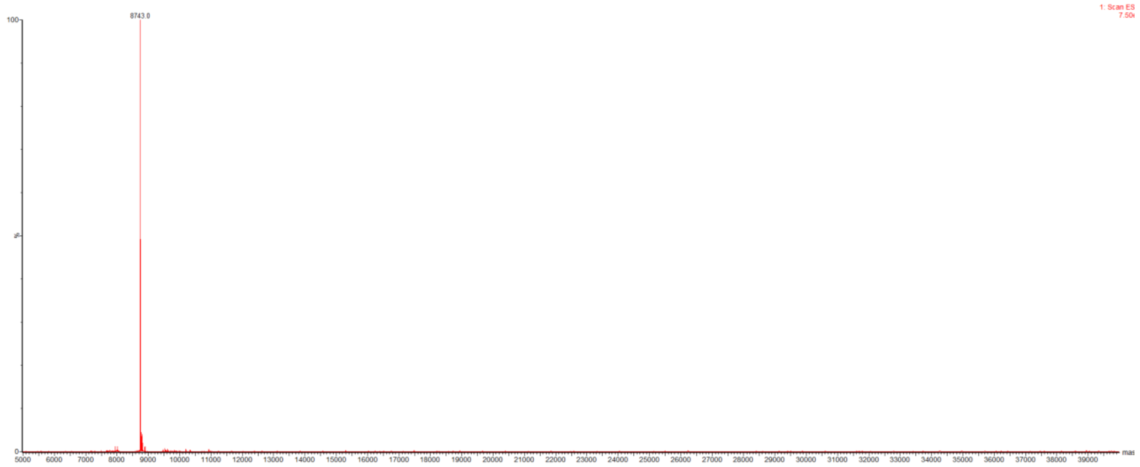

Figure S31. LC–MS spectra of Ub-**10**; **a**) ion series and **b**) deconvoluted spectrum.

Ub-7, Ellman's chemical control

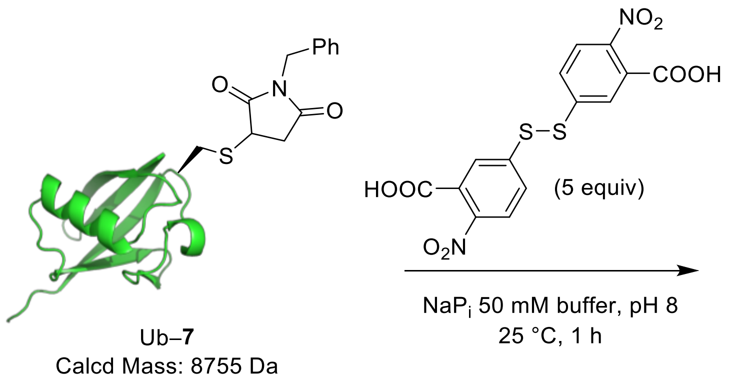

A 20 μL aliquot of the Ub-**7** (10 μM) in NaP$_i$ buffer (20 mM, pH 8.0) was prepared. 1 μL of a 1 mM of Ellman's reagent in DMF was added at room temperature and the resulting mixture vortexed for 10 seconds. The resulting reaction mixture was then shaken at 25 ºC. After 1 h, a 10 μL aliquot of each reaction mixture was analysed by LC–MS.

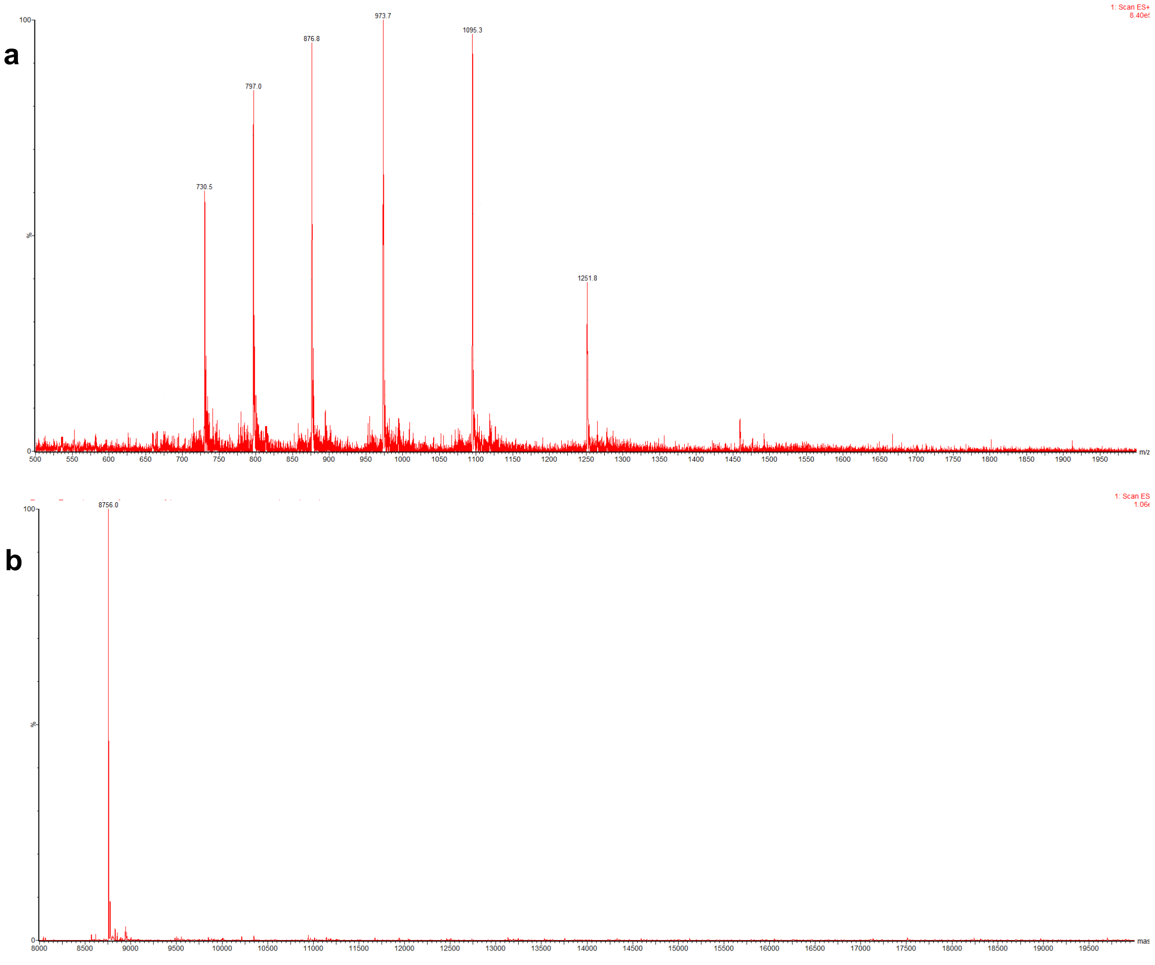

Figure S32. LC–MS spectra of Ub-**7** after treatment with Ellman's reagent. No addition observed; **a**) ion series and **b**) deconvoluted spectrum.

Ub Conjugation with 10 at Different pHs

Conjugation Reaction Between Ub-K63C and **10** at pH 5

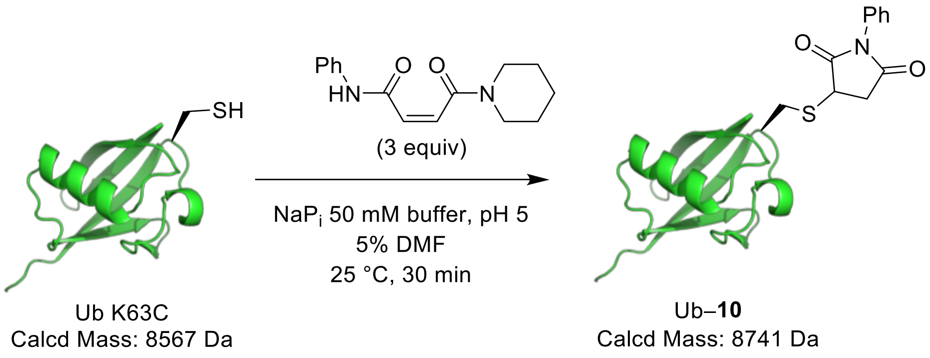

4.4 µL of a stock solution of Ub-K63C (90 µM) was added to an eppendorf containing 15.6 µL of sodium acetate buffer (pH 5, 50 mM). The resulting mixture was vortexed, and afterwards 1 mM solution of **10** (1.2 µL, 3 equiv) in DMF was added. The reaction mixture was then shaken for 30 min at 25 ºC. After this time, a 10 µL aliquot of the reaction mixture was analysed by LC–MS. At higher pH, the hydrolysed maleimide is the main species observed.

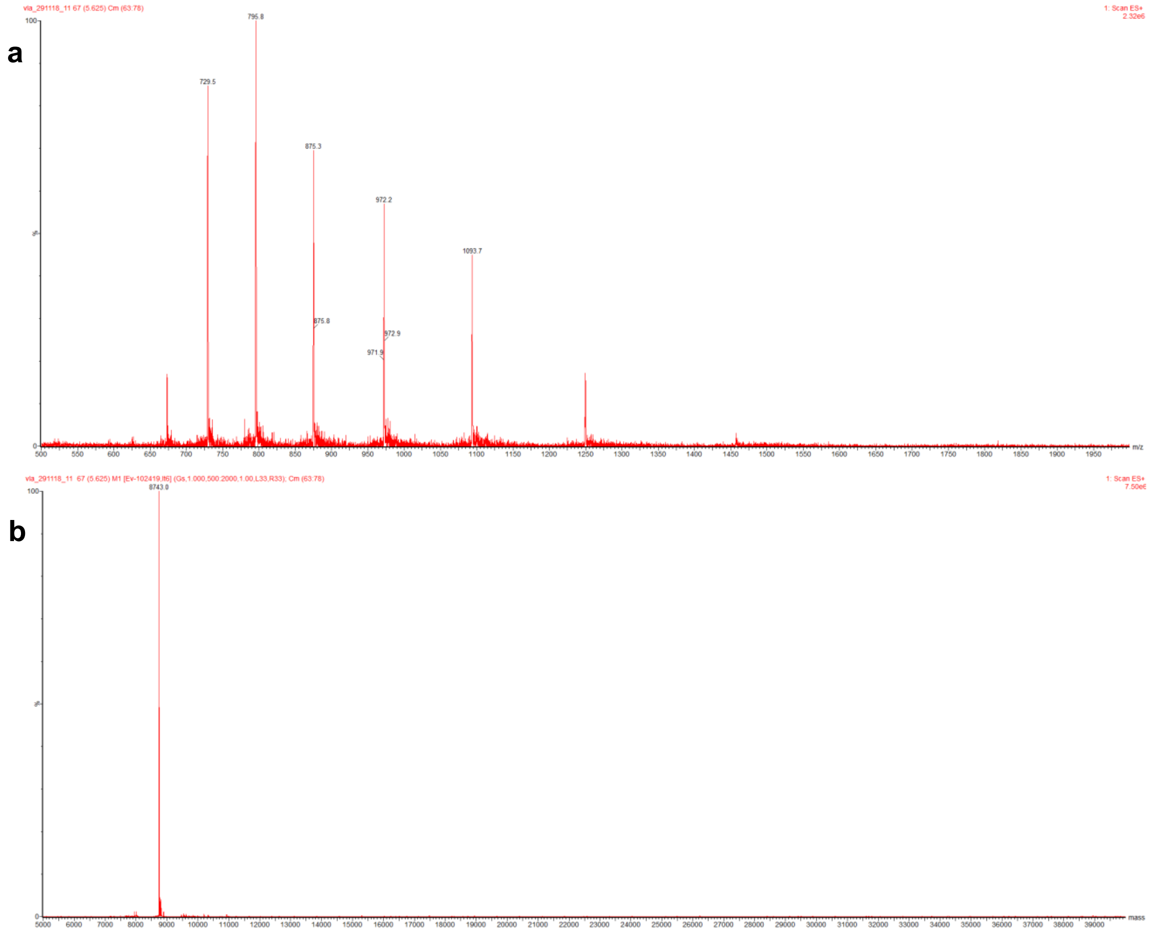

Figure S33. LC–MS spectra of the reaction between Ub-K63C and **10** at pH 5; **a**) ion series and **b**) deconvoluted spectrum.

Conjugation Reaction Between Ub-K63C and **10** at pH 6

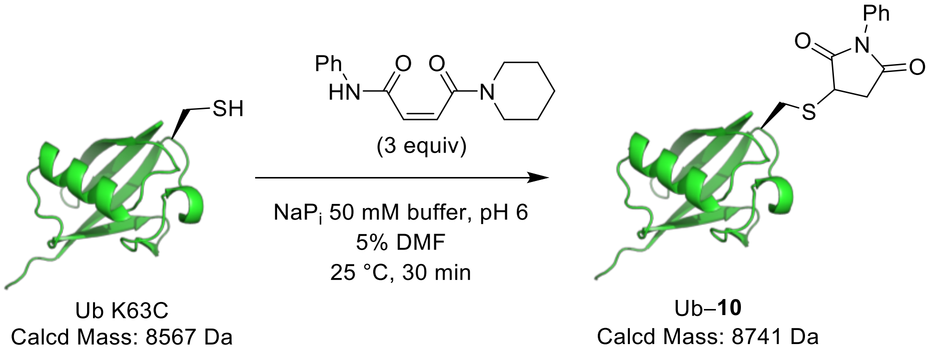

4.4 μL of a stock solution of Ub-K63C (90 μM) was added to an eppendorf containing 15.6 μL of sodium acetate buffer (pH 6, 50 mM). The resulting mixture was vortexed, and afterwards 1 mM solution of **10** (1.2 μL, 3 equiv) in DMF was added. The reaction mixture was then shaken for 30 min at 25 ºC. After this time, a 10 μL aliquot of the reaction mixture was analysed by LC–MS.

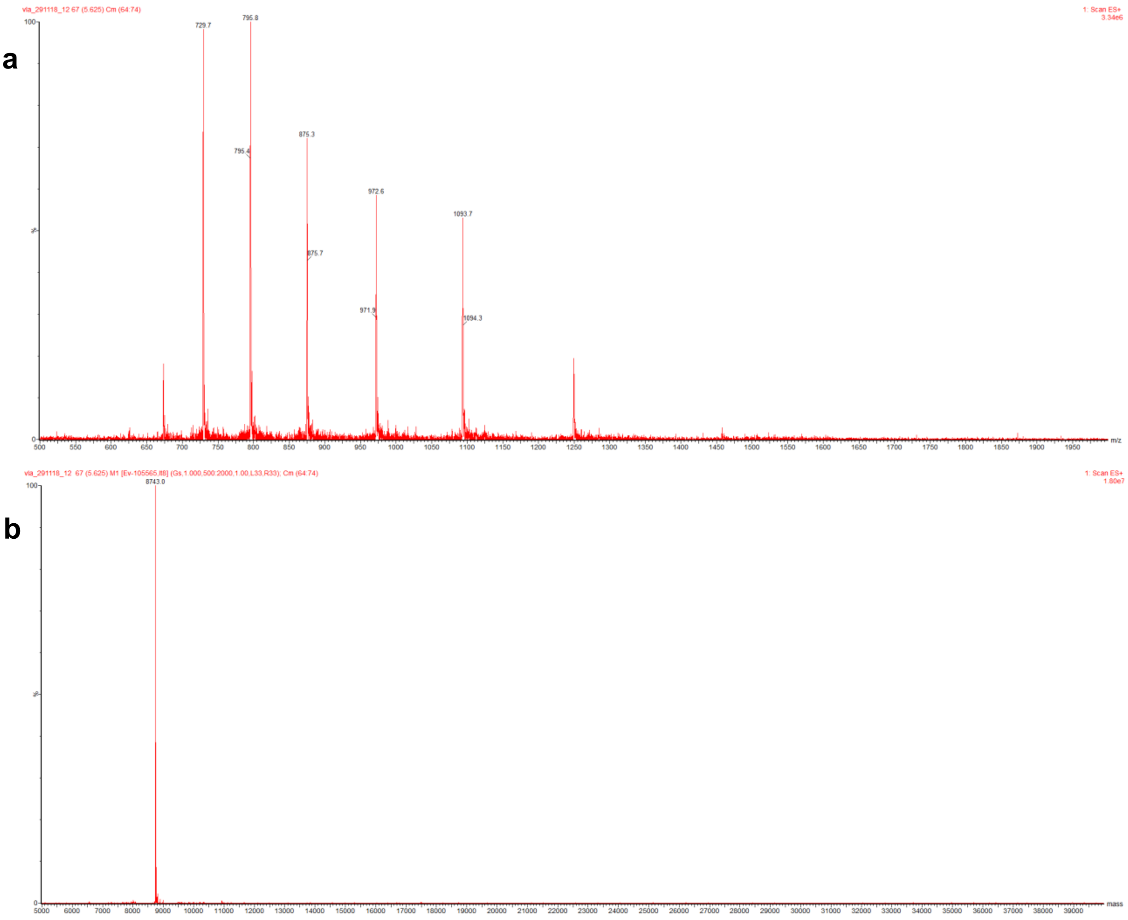

Figure S34. LC–MS spectra of the reaction between Ub-K63C and **10** at pH 6; **a**) ion series and **b**) deconvoluted spectrum.

Conjugation Reaction Between Ub-K63C and **10** at pH 7

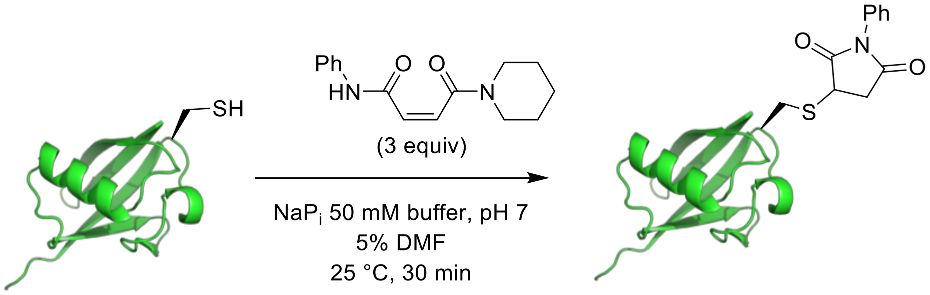

Ub K63C
Calcd Mass: 8567 Da

Ub–**10**
Calcd Mass: 8741 Da

4.4 µL of a stock solution of Ub-K63C (90 µM) was added to an eppendorf containing 15.6 µL of NaP$_i$ buffer (pH 7.0, 50 mM). The resulting mixture was vortexed, and afterwards 1 mM solution of **10** (1.2 µL, 3 equiv) in DMF was added. The reaction mixture was then shaken for 30 min at 25 ºC. After this time, a 10 µL aliquot of the reaction mixture was analysed by LC–MS.

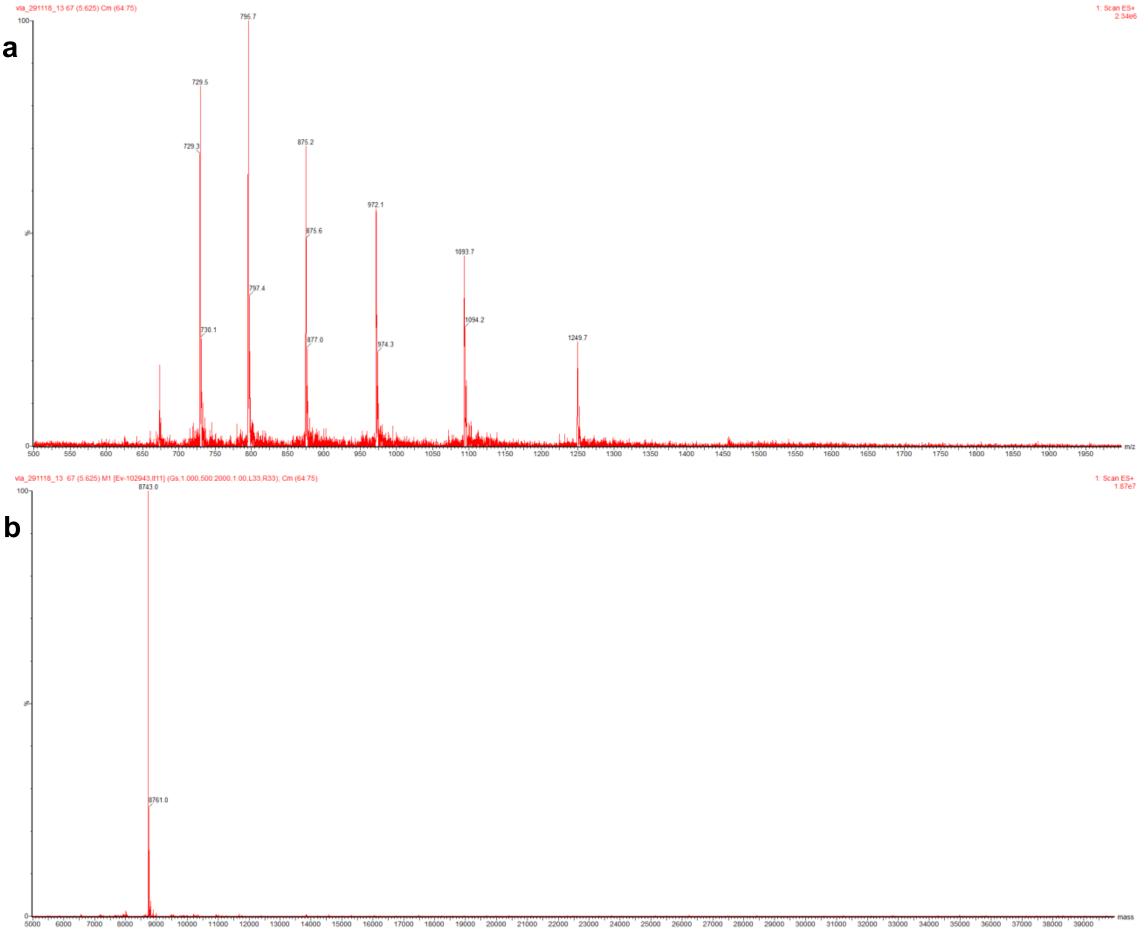

Figure S35. LC–MS spectra of the reaction between Ub-K63C and **10** at pH 7; **a**) ion series and **b**) deconvoluted spectrum.

Conjugation Reaction Between Ub-K63C and **10** at pH 8 and 37 ºC

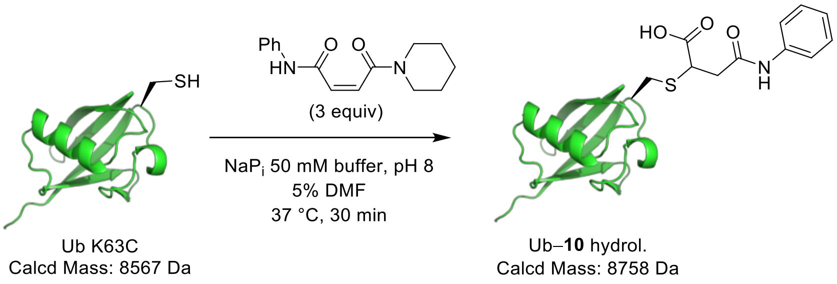

4.4 µL of a stock solution of Ub-K63C (90 µM) was added to an eppendorf containing 15.6 µL of NaP$_i$ buffer (pH 8.0, 50 mM). The resulting mixture was vortexed, and afterwards 1 mM solution of **10** (1.2 µL, 3 equiv) in DMF was added. The reaction mixture was then shaken for 30 min at 37 ºC. After this time, a 10 µL aliquot of the reaction mixture was analysed by LC–MS.

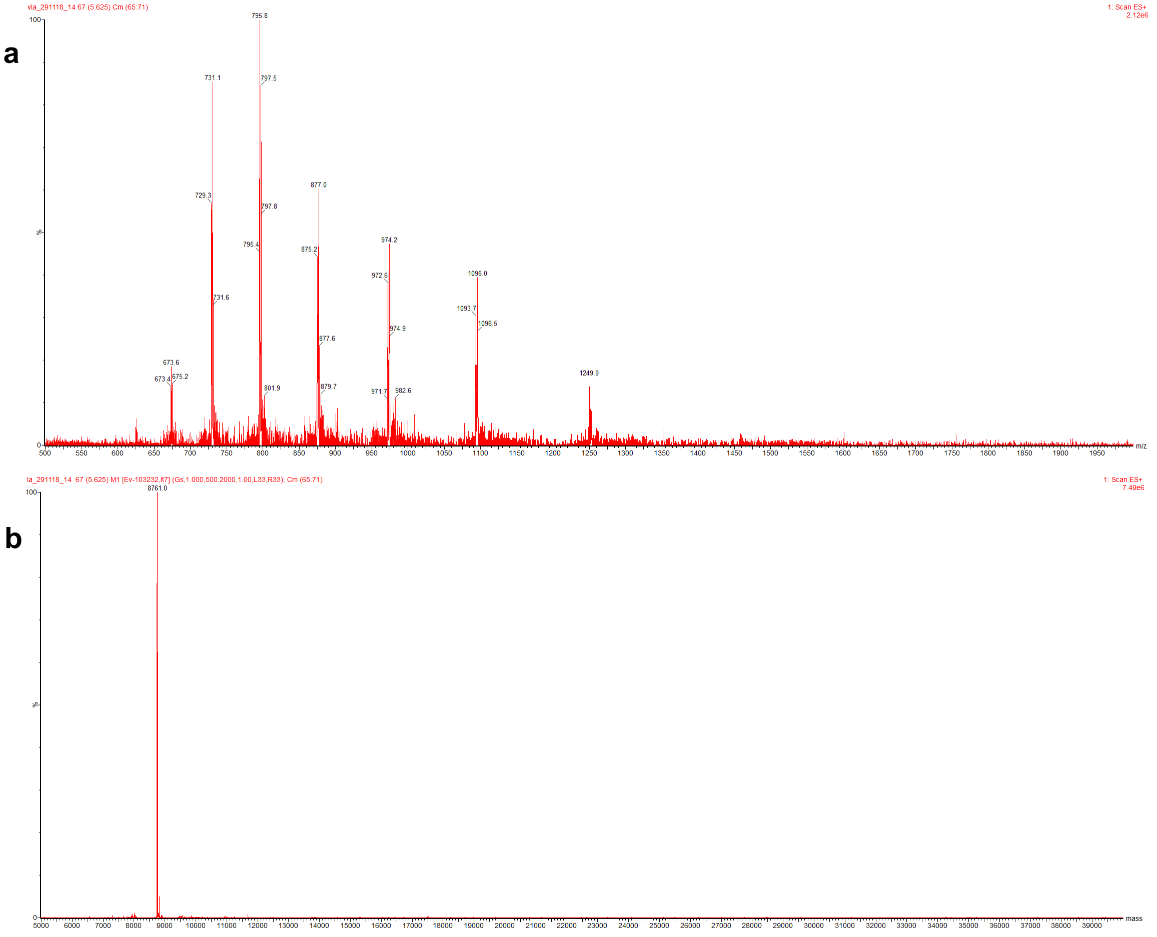

Figure S36. LC–MS spectra of the reaction between Ub-K63C and **10** at pH 8 and 37 ºC; **a**) ion series and **b**) deconvoluted spectrum.

The hydrolyzed succinimide linkage is observed.

Conjugation Reaction Between Ub-K63C and **10** at pH 9

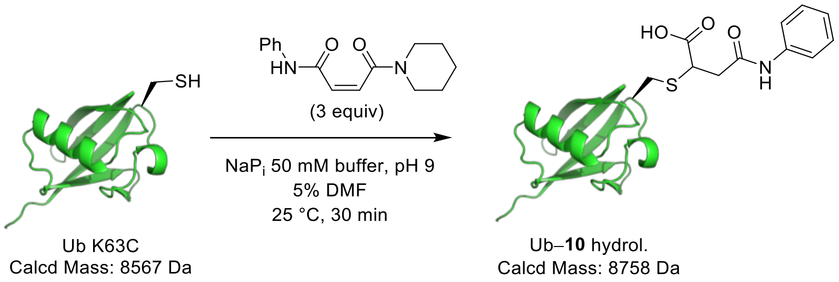

4.4 µL of a stock solution of Ub-K63C (90 µM) was added to an eppendorf containing 15.6 µL of Tris buffer (pH 9.0, 50 mM). The resulting mixture was vortexed, and afterwards 1 mM solution of **10** (1.2 µL, 3 equiv) in DMF was added. The reaction mixture was then shaken for 30 min at 25 ºC. After this time, a 10 µL aliquot of the reaction mixture was analysed by LC–MS.

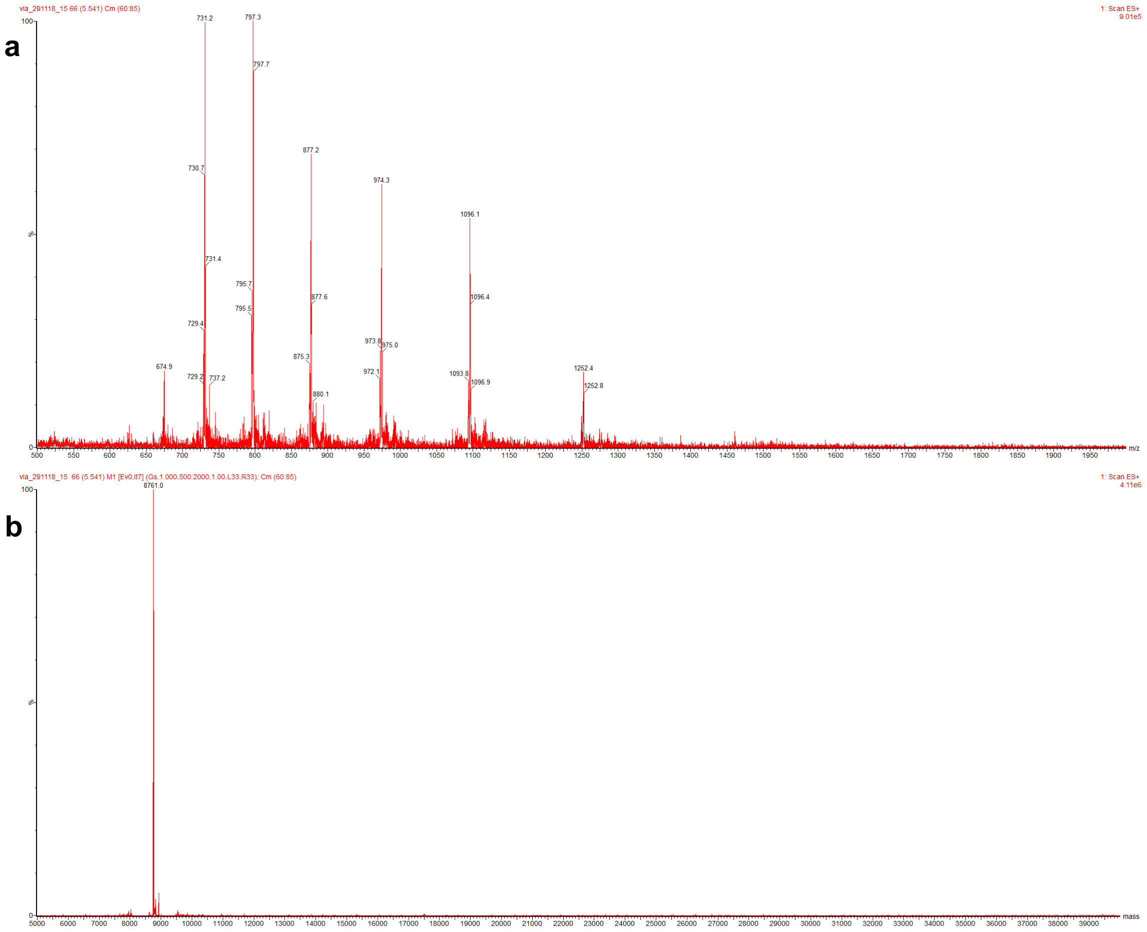

Figure S37. LC–MS spectra of the reaction between Ub-K63C and **10** at pH 9; **a**) ion series and **b**) deconvoluted spectrum.

Regioselectivity of Compound 9 at different pHs

Conjugation Reaction Between Ub-K63C and **9** at pH 7

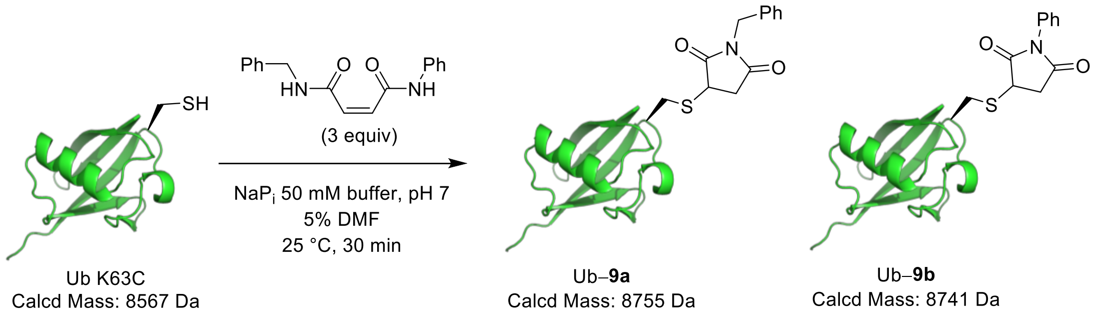

4.4 µL of a stock solution of Ub-K63C (90 µM) was added to an eppendorf containing 15.6 µL of NaP$_i$ buffer (pH 7.0, 50 mM). The resulting mixture was vortexed, and afterwards 1 mM solution of **9** (1.2 µL, 3 equiv) in DMF was added. The reaction mixture was then shaken for 30 min at 25 ºC. After this time, a 10 µL aliquot of the reaction mixture was analysed by LC–MS.

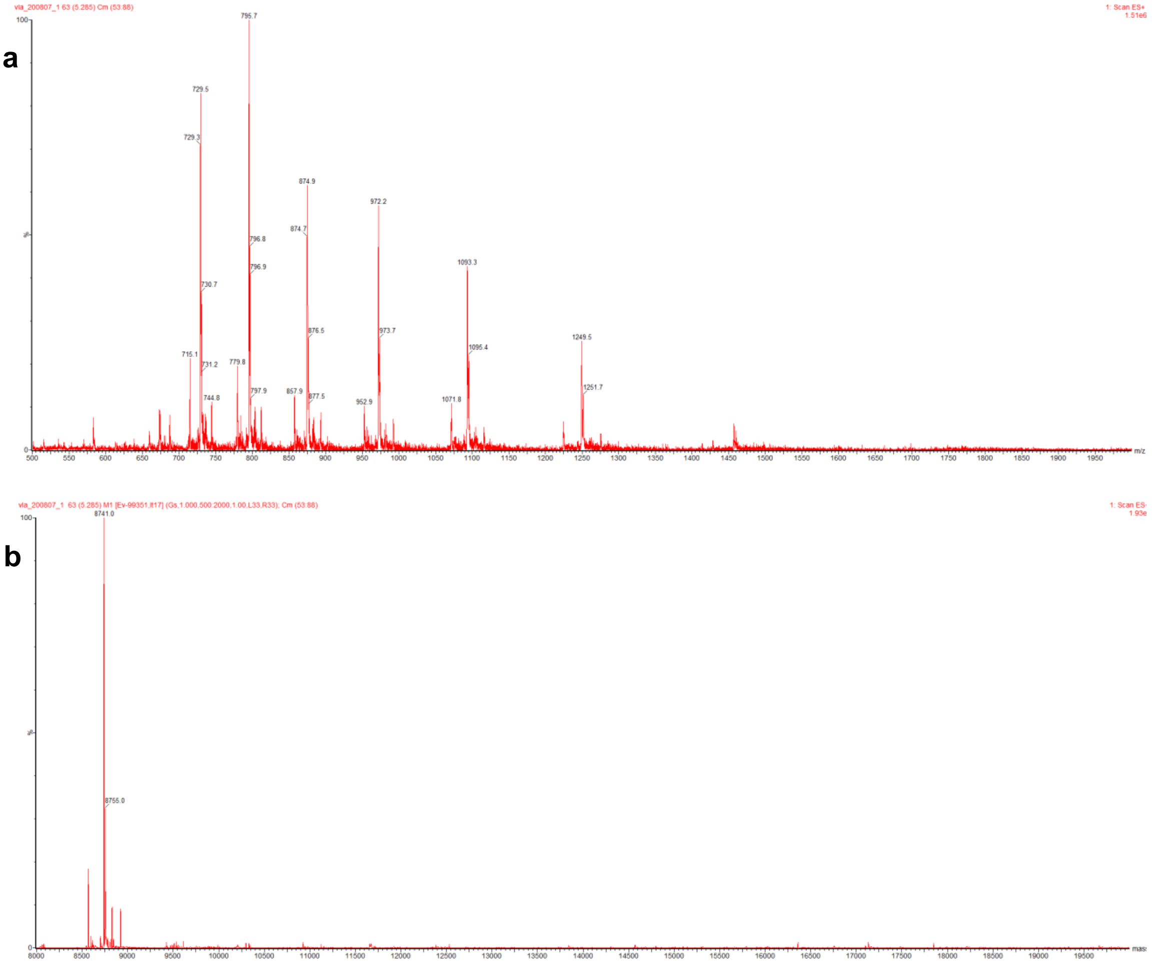

Figure S38. LC–MS spectra of the reaction between Ub-K63C and **9** at pH 7; **a**) ion series and **b**) deconvoluted spectrum.

Conjugation Reaction Between Ub-K63C and **9** at pH 9

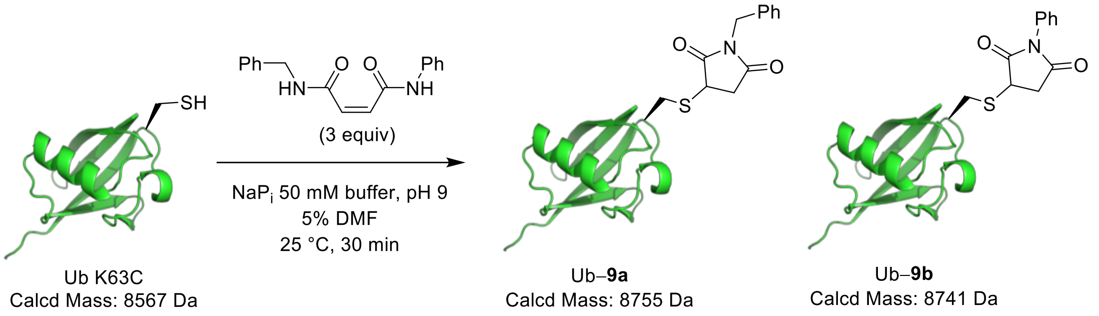

4.4 μL of a stock solution of Ub-K63C (90 μM) was added to an eppendorf containing 15.6 μL of NaP$_i$ buffer (pH 7.0, 50 mM). The resulting mixture was vortexed, and afterwards 1 mM solution of **9** (1.2 μL, 3 equiv) in DMF was added. The reaction mixture was then shaken for 30 min at 25 ºC. After this time, a 10 μL aliquot of the reaction mixture was analysed by LC–MS.

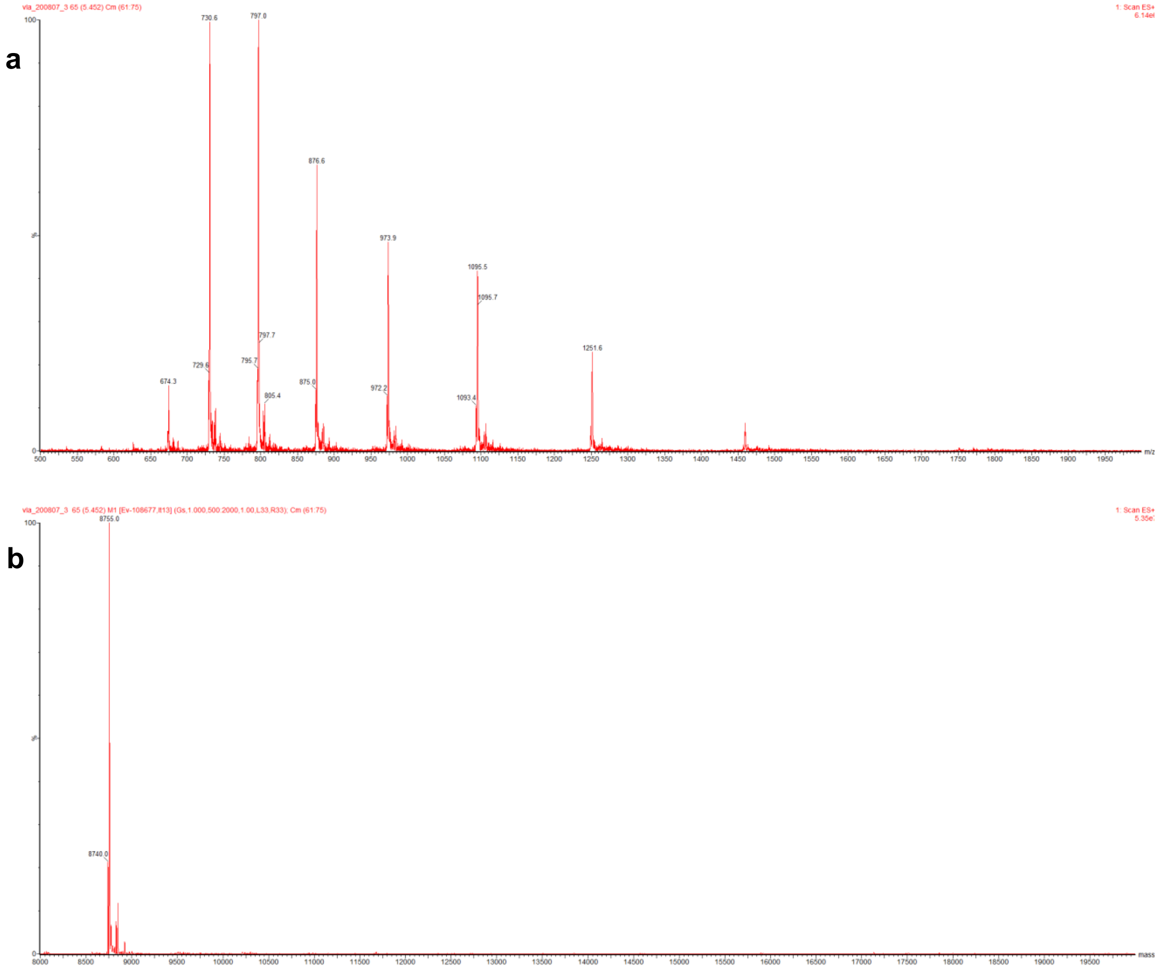

Figure S39. LC–MS spectra of the reaction between Ub-K63C and **9** at pH 9; **a**) ion series and **b**) deconvoluted spectrum.

Conjugation of Cys Containing Proteins with Compound 7

Conjugation Reaction Between HSA and **7**

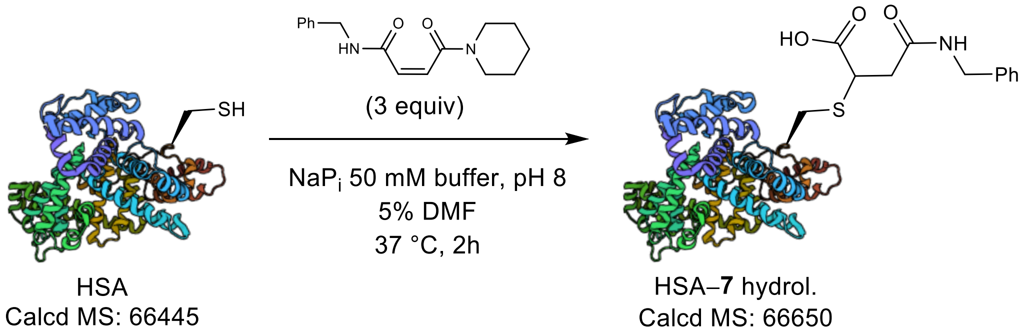

6.7 μL of a stock solution of HSA (150 μM) was added to an eppendorf containing 43.3 μL of NaP$_i$ buffer (pH 8.0, 50 mM). The resulting mixture was vortexed, and afterwards 1 mM solution of **7** (3 μL, 3 equiv) in DMF was added. The reaction mixture was then shaken for 2 h at 37 ºC. After this time, a 10 μL aliquot of the reaction mixture was analysed by LC–MS.

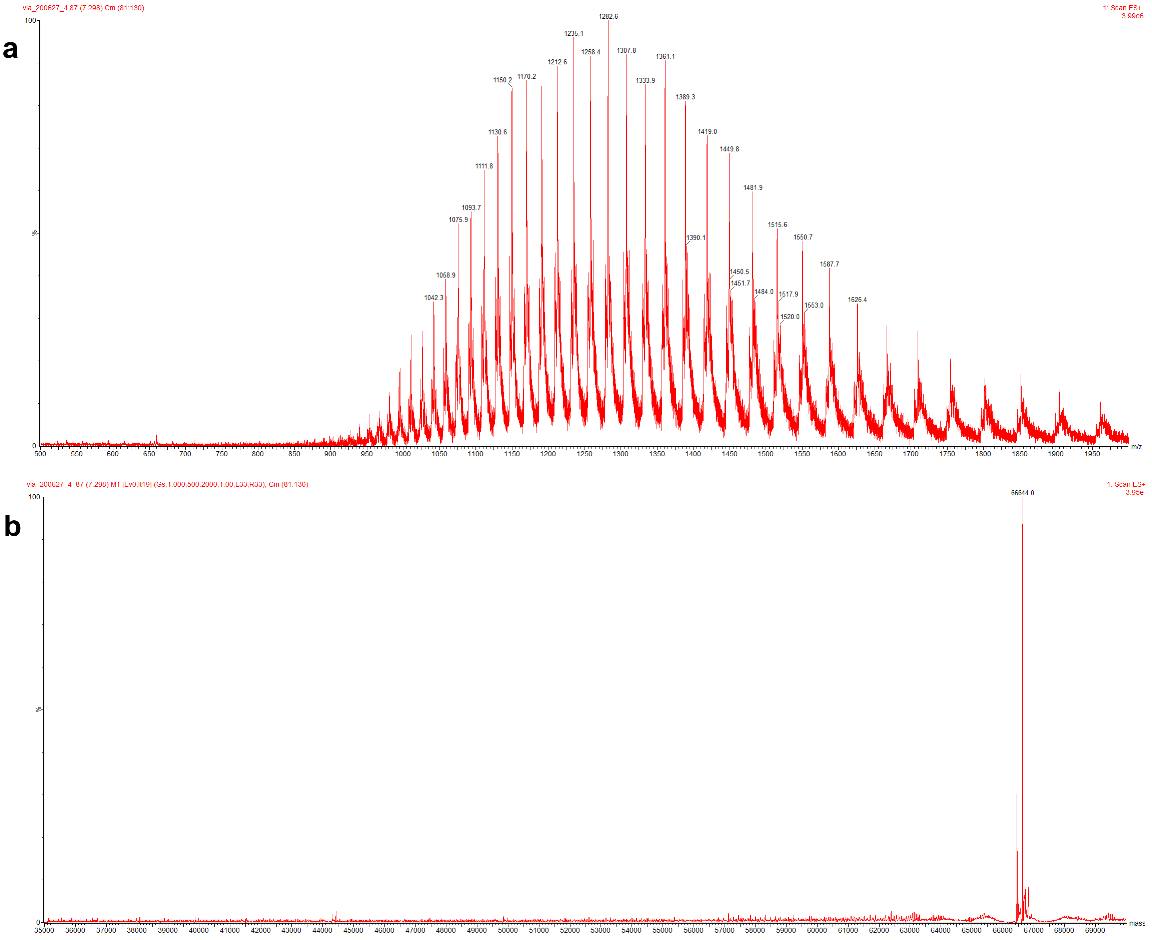

Figure S40. LC–MS spectra of HSA-**7**; **a**) ion series and **b**) deconvoluted spectrum.

Conjugation Reaction Between HET and **7**

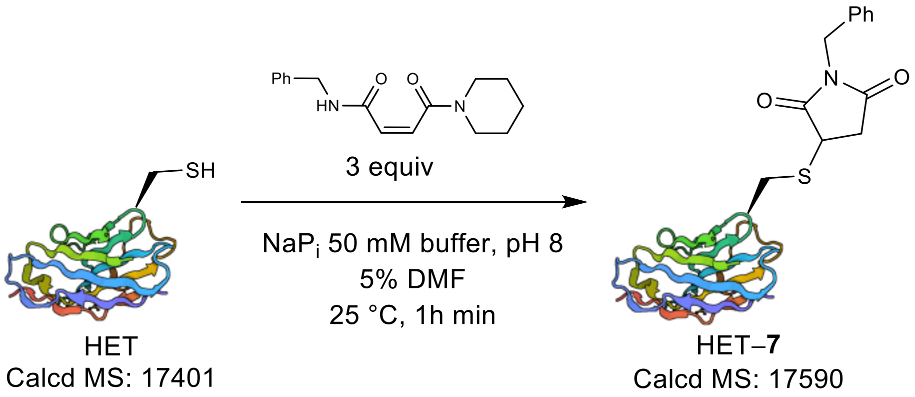

50 µL of a stock solution of HET (21 µM) was added to an eppendorf. 1 mM solution of **7** (3 µL, 3 equiv) in DMF was added. The reaction mixture was then shaken for 30 min at 25 ºC. After this time, a 10 µL aliquot of the reaction mixture was analysed by LC–MS.

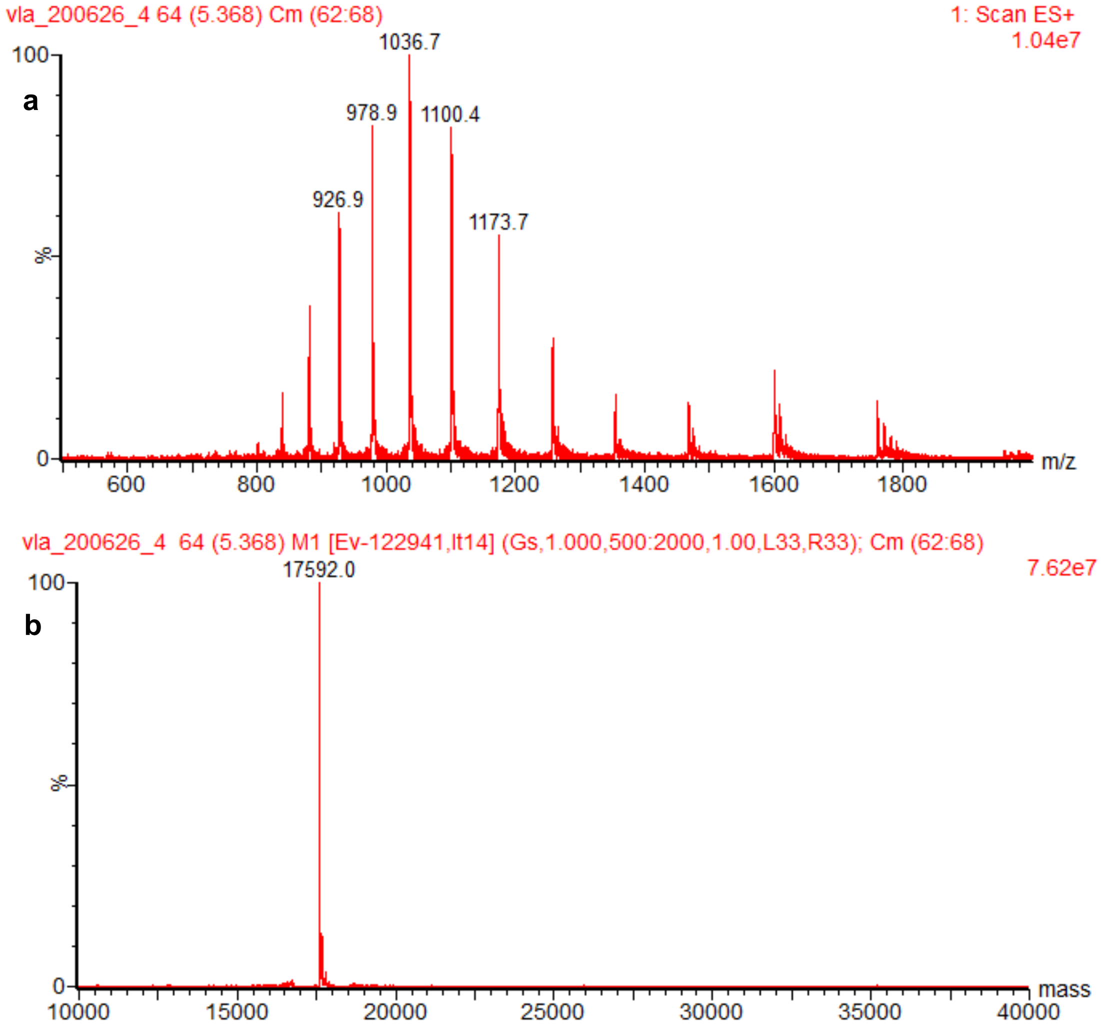

Figure S41. LC–MS spectra of HET-**7**; **a**) ion series and **b**) deconvoluted spectrum.

Conjugation Reaction Between R434 and **7**

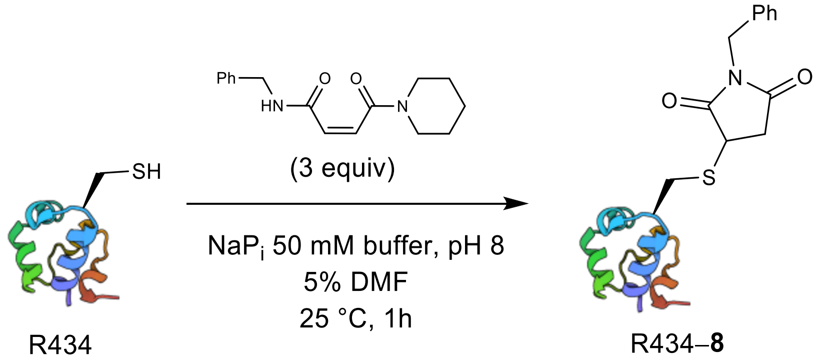

R434
Calcd MS: 8010

R434–**8**
Calcd MS: 8198

9.1 µL of a stock solution of R434-K63C (110 µM) was added to an eppendorf containing 40.9 µl of NaPi buffer (pH 8.0, 50 mM). The resulting mixture was vortexed, and afterwards 1 mM solution of **7** (3 µL, 3 equiv) in DMF was added. The reaction mixture was then shaken for 30 min at 25 ºC. After this time, a 10 µL aliquot of the reaction mixture was analysed by LC–MS.

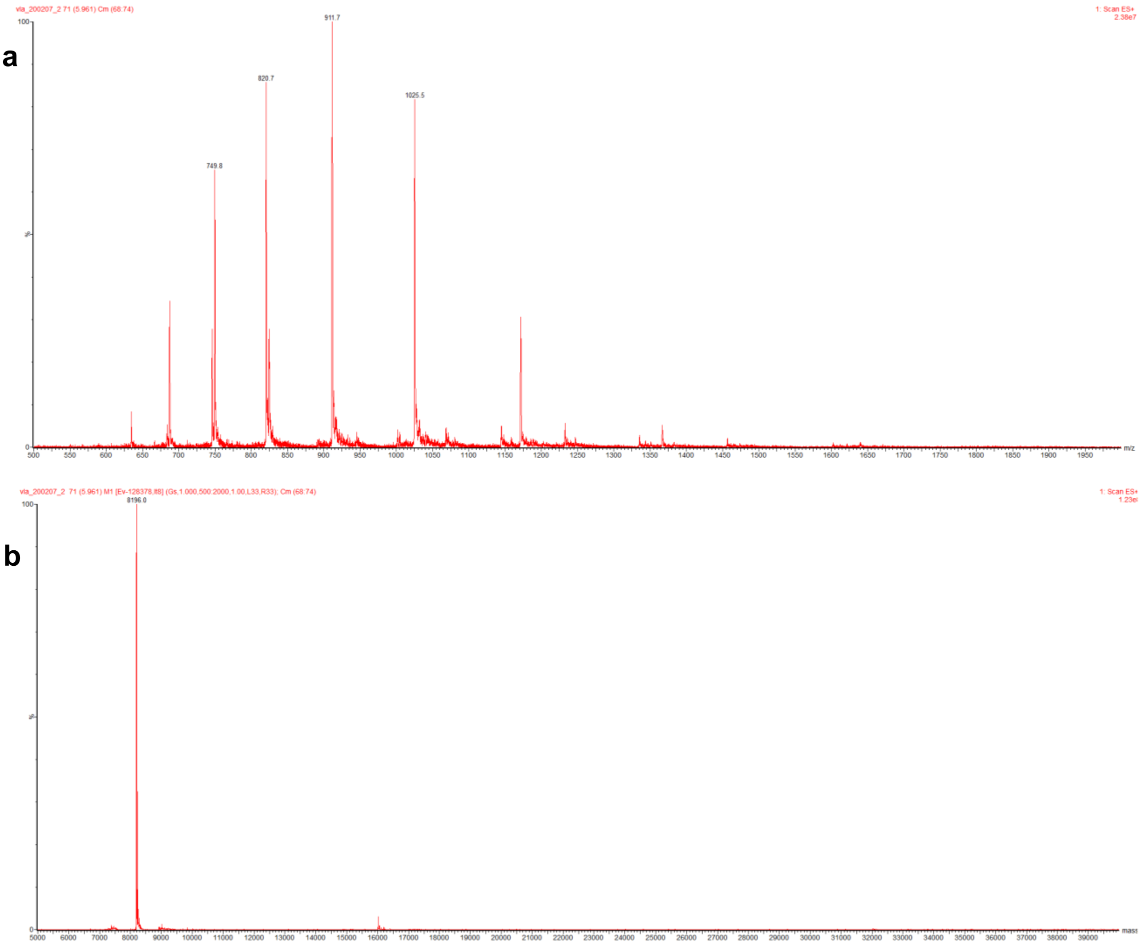

Figure S42. LC–MS spectra of R434-**7**; **a**) ion series and **b**) deconvoluted spectrum.

Conjugation of Ub-K63C with Compounds 11–14

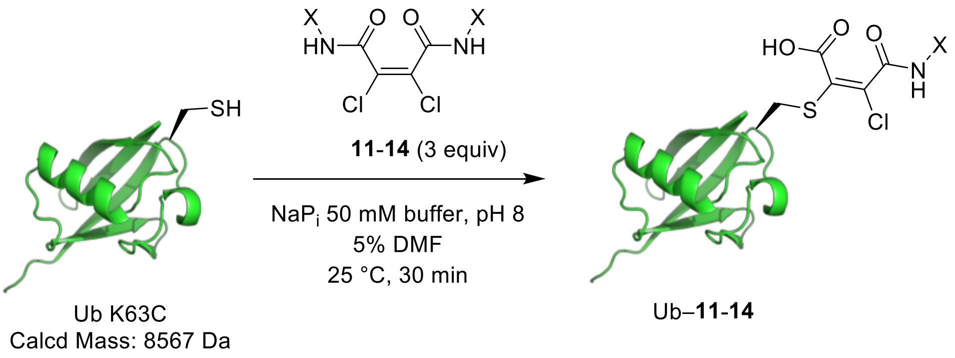

4.4 μL of a stock solution of Ub-K63C (90 μM) was added to an eppendorf containing 15.6 μL of NaP$_i$ buffer (pH 8.0, 50 mM). The resulting mixture was vortexed, and afterwards 1 mM solution of **11–14** (1.2 μL, 3 equiv) in DMF was added. The reaction mixture was then shaken for 30 min at 25 ºC. After this time, a 10 μL aliquot of the reaction mixture was analysed by LC–MS.

Conjugation Reaction Between Ub and **11**

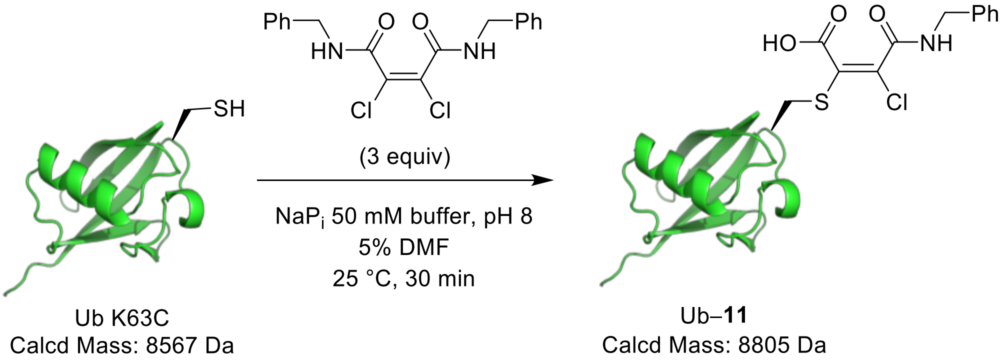

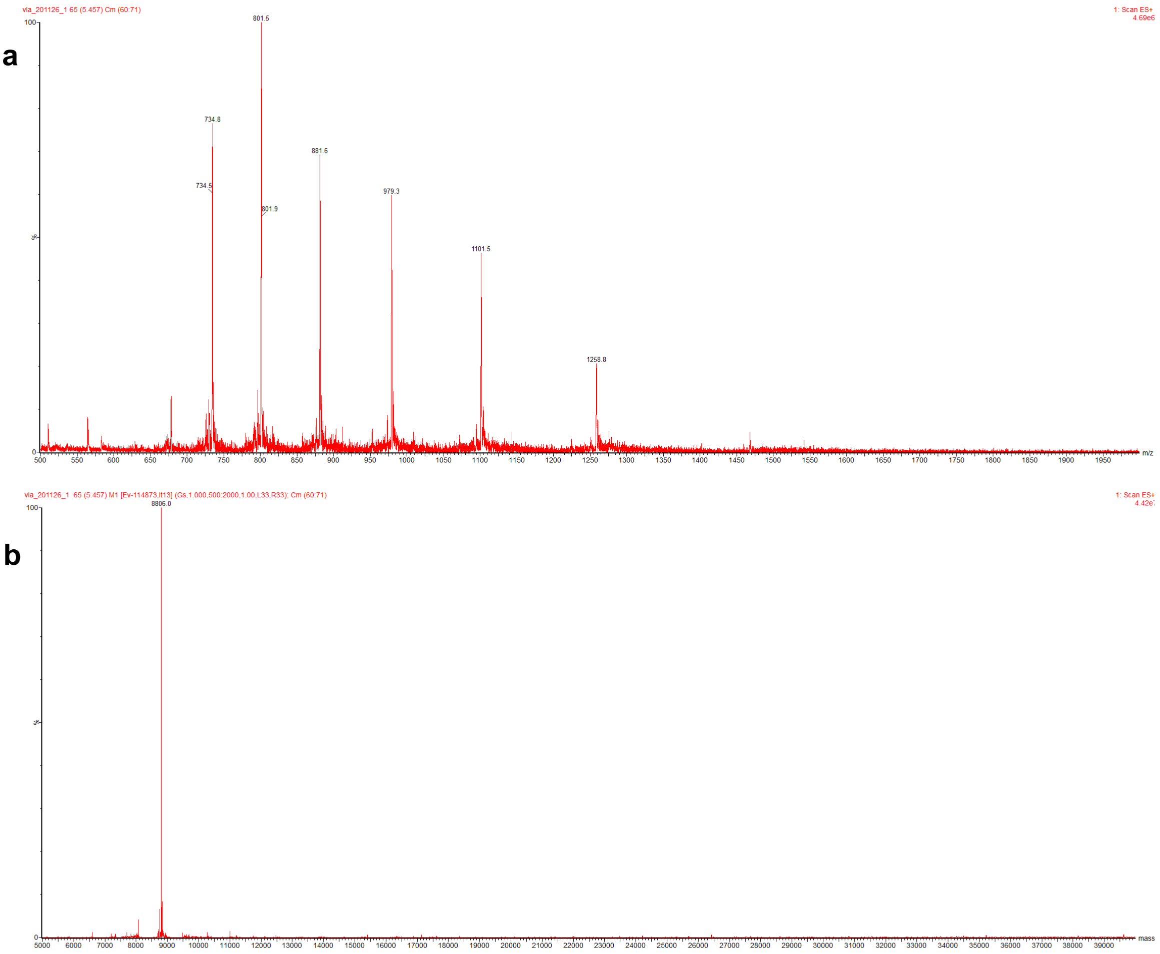

Figure S43. LC–MS spectra of Ub-**11**; **a**) ion series and **b**) deconvoluted spectrum.

Conjugation Reaction Between Ub and **12**

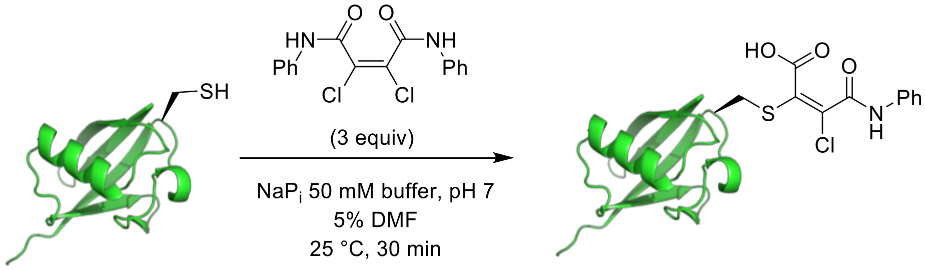

Ub K63C
Calcd Mass: 8567 Da

Ub–**12**
Calcd Mass: 8791 Da

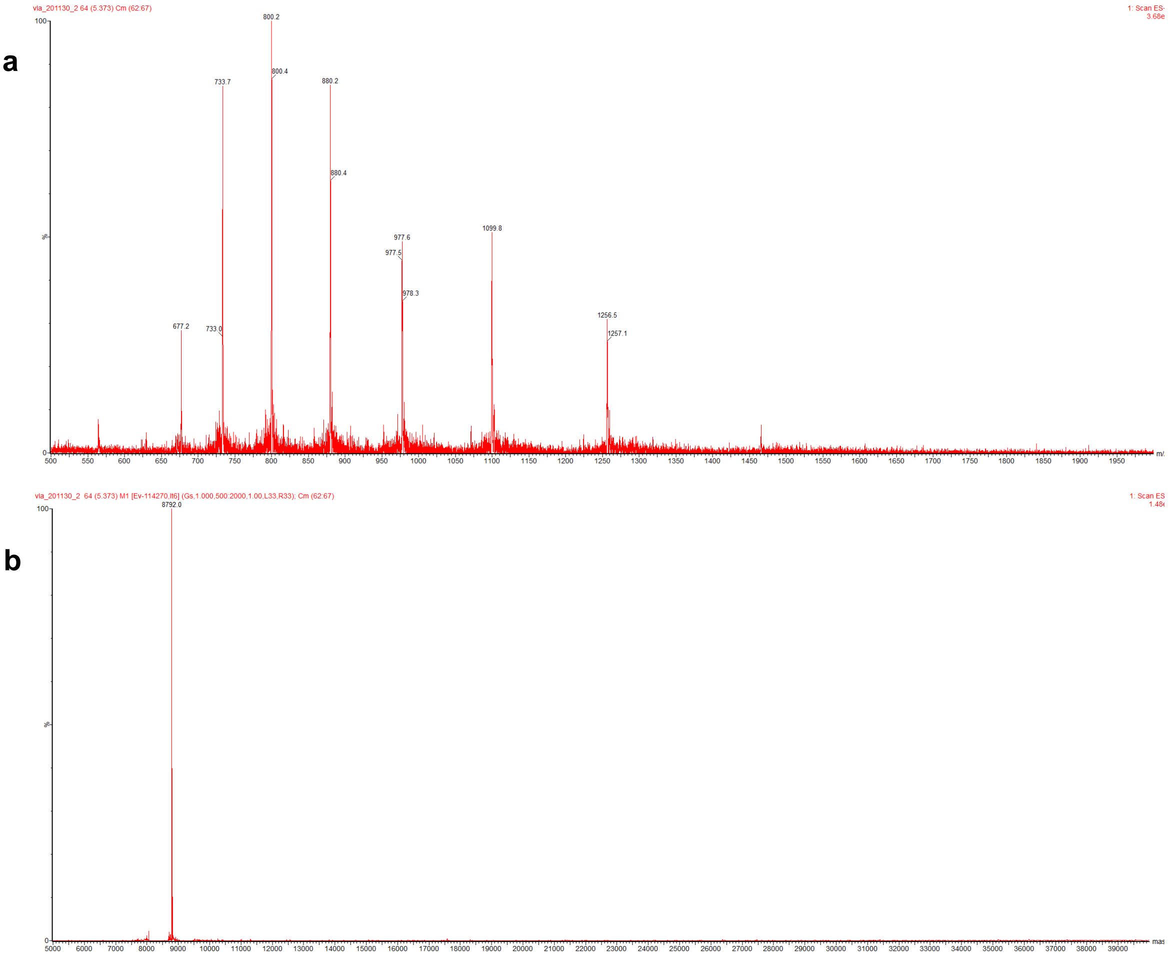

Figure S44. LC–MS spectra of Ub-**12**; **a**) ion series and b) deconvoluted spectrum.

Conjugation Reaction Between Ub and **13**

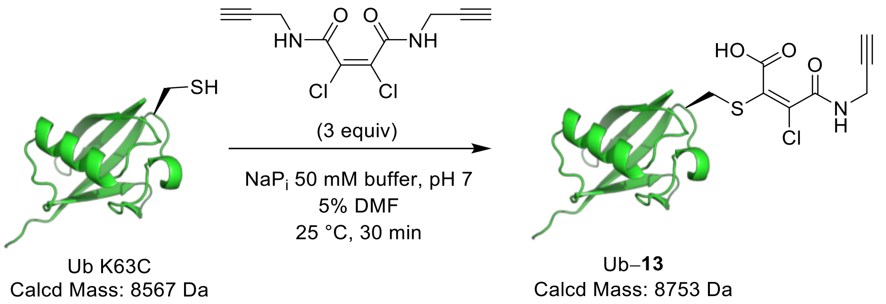

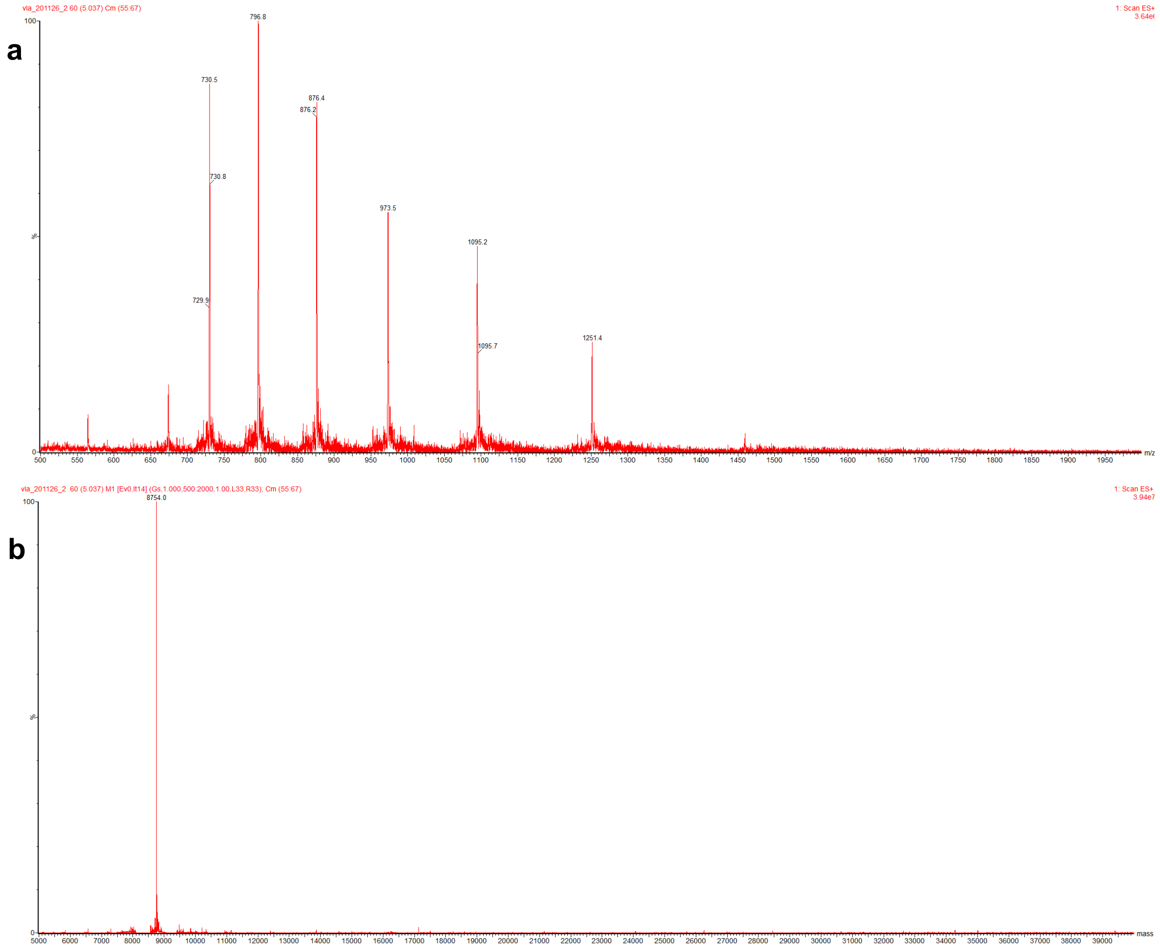

Figure S45. LC–MS spectra of Ub-**13**; **a**) ion series and **b**) deconvoluted spectrum.

Conjugation Reaction Between Ub and **14**

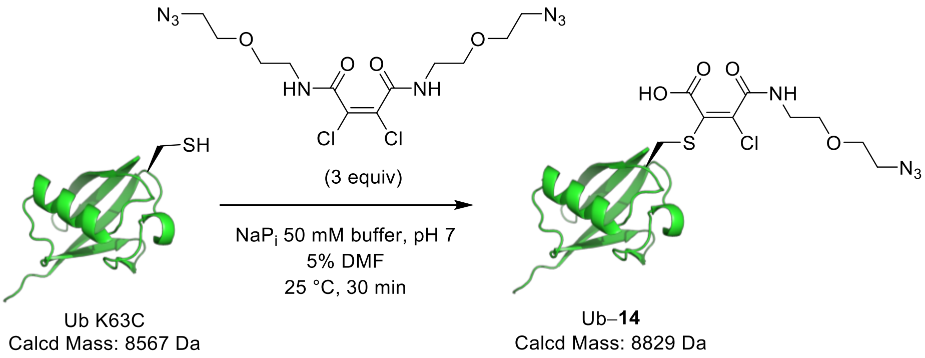

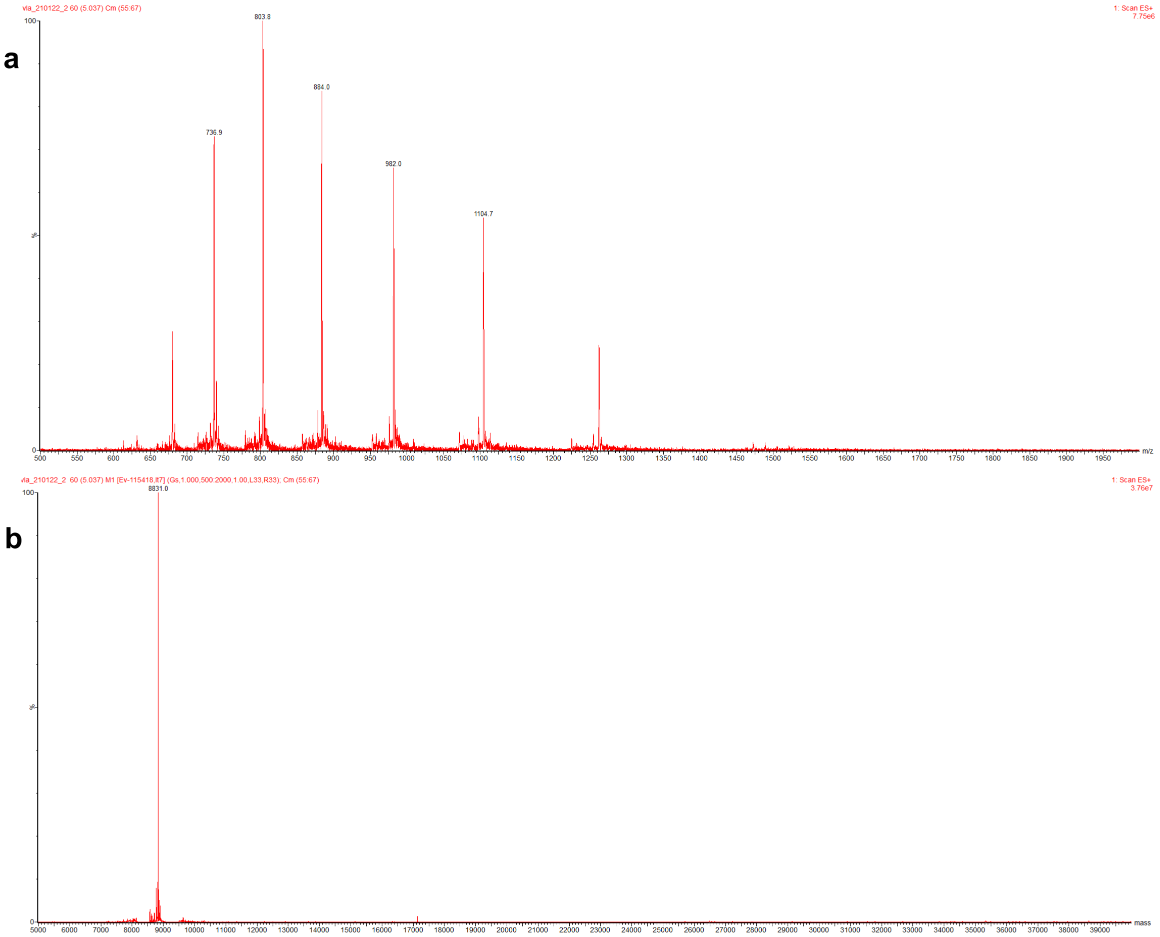

Figure S46. LC–MS spectra of Ub-**14**; **a**) ion series and **b**) deconvoluted spectrum.

Conjugation of Cys Containing Proteins with Compound 14

Conjugation Reaction Between C2am and **14**

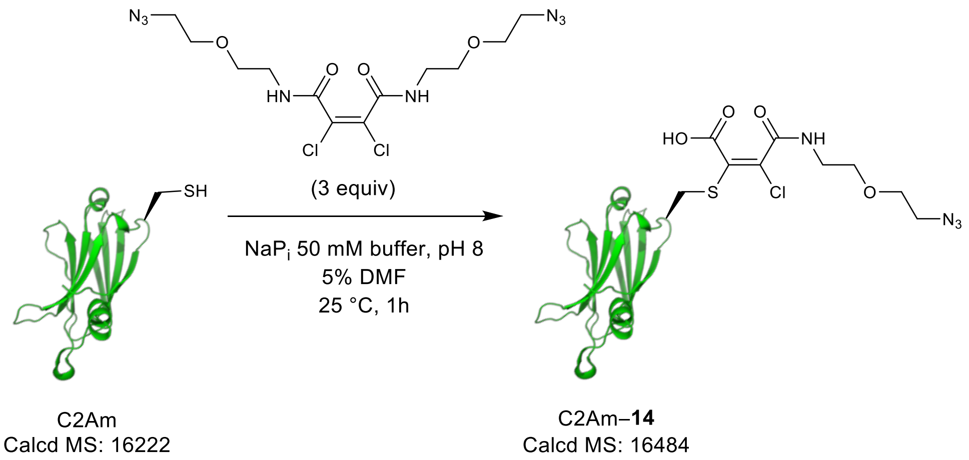

7.9 μL of a stock solution of C2Am (127 μM) was added to an eppendorf containing 42 μL of NaPi buffer (pH 8.0, 50 mM). 1 μL of a TCEP solution (10 mM, 10 equiv) was added and the mixture was stirred for 1 h at 25 ºC. After this, the small molecules were removed using a desalting column and a 1 mM solution of **14** (3 μL, 3 equiv) in DMF was added. The reaction mixture was then shaken for 1 h at 25 ºC. After this time, a 10 μL aliquot of the reaction mixture was analysed by LC–MS.

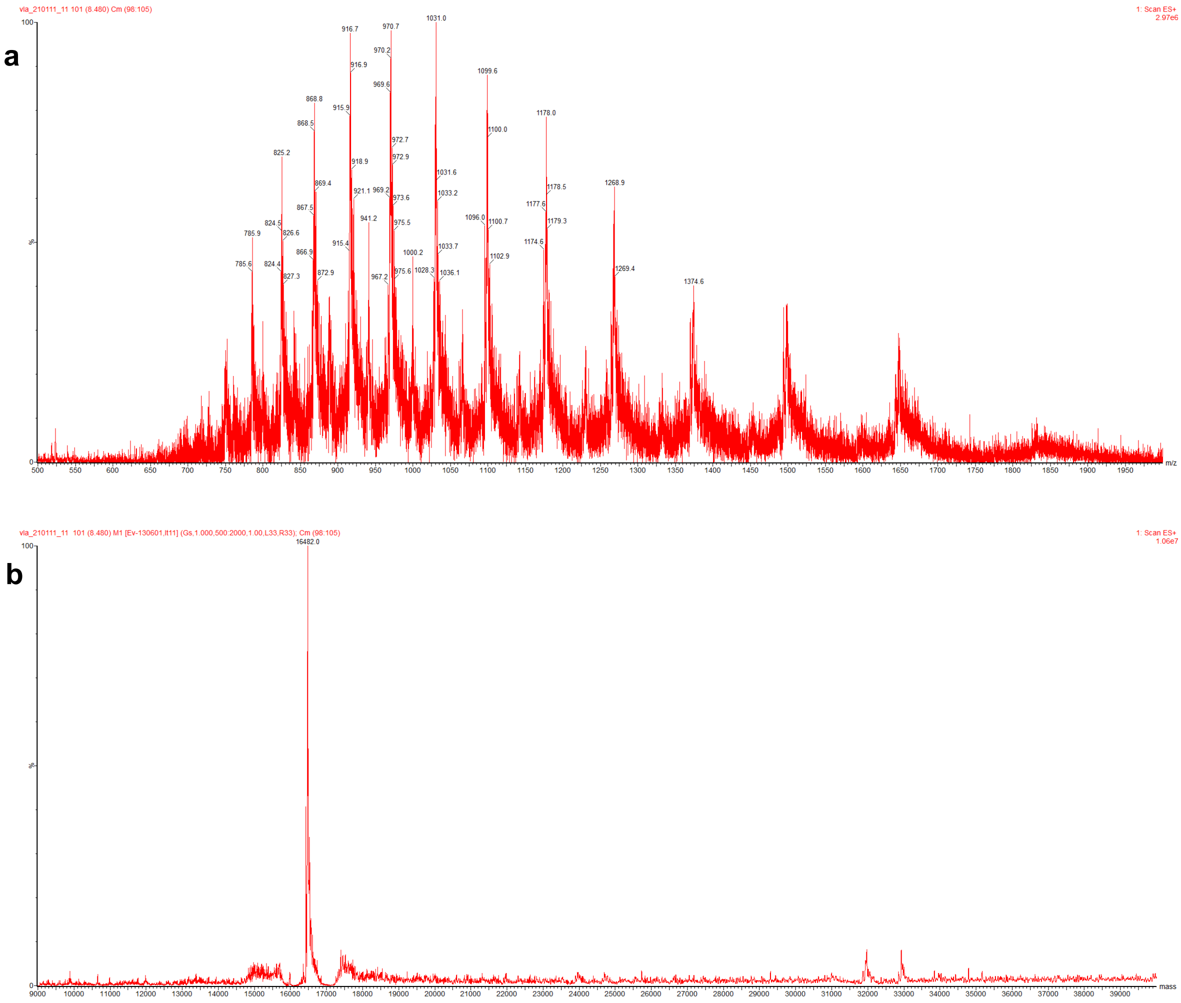

Figure S47. LC–MS spectra of C2Am-**14**; **a**) ion series and **b**) deconvoluted spectrum.

Conjugation Reaction Between ASCATN and **14**

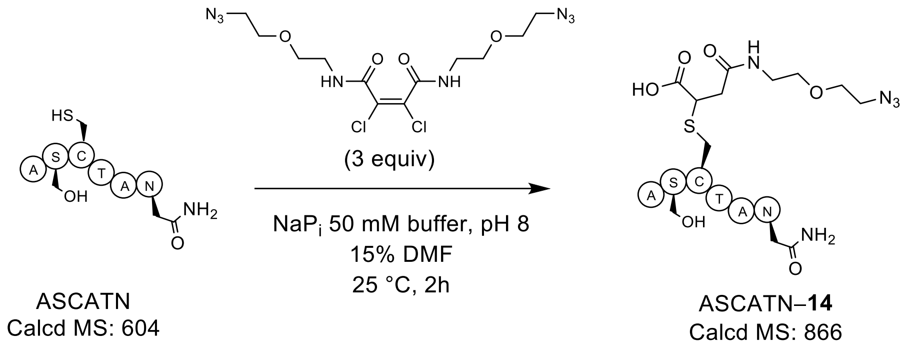

10 μL of a stock solution of ASCATN (1 mM) were diluted down to 500 μM using NaP$_i$ buffer (pH 8.0, 50 mM). The resulting mixture was vortexed, and afterwards 3 μL of a 10 mM solution of **14** (3 equiv) in DMF was added. The reaction mixture was then shaken for 2 h at 25 ºC. After this time, a 10 μL aliquot of the reaction mixture was analysed by LC–MS.

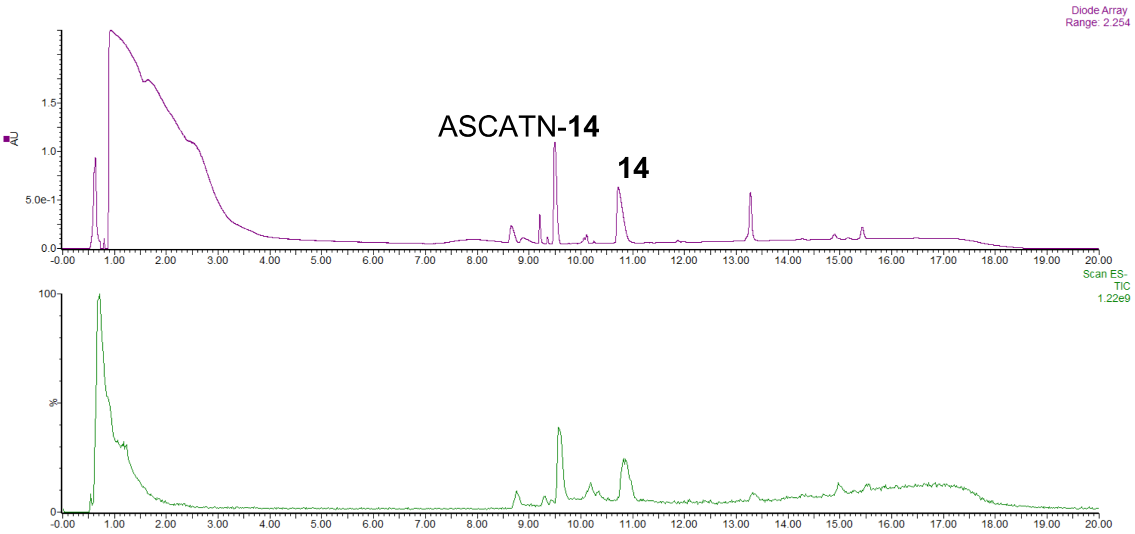

Figure S48. LC–MS UV trace negative and ion scan of ASCATN-**14**.

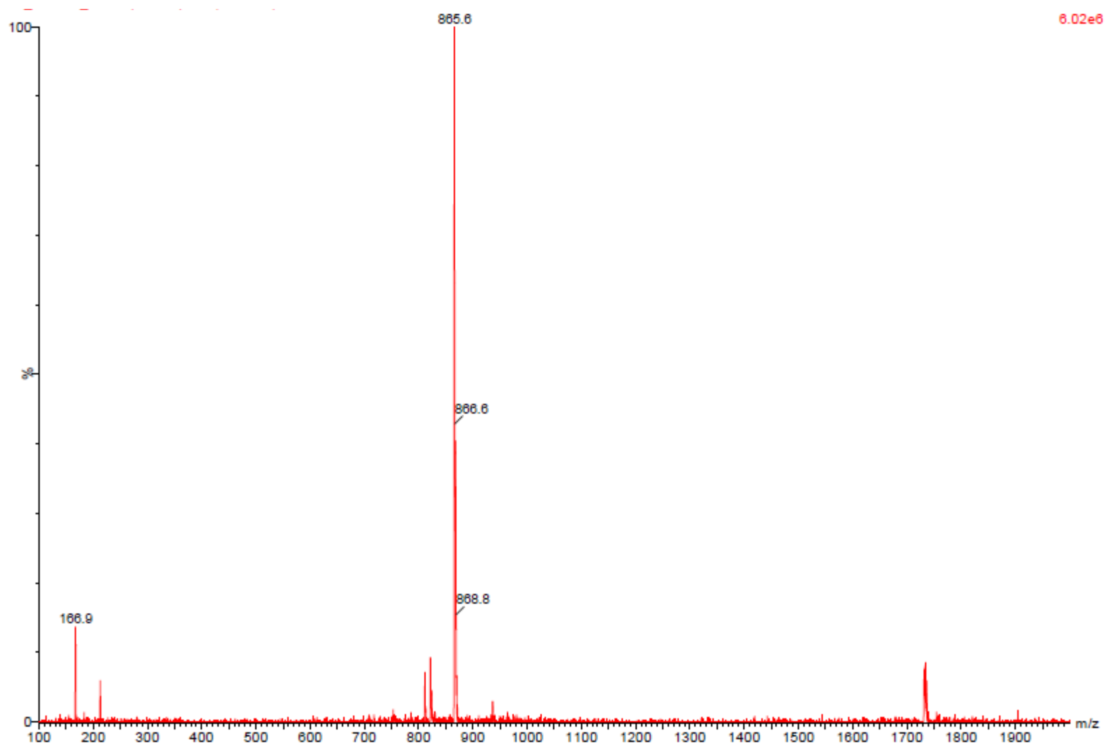

Figure S49. Ion series of main peak at 9.5 min of ASCATN-**14**.

Conjugation Reaction Between HSA and **14**

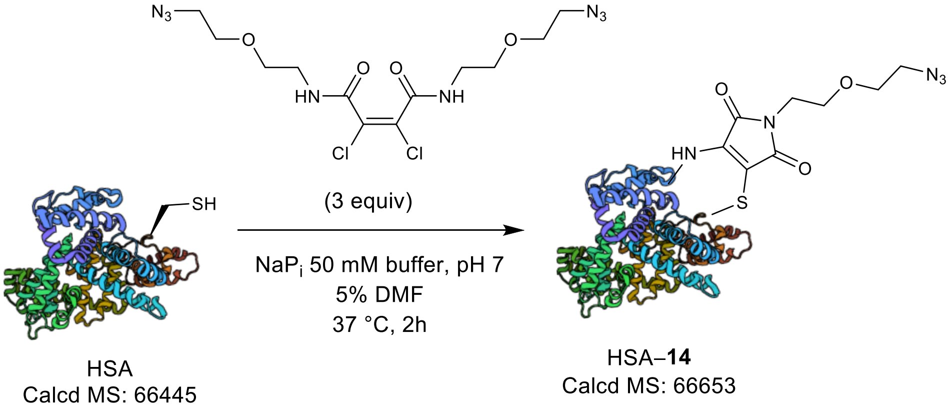

6.7 μL of a stock solution of HSA (150 μM) was added to an eppendorf containing 43 μL of NaP$_i$ buffer (pH 7.0, 50 mM). The resulting mixture was vortexed, and afterwards 1 mM solution of **14** (3 μL, 3 equiv) in DMF was added. The reaction mixture was then shaken for 2 h at 37 ºC. After this time, a 10 μL aliquot of the reaction mixture was analysed by LC–MS.

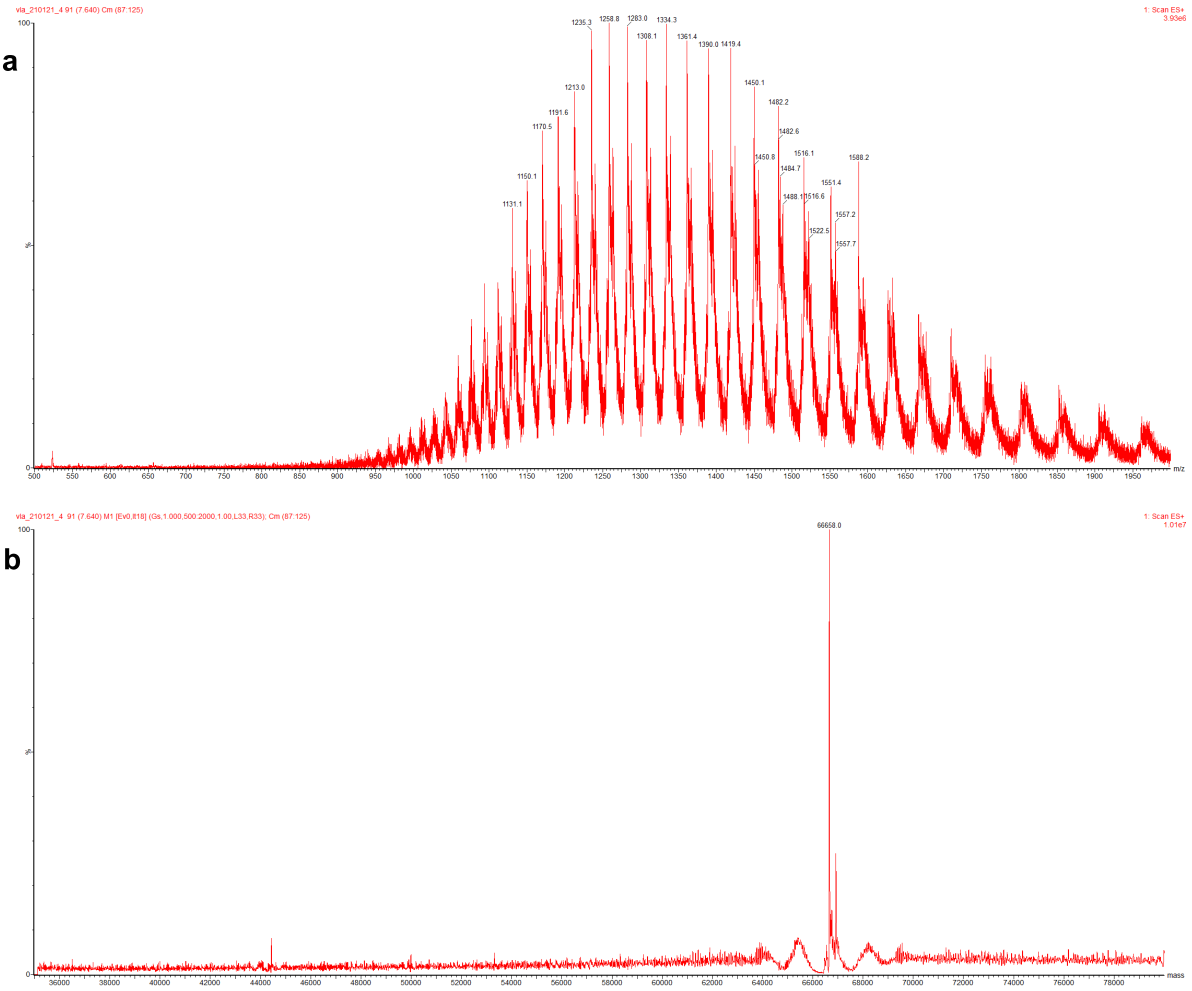

Figure S50. LC–MS spectra of HSA-**14**; **a**) ion series and **b**) deconvoluted spectrum.

Conjugation Reaction Between H3K4C and **14**

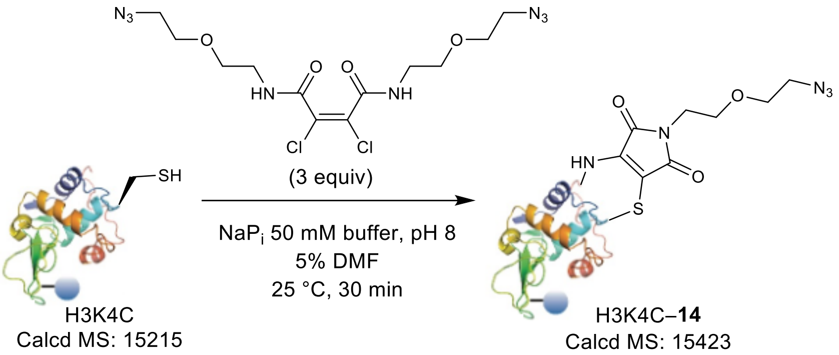

10 µL of a stock solution of H3K4C (100 µM) was added to an eppendorf containing 40 µL of NaP_i buffer (pH 8.0, 50 mM). 1 µL of a TCEP solution (10 mM, 10 equiv) was added and the mixture was stirred for 1 h at 25 ºC. After this, the small molecules were removed using a desalting column and a 1 mM solution of **14** (3 µL, 3 equiv) in DMF was added. The reaction mixture was then shaken for 30 min at 25 °C. After this time, a 10 µL aliquot of the reaction mixture was analysed by LC–MS.

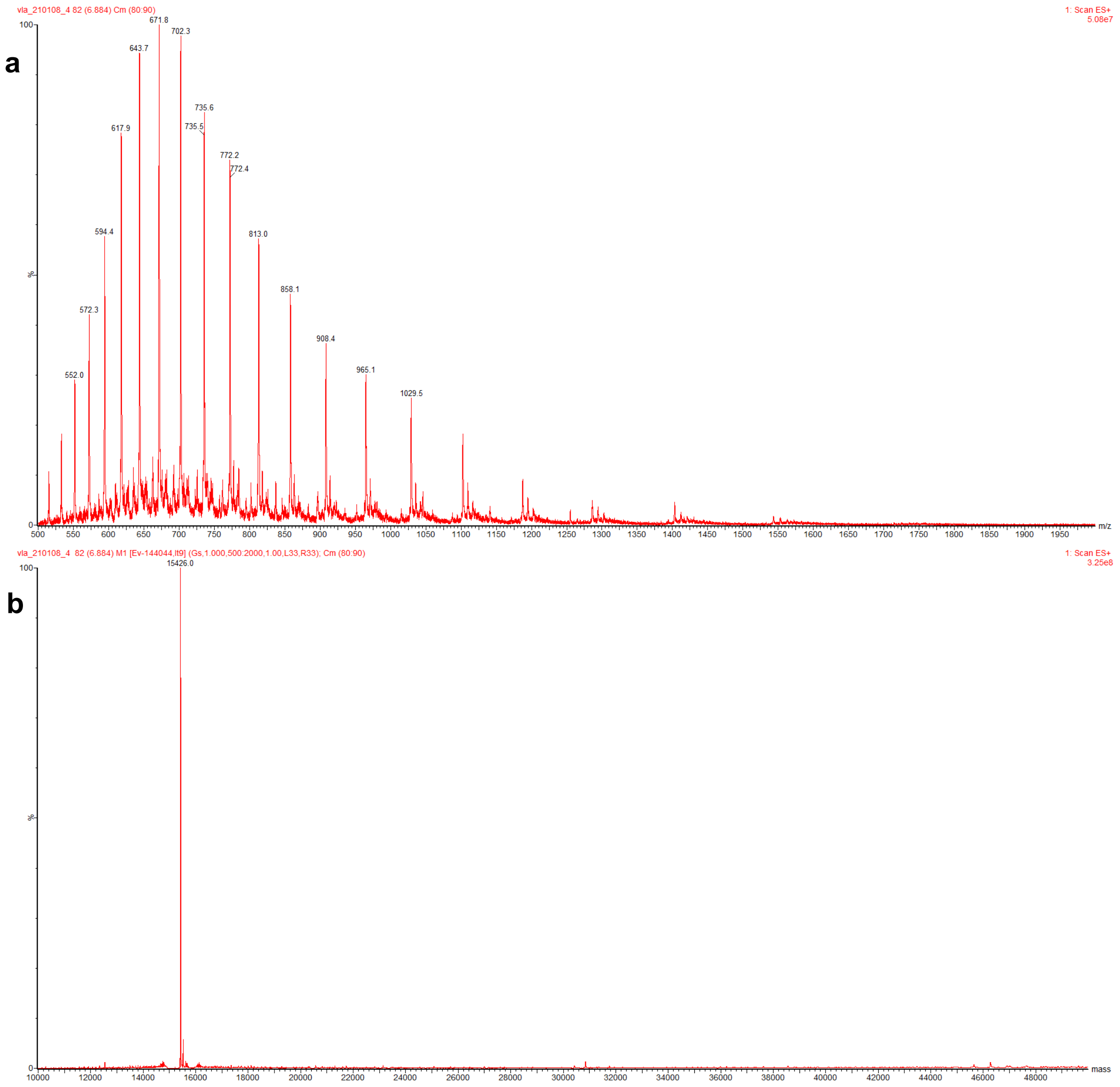

Figure S51. LC–MS spectra of H3K4C-**14**; **a**) ion series and b) deconvoluted spectrum.

Conjugation Reaction Between HET and **14**

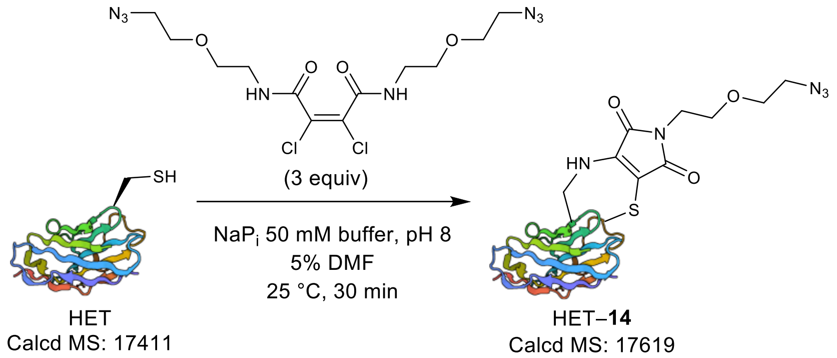

50 μL of a stock solution of HET (21 μM) was mixed in an eppendorf with a 1 mM solution of **14** (3 μL, 3 equiv) in DMF. The reaction mixture was then shaken for 30 min at 25 ºC. After this time, a 10 μL aliquot of the reaction mixture was analysed by LC–MS.

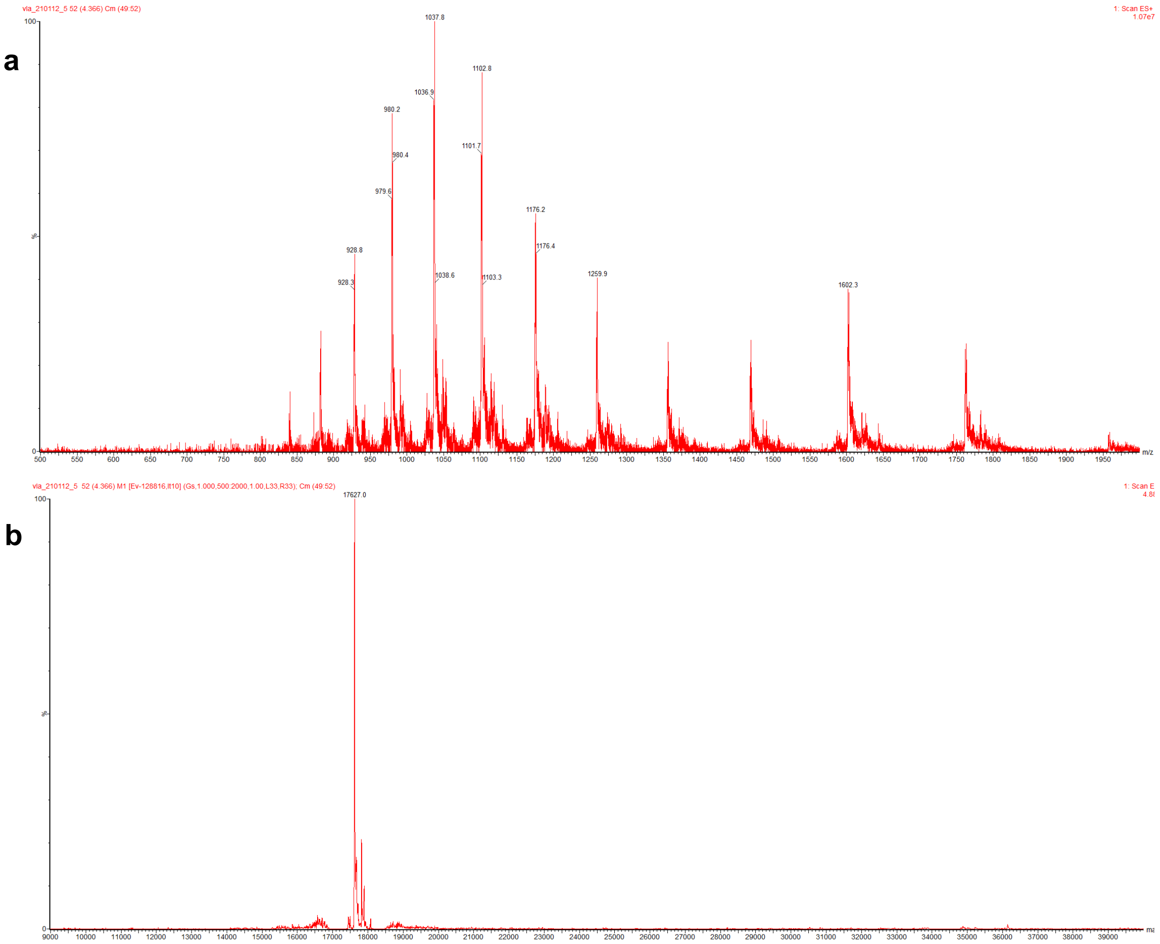

Figure S52. LC–MS spectra of HET-**14**; **a**) ion series and b) deconvoluted spectrum.

Conjugation Reaction Between Gemtuzumab V205C and **14**

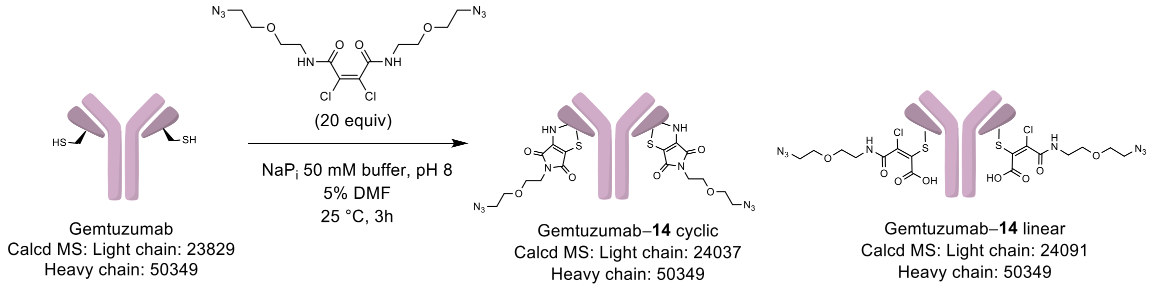

33 µL of a stock solution of Gemtuzumab V205C (30 µM) was added to an eppendorf containing 17 µL of NaPi buffer (pH 8.0, 50 mM). After this 2 µL of a solution of **14** (10 mM, 20 equiv, 10 equiv for each cysteine) is added and the mixture is then shaken for 3 h at 25 ºC. After this time, a 10 µL aliquot of the reaction mixture was reduced with TCEP (20 equiv, 25 ºC, 30 min) and analysed by LC–MS.

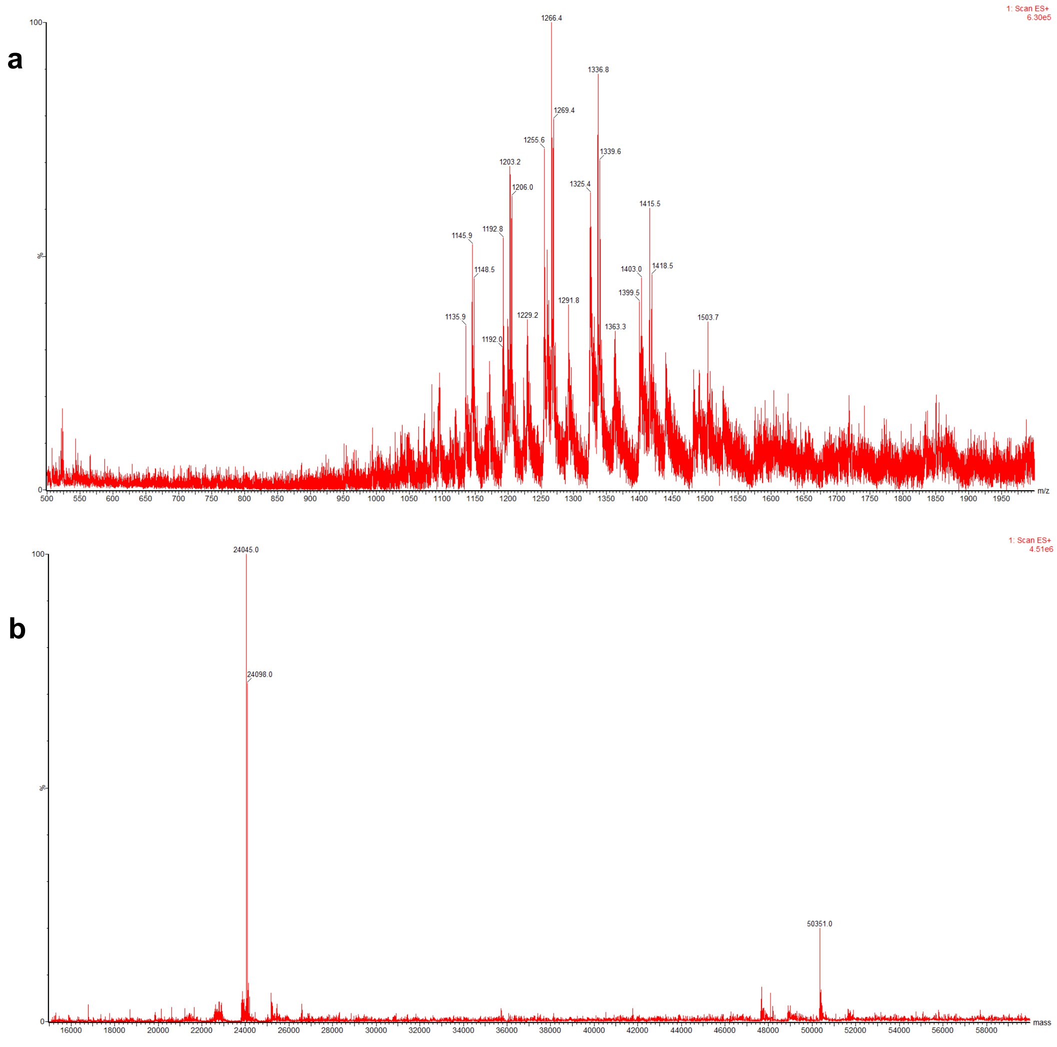

Figure S53. LC–MS spectra of Gemtuzumab-**14**; **a**) ion series and **b**) deconvoluted spectrum.

Conjugation Reaction Between Trastuzumab V205C and **14**

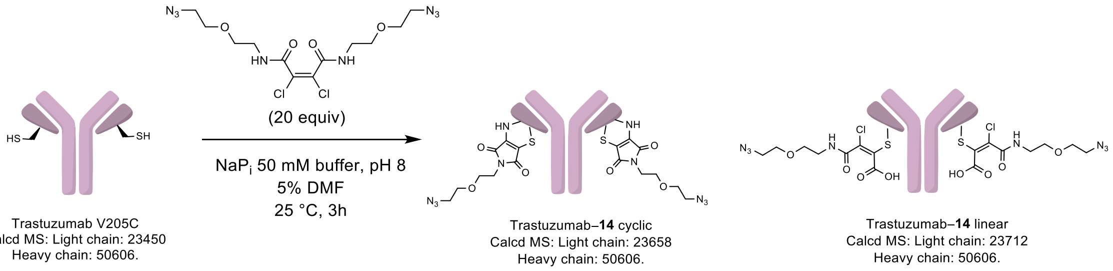

18 µL of a stock solution of Trastuzumab V205C (55 µM) was added to an eppendorf containing 32 µL of NaP$_i$ buffer (pH 8.0, 50 mM). After this 2 µL of a solution of **14** (10 mM, 20 equiv, 10 equiv for each cysteine) is added and the mixture is then shaken for 3 h at 25 ºC. After this time, a 10 µL aliquot of the reaction mixture was reduced with TCEP (20 equiv, 25 ºC, 30 min) and analysed by LC–MS.

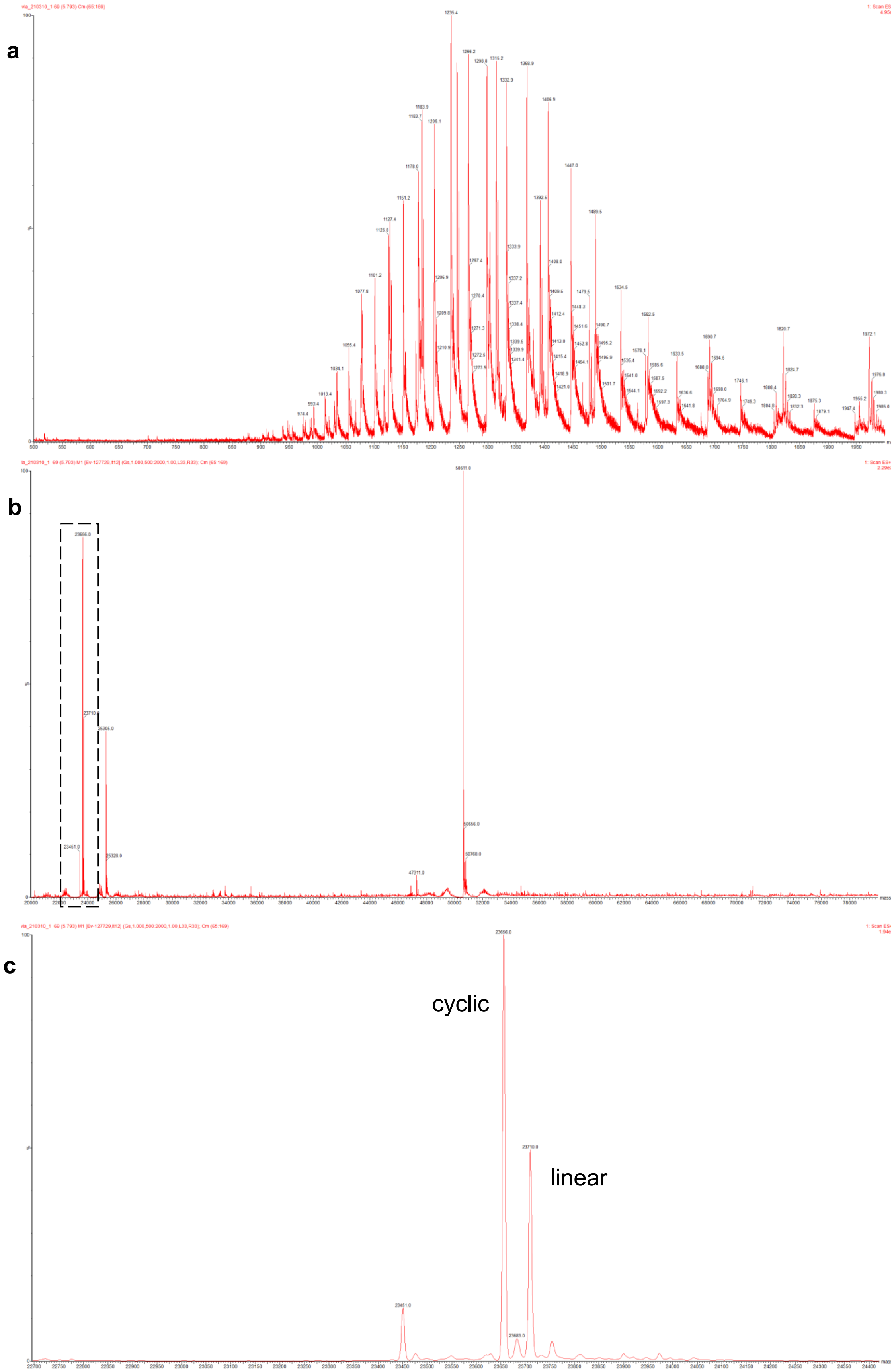

Figure S54. LC–MS spectra of Trastuzumab-**14; a**) ion series, **b**) deconvoluted spectrum and **c**) highlighted deconvoluted area around light chain modification.

Conjugation Reaction Between Trastuzumab V205C, K207A and **14**

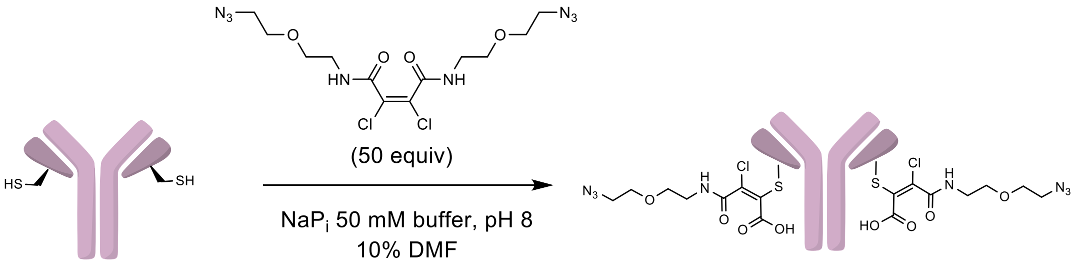

Trastuzumab V205C K207A
Calcd MS: Light chain: 23391
Heavy chain: 50634.

Trastuzumab V205C K207A–**14**
Calcd MS: Light chain: 23653
Heavy chain: 50634.

15 µL of a stock solution of Trastuzumab V205C, K207A (65 µM) was added to an eppendorf containing 35 µL of NaPi buffer (pH 8.0, 50 mM). After this, 5 µL of a solution of **14** (10 mM, 50 equiv, 25 equiv for each cysteine) is added and the mixture is then shaken for 12 h at 25 ºC. After this time, a 10 µL aliquot of the reaction mixture was reduced with TCEP (20 equiv, 25 ºC, 30 min) and analysed by LC–MS.

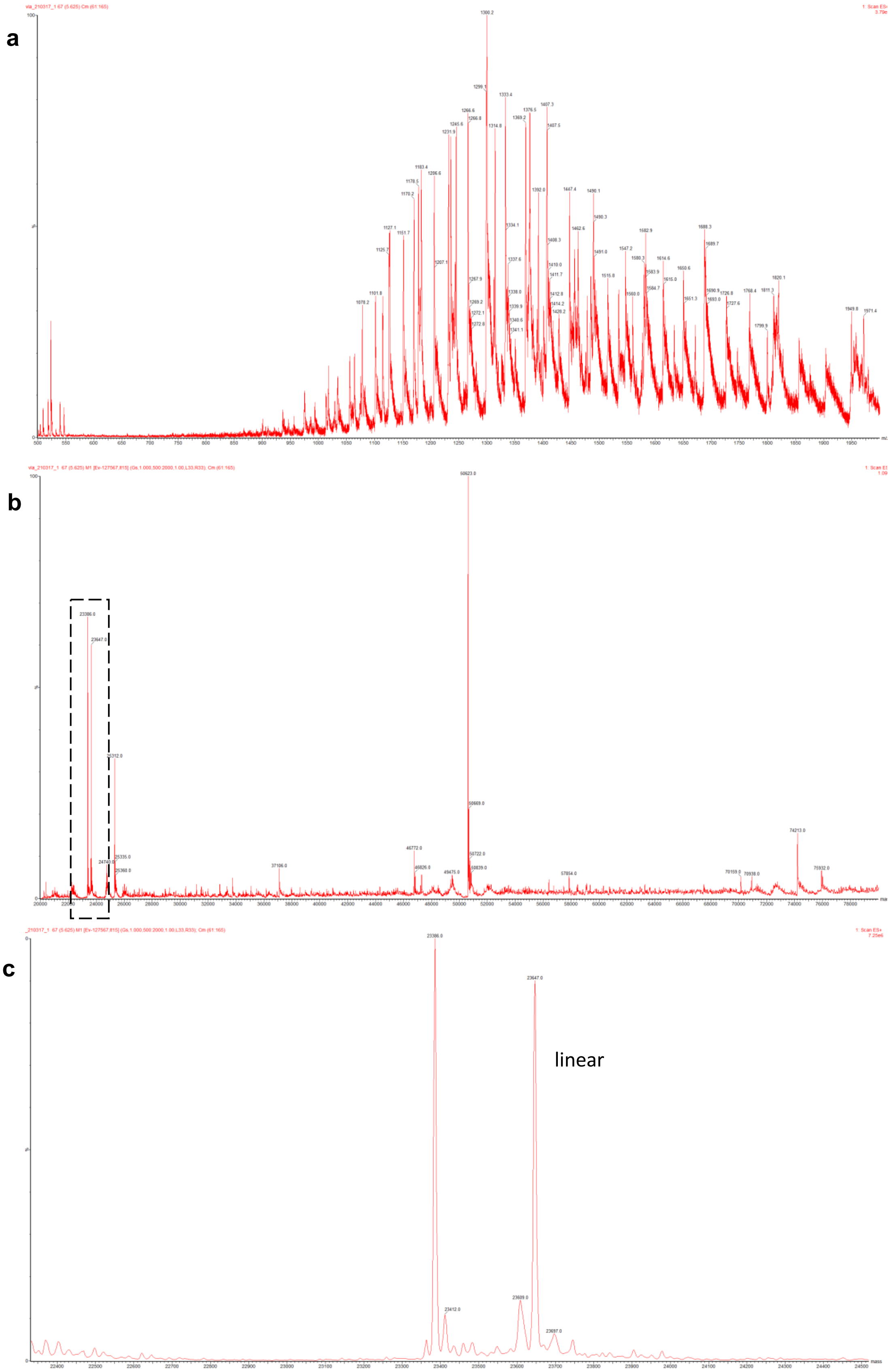

Figure S55. LC–MS spectra of Trastuzumab V205C, K207A-**14**; **a**) ion series, **b**) deconvoluted spectrum and **c**) highlighted deconvoluted area around light chain modification.

Conjugation Reaction Between Fc Fragments and **14**

239iC

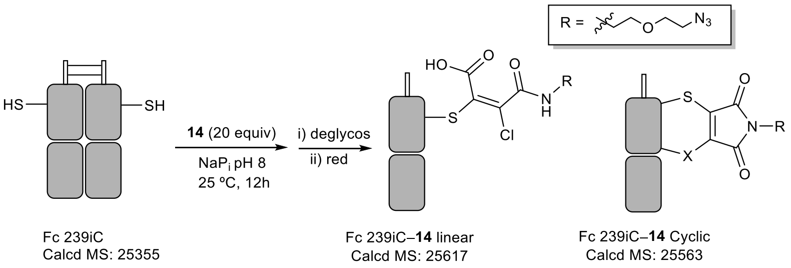

9.5 μL of a stock solution of Fc fragment 239iC (105 μM) was added to an eppendorf containing 40.5 μL of NaP_i buffer (pH 8.0, 50 mM). After this, 2 μL of a solution of **14** (10 mM, 20 equiv, 10 equiv for each cysteine) is added and the mixture is then shaken for 12 h at 25 ºC. Then, a 10 μL aliquot of the reaction mixture is deglycosylated using a PNGase F Glycan Cleavage Kit, reduced by adding 20 equiv of TCEP (30 min at 25 ºC) and analysed by LC–MS.

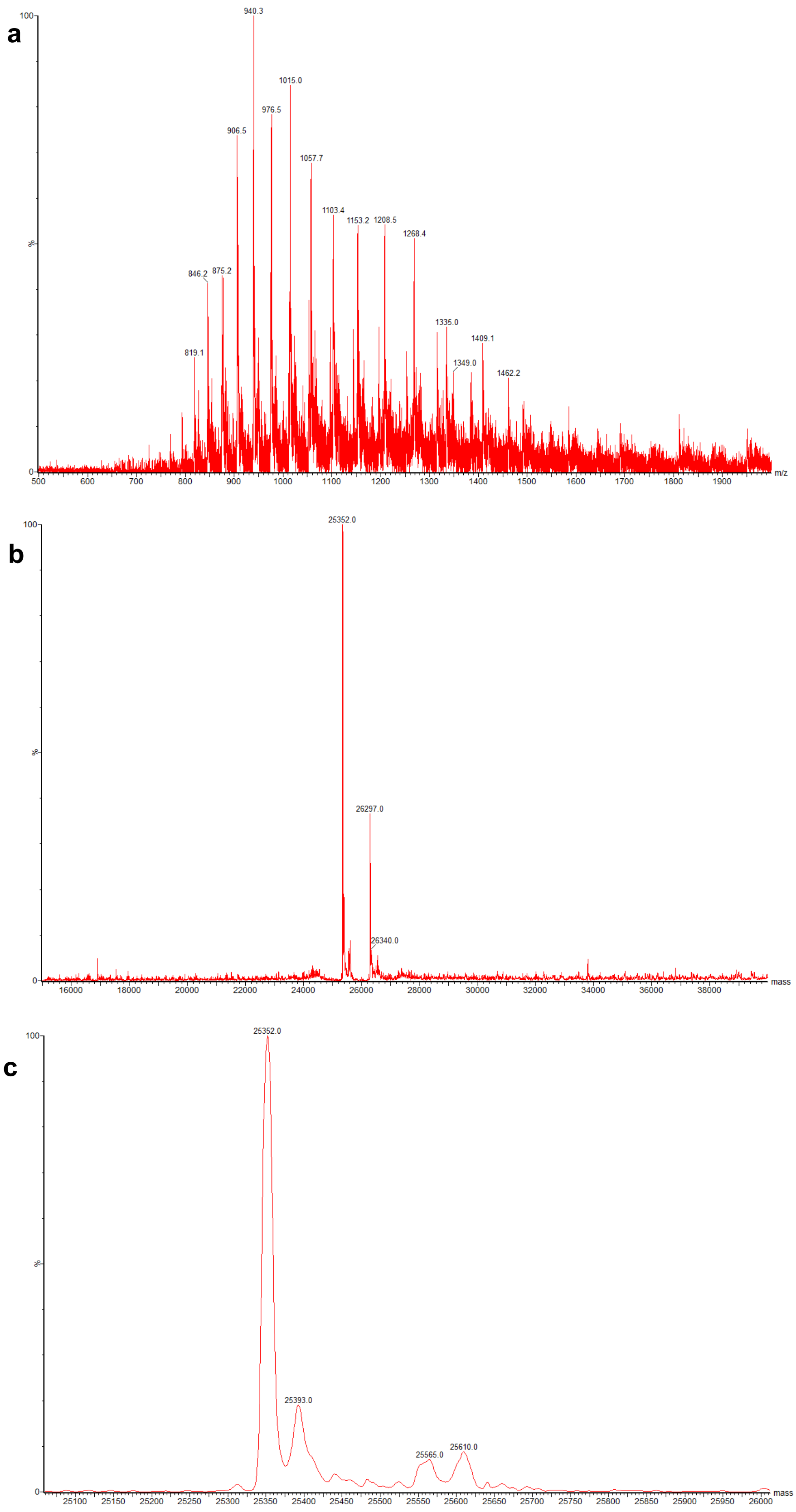

Figure S56. LC–MS spectra of Fc 239iC after failed conjugation to **14**; **a**) ion series, **b**) deconvoluted spectrum and **c**) highlighted deconvoluted area around modification.

268C

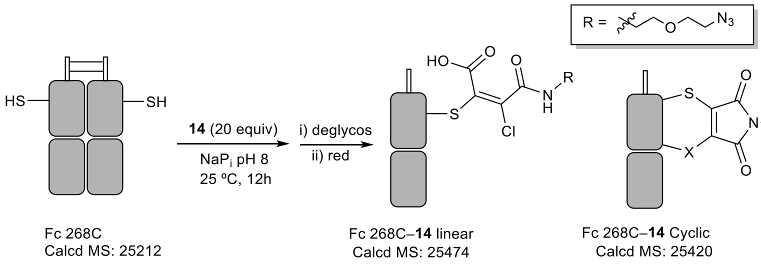

5.7 µL of a stock solution of Fc fragment 268C (175 µM) was added to an eppendorf containing 44.3 µL of NaP_i buffer (pH 8.0, 50 mM). After this, 2 µL of a solution of **14** (10 mM, 20 equiv, 10 equiv for each cysteine) is added and the mixture is then shaken for 12 h at 25 ºC. Then, a 10 µL aliquot of the reaction mixture is deglycosylated using a PNGase F Glycan Cleavage Kit, reduced by adding 20 equiv of TCEP (30 min at 25 ºC) and analysed by LC–MS.

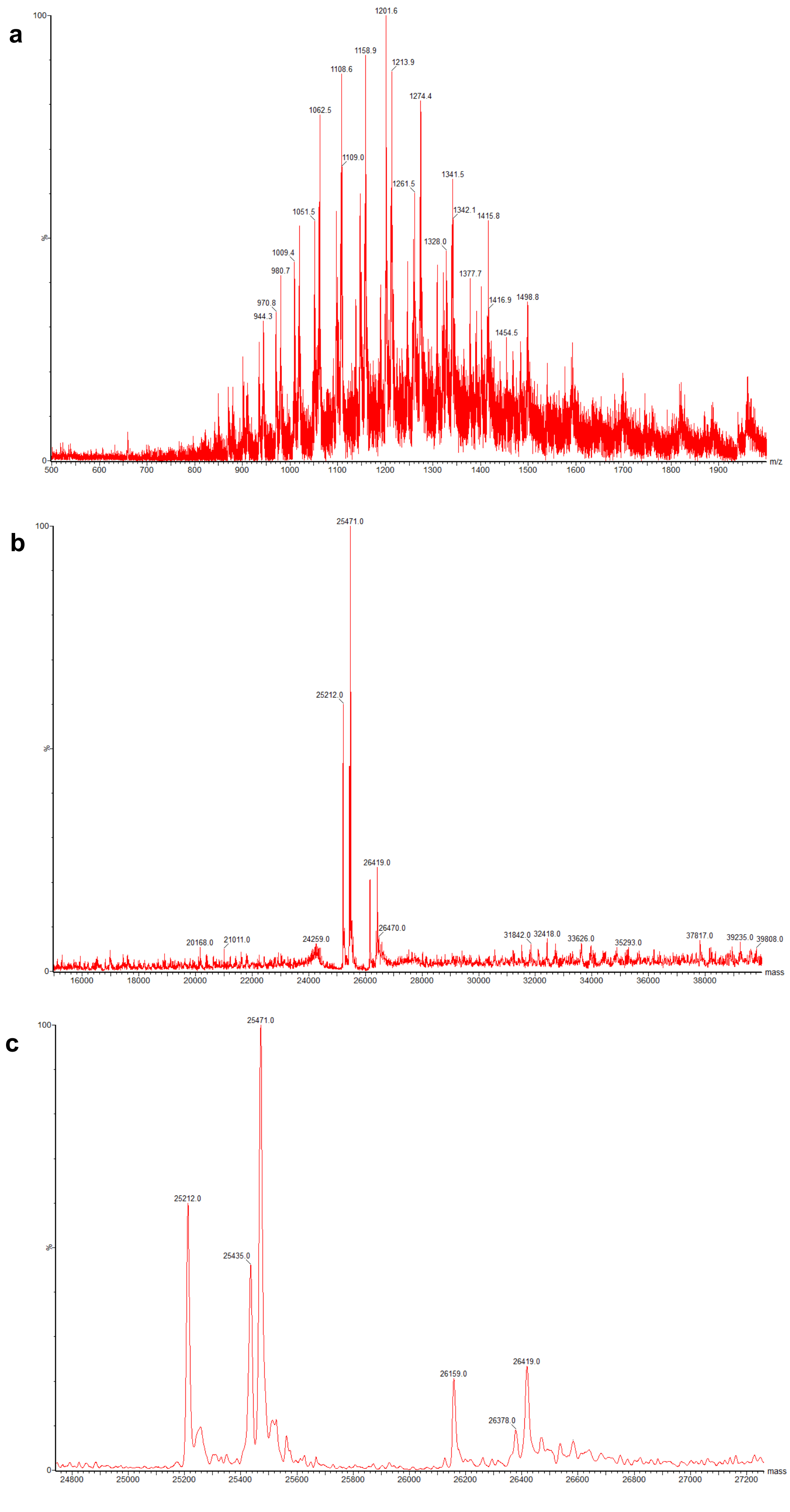

Figure S57. LC–MS spectra of Fc 268C-**14**; **a**) ion series, **b**) deconvoluted spectrum and **c**) highlighted deconvoluted area around modification.

274C

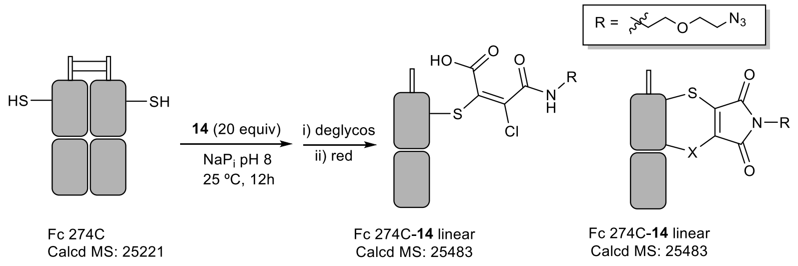

9 µL of a stock solution of Fc fragment 274C (110 µM) was added to an eppendorf containing 41 µL of NaP_i buffer (pH 8.0, 50 mM). After this, 2 µL of a solution of **14** (10 mM, 20 equiv, 10 equiv for each cysteine) is added and the mixture is then shaken for 12 h at 25 ºC. Then, a 10 µL aliquot of the reaction mixture is deglycosylated using a PNGase F Glycan Cleavage Kit, reduced by adding 20 equiv of TCEP (30 min at 25 ºC) and analysed by LC–MS.

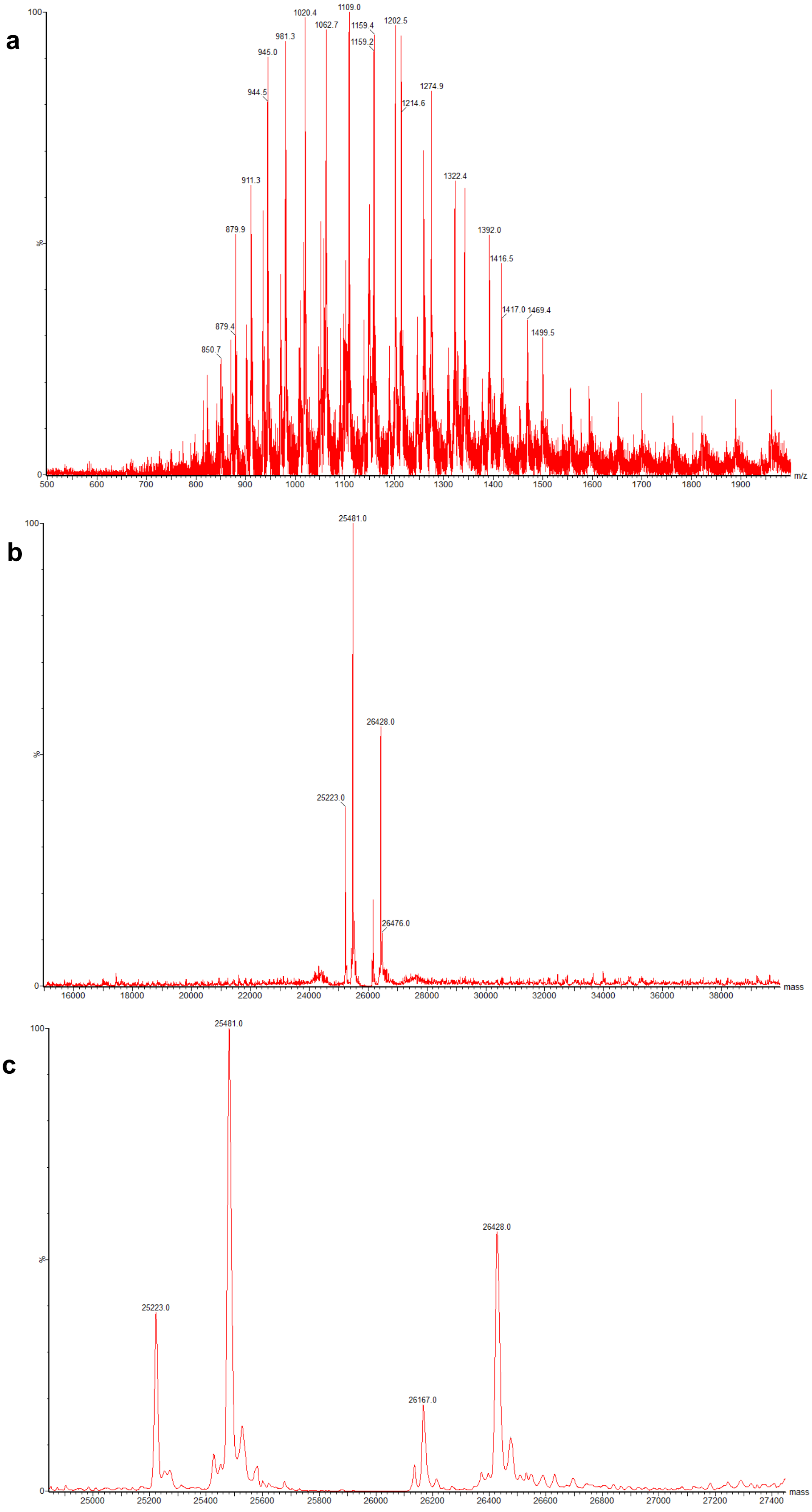

Figure S58. LC–MS spectra of Fc 274C-**14**; **a**) ion series, **b**) deconvoluted spectrum and **c**) highlighted deconvoluted area around modification.

289C

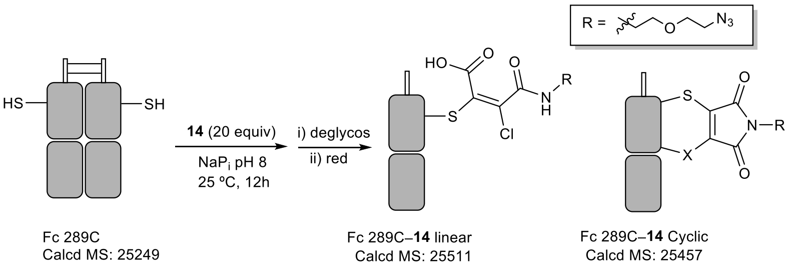

11.5 µL of a stock solution of Fc fragment 289C (87 µM) was added to an eppendorf containing 40.5 µL of NaP_i buffer (pH 8.0, 50 mM). After this, 2 µL of a solution of **14** (10 mM, 20 equiv, 10 equiv for each cysteine) is added and the mixture is then shaken for 12 h at 25 ºC. Then, a 10 µL aliquot of the reaction mixture is deglycosylated using a PNGase F Glycan Cleavage Kit, reduced by adding 20 equiv of TCEP (30 min at 25 ºC) and analysed by LC–MS.

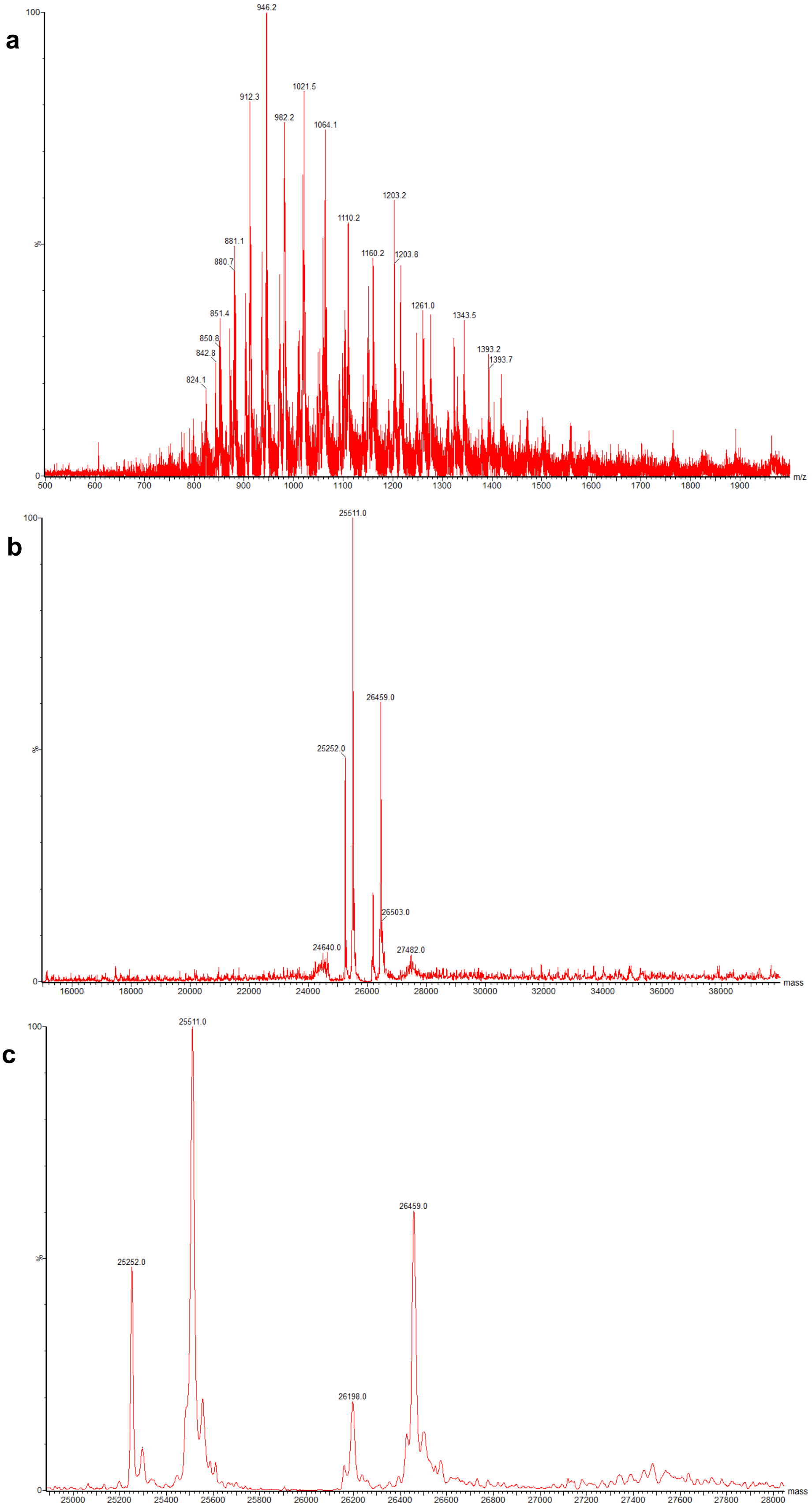

Figure S59. LC–MS spectra of Fc 289C-**14**; **a**) ion series, **b**) deconvoluted spectrum and **c**) highlighted deconvoluted area around modification.

442C

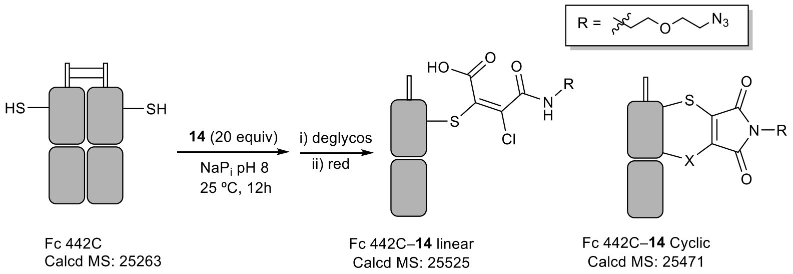

7.9 µL of a stock solution of Fc fragment 442C (126 µM) was added to an eppendorf containing 42.1 µL of NaP_i buffer (pH 8.0, 50 mM). After this, 2 µL of a solution of **14** (10 mM, 20 equiv, 10 equiv for each cysteine) is added and the mixture is then shaken for 12 h at 25 ºC. Then, a 10 µL aliquot of the reaction mixture is deglycosylated using a PNGase F Glycan Cleavage Kit, reduced by adding 20 equiv of TCEP (30 min at 25 ºC) and analysed by LC–MS.

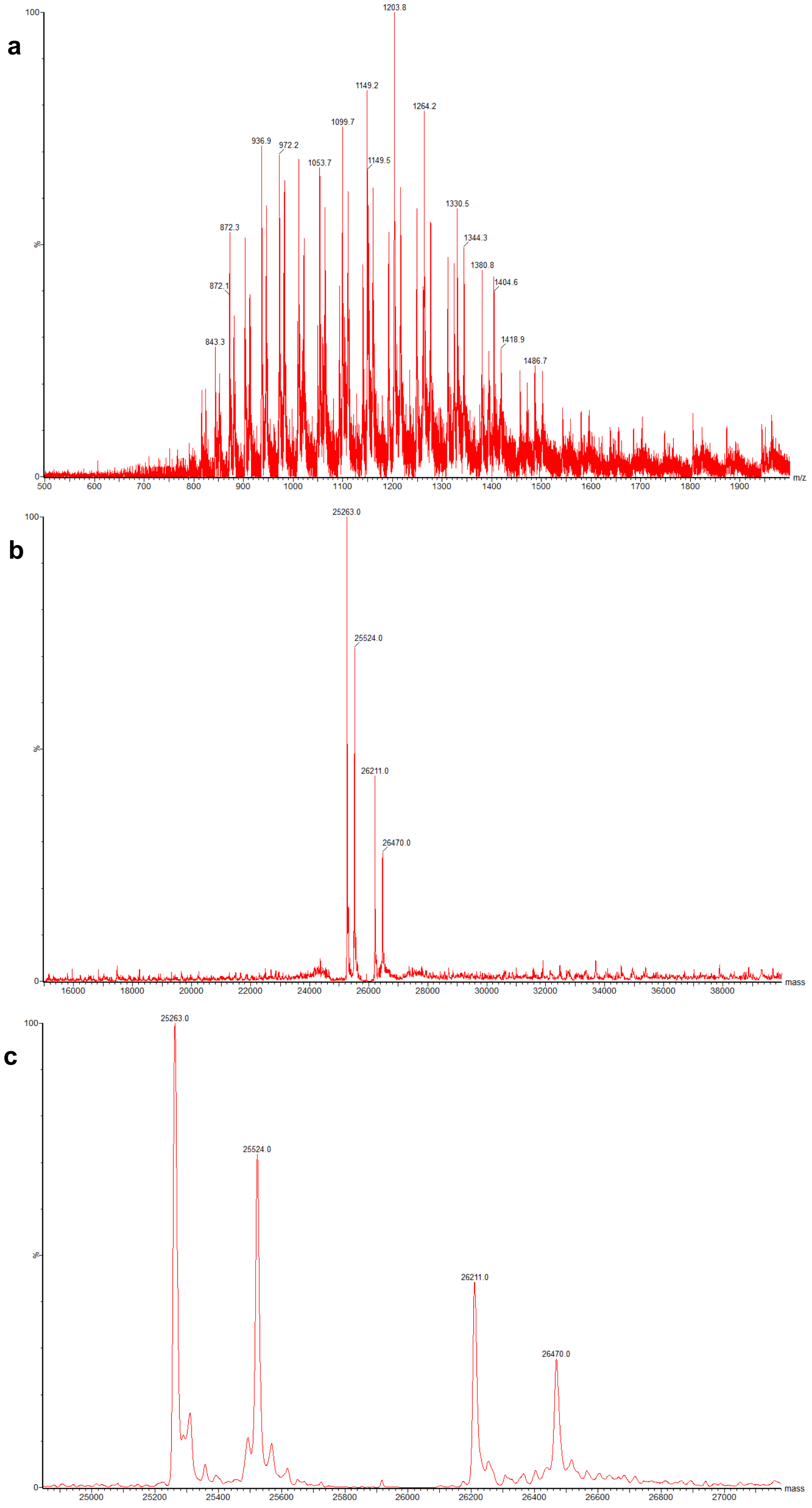

Figure S60. LC–MS spectra of Fc 442C-**14**; **a**) ion series, **b**) deconvoluted spectrum and **c**) highlighted deconvoluted area around modification.

Cys mutation	Conversion	Linear:cyclic
239iC	10	1:1
268C	70	3:1
274C	80	99:1
289C	70	99:1
442C	40	99:1

Table S3. Summary of conversions and selectivity of the reaction of different Fc fragments with **14**.

Conjugation Cys Containing Proteins with Compound 11

Conjugation Reaction Between Trastuzumab V205C and **11**

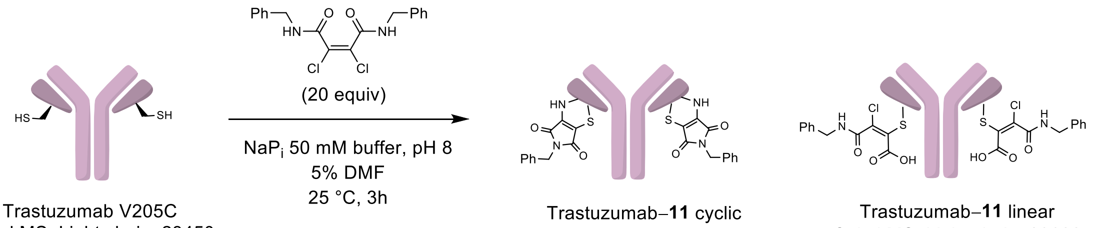

Trastuzumab V205C
Calcd MS: Light chain: 23450
Heavy chain: 50606.

Trastuzumab–**11** cyclic
Calcd MS: Light chain: 23635
Heavy chain: 50606.

Trastuzumab–**11** linear
Calcd MS: Light chain: 23688
Heavy chain: 50606.

18 μL of a stock solution of Trastuzumab V205C (55 μM) was added to an eppendorf containing 32 μL of NaP$_i$ buffer (pH 8.0, 50 mM). After this 2 μL of a solution of **11** (10 mM, 20 equiv, 10 equiv for each cysteine) is added and the mixture is then shaken for 3 h at 25 ºC. After this time, a 10 μL aliquot of the reaction mixture was reduced with TCEP (20 equiv, 25 ºC, 30 min) and analysed by LC–MS.

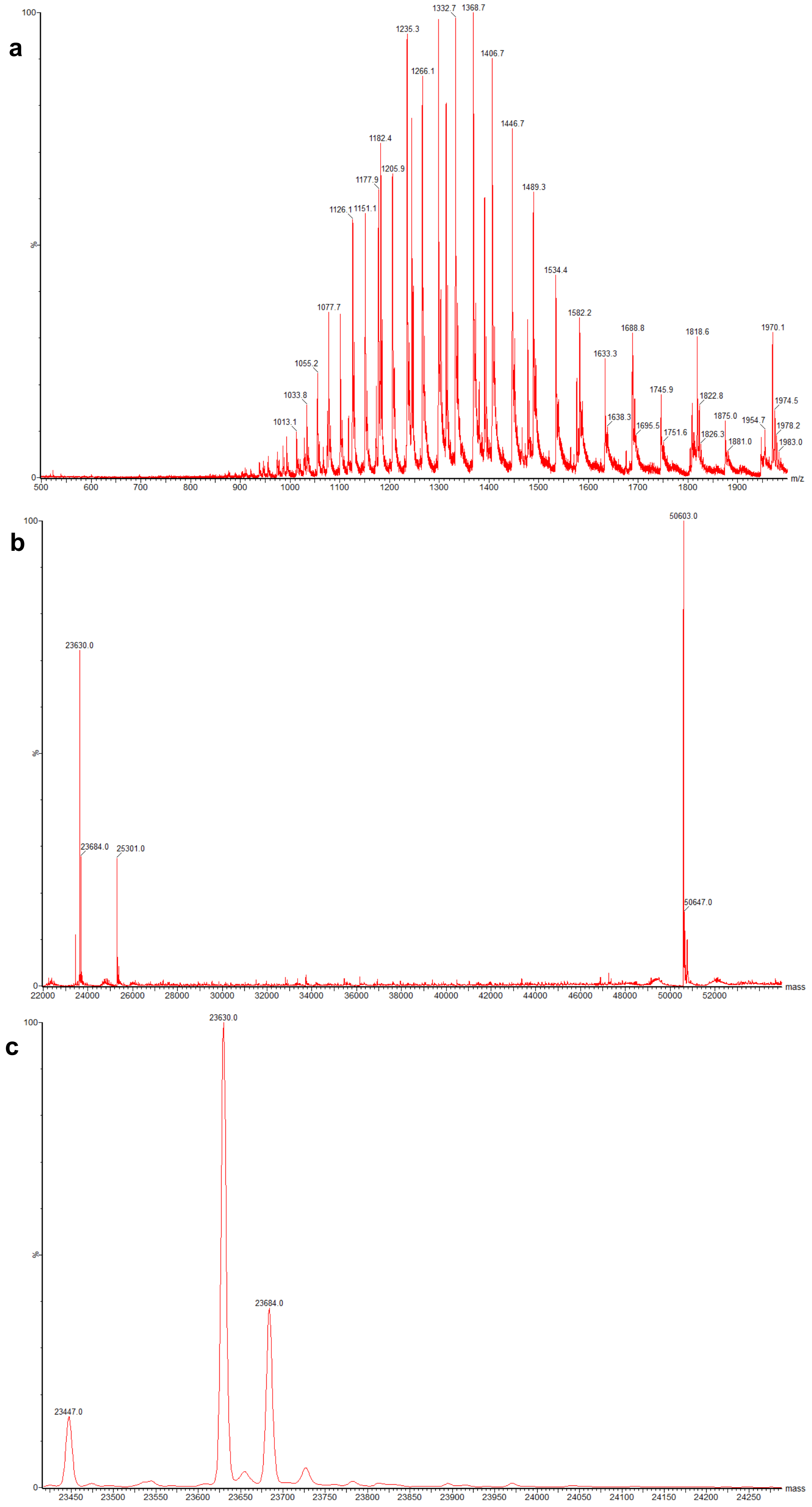

Figure S61. LC–MS spectra of Trastuzumab-**11**; **a**) ion series, **b**) deconvoluted spectrum and **c**) highlighted deconvoluted area around modification.

Conjugation Reaction Between Fc 274C Fragment and **11**

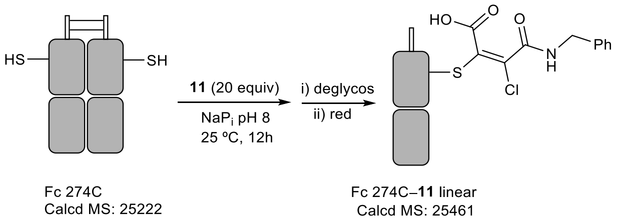

9 µL of a stock solution of Fc fragment 274C (110 µM) was added to an eppendorf containing 41 µL of NaP_i buffer (pH 8.0, 50 mM). After this, 2 µL of a solution of **11** (10 mM, 20 equiv, 10 equiv for each cysteine) is added and the mixture is then shaken for 12 h at 25 ºC. Then, a 10 µL aliquot of the reaction mixture is deglycosylated using a PNGase F Glycan Cleavage Kit, reduced by adding 20 equiv of TCEP (30 min at 25 ºC) and analysed by LC–MS.

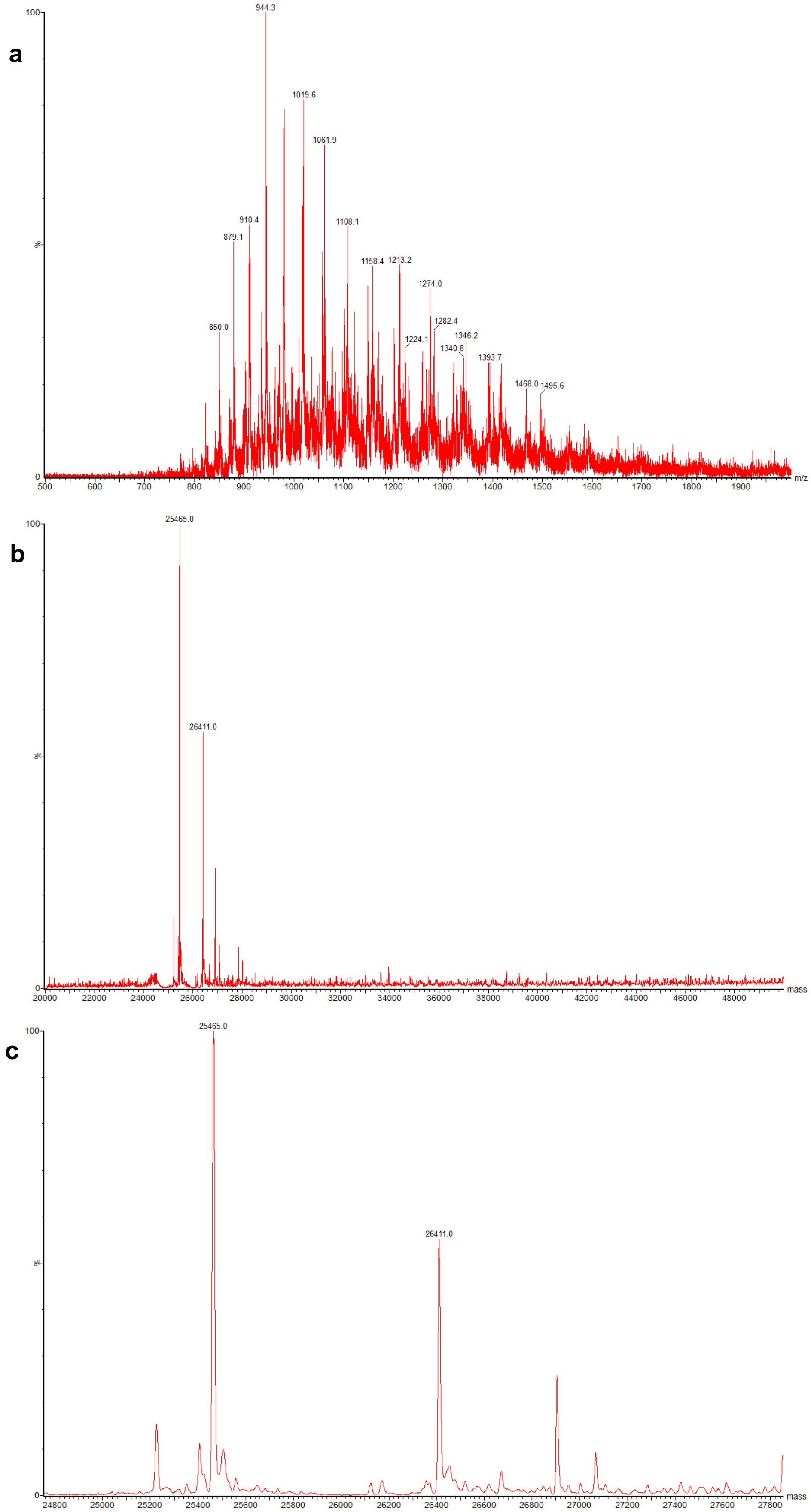

Figure S62. LC–MS spectra of Fc 274C-**11**; **a**) ion series, **b**) deconvoluted spectrum and **c**) highlighted deconvoluted area around modification.

Conjugation Reaction Between Fc 289C Fragments and **11**

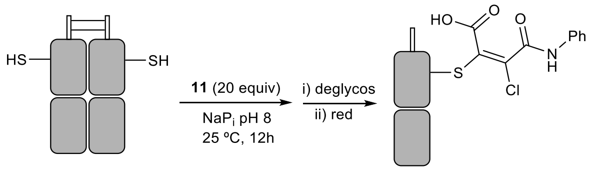

11.5 µL of a stock solution of Fc fragment 289C (87 µM) was added to an eppendorf containing 40.5 µL of NaP_i buffer (pH 8.0, 50 mM). After this, 2 µL of a solution of **11** (10 mM, 20 equiv, 10 equiv for each cysteine) is added and the mixture is then shaken for 12 h at 25 ºC. Then, a 10 µL aliquot of the reaction mixture is deglycosylated using a PNGase F Glycan Cleavage Kit, reduced by adding 20 equiv of TCEP (30 min at 25 ºC) and analysed by LC–MS.

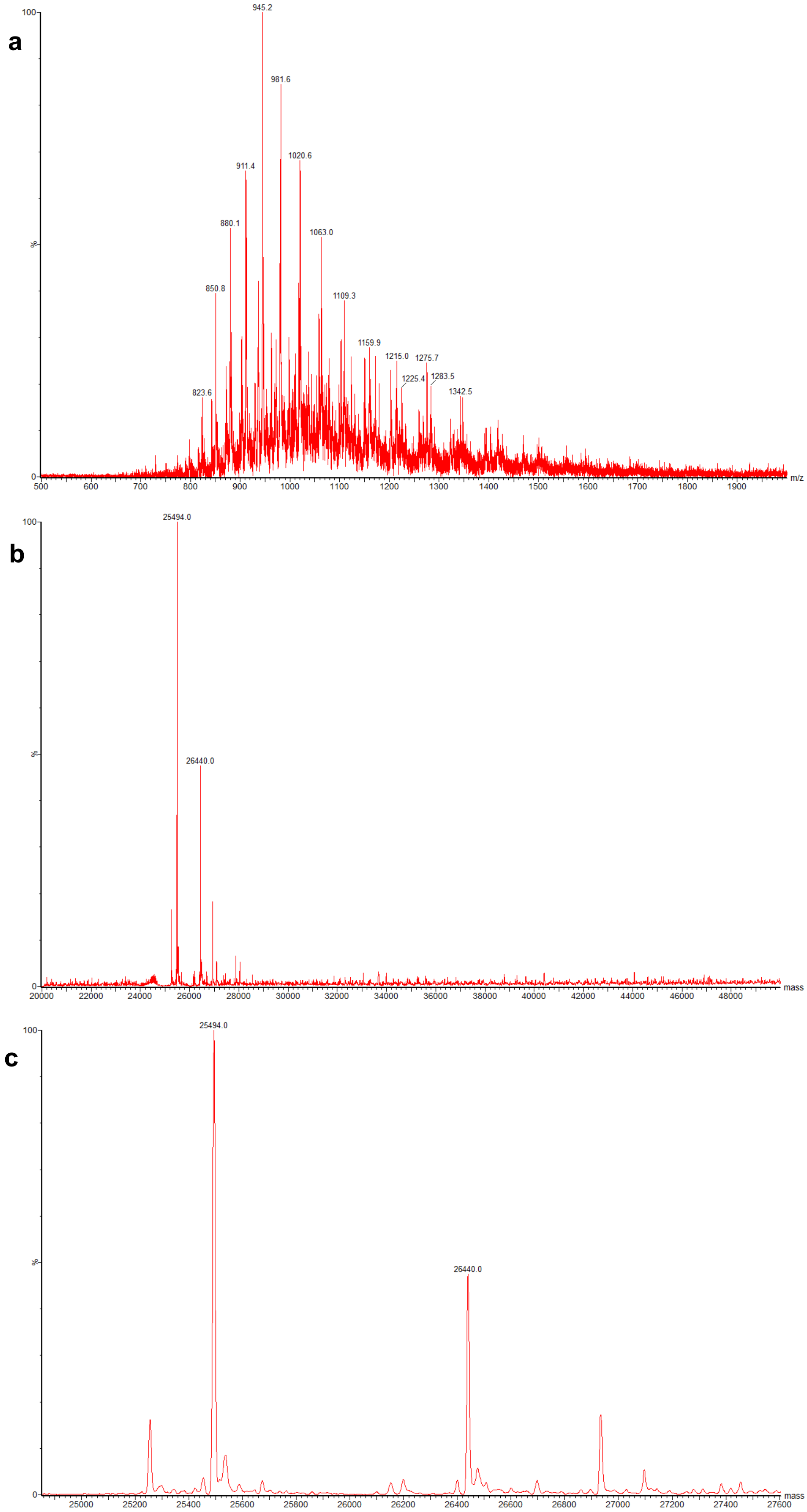

Figure S63. LC–MS spectra of Fc 289C-**11**; **a**) ion series, **b**) deconvoluted spectrum and **c**) highlighted deconvoluted area around modification.

Conjugation of Cys Containing Proteins with Benzyl Maleimide

Conjugation Reaction between Trastuzumab V205C and benzyl maleimide

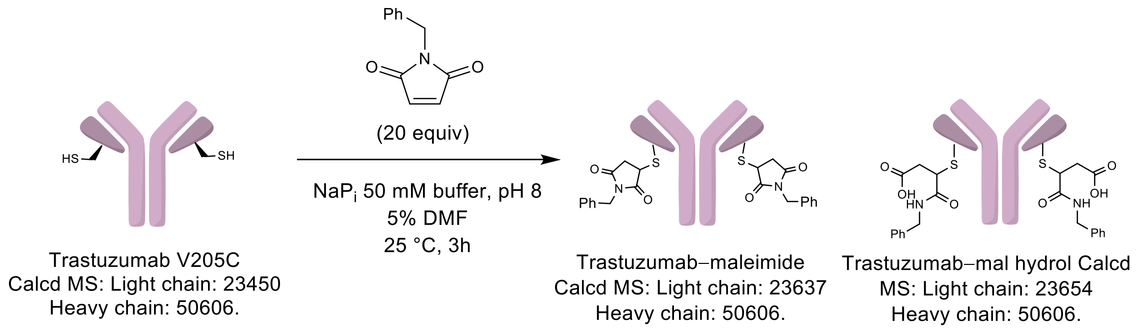

18 µL of a stock solution of Trastuzumab V205C (55 µM) was added to an eppendorf containing 32 µL of NaPi buffer (pH 8.0, 50 mM). After this 2 µL of a solution of benzyl maleimide (10 mM, 20 equiv, 10 equiv for each cysteine) is added and the mixture is then shaken for 3 h at 25 ºC. After this time, a 10 µL aliquot of the reaction mixture was reduced with TCEP (20 equiv, 25 ºC, 30 min) and analysed by LC–MS.

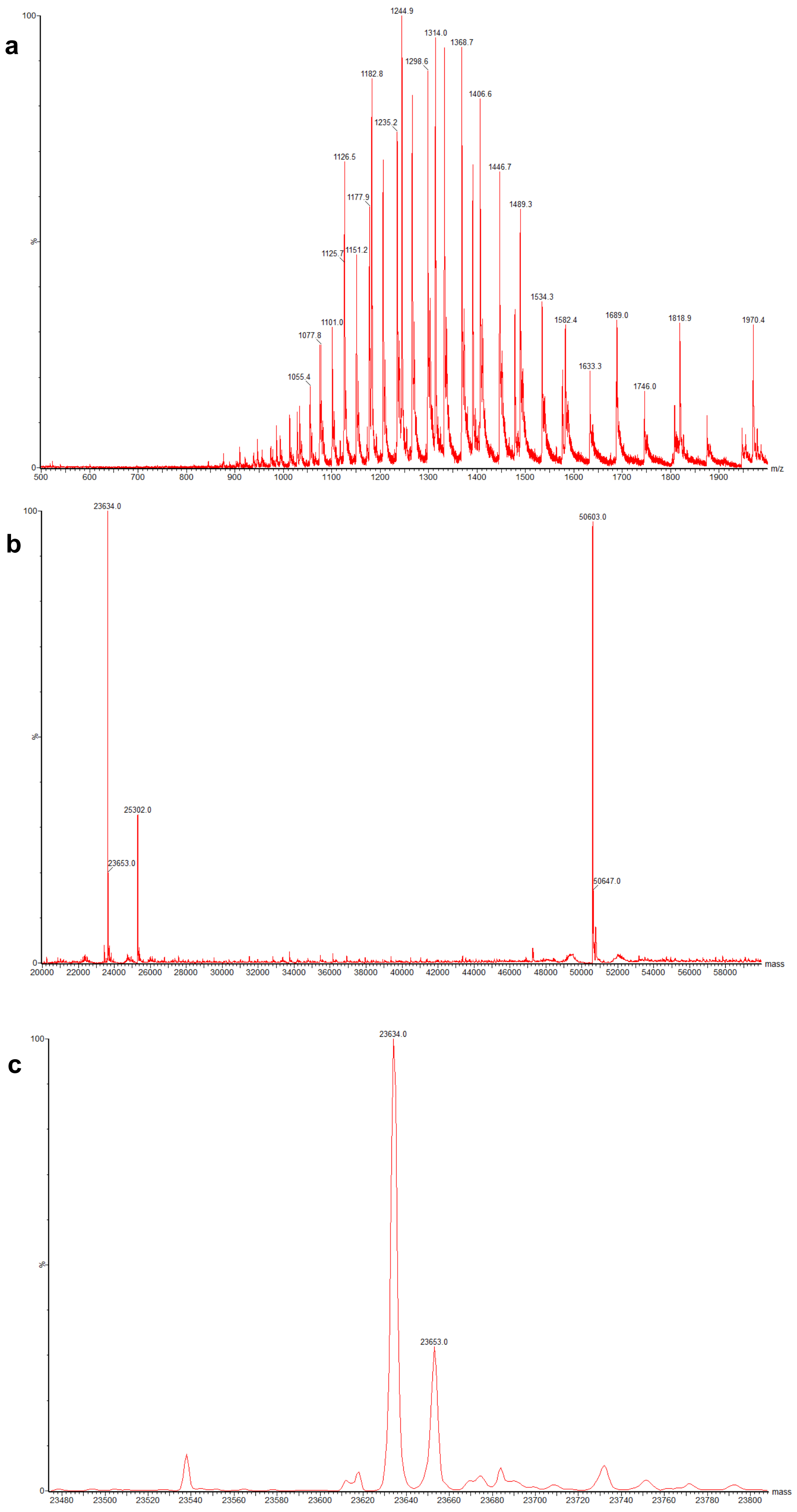

Figure S64. LC–MS spectra of Trastuzumab-maleimide; **a**) ion series, **b**) deconvoluted spectrum and **c**) highlighted deconvoluted area around modification.

Conjugation Reaction Between Fc 274C Fragments and Benzyl Maleimide

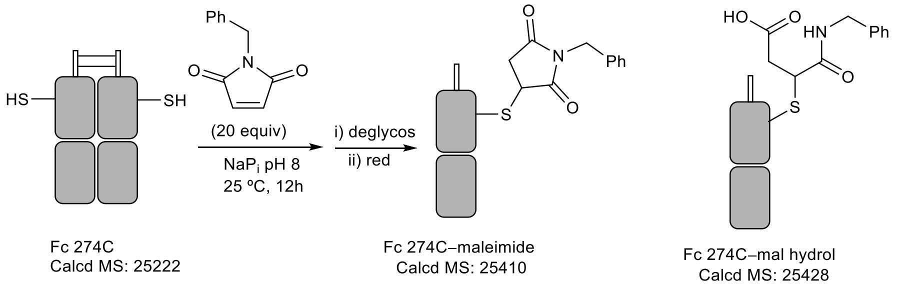

9 μL of a stock solution of Fc fragment 274C (110 μM) was added to an eppendorf containing 41 μL of NaP_i buffer (pH 8.0, 50 mM). After this, 2 μL of a solution of benzyl maleimide (10 mM, 20 equiv, 10 equiv for each cysteine) is added and the mixture is then shaken for 12 h at 25 ºC. Then, a 10 μL aliquot of the reaction mixture is deglycosylated using a PNGase F Glycan Cleavage Kit, reduced by adding 20 equiv of TCEP (30 min at 25 ºC) and analysed by LC–MS.

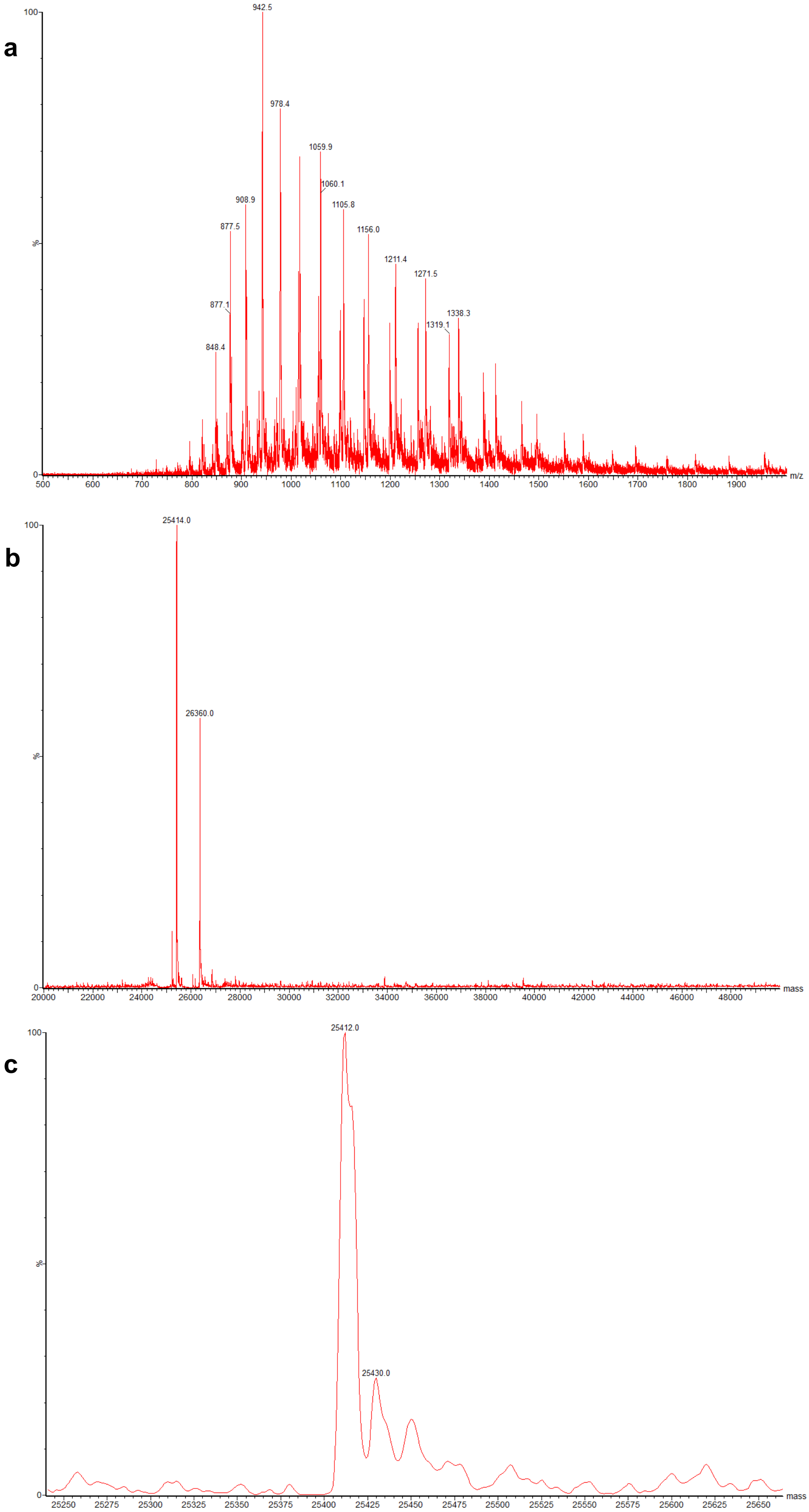

Figure S65. LC–MS spectra of Fc 274C-maleimide; **a**) ion series, **b**) deconvoluted spectrum and **c**) highlighted deconvoluted area around modification.

Conjugation Reaction Between Fc 289C Fragments and Benzyl Maleimide

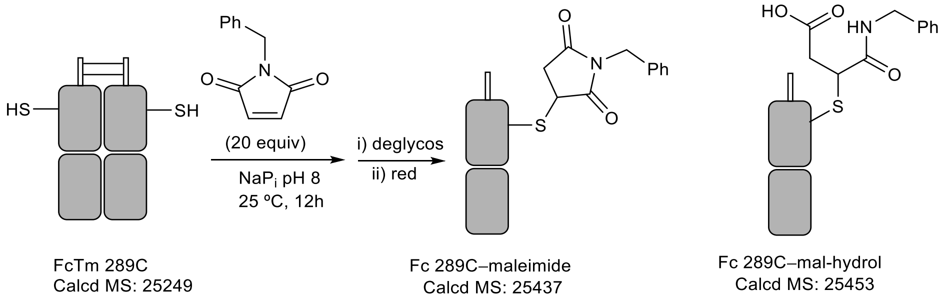

11.5 µL of a stock solution of Fc fragment 289C (87 µM) was added to an eppendorf containing 40.5 µL of NaP$_i$ buffer (pH 8.0, 50 mM). After this, 2 µL of a solution of benzyl maleimide (10 mM, 20 equiv, 10 equiv for each cysteine) is added and the mixture is then shaken for 12 h at 25 ºC. Then, a 10 µL aliquot of the reaction mixture is deglycosylated using a PNGase F Glycan Cleavage Kit, reduced by adding 20 equiv of TCEP (30 min at 25 ºC) and analysed by LC–MS.

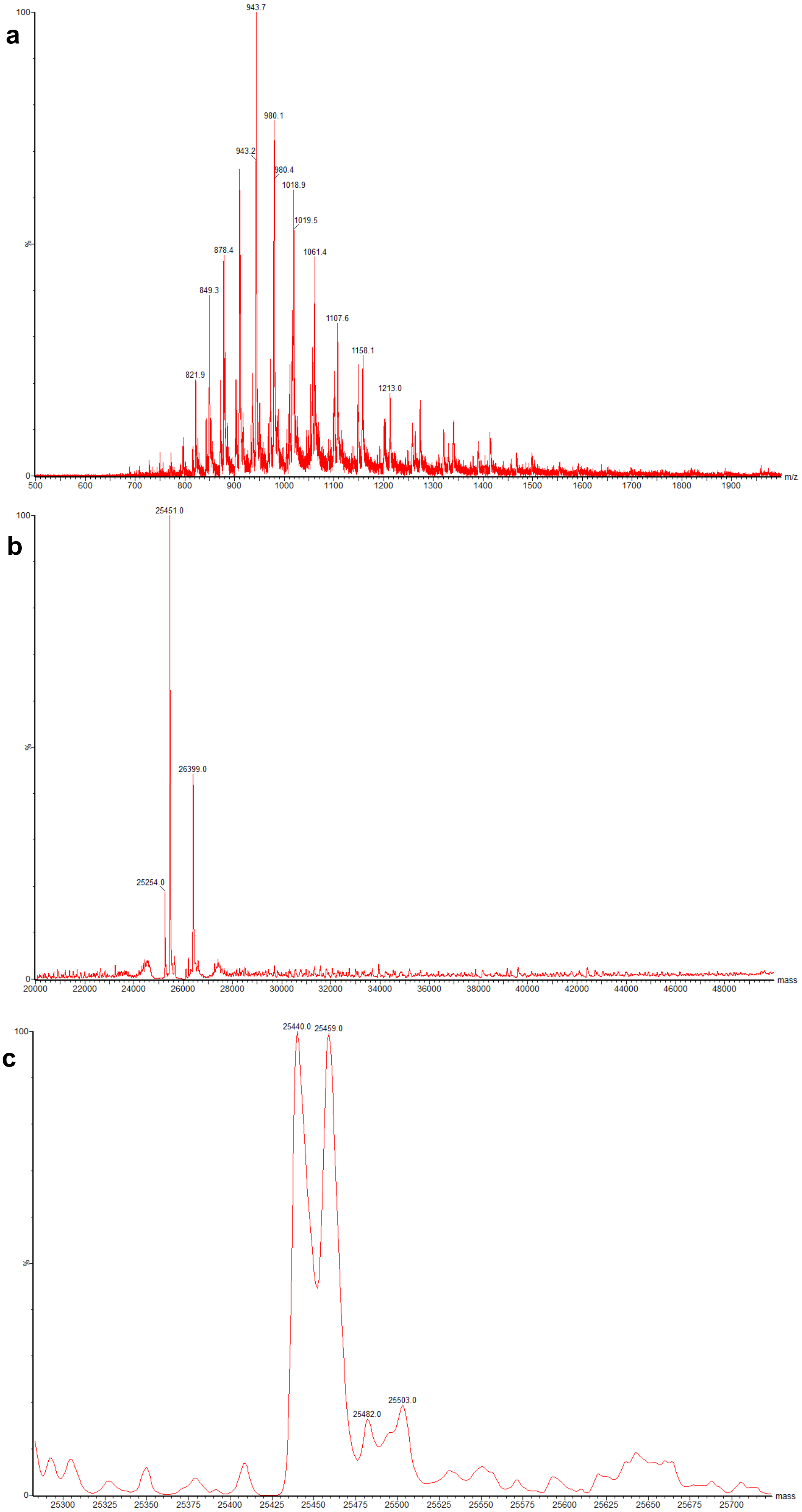

Figure S66. LC–MS spectra of Fc 289C-maleimide; **a**) ion series, **b**) deconvoluted spectrum and **c**) highlighted deconvoluted area around modification.

Reaction Between Ub and 11 at Different pH

Conjugation Reaction Between Ub and **11** at pH 5

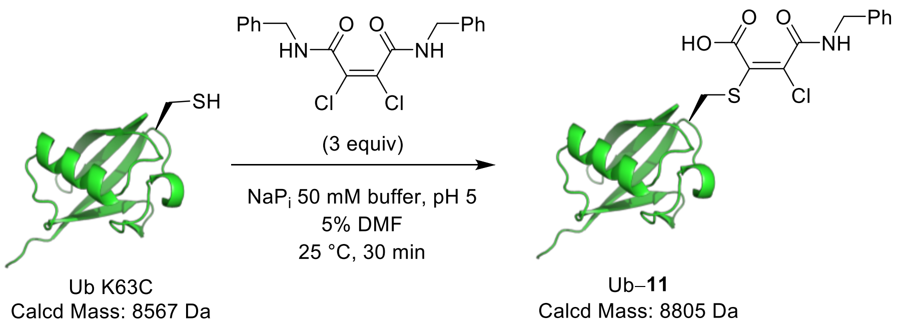

4.4 µL of a stock solution of Ub–K63C (90 µM) was added to an eppendorf containing 15.6 µL of NaOAc buffer (pH 5.0, 50 mM). The resulting mixture was vortexed, and afterwards 1 mM solution of **11** (1.2 µL, 3 equiv) in DMF was added. The reaction mixture was then shaken for 30 min at 25 ºC. After this time, a 10 µL aliquot of the reaction mixture was analysed by LC–MS.

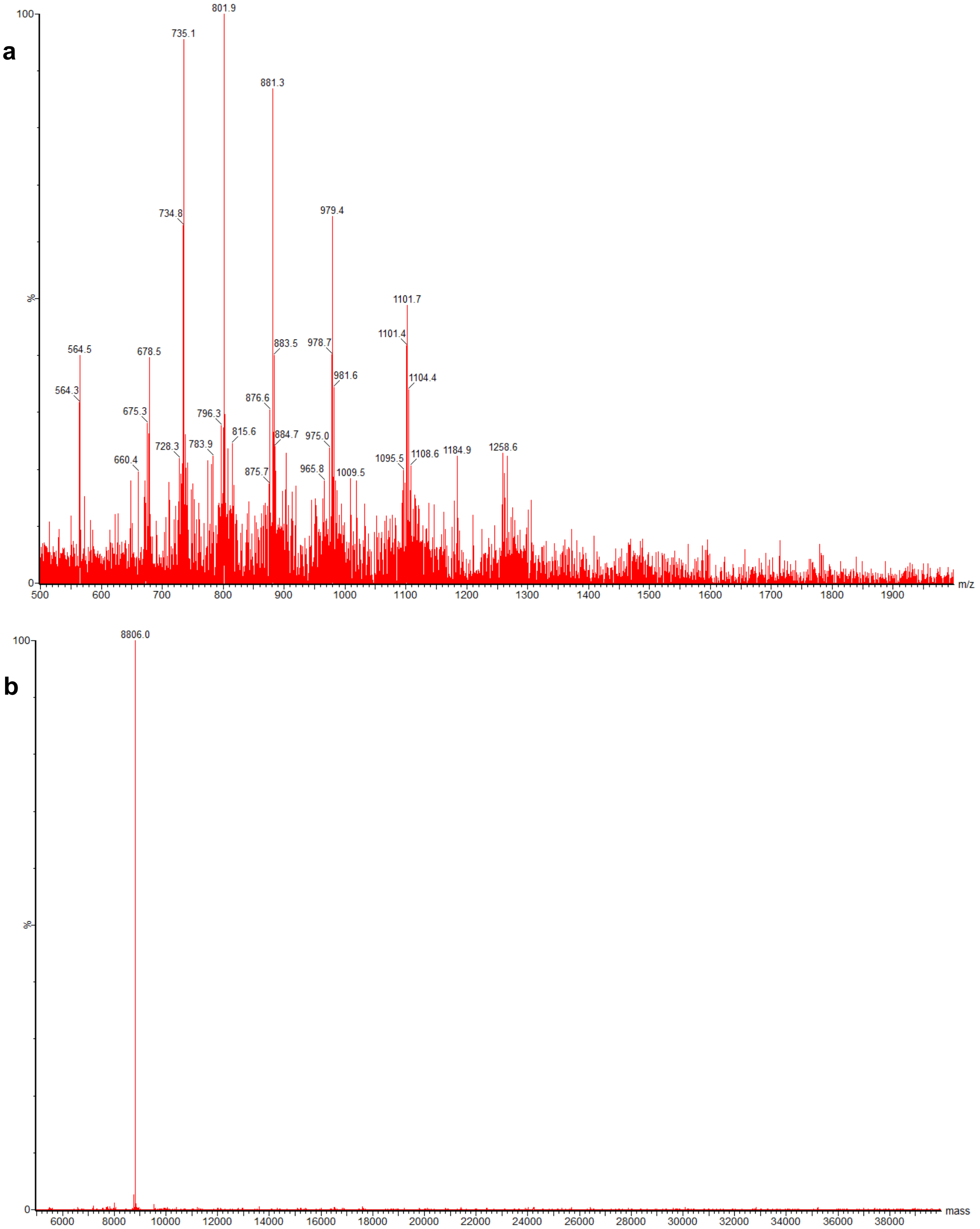

Figure S67. LC–MS spectra of Ub-**11** formed at pH 5; **a**) ion series and **b**) deconvoluted spectrum.

Conjugation Reaction Between Ub and **11** at pH 6

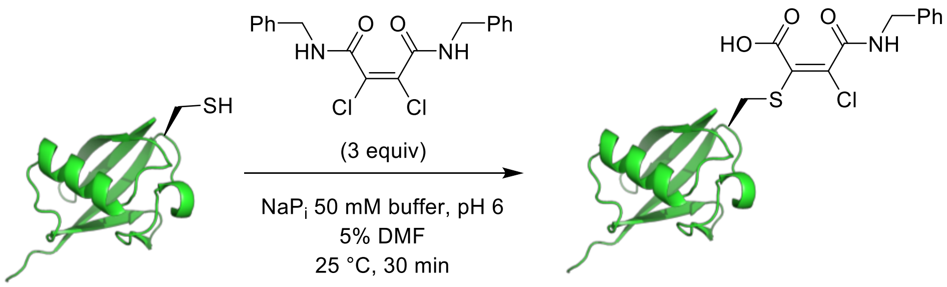

Ub K63C
Calcd Mass: 8567 Da

Ub–**11**
Calcd Mass: 8805 Da

4.4 μL of a stock solution of Ub–K63C (90 μM) was added to an eppendorf containing 15.6 μL of NaOAc buffer (pH 6.0, 50 mM). The resulting mixture was vortexed, and afterwards 1 mM solution of **11** (1.2 μL, 3 equiv) in DMF was added. The reaction mixture was then shaken for 30 min at 25 ºC. After this time, a 10 μL aliquot of the reaction mixture was analysed by LC–MS.

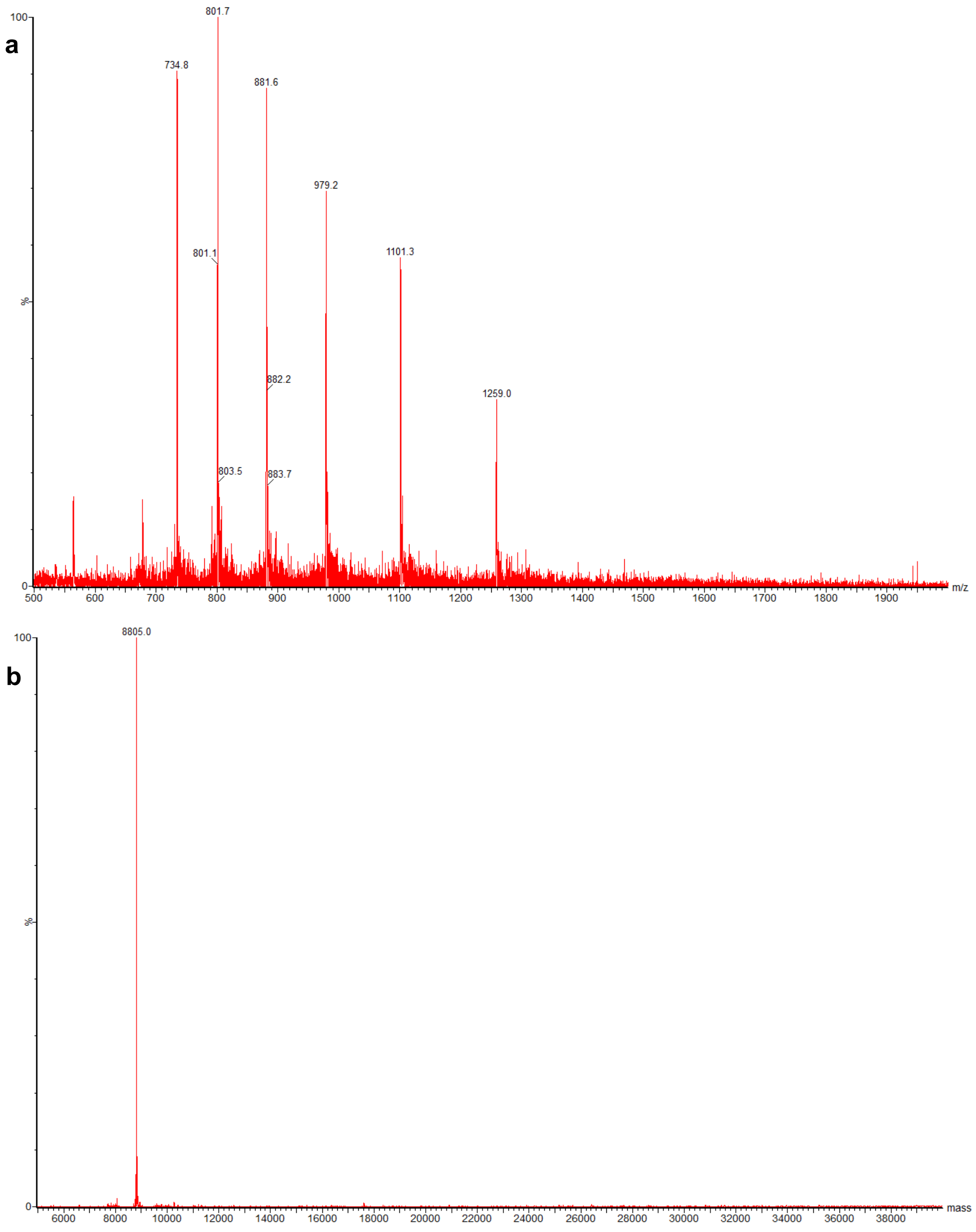

Figure S68. LC–MS spectra of Ub-**11** formed at pH 6; **a**) ion series and **b**) deconvoluted spectrum.

Conjugation Reaction Between Ub and **11** at pH 7

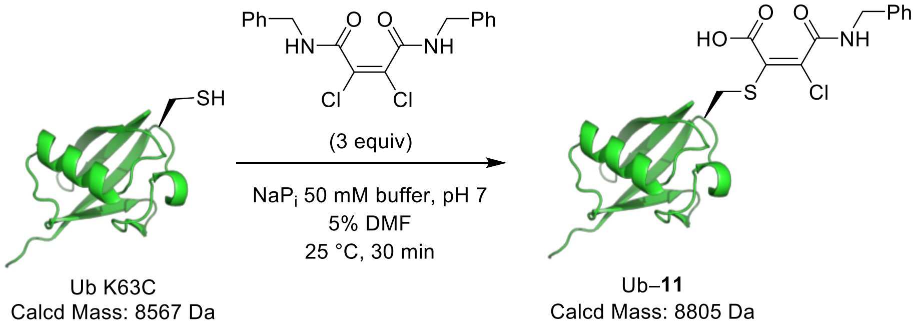

4.4 µL of a stock solution of Ub–K63C (90 µM) was added to an eppendorf containing 15.6 µL of NaP$_i$ buffer (pH 7.0, 50 mM). The resulting mixture was vortexed, and afterwards 1 mM solution of **11** (1.2 µL, 3 equiv) in DMF was added. The reaction mixture was then shaken for 30 min at 25 ºC. After this time, a 10 µL aliquot of the reaction mixture was analysed by LC–MS.

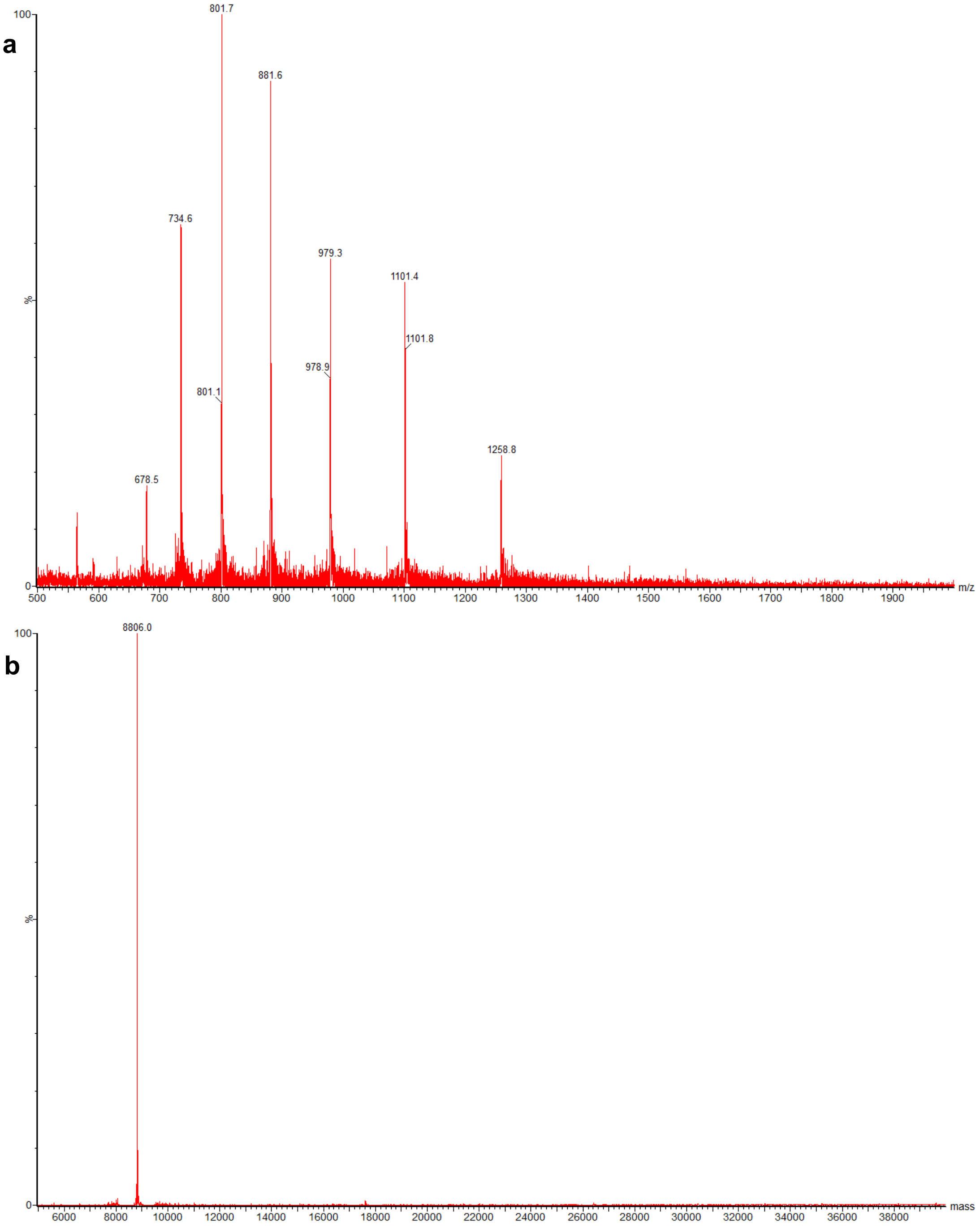

Figure S69. LC–MS spectra of Ub-**11** formed at pH 7; **a**) ion series and **b**) deconvoluted spectrum.

Conjugation reaction between Ub and 11 at pH 9

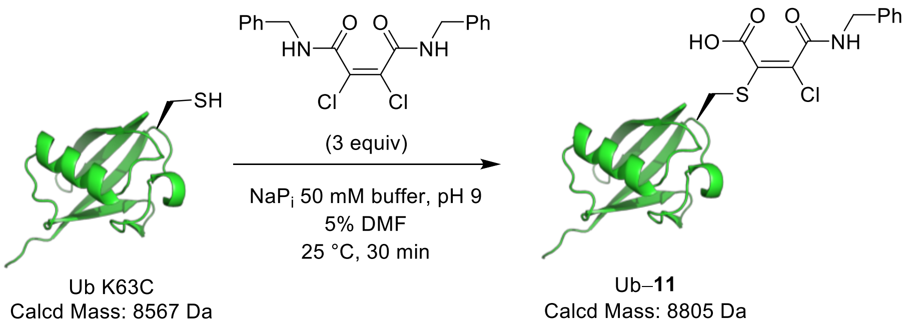

4.4 μL of a stock solution of Ub–K63C (90 μM) was added to an eppendorf containing 15.6 μL of Tris buffer (pH 9.0, 50 mM). The resulting mixture was vortexed, and afterwards 1 mM solution of **11** (1.2 μL, 3 equiv) in DMF was added. The reaction mixture was then shaken for 30 min at 25 ºC. After this time, a 10 μL aliquot of the reaction mixture was analysed by LC–MS.

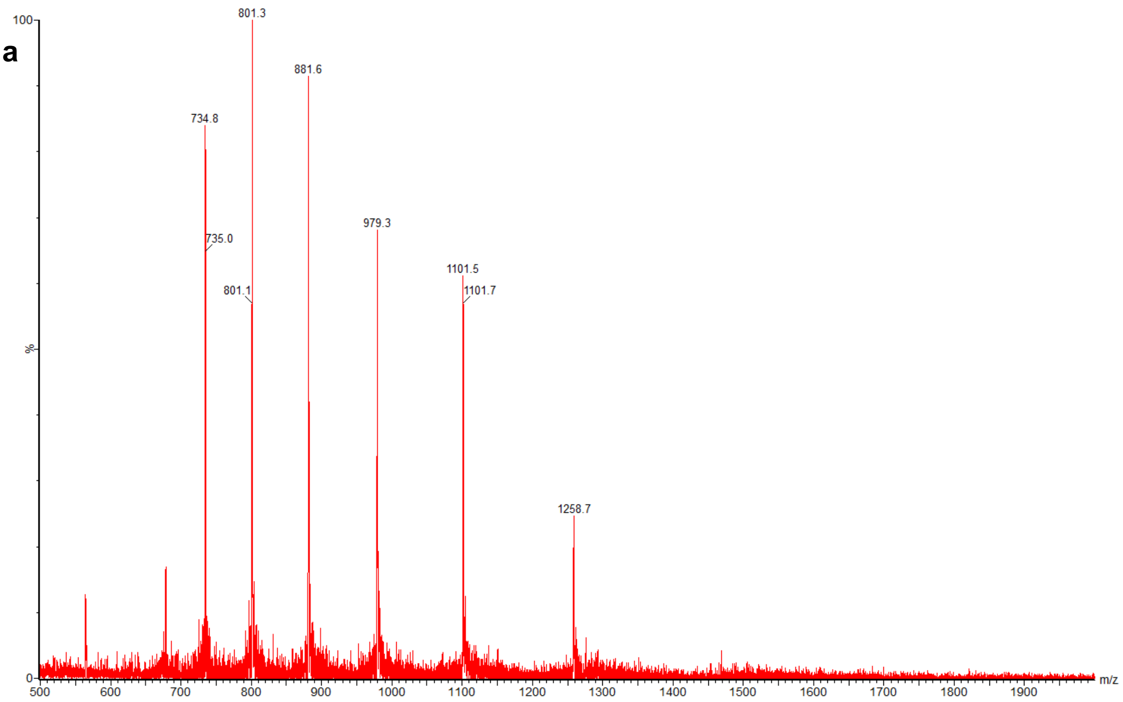

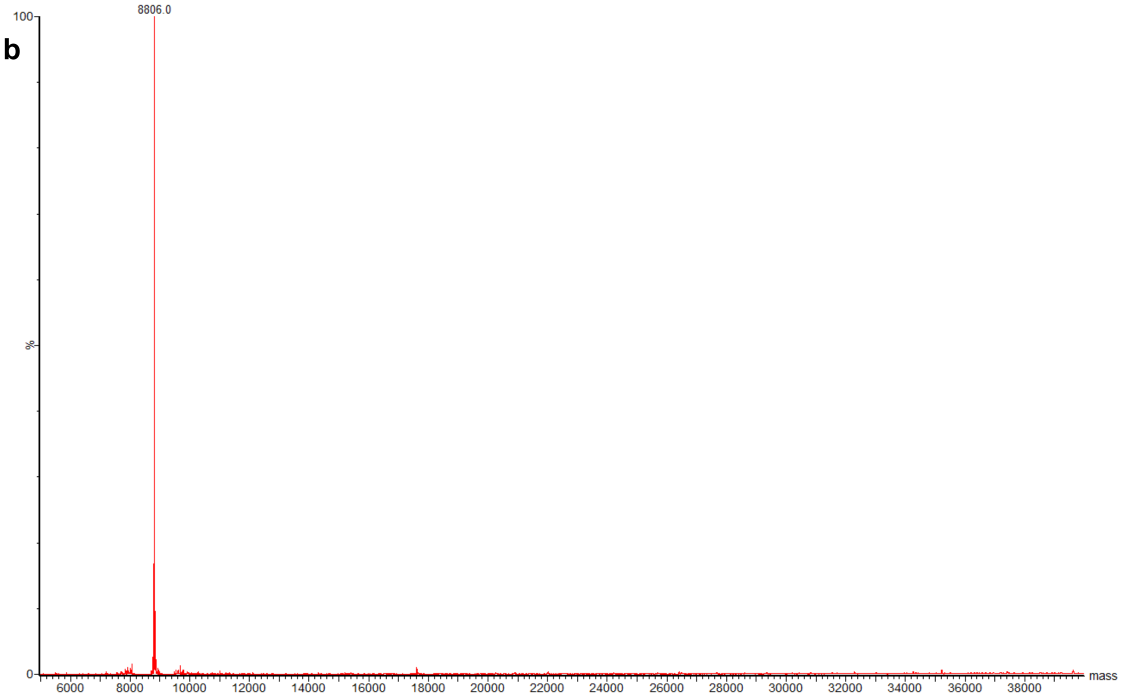

Figure S70. LC–MS spectra of Ub-**11** formed at pH 9; **a**) ion series and **b**) deconvoluted spectrum.

Hydrolysis of Ub-14

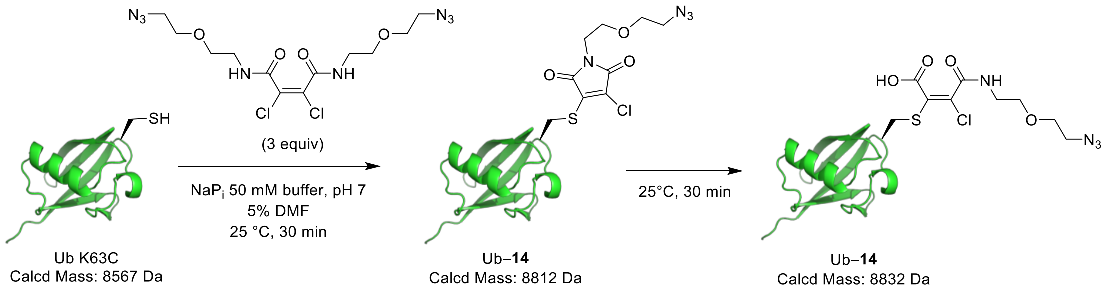

8.8 µL of a stock solution of Ub–K63C (90 µM) was added to an eppendorf containing 31.2 µL of NaP_i buffer (pH 7.0, 50 mM). The resulting mixture was vortexed, and afterwards 1 mM solution of 14 (2.4 µL, 3 equiv) in DMF was added. The reaction mixture was analysed by LC-MS straight after addition and then shaken for 30 min at 25 °C and analysed again. When analysed just after addition, cyclic structure can be observed but after 30 min it has completely hydrolysed to the linear version.

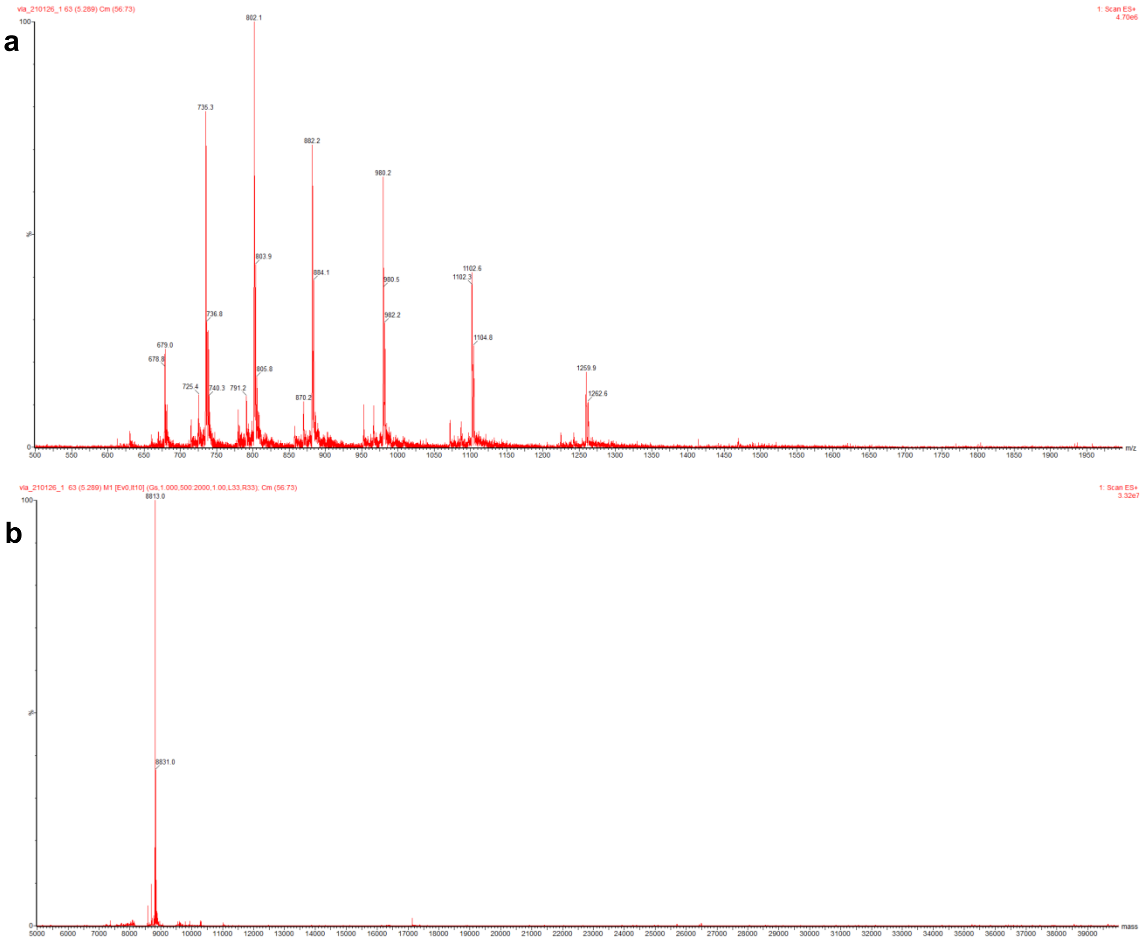

Figure S71. LC–MS spectra of Ub-**14** analysed straight after mixing; **a**) ion series and **b**) deconvoluted spectrum.

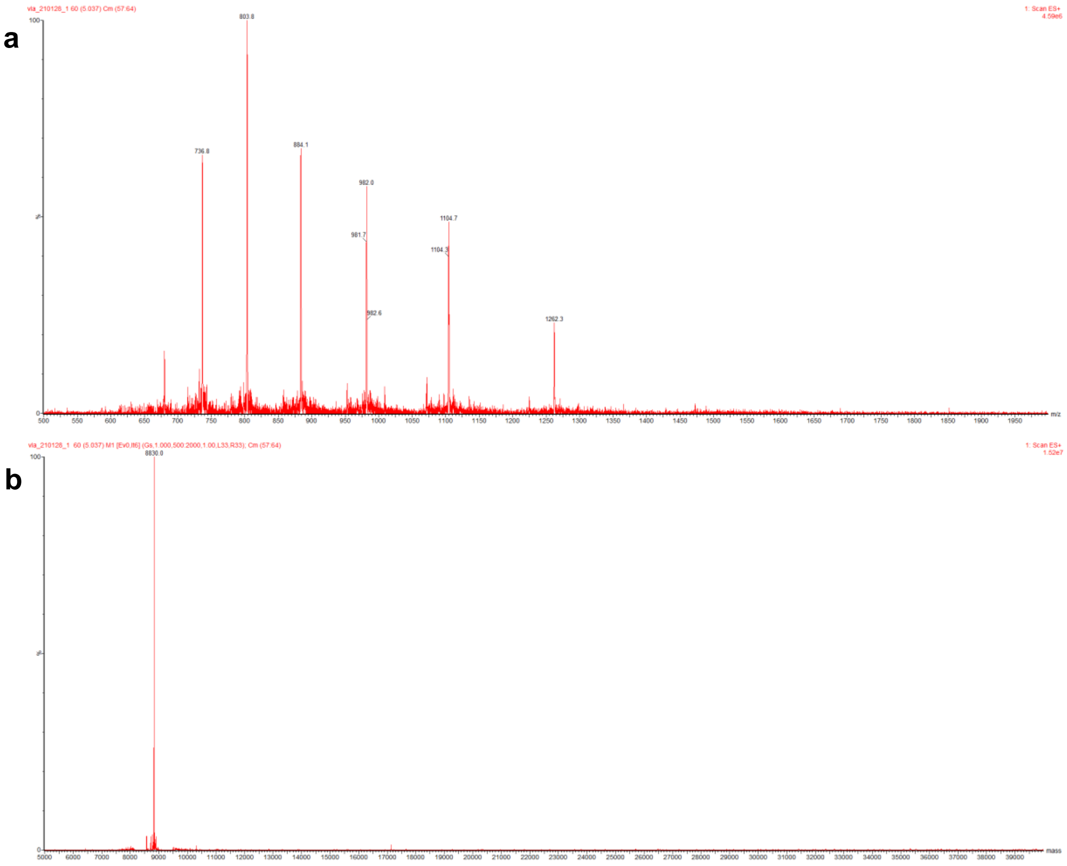

Figure S72. LC–MS spectra of Ub-**14** analysed after 30 min; **a**) ion series and **b**) deconvoluted spectrum.

5. Stability Studies

Stability of Ub-14 in the Presence of GSH (1 mM)

A 50 µL aliquot of the bioconjugate Ub-**14** (10 µM) in NaP_i buffer (20 mM, pH 8.0) was prepared. 2.5 µL of a 20 mM glutathione solution (6 mg glutathione dissolved in 1 µL of TrisHCl buffer (20 mM, pH 8.0)) was added at room temperature and the resulting mixture vortexed for 10 seconds. The resulting reaction mixture was then shaken at 37 ºC. After 1 and 48 h, a 10 µL aliquot of each reaction mixture was analysed by LC–MS.

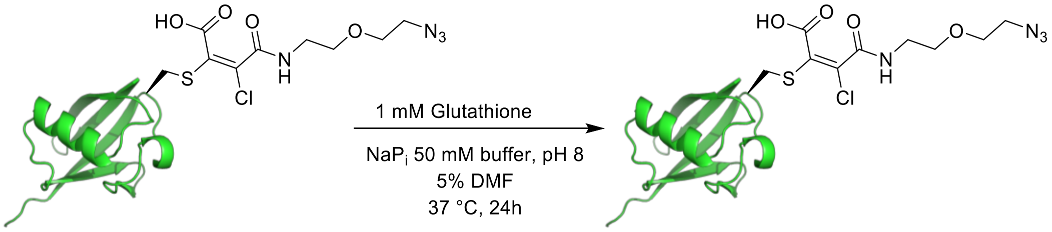

Ub–**14**
Calcd Mass: 8830 Da

Ub–**14**
Calcd Mass: 8830 Da

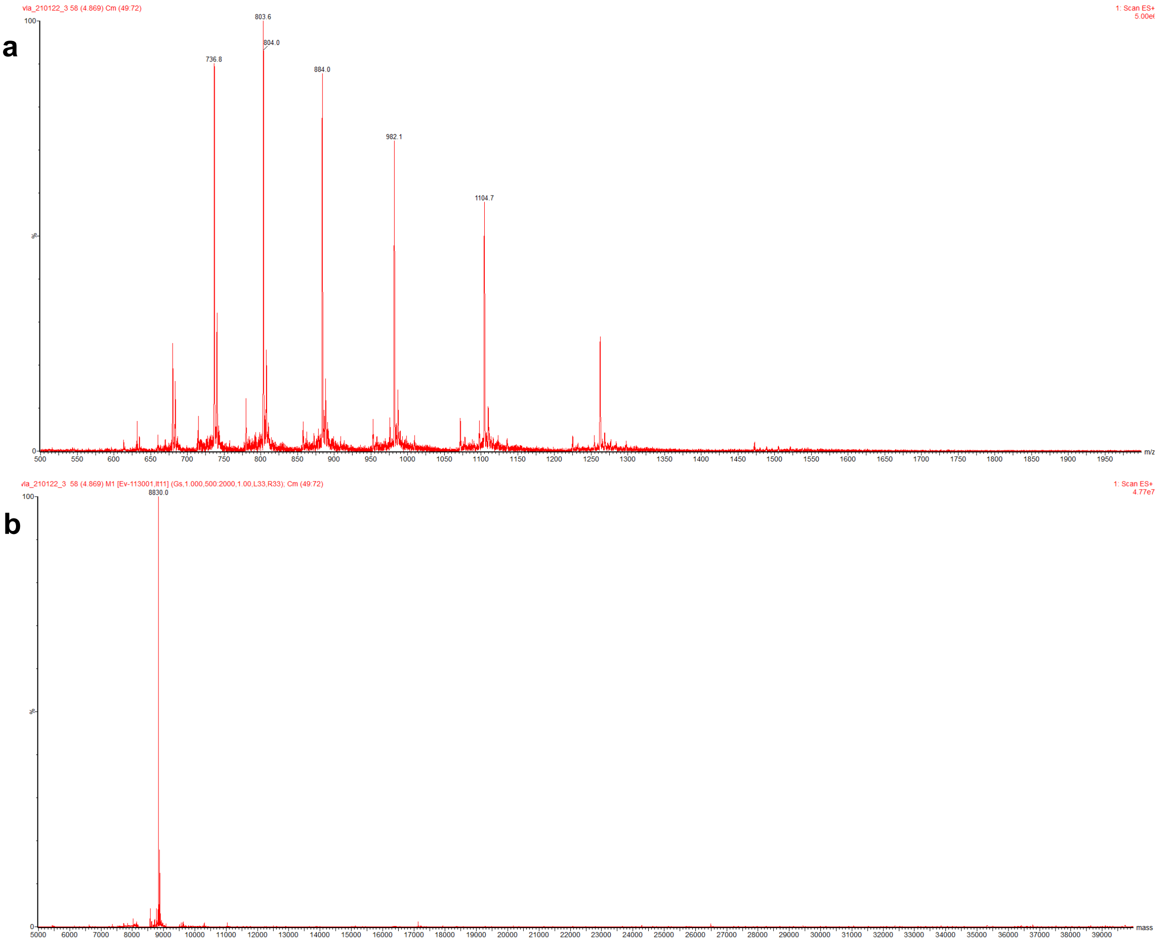

Figure S73. LC–MS spectra of Ub-**14** after 48 h in the presence of GSH; **a**) ion series and **b**) deconvoluted spectrum.

Stability of Ub-14 in human plasma

A 50 µL aliquot of the bioconjugate (10 µM) in NaP$_i$ buffer (20 mM, pH 8.0) was prepared. 2.5 µL of reconstituted human plasma was added at room temperature and the resulting mixture vortexed for 10 seconds. The resulting reaction mixture was then mixed at 37 ºC. After 1 and 48 h, a 10 µL aliquot of each reaction mixture was analysed by LC–MS.

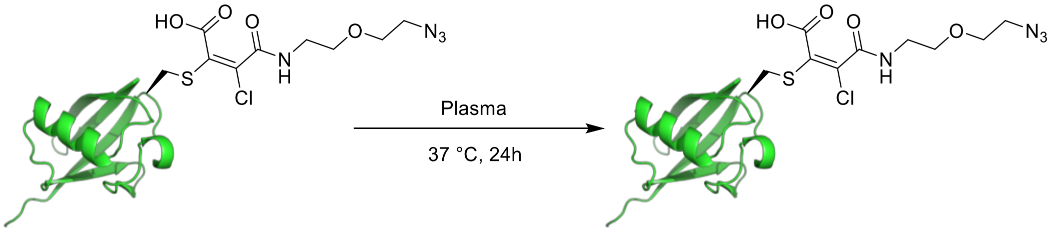

Ub–**14**
Calcd Mass: 8830 Da

Ub–**14**
Calcd Mass: 8830 Da

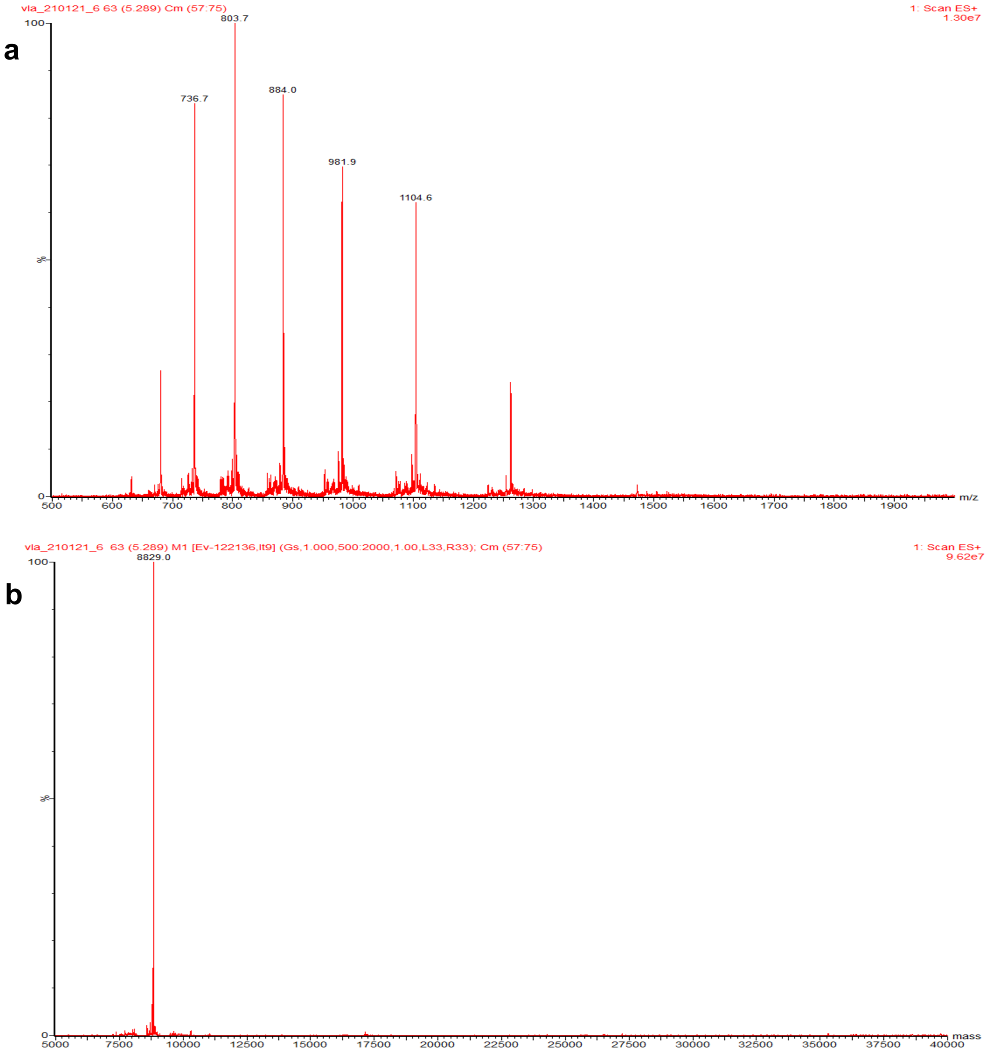

Figure S74. LC–MS spectra of Ub-**14** after 48 h in the presence of plasma; **a**) ion series and **b**) deconvoluted spectrum.

Comparison of Stability Between Trastuzumab-11 and Trastuzumab-maleimide

Stability of Trastuzumab-**11** in the presence of GSH (1 mM)

A 50 µL aliquot of Trastuzumab-**11** (10 µM) in NaP_i buffer (20 mM, pH 8.0) was prepared. 2.5 µL of a 20 mM glutathione solution (6 mg glutathione dissolved in 1 µL of TrisHCl buffer (20 mM, pH 8.0)) was added at room temperature and the resulting mixture vortexed for 10 seconds. The resulting reaction mixture was then shaken at 37 ºC. After 66 h, the protein was reduced by adding 20 equiv of TCEP (stirred for 30 min at 25 ºC) and 10 µL aliquot of reaction mixture was analysed by LC–MS.

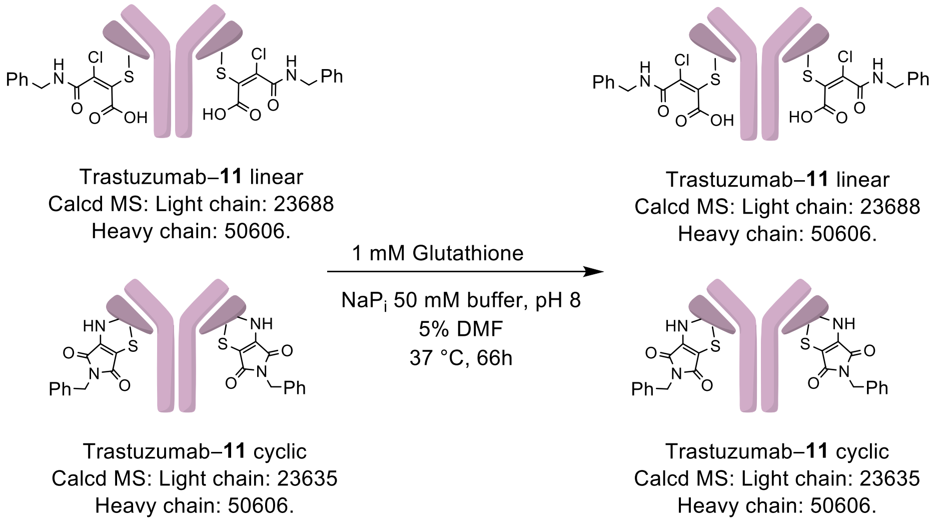

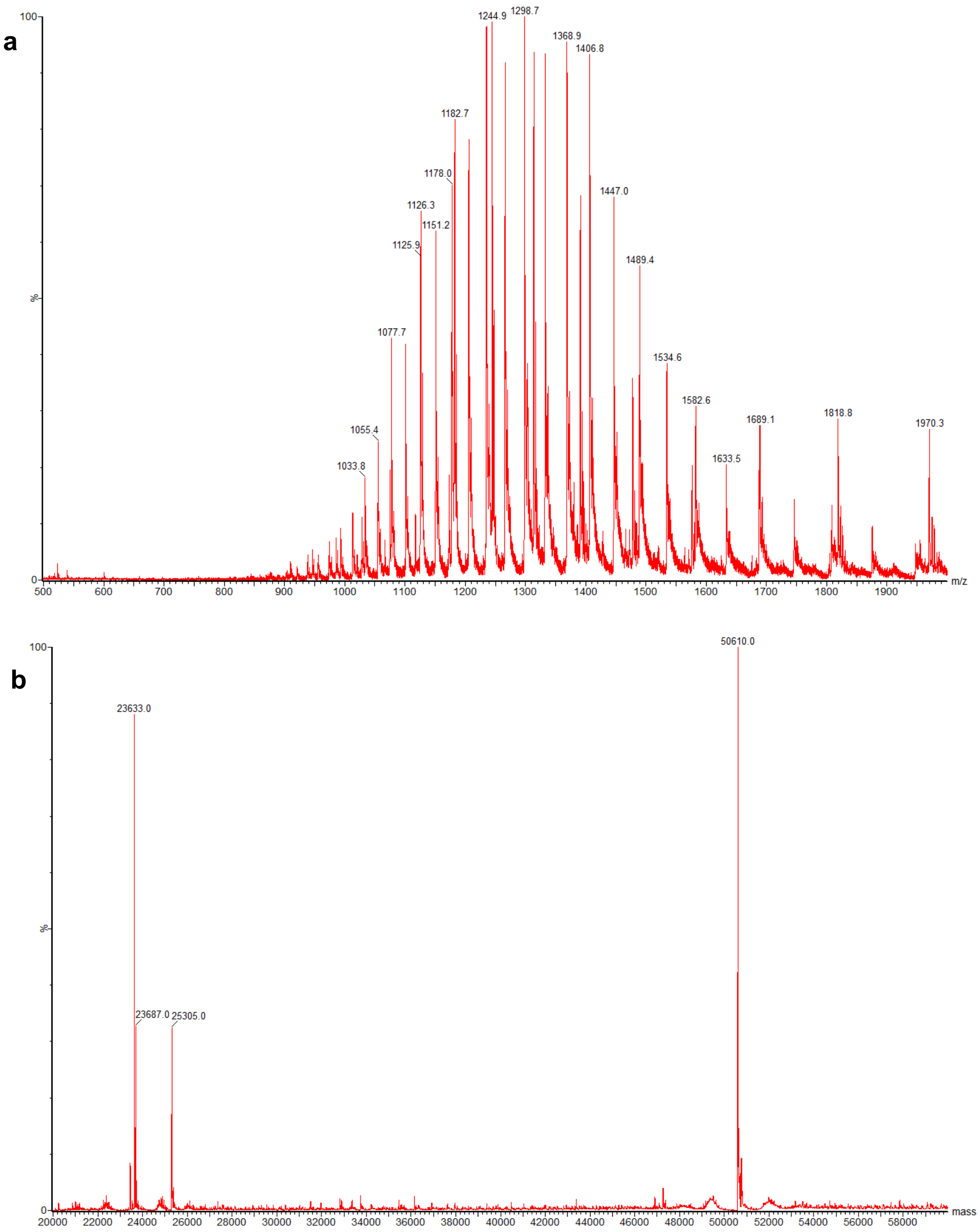

Figure S75. LC–MS spectra of Trastuzumab-**11** after 66 h in the presence of GSH; **a**) ion series and **b**) deconvoluted spectrum.

Stability of Trastuzumab-maleimide in the presence of GSH (1 mM)

A 50 µL aliquot of Trastuzumab-maleimide (10 µM) in NaP_i buffer (20 mM, pH 8.0) was prepared. 2.5 µL of a 20 mM glutathione solution (6 mg glutathione dissolved in 1 µL of TrisHCl buffer (20 mM, pH 8.0)) was added at room temperature and the resulting mixture vortexed for 10 seconds. The resulting reaction mixture was then shaken at 37 ºC. After 66 h, the protein was reduced by adding 20 equiv of TCEP (stirred for 30 min at 25 ºC) and 10 µL aliquot of reaction mixture was analysed by LC–MS.

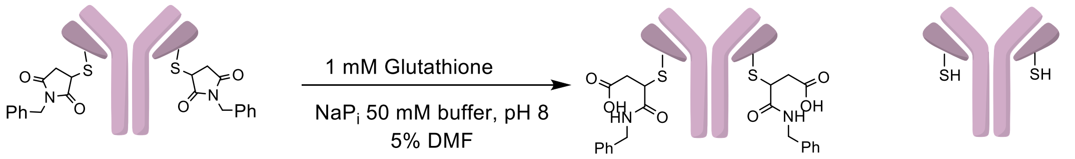

Trastuzumab–maleimide
Calcd MS: Light chain: 23637
Heavy chain: 50606.

Trastuzumab–mal hydrol
Calcd MS: Light chain: 23654
Heavy chain: 50606.

Trastuzumab
Calcd MS: Light chain: 23439
Heavy chain: 50606.

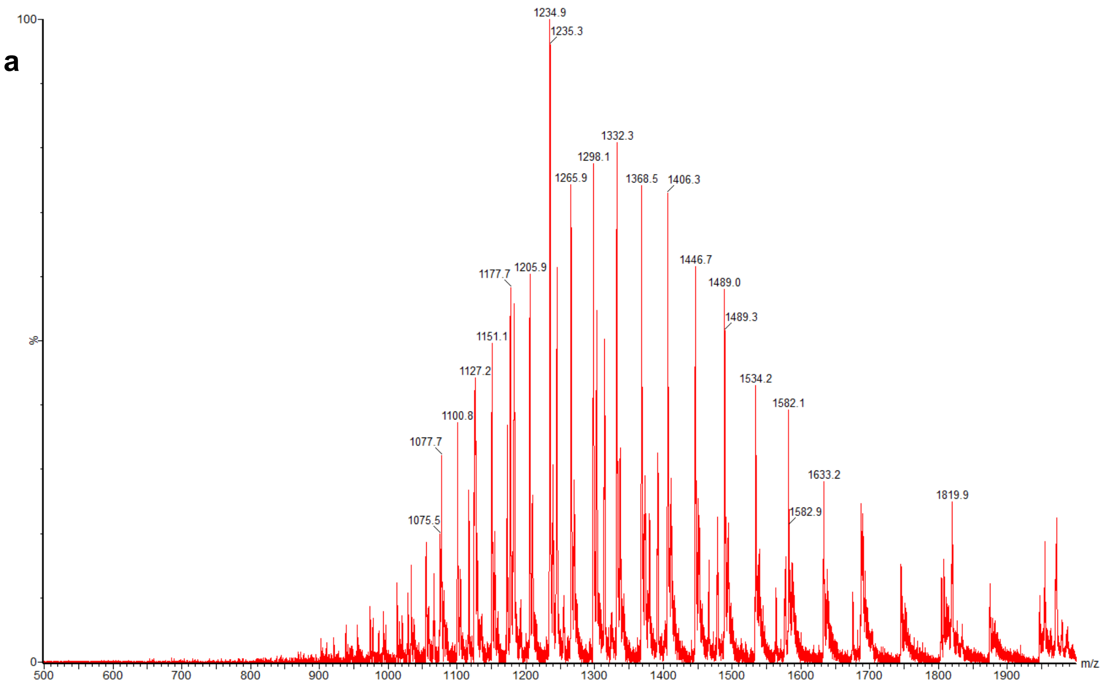

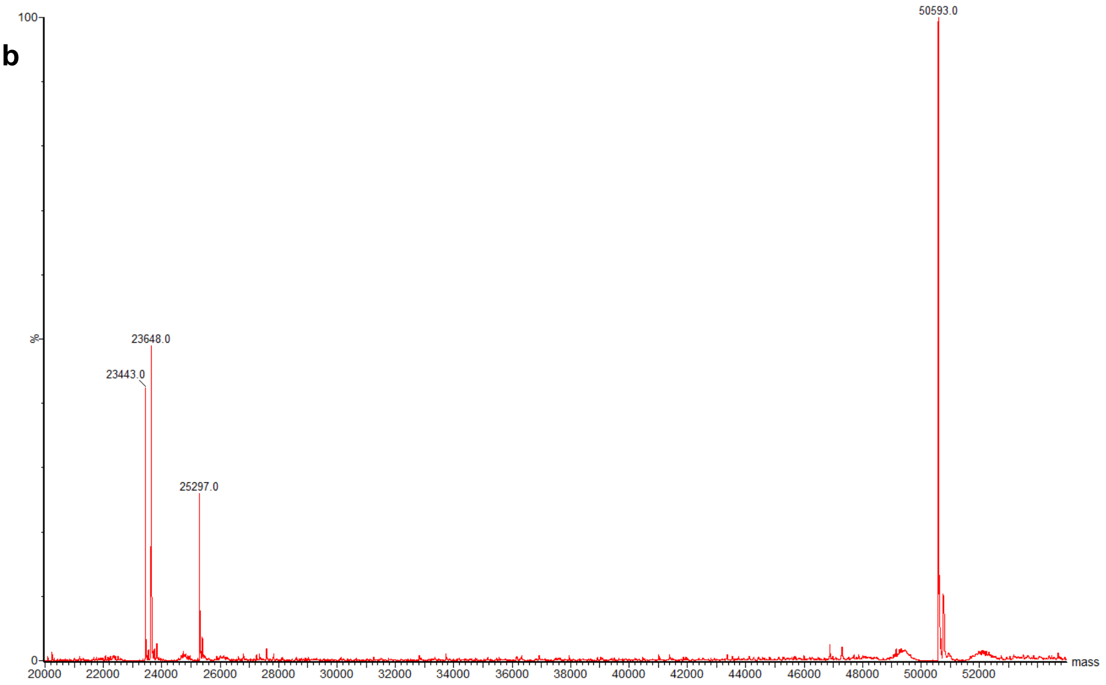

Figure S76. LC–MS spectra of Trastuzumab-maleimide after 66 h in the presence of GSH; **a**) ion series and **b**) deconvoluted spectrum.

Stability of Fc 274C-**11** in the presence of GSH (1 mM)

A 50 µL aliquot of Fc 274C-**11** (10 µM) in NaP_i buffer (20 mM, pH 8.0) was prepared. 2.5 µL of a 20 mM glutathione solution (6 mg glutathione dissolved in 1 µL of TrisHCl buffer (20 mM, pH 8.0)) was added at room temperature and the resulting mixture vortexed for 10 seconds. The resulting reaction mixture was then shaken at 37 ºC. After 66 h, the protein was deglycosylated using a PNGase F deglycosylation kit and reduced by adding 20 equiv of TCEP (stirred for 30 min at 25 ºC) and 10 µL aliquot of reaction mixture was analysed by LC–MS.

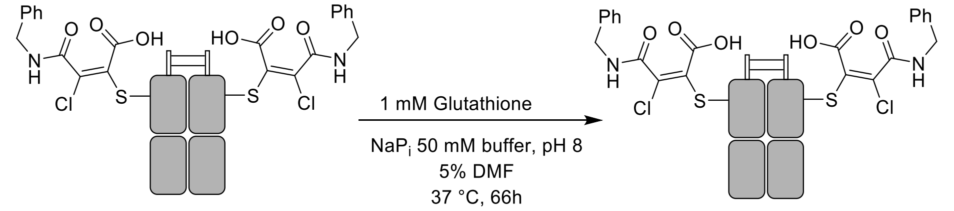

Fc 274C–**11**
Calcd MS: 25461

Fc 274C–**11**
Calcd MS: 25461

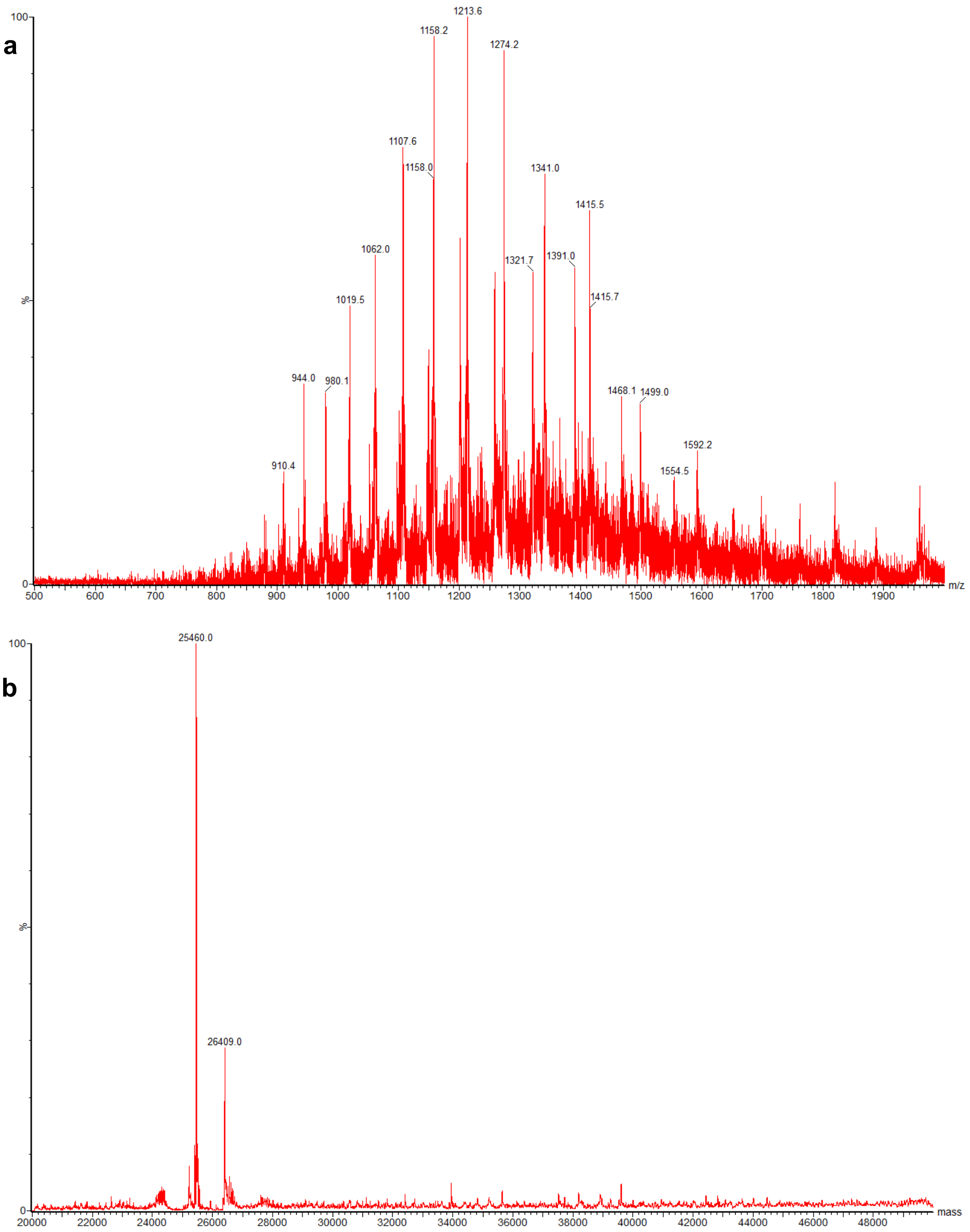

Figure S77. LC–MS spectra of Fc 274C-**11** after 66 h in the presence of GSH; **a**) ion series and **b**) deconvoluted spectrum.

Stability of Fc 274C-maleimide in the presence of GSH (1 mM)

A 50 µL aliquot of Fc 274C-maleimide (10 µM) in NaP_i buffer (20 mM, pH 8.0) was prepared. 2.5 µL of a 20 mM glutathione solution (6 mg glutathione dissolved in 1 µL of TrisHCl buffer (20 mM, pH 8.0)) was added at room temperature and the resulting mixture vortexed for 10 seconds. The resulting reaction mixture was then shaken at 37 ºC. After 66 h, the protein was deglycosylated using a PNGase F deglycosylation kit and reduced by adding 20 equiv of TCEP (stirred for 30 min at 25 ºC) and 10 µL aliquot of reaction mixture was analysed by LC–MS.

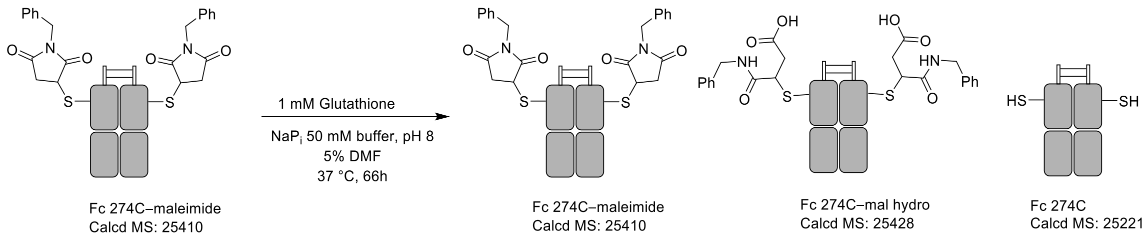

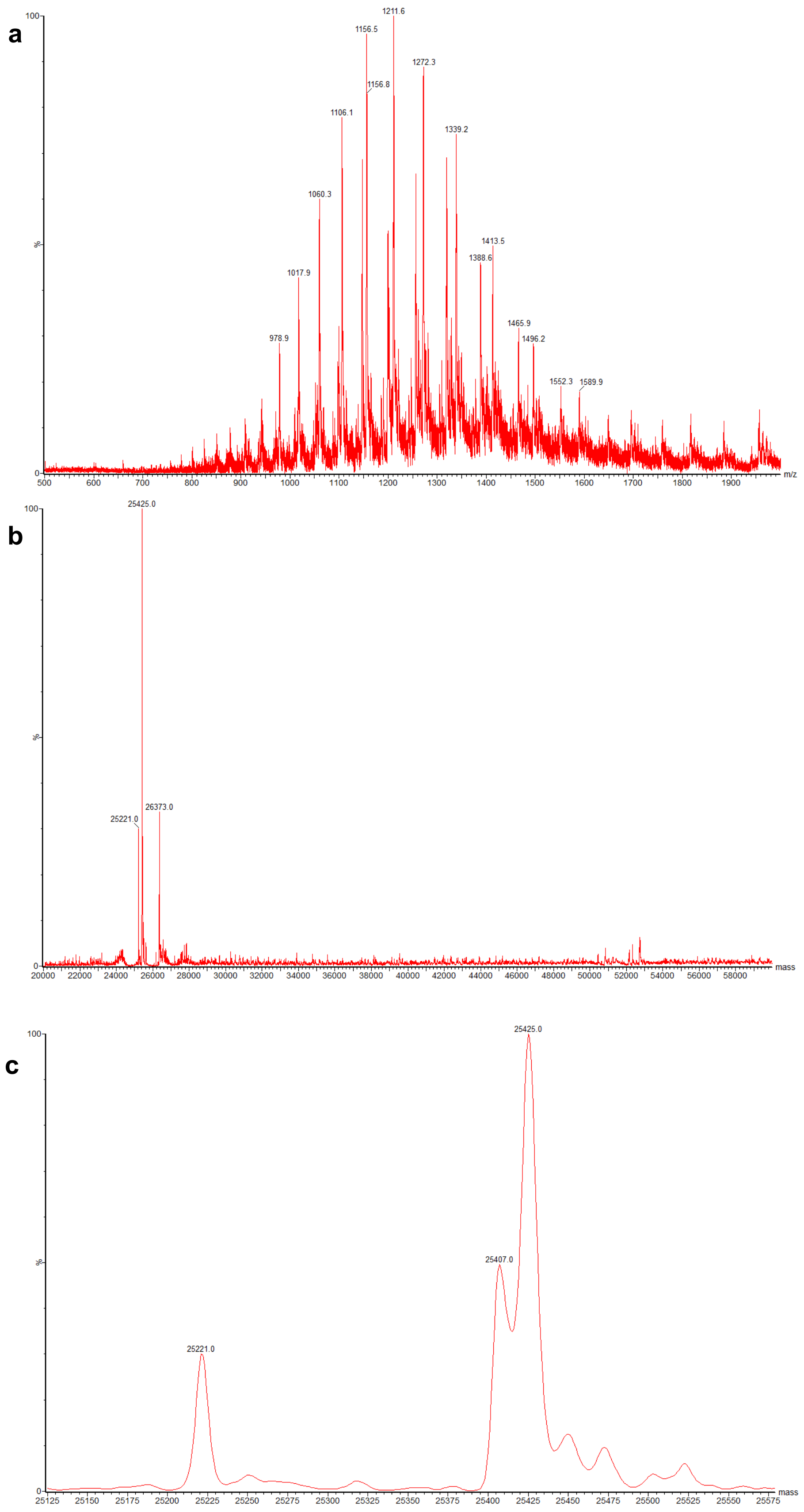

Figure S78. LC–MS spectra of Fc 274C-maleimide after 66 h in the presence of GSH; **a**) ion series, **b**) deconvoluted spectrum and **c**) highlighted deconvoluted area around modification.

Stability of Fc 289C-**11** in the presence of GSH (1 mM)

A 50 µL aliquot of Fc 289C-**11** (10 µM) in NaP_i buffer (20 mM, pH 8.0) was prepared. 2.5 µL of a 20 mM glutathione solution (6 mg glutathione dissolved in 1 µL of TrisHCl buffer (20 mM, pH 8.0)) was added at room temperature and the resulting mixture vortexed for 10 seconds. The resulting reaction mixture was then shaken at 37 ºC. After 66 h, the protein was deglycosylated using a PNGase F deglycosylation kit and reduced by adding 20 equiv of TCEP (stirred for 30 min at 25 ºC) and 10 µL aliquot of reaction mixture was analysed by LC–MS.

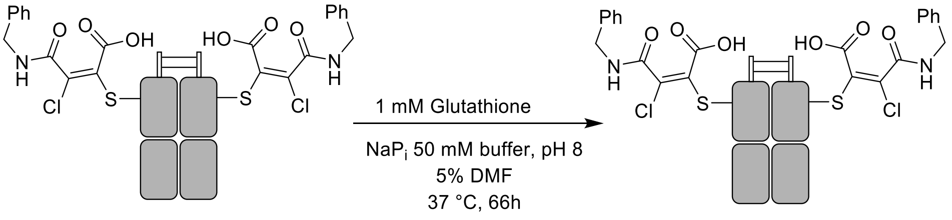

Fc 289C–**11**
Calcd MS: 25448

Fc 289C–**11**
Calcd MS: 25448

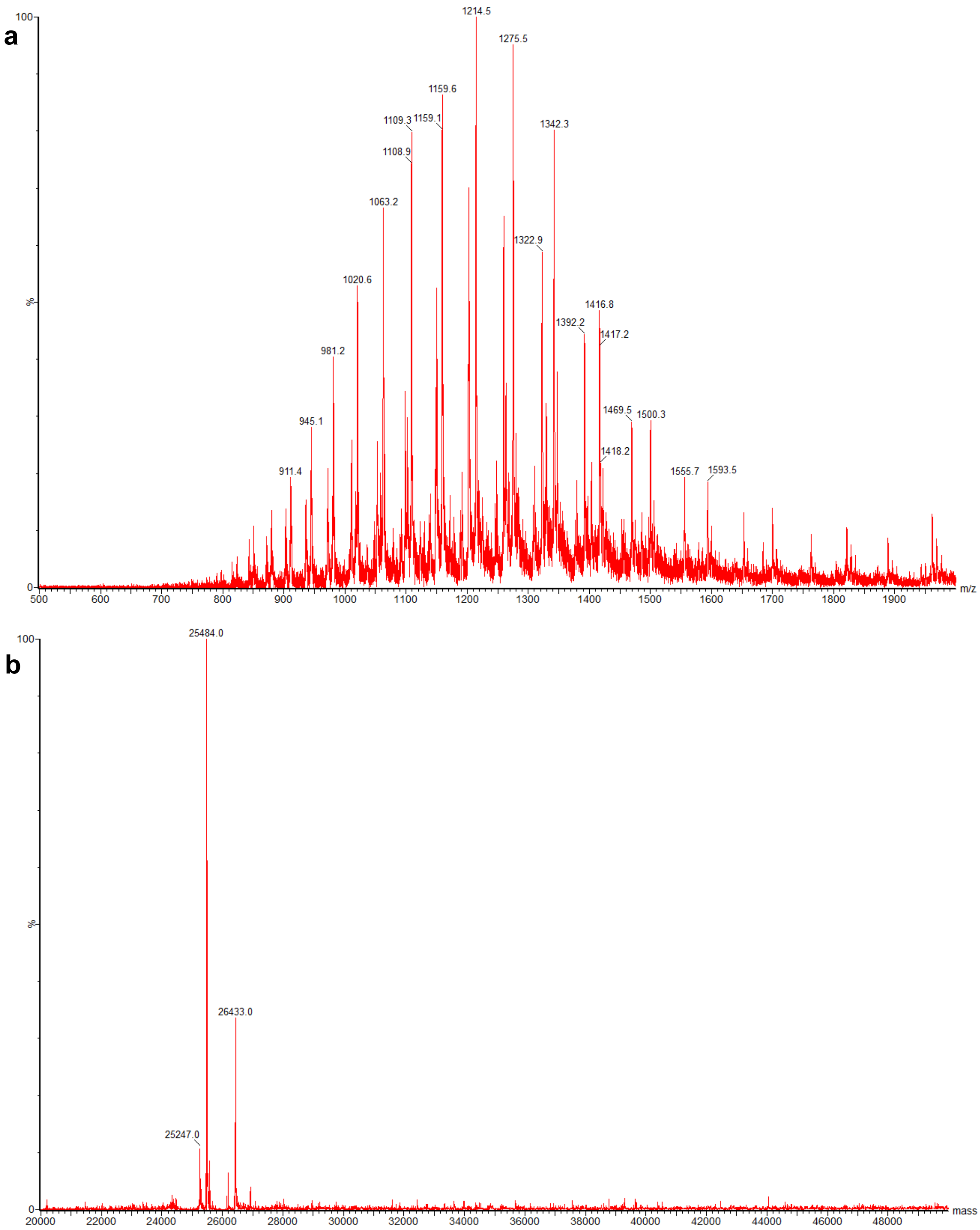

Figure S79. LC–MS spectra of Fc 289C-**11** after 66 h in the presence of GSH; **a**) ion series and b) deconvoluted spectrum.

Stability of Fc 289C -maleimide in the presence of GSH (1 mM)

A 50 μL aliquot of Fc 289C-maleimide (10 μM) in NaP_i buffer (20 mM, pH 8.0) was prepared. 2.5 μL of a 20 mM glutathione solution (6 mg glutathione dissolved in 1 μL of TrisHCl buffer (20 mM, pH 8.0)) was added at room temperature and the resulting mixture vortexed for 10 seconds. The resulting reaction mixture was then shaken at 37 ºC. After 66 h, the protein was deglycosylated using a PNGase F deglycosylation kit and reduced by adding 20 equiv of TCEP (stirred for 30 min at 25 ºC) and 10 μL aliquot of reaction mixture was analysed by LC–MS.

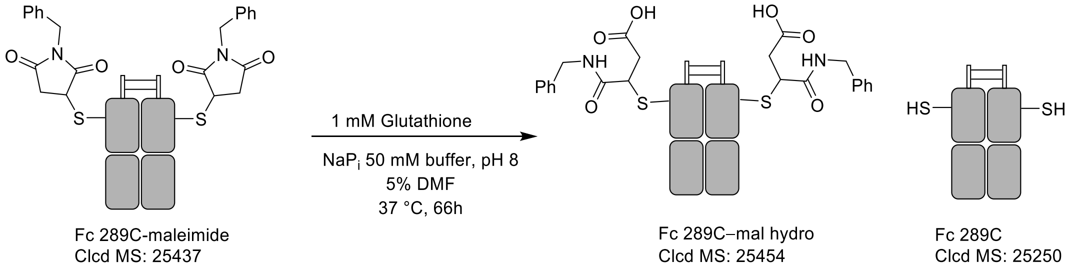

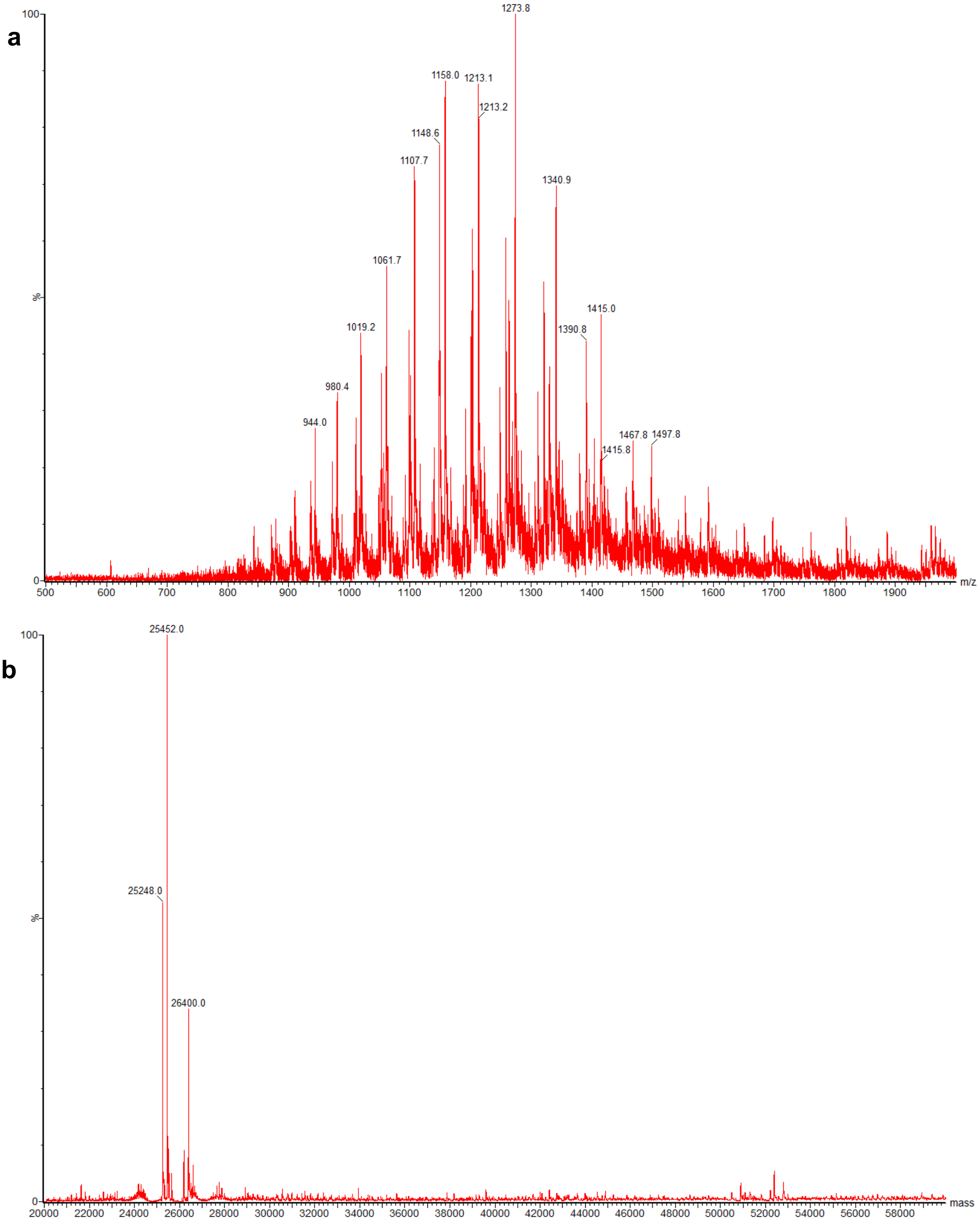

Figure S80. LC–MS spectra of Fc 289C-maleimide after 66 h in the presence of GSH; **a**) ion series and **b**) deconvoluted spectrum.

6. Competition Experiments

Competition Experiment Between **11** and benzyl maleimide with Fc 289C

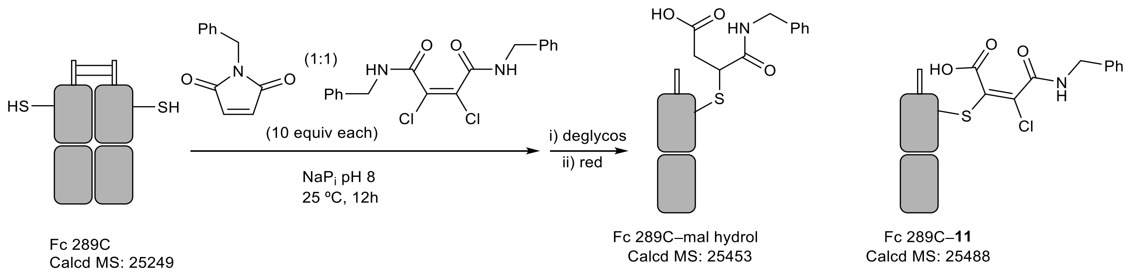

11.5 µL of a stock solution of Fc fragment 289C (87 µM) was added to an eppendorf containing 38.5 µL of NaP$_i$ buffer (pH 8.0, 50 mM). After this, 1 µL of a solution of benzyl maleimide (10 mM, 10 equiv, 5 equiv for each cysteine) and **11** (10 mM, 10 equiv) is added and the mixture is then shaken for 12 h at 25 ºC. Then, a 10 µL aliquot of the reaction mixture is deglycosylated using a PNGase F Glycan Cleavage Kit, reduced by adding 20 equiv of TCEP (30 min at 25 ºC) and analysed by LC–MS.

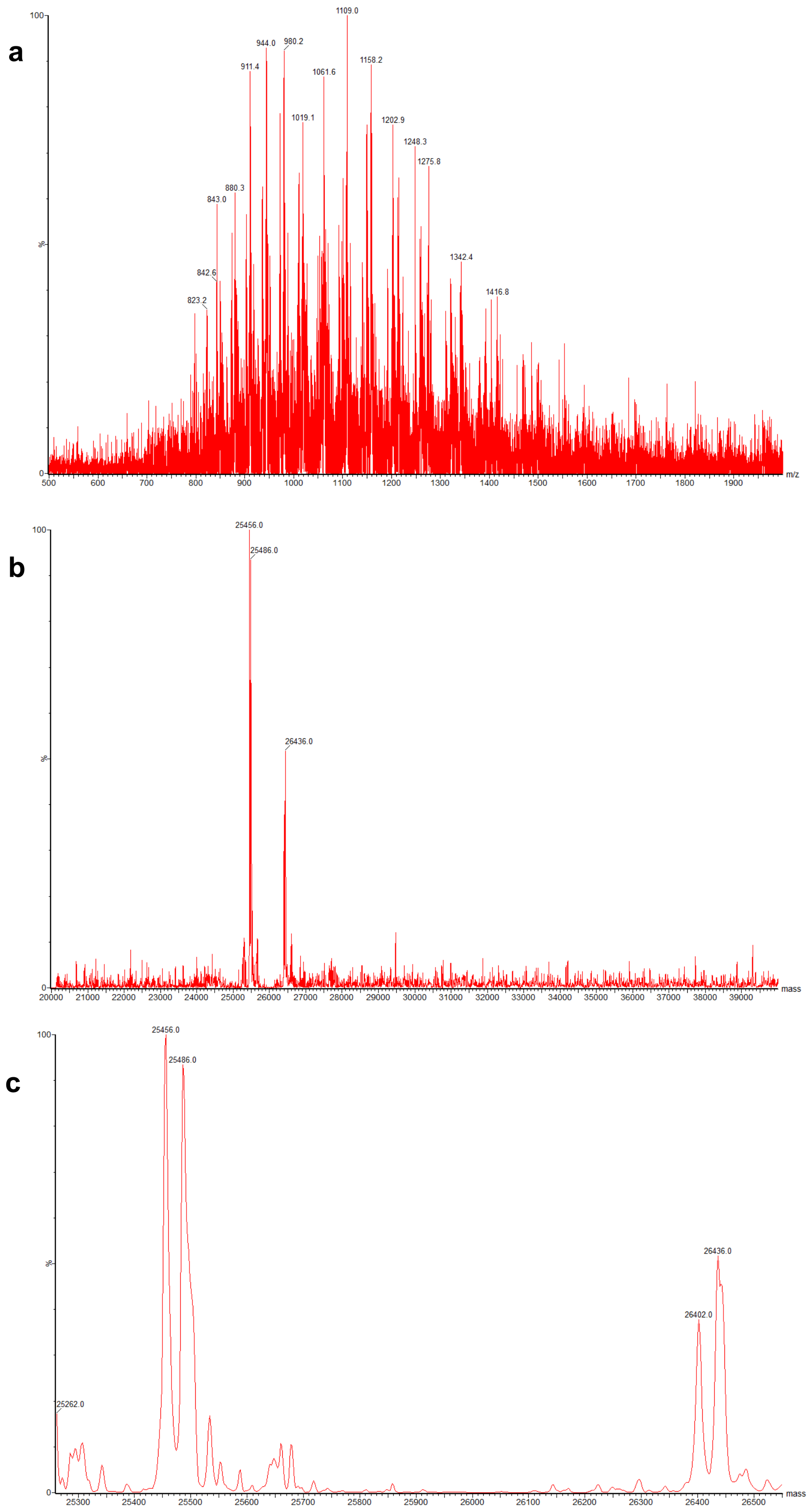

Figure S81. LC–MS spectra of Fc 289C-maleimide and Fc 289C-**11** after competition reaction; **a**) ion series, **b**) deconvoluted spectrum and **c**) highlighted deconvoluted area around modification.

7. LC-MS/MS Studies

The corresponding proteins (with and without modifications) were prepared for LC-MS/MS analysis. The corresponding proteins (Ub-K63C 0.5 µg, and HSA 2.0 µg) were denatured with 6 M urea in NH_4HCO_3 buffer (20 µL). Proteins were reduced with TCEP (10 mM) for 30 min, alkylated with iodo-acetamide (25 mM) for 30 min, digested with trypsin (0.5 µg) in the presence of 1 mM $CaCl_2$ and 2 M urea for 5 h at 37 °C and then acidified with acetic acid, desalted over C18 spin columns and analysed by nanoLC and ESI MS/MS. Mass adducts of +187.0633 Da, +205.0739 Da (hydrolysis modification) were searched on cysteines (as well as IAA modification).

Ub-7

Cysteine modification was observed on C63 as hydrolysis modification.

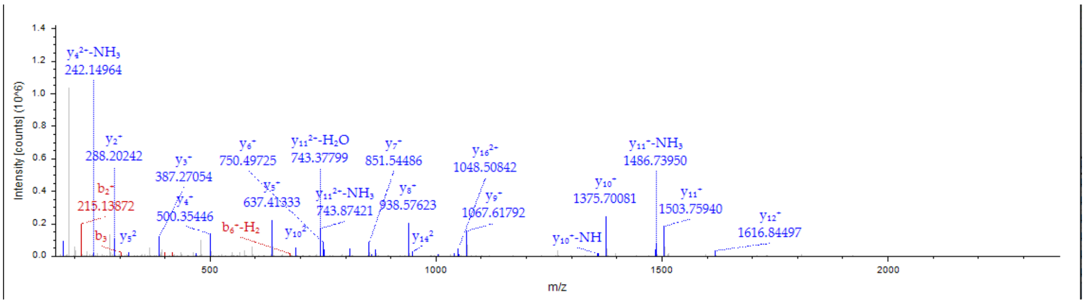

Figure S82. MS/MS spectrum of the m/z 770.72 triple charged ion of the cysteine modified tryptic peptide **TLSDYNIQ<u>C</u>ESTLHLVLR** from Ub-**7** containing a modification at the cysteine residue. The underscore relates to the modified amino acid.

HSA-7

Cysteine modification was observed on C34 as hydrolysis modification.

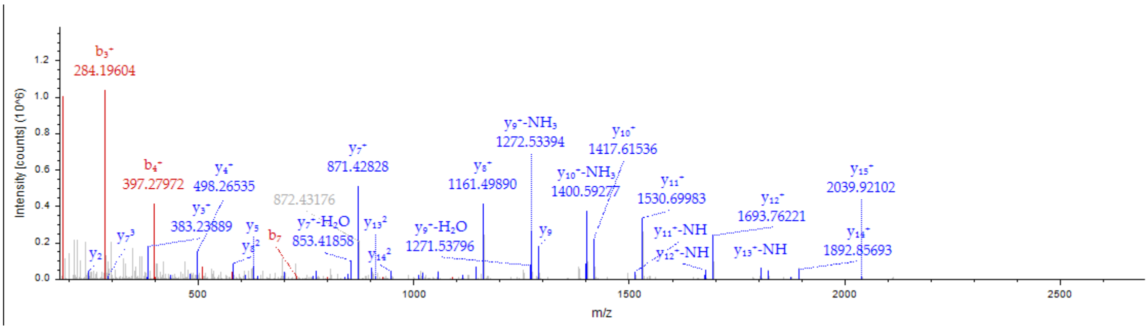

Figure S83. MS/MS spectrum of the m/z 880.11 triple charged ion of the cysteine modified tryptic peptide **ALVLIAFAQYLQQCPFEDHVK** from HSA-**7** containing a modification at the cysteine residue. The underscore relates to the modified amino acid.

Trastuzumab V205C-14

Cysteine modification was observed on C205 of the light chain for the linear linkage and C205 and K207 for the cyclic linkage.

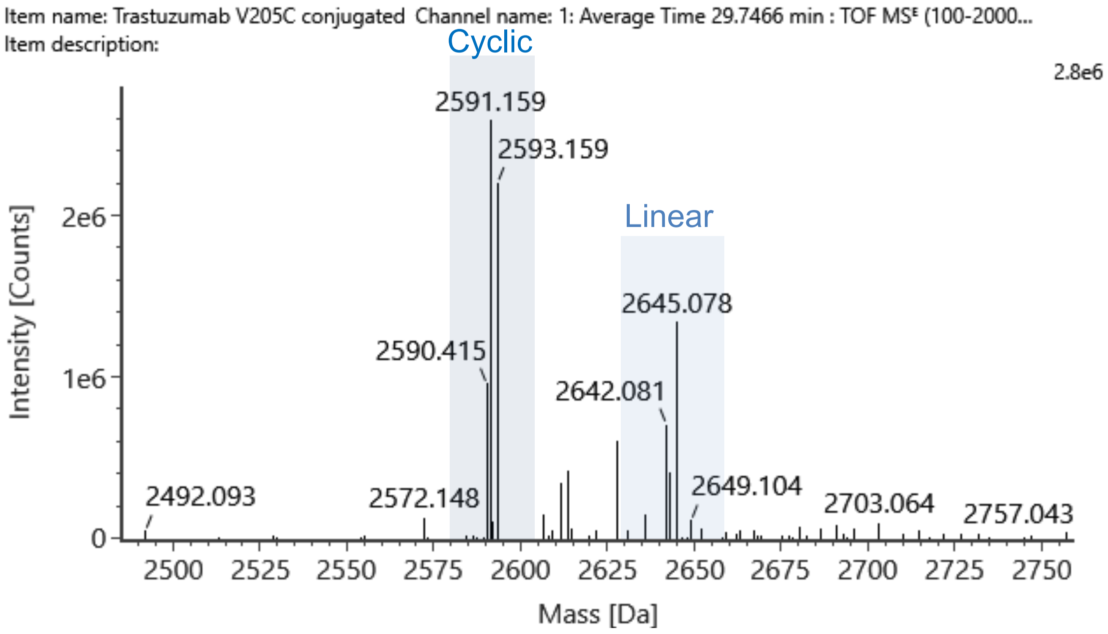

Figure S84. Spectrum of the of the cysteine modified tryptic peptide **VYA<u>C</u>EVTHQGLSSP<u>C</u>T<u>K</u>** from Trastuzumab V205C-**14,** containing two cysteine residues one with the modification from **14**, the other with an acetamide modification. The underscore relates to the modified amino acids. Conjugation with **14** can form 2 linkages as described on the text, one referring to the cyclic linkage (2591 Da) which also involves the lysine on 207 and the other referring to the linear linkage (2645.11 Da).

8. CD Studies

Circular dichroism (CD) spectroscopy was used to analyze protein secondary structure in solution. Samples were concentrated to 10 nM in NaP$_i$ buffer (50 mM, pH 8.0). CD measurements were recorded using a Chirascan spectrophotometer equipped with a Quantum TC125 temperature control unit (25 ºC). The data was acquired in a 0.1 cm path length with a response time of 1 s, a per-point acquisition delay of 5 ms and a pre- and post-scan delay of 50 ms. Spectra were averaged over three scans, in a wavelength range from 200 nm to 260 nm, and the spectrum from a blank sample containing only buffer was subtracted from the averaged data.

CD comparison of Ub and Ub-14

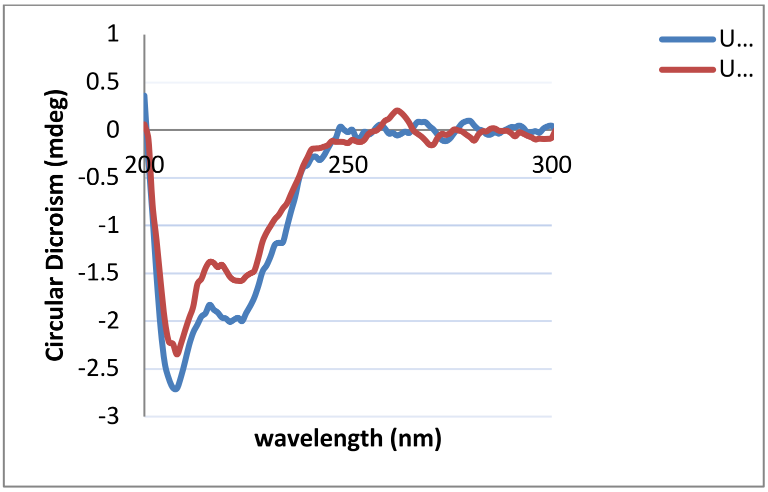

Figure S85. CD traces of Ub and Ub-**14**.

CD comparing HSA and HSA-14

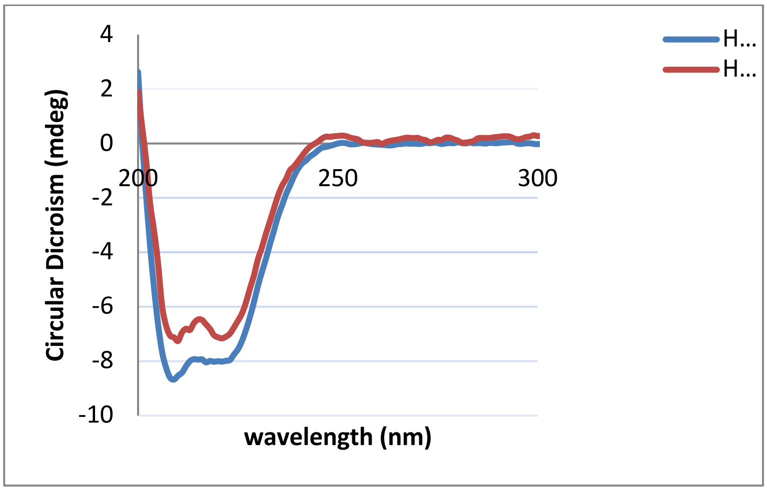

Figure S86. CD traces HSA and HSA-**14**.

9. Molecular Dynamics (MD) Simulations

MD simulations were run on HER-2 carrying derivative **14** at Cys-205 and on the corresponding cyclic derivative with Lys-207. The X-ray structure of HER-2 (PDB entry 1N8Z) was used for the starting structure of the protein.[10] The calculations were carried out with AMBER 18 package[11] implemented with ff14SB[12] and GAFF.[13] The partial charges of the non-standard residues were set to fit the electrostatic potential generated with HF/6-31G(d) by RESP.[14] The charges were calculated according to the Merz-Singh-Kollman scheme using Gaussian 09.[15] In all cases, the studied protein was immersed in a water box with a 10 Å buffer of TIP3P water molecules.[16] The system was neutralized by adding explicit counter ions (Cl^-). A two-stage geometry optimization approach was performed. The first stage minimizes only the positions of solvent molecules and the second stage is an unrestrained minimization of all the atoms in the simulation cell. The systems were then gently heated by incrementing the temperature from 0 to 300 K under a constant pressure of 1 atm and periodic boundary conditions. Harmonic restraints of 30 kcal·mol^{-1} were applied to the solute, and the Andersen temperature-coupling scheme was used to control and equalize the temperature. The time step was kept at 1 fs during the heating stages, allowing potential inhomogeneities to self-adjust. Long-range electrostatic effects were modelled using the particle-mesh-Ewald method.[17] An 8 Å cut-off was applied to Lennard-Jones interactions. Each system was equilibrated for 2 ns with a 2-fs time step at a constant volume and temperature of 300 K. Production trajectories were then run for additional 0.5 μs under the same simulation conditions.

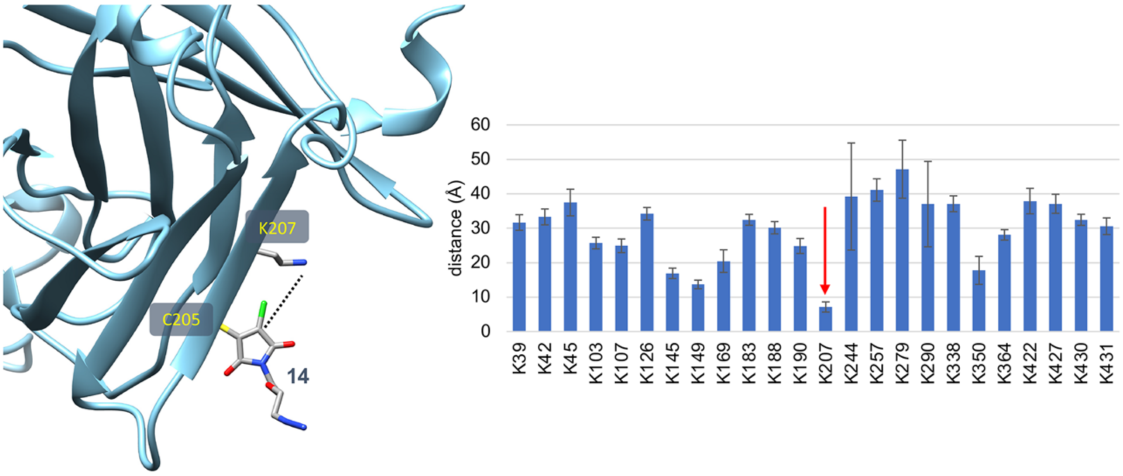

Figure S87. Monitoring the distance from all lysine residues to the reactive carbon (C-Cl) on HER 2-**14** (pdb entry 1N8Z) by 0.5 μs MD simulations. An illustrative frame derived from the MD simulations is also shown. The error is shown as ± SD.

10. References

[1] C. Bonneaud, M. Decostanzi, J. Burgess, G. Trusiano, T. Burgess, R. Bongiovanni, C. Joly-Duhamel, C. M. Friesen, *RSC Adv.* **2018**, *8*, 32664–32671.

[2] A. Sánchez, E. Pedroso, A. Grandas, *Eur. J. Org. Chem.* **2010**, *2010*, 2600–2606.

[3] M. T. W. Hearn, A. D. Ward, *Aust. J. Chem.* **1977**, *30*, 2031–2043.

[4] G. Bonse, H. U. Blank, *Liebigs Ann. Chem.* **1981**, *1981*, 1658–1664.

[5] I. S. Alam, A. A. Neves, T. H. Witney, J. Boren, K. M. Brindle, *Bioconjugate Chem.* **2010**, *21*, 884–891.

[6] B. Lee, S. Sun, E. Jiménez-Moreno, A. A. Neves, G. J. L. Bernardes, *Bioorg. Med. Chem.* **2018**, *26*, 3060–3064.

[7] P. R. Lindstedt, F. A. Aprile, P. Sormanni, R. Rakoto, C. M. Dobson, G. J. L. Bernardes, M. Vendruscolo, *Cell Chem. Biol.* **2021**, *28*, 70–77 e75.

[8] N. Y. Sardesai, J. K. Barton, *JBIC* **1997**, *2*, 762–771.

[9] H. Klinker, C. Haas, N. Harrer, P. B. Becker, F. Mueller-Planitz, *PLoS One* **2014**, *9*, e104029.

[10] H. S. Cho, K. Mason, K. X. Ramyar, A. M. Stanley, S. B. Gabelli, D. W. Denney, Jr., D. J. Leahy, *Nature* **2003**, *421*, 756–760.

[11] I. Y. B.-S. D.A. Case, S.R. Brozell, D.S. Cerutti, T.E. Cheatham, III, V.W.D. Cruzeiro, T.A. Darden, R.E. Duke, D. Ghoreishi, M.K. Gilson, H. Gohlke, A.W. Goetz, D. Greene, R Harris, N. Homeyer, S. Izadi, A. Kovalenko, T. Kurtzman, T.S. Lee, S. LeGrand, P. Li, C. Lin, J. Liu, T. Luchko, R. Luo, D.J. Mermelstein, K.M. Merz, Y. Miao, G. Monard, C. Nguyen, H. Nguyen, I. Omelyan, A. Onufriev, F. Pan, R. Qi, D.R. Roe, A. Roitberg, C. Sagui, S. Schott-Verdugo, J. Shen, C.L. Simmerling, J. Smith, R. Salomon-Ferrer, J. Swails, R.C. Walker, J. Wang, H. Wei, R.M. Wolf, X. Wu, L. Xiao, D.M. York and P.A. Kollman, **2018**.

[12] J. A. Maier, C. Martinez, K. Kasavajhala, L. Wickstrom, K. E. Hauser, C. Simmerling, *J. Chem. Theory Comput.* **2015**, *11*, 3696–3713.

[13] J. Wang, R. M. Wolf, J. W. Caldwell, P. A. Kollman, D. A. Case, *J. Comput. Chem.* **2004**, *25*, 1157–1174.

[14] C. I. Bayly, P. Cieplak, W. Cornell, P. A. Kollman, *J. Phys. Chem.* **1993**, *97*, 10269–10280.

[15] M. J. Frisch, G. W. Trucks, H. B. Schlegel, G. E. Scuseria, M. A. Robb, J. R. Cheeseman, G. Scalmani, V. Barone, G. A. Petersson, H. Nakatsuji, X. Li, M. Caricato, A. V. Marenich, J. Bloino, B. G. Janesko, R. Gomperts, B. Mennucci, H. P. Hratchian, J. V. Ortiz, A. F. Izmaylov, J. L. Sonnenberg, Williams, F. Ding, F. Lipparini, F. Egidi, J. Goings, B. Peng, A. Petrone, T. Henderson, D. Ranasinghe, V. G. Zakrzewski, J. Gao, N. Rega, G. Zheng, W. Liang, M. Hada, M. Ehara, K. Toyota, R. Fukuda, J. Hasegawa, M. Ishida, T. Nakajima, Y. Honda, O. Kitao, H. Nakai, T. Vreven, K. Throssell, J. A. Montgomery Jr., J. E. Peralta, F. Ogliaro, M.

J. Bearpark, J. J. Heyd, E. N. Brothers, K. N. Kudin, V. N. Staroverov, T. A. Keith, R. Kobayashi, J. Normand, K. Raghavachari, A. P. Rendell, J. C. Burant, S. S. Iyengar, J. Tomasi, M. Cossi, J. M. Millam, M. Klene, C. Adamo, R. Cammi, J. W. Ochterski, R. L. Martin, K. Morokuma, O. Farkas, J. B. Foresman, D. J. Fox, Wallingford, CT, **2016**.

[16] P. Kenji Kiyohara Keith E. Gubbins Athanassios Z, *Mol. Phys.* **1998**, *94*, 803–808.

[17] T. Darden, D. York, L. Pedersen, *J. Chem. Phys.* **1993**, *98*, 10089–10092.

11. ^1H and ^{13}C NMR Spectra

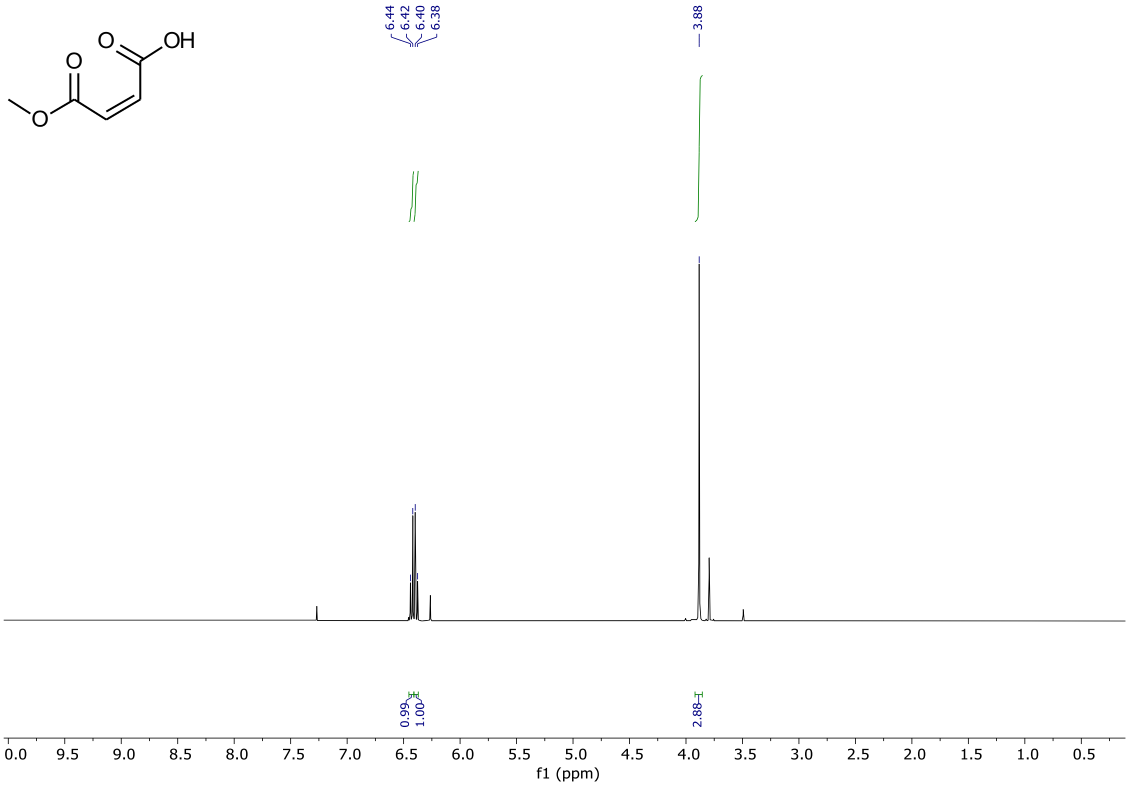

Figure S88. ^1H NMR Spectrum of **1**.

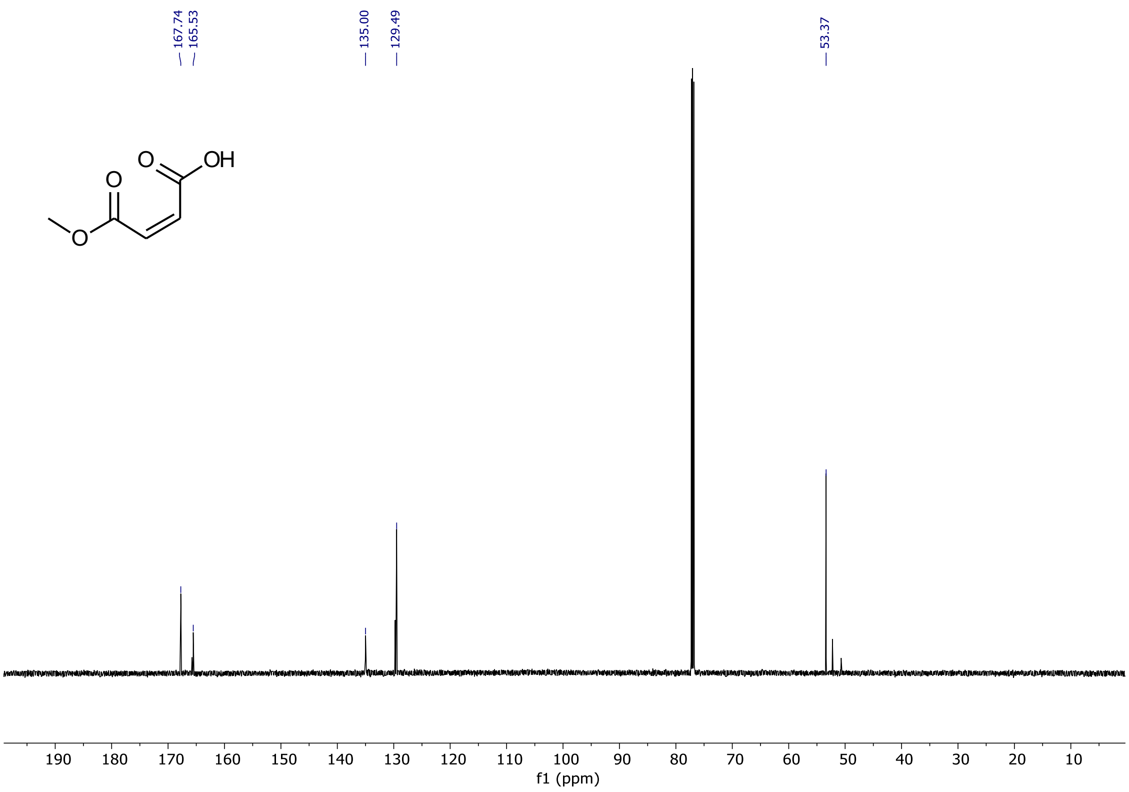

Figure S89. ^{13}C NMR Spectrum of **1**.

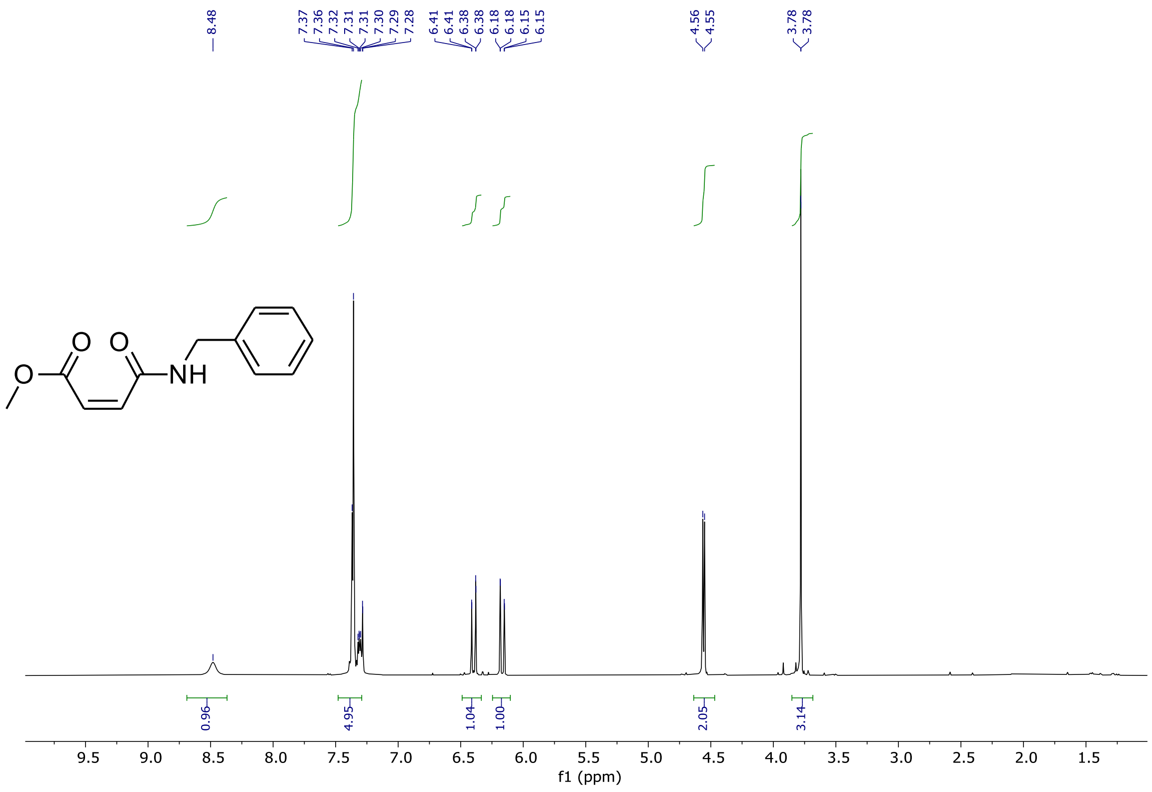

Figure S90. ^{1}H NMR Spectrum of **2**.

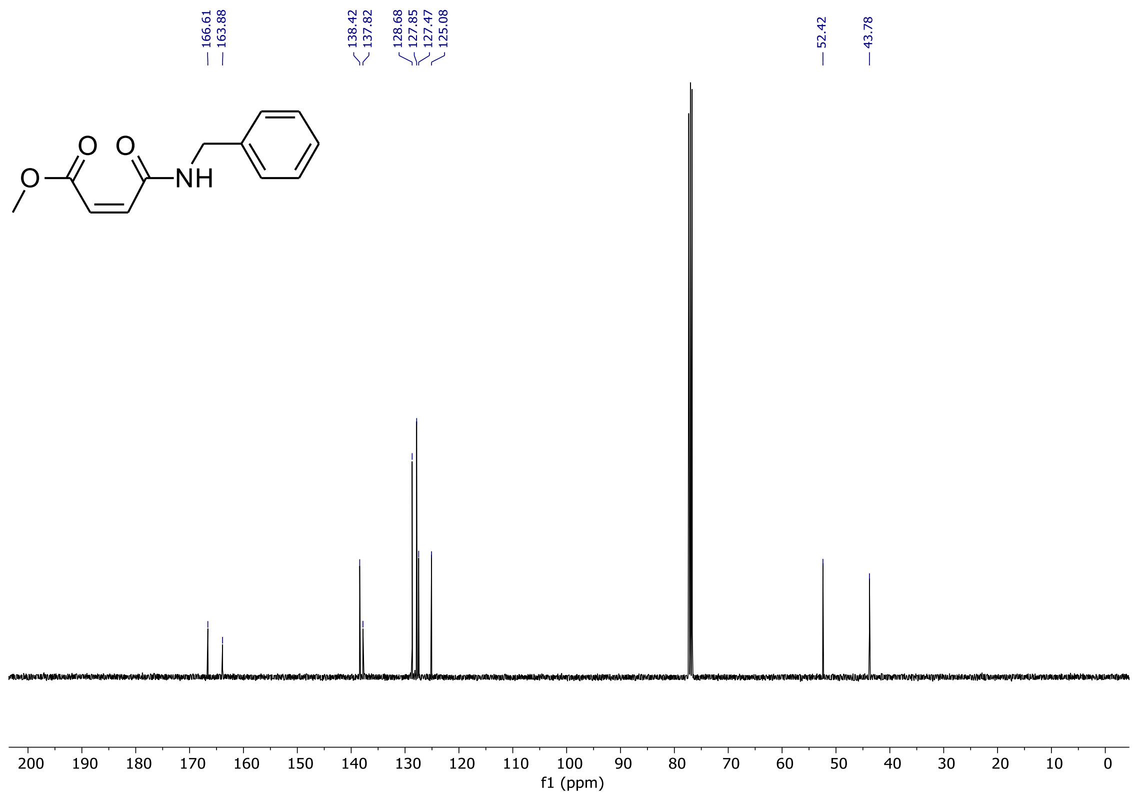

Figure S91. ^{13}C NMR Spectrum of **2**.

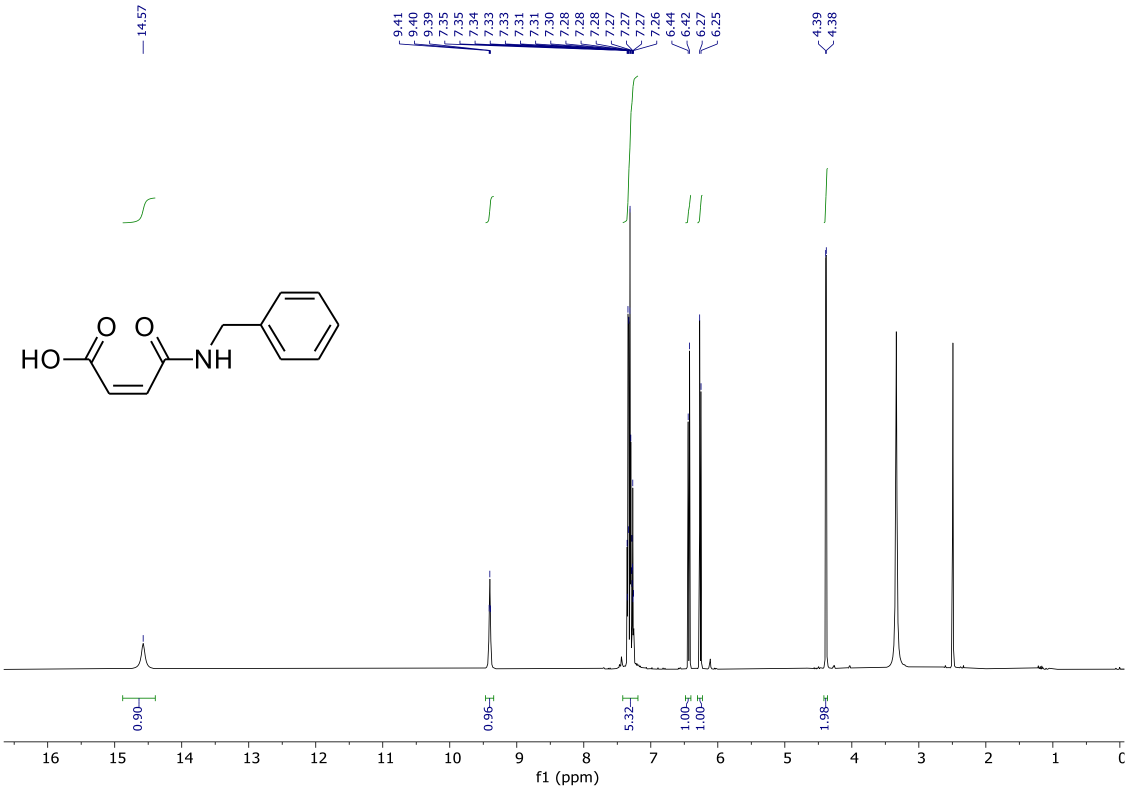

Figure S92. ^{1}H NMR Spectrum of **4**.

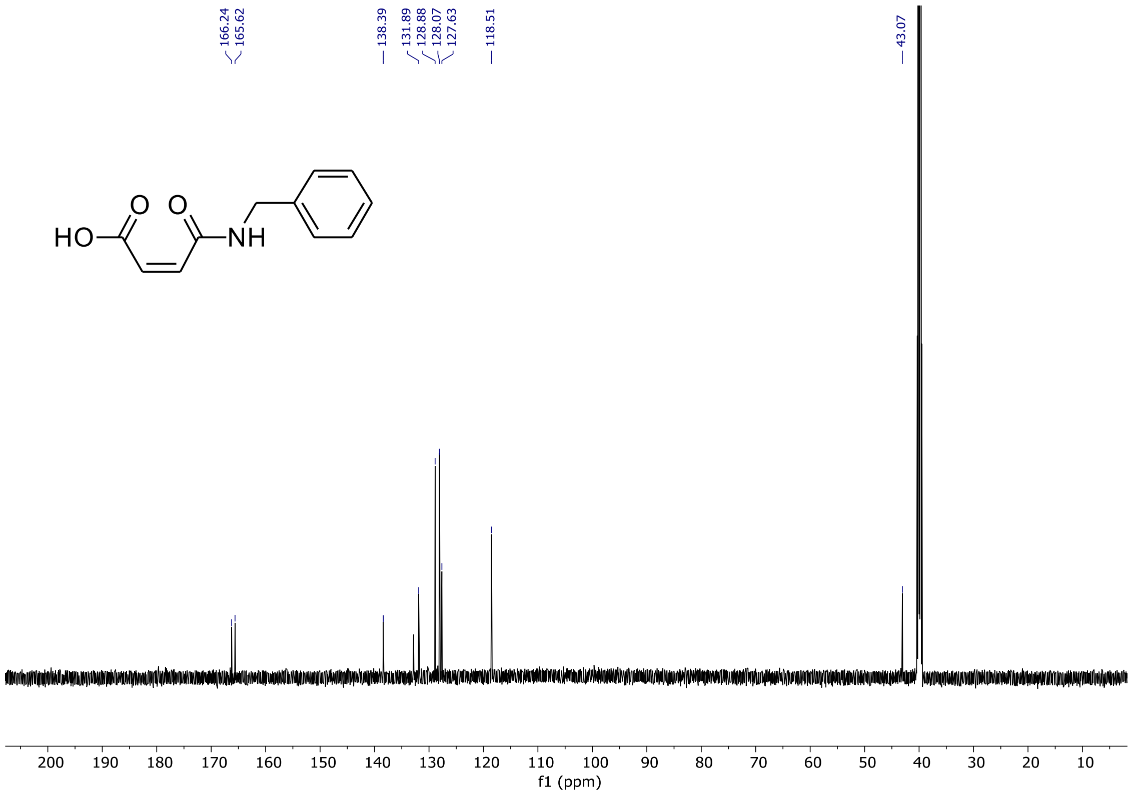

Figure S93. ^{13}C NMR Spectrum of **4**.

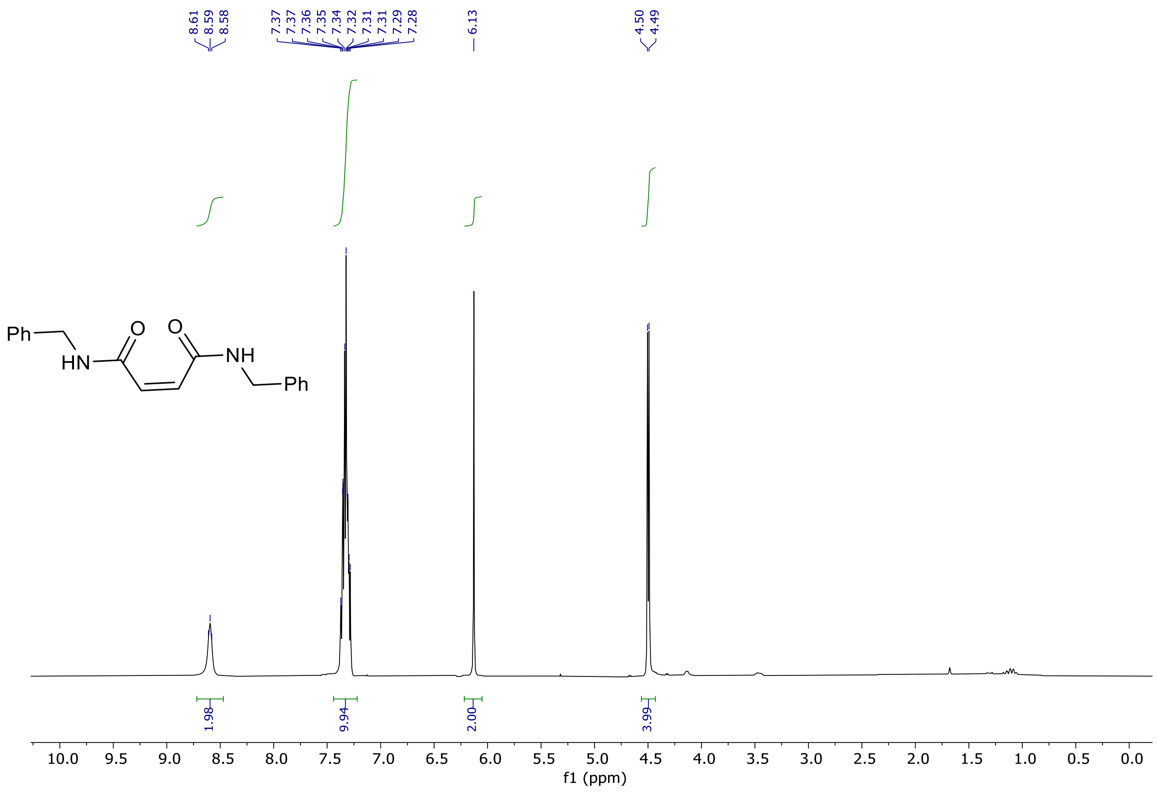

Figure S94. ^{1}H NMR Spectrum of **5**.

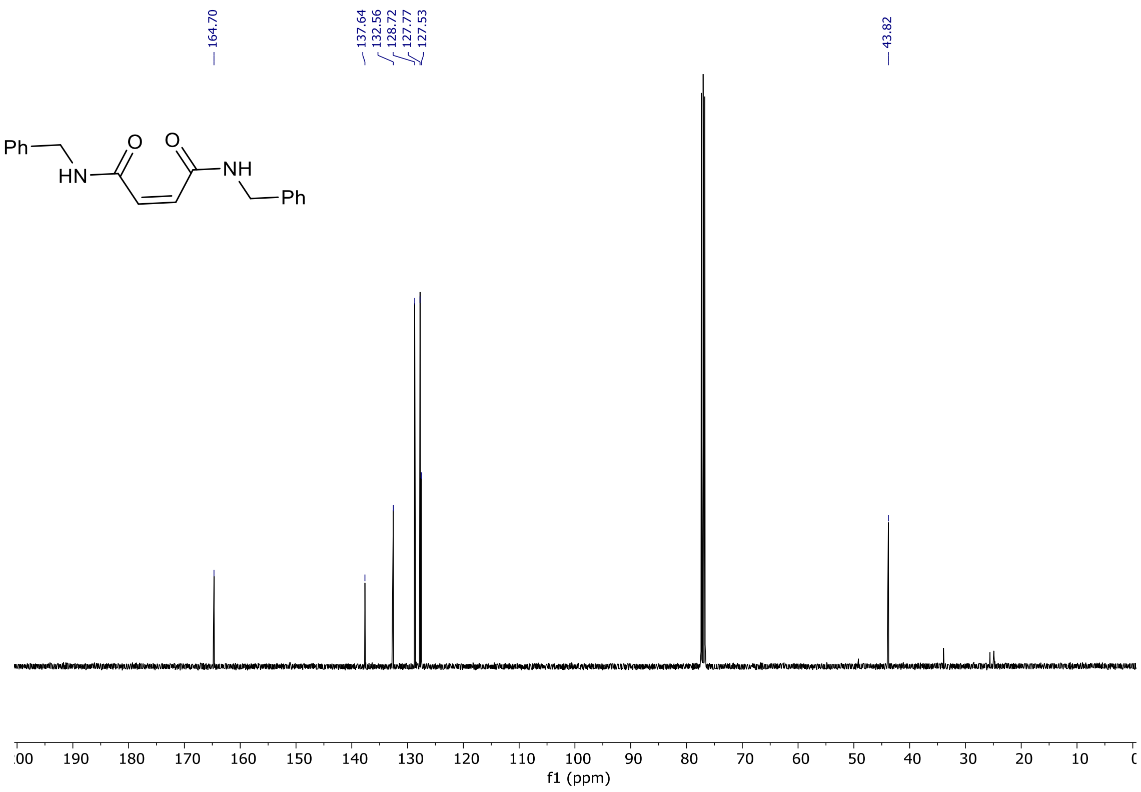

Figure S95. ^{13}C NMR Spectrum of **5**.

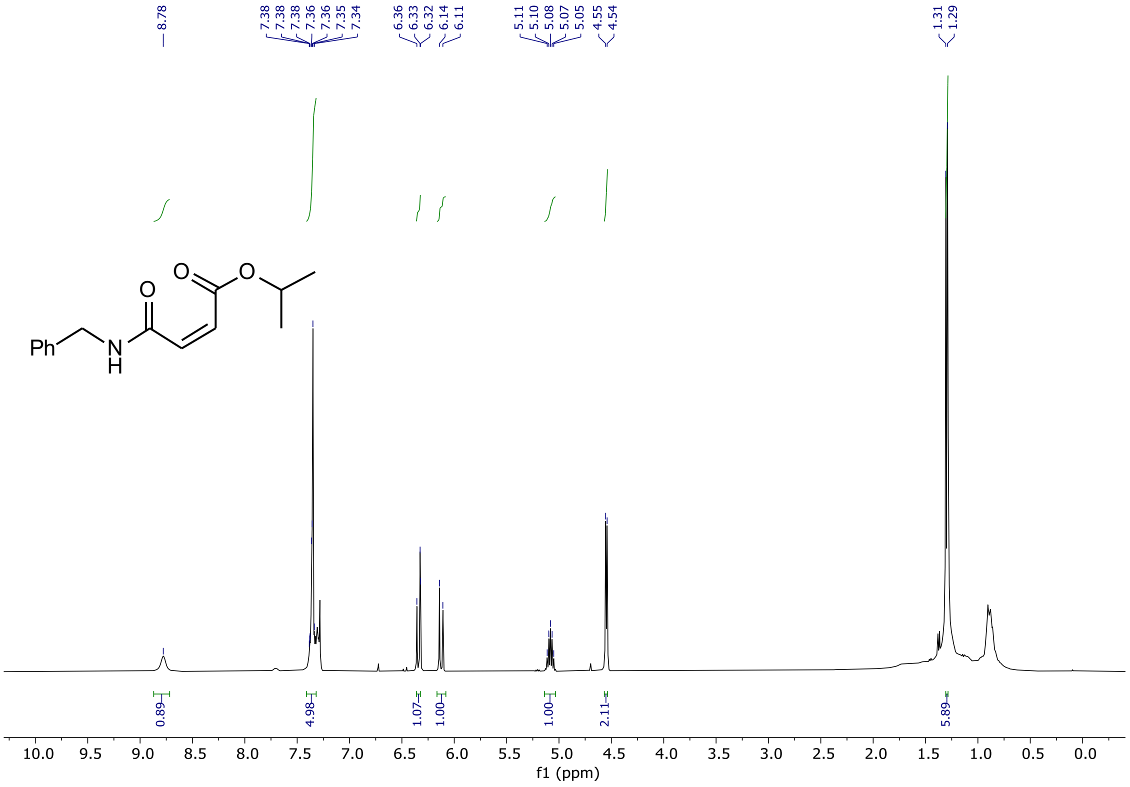

Figure S96. ^{1}H NMR Spectrum of **6**.

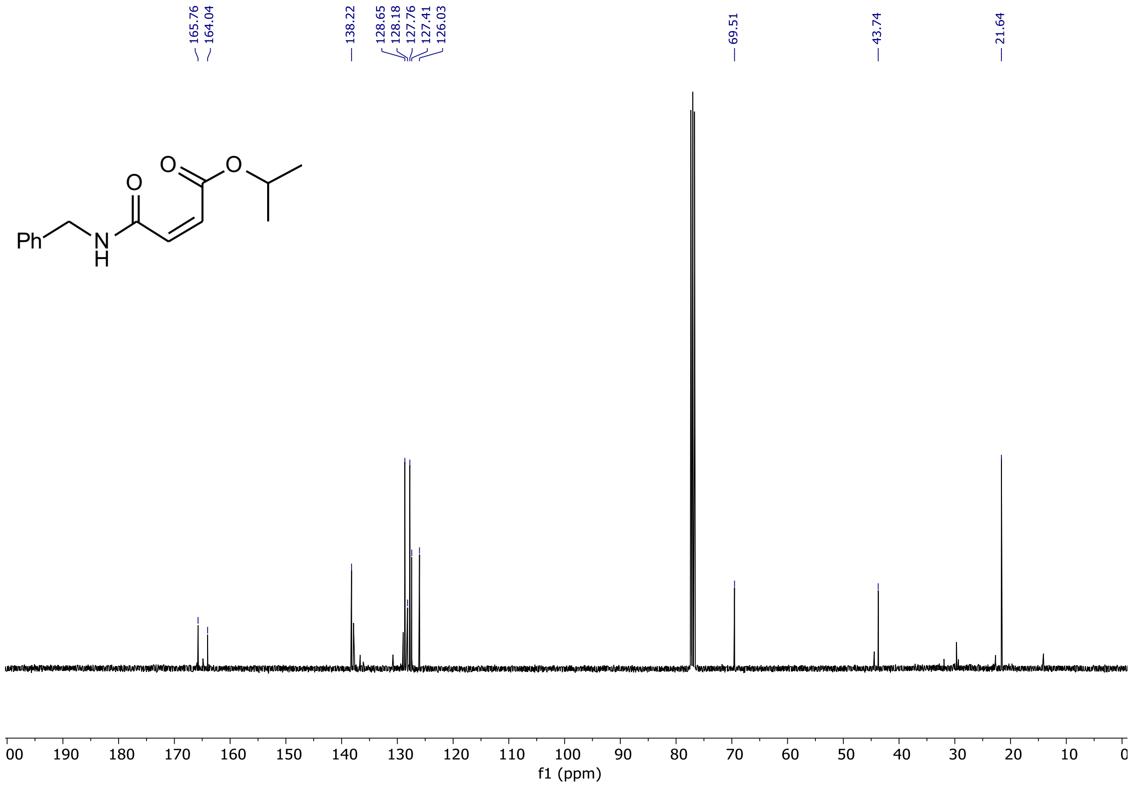

Figure S97. ^{13}C NMR Spectrum of **6**.

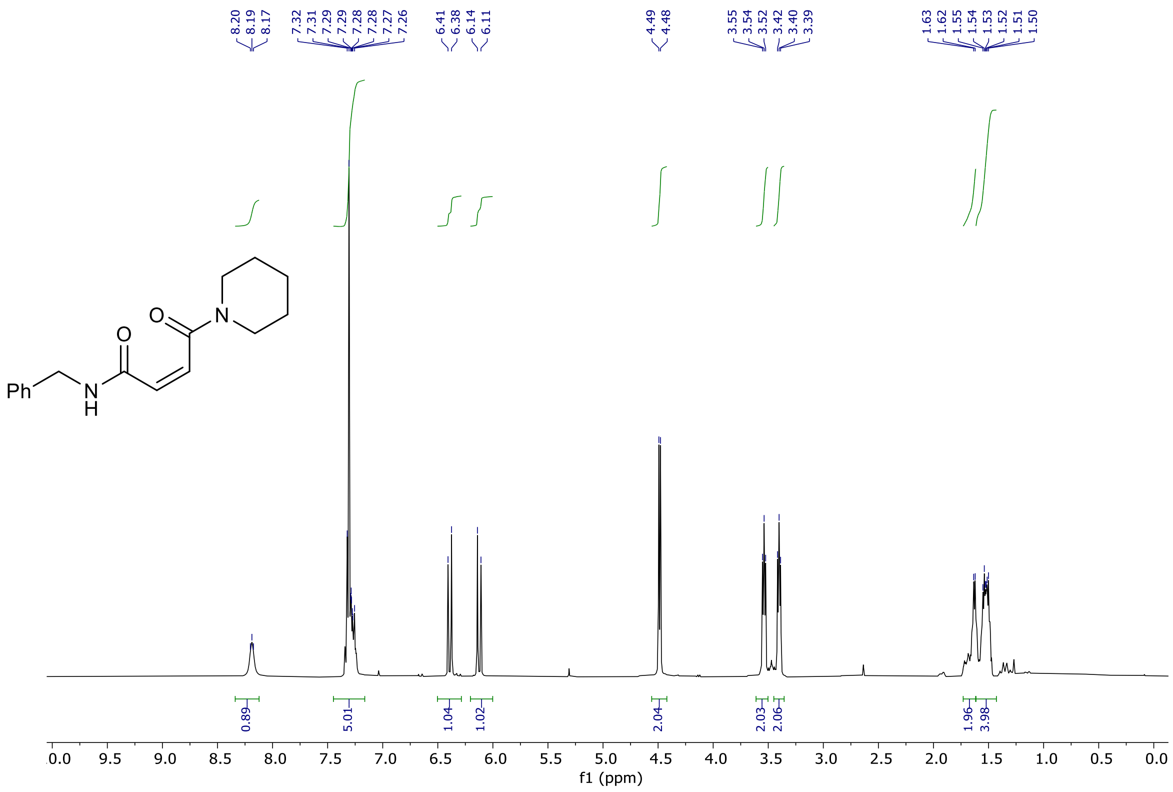

Figure S98. ^{1}H NMR Spectrum of **7**.

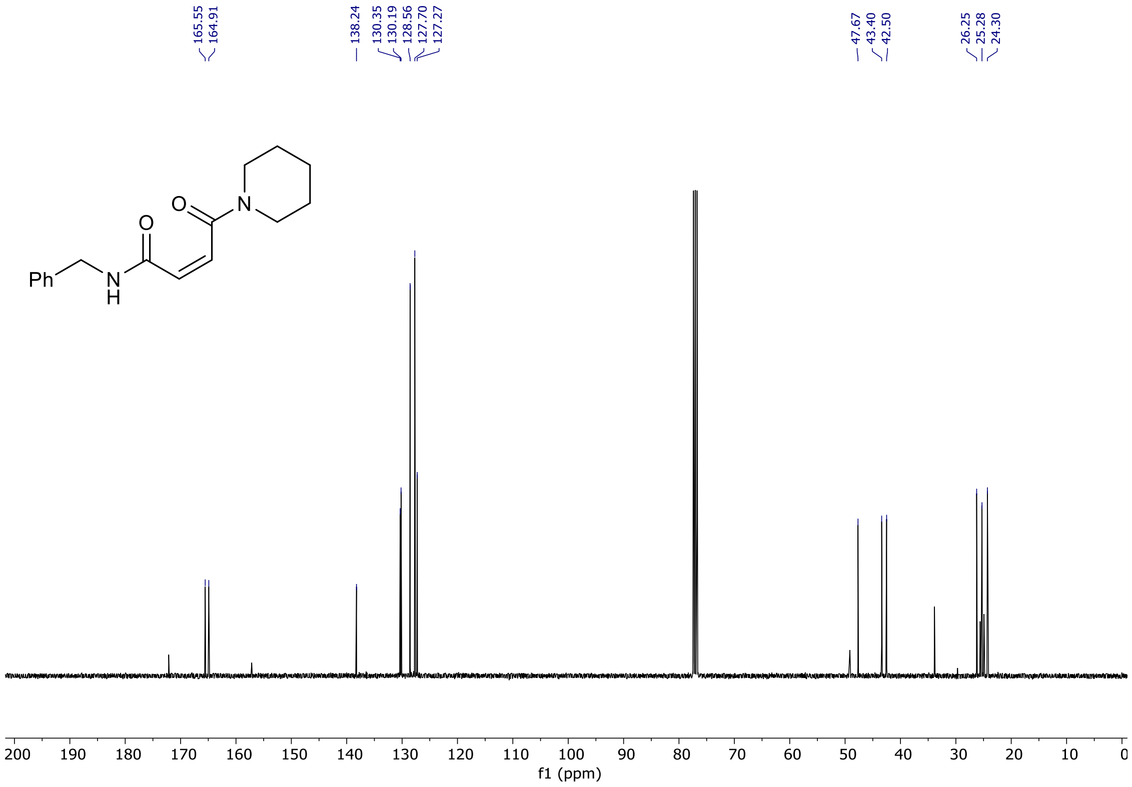

Figure S99. ^{13}C NMR Spectrum of **7**.

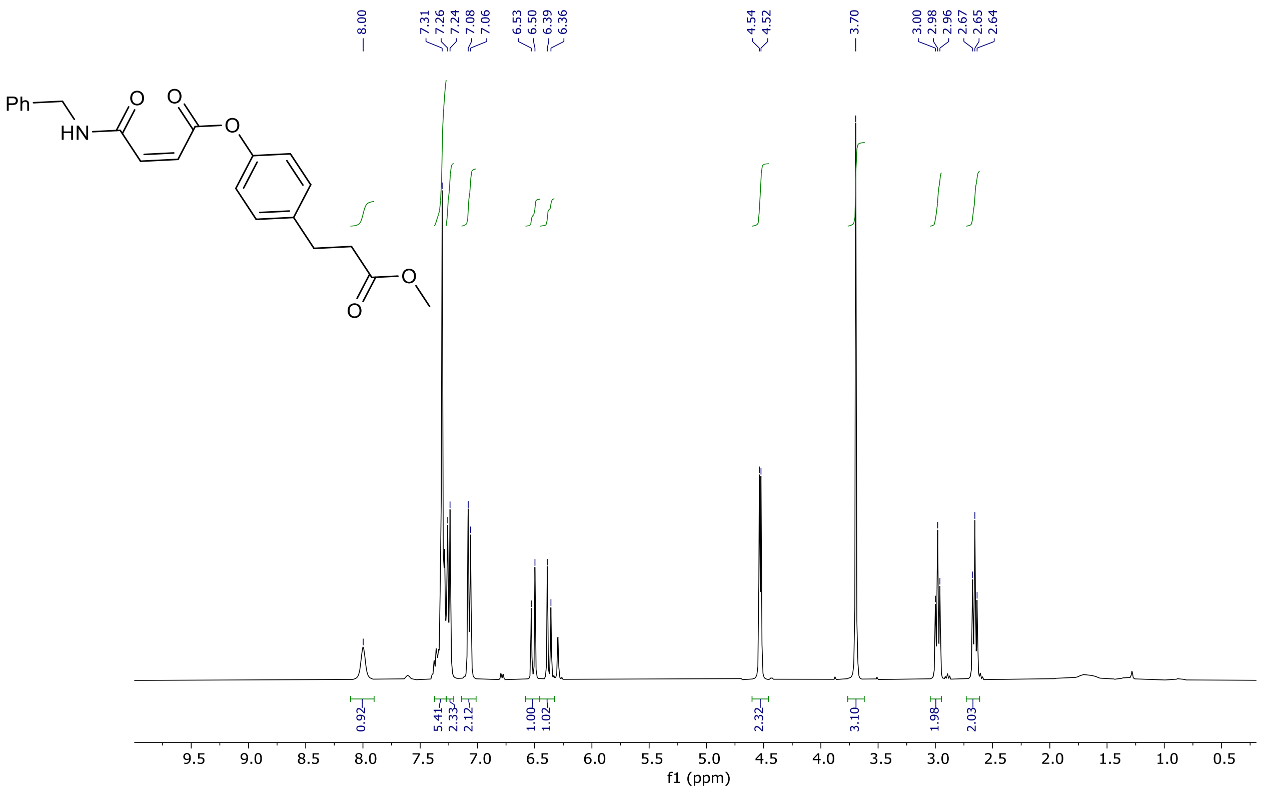

Figure S100. ^{1}H NMR Spectrum of **8**.

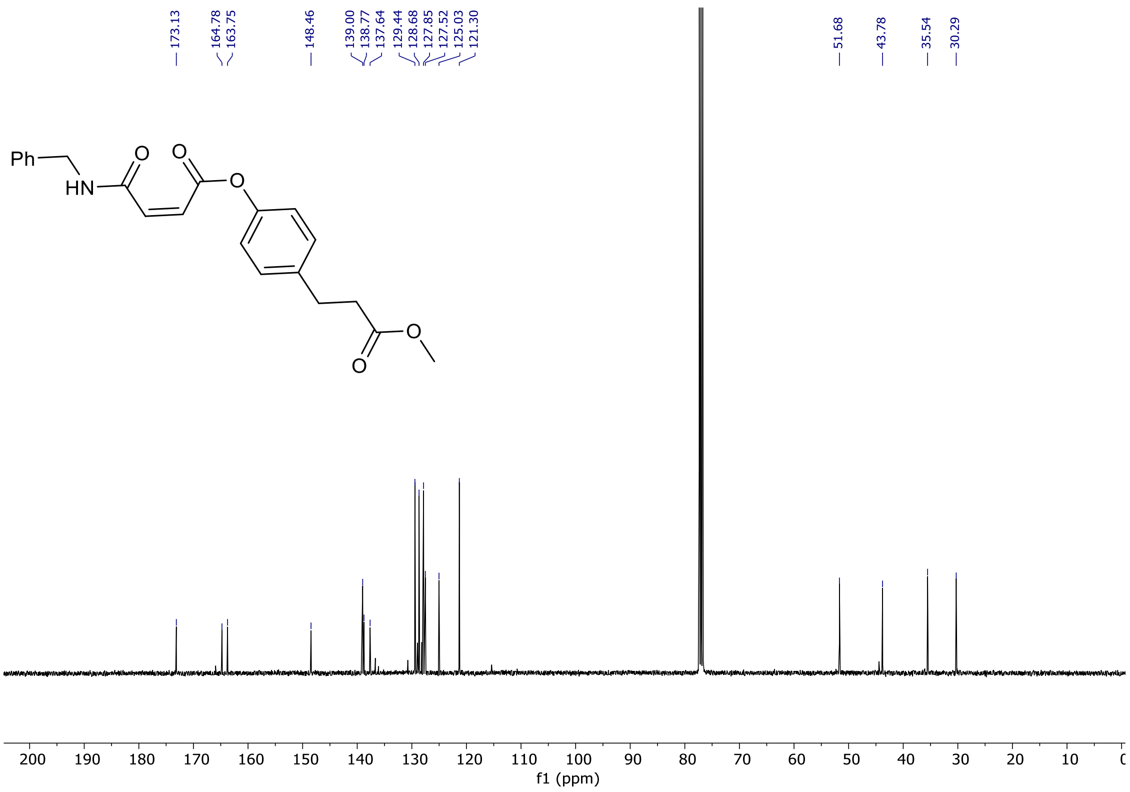

Figure S101. ^{13}C NMR Spectrum of **8**.

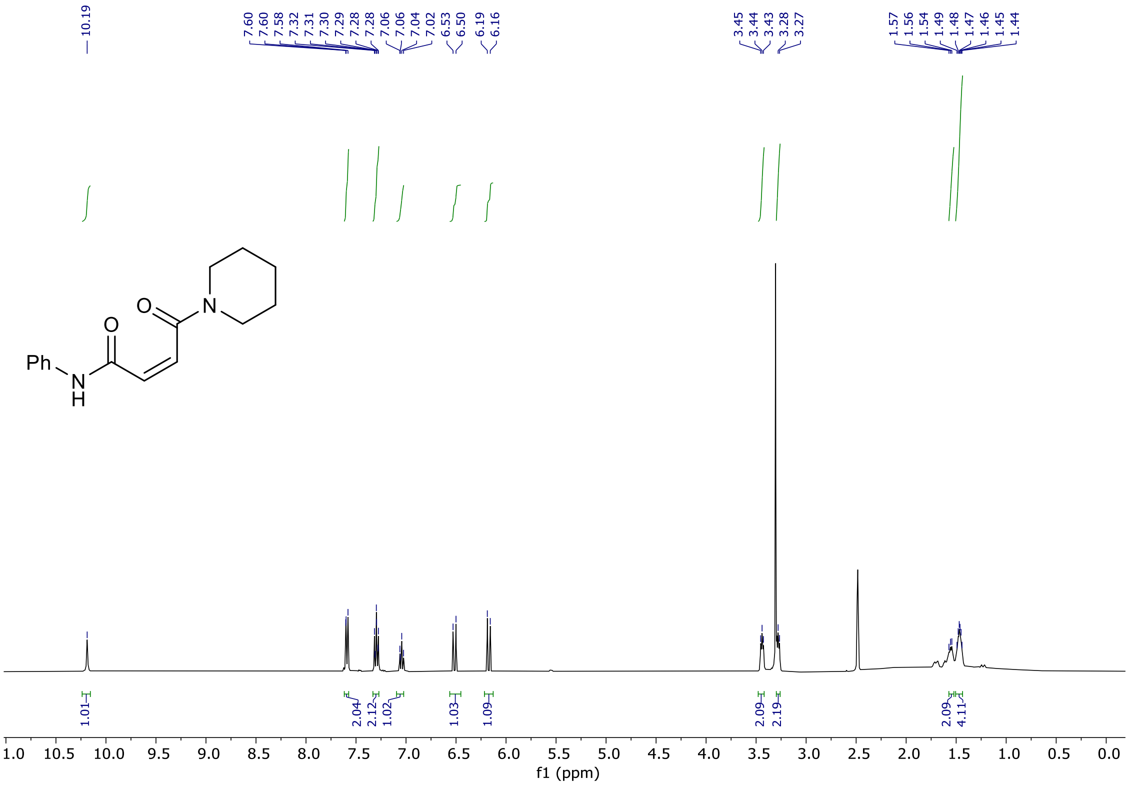

Figure S102. ^{1}H NMR Spectrum of **9**.

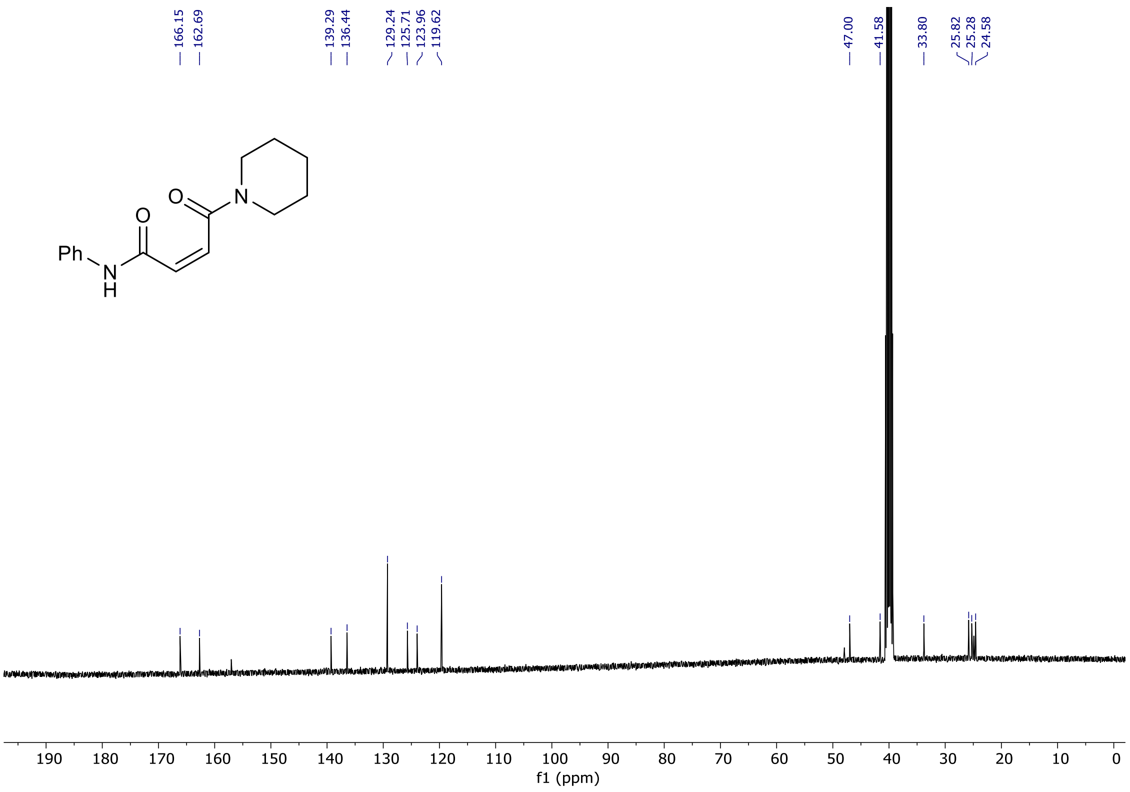

Figure S103. ^{13}C NMR Spectrum of **9**.

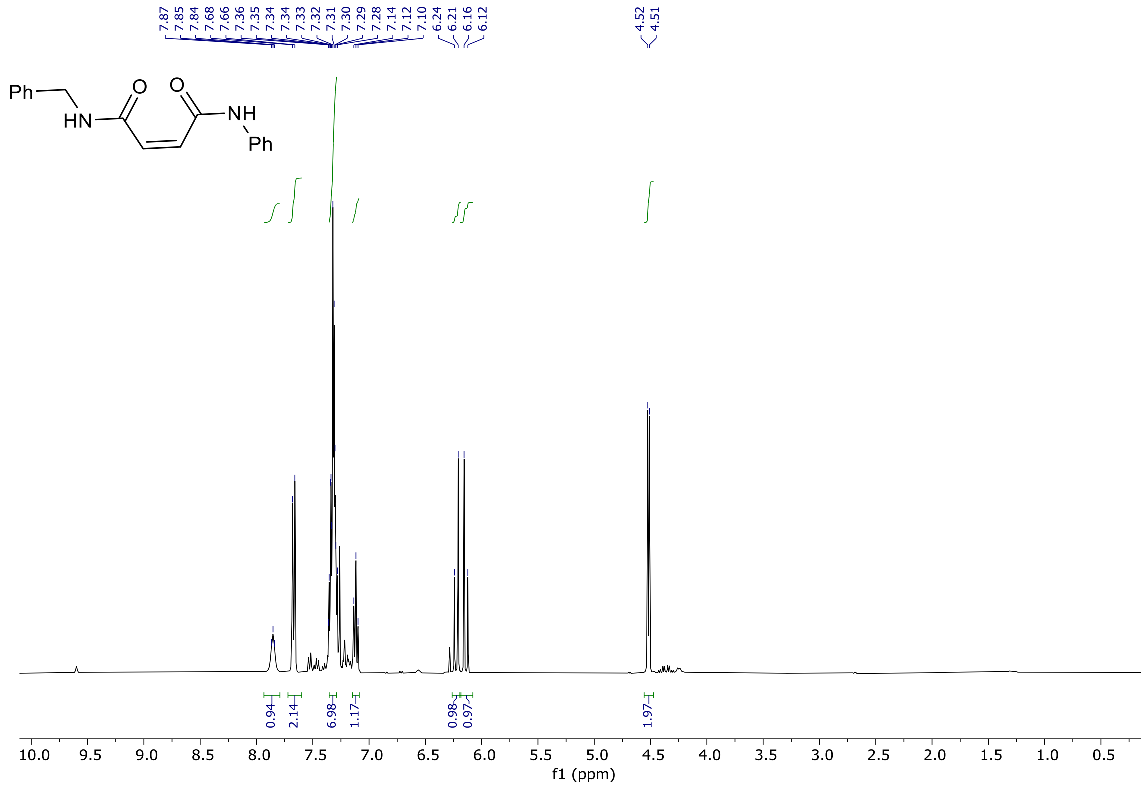

Figure S104. ^{1}H NMR Spectrum of **10**.

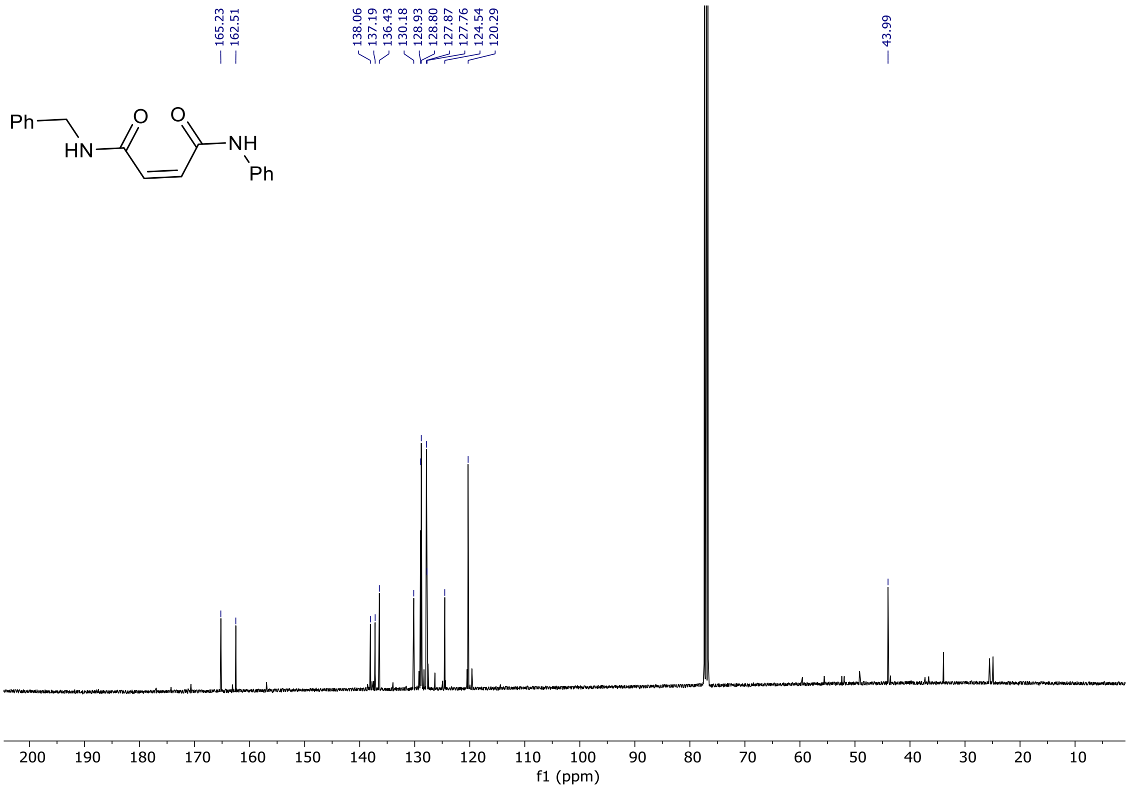

Figure S105. ^{13}C NMR Spectrum of **10**.

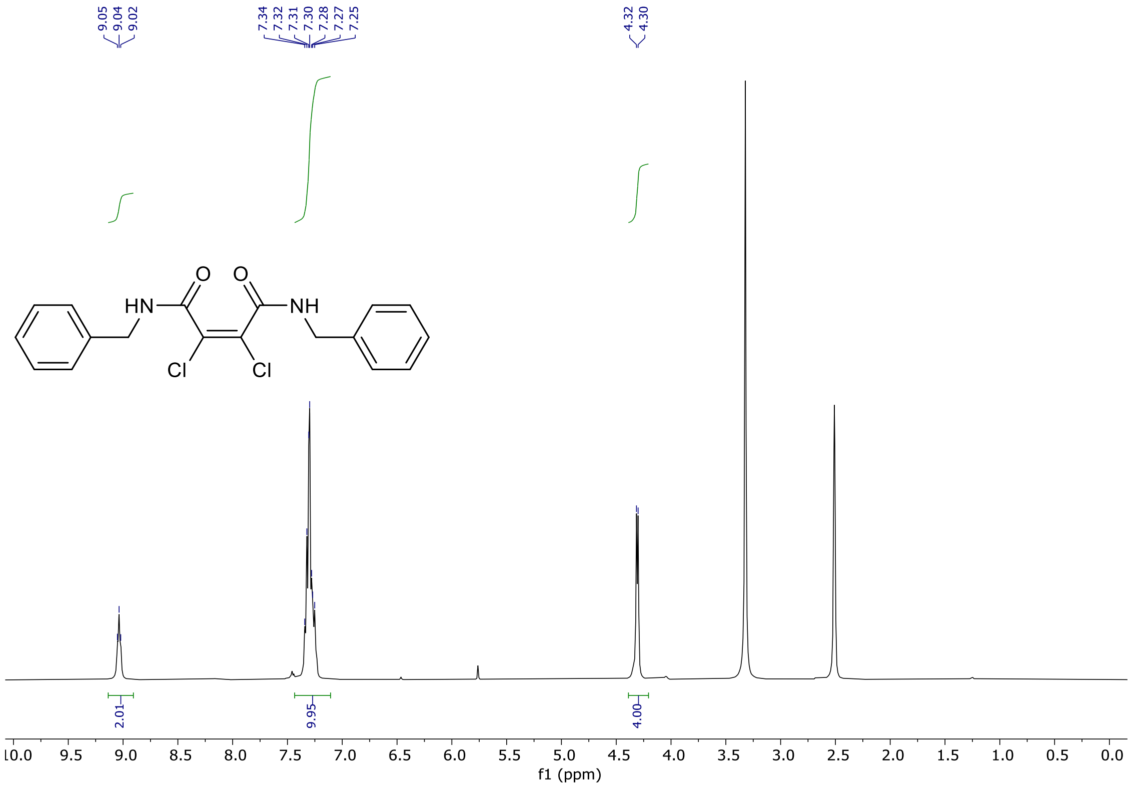

Figure S106. ^{1}H NMR Spectrum of **11**.

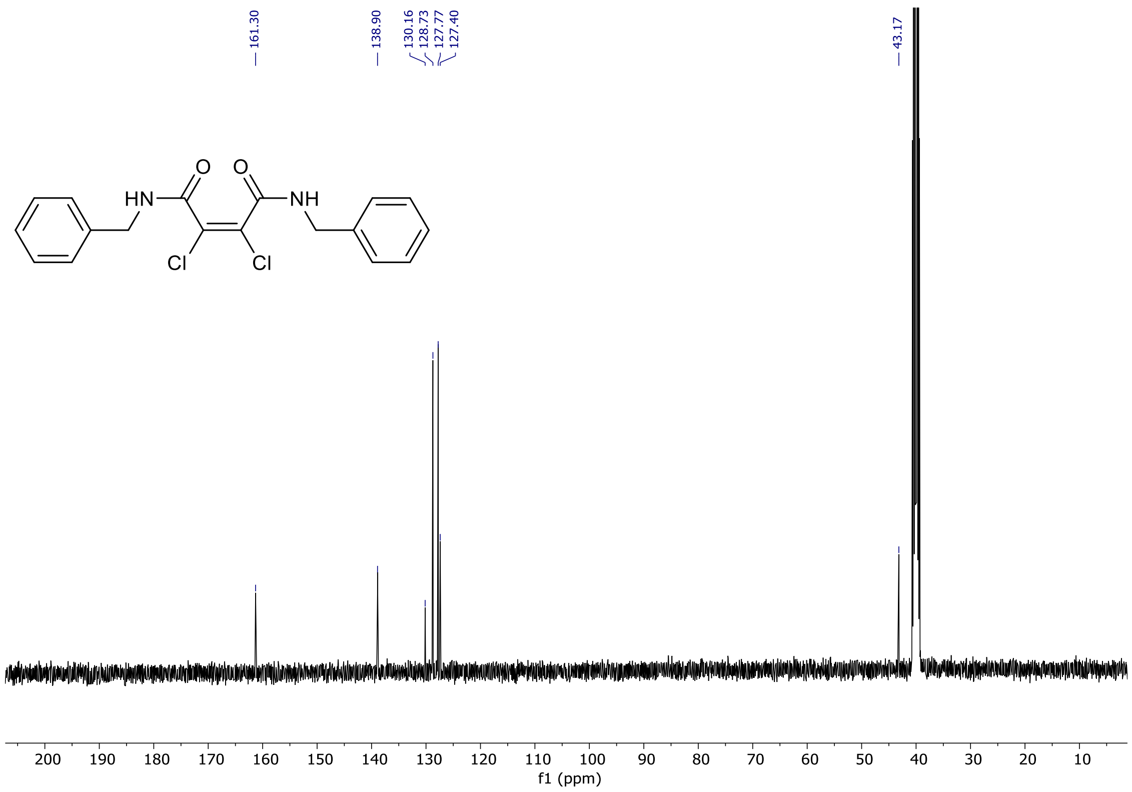

Figure S107. ^{13}C NMR Spectrum of **11**.

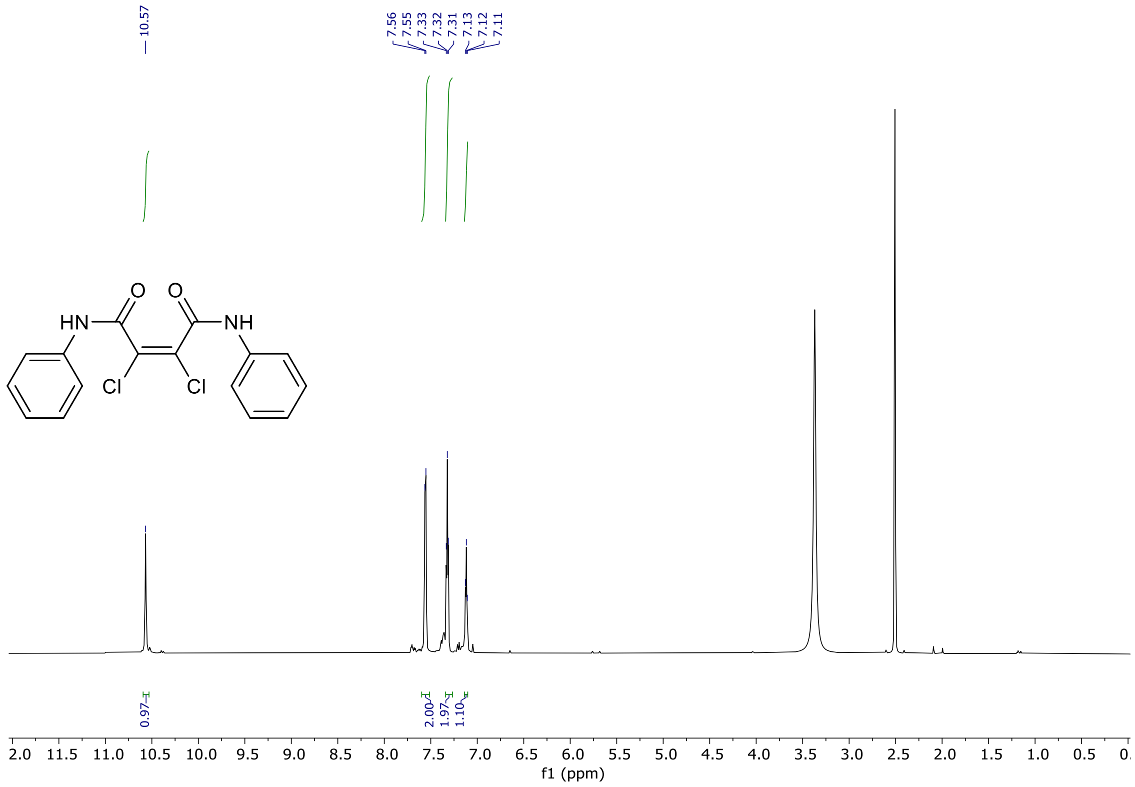

Figure S108. ^{1}H NMR Spectrum of **12**.

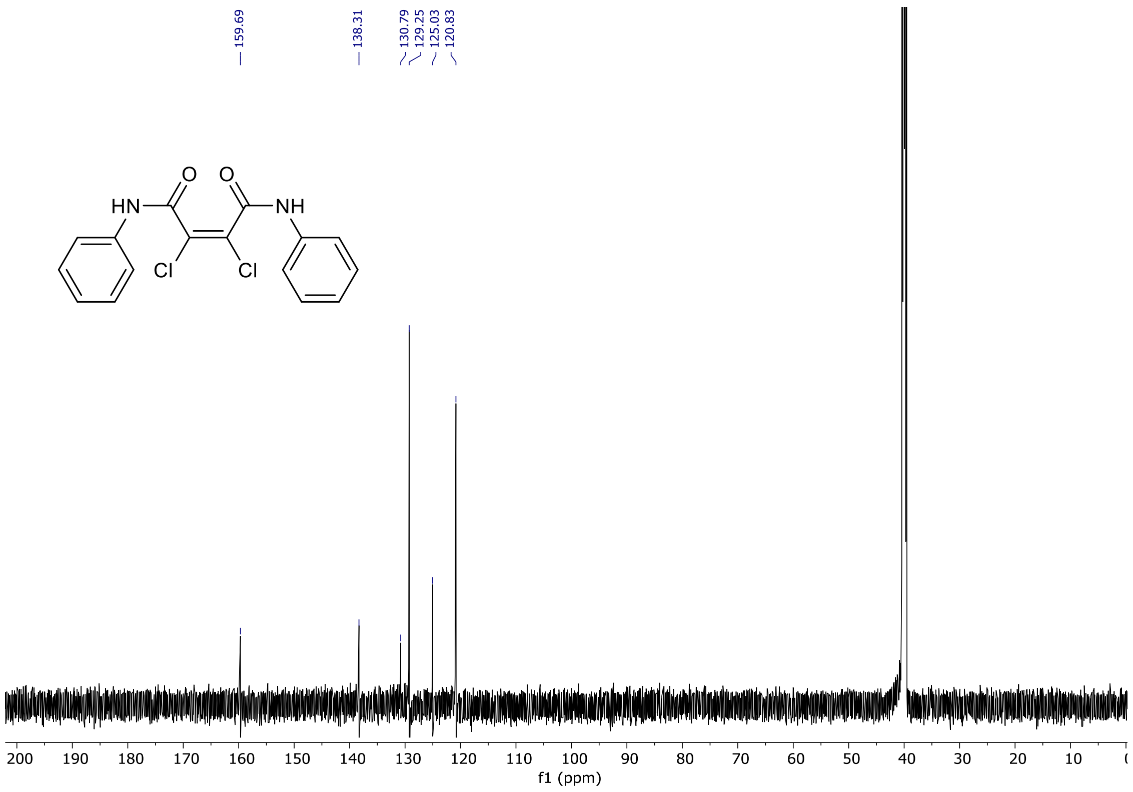

Figure S109. ^{13}C NMR Spectrum of **12**.

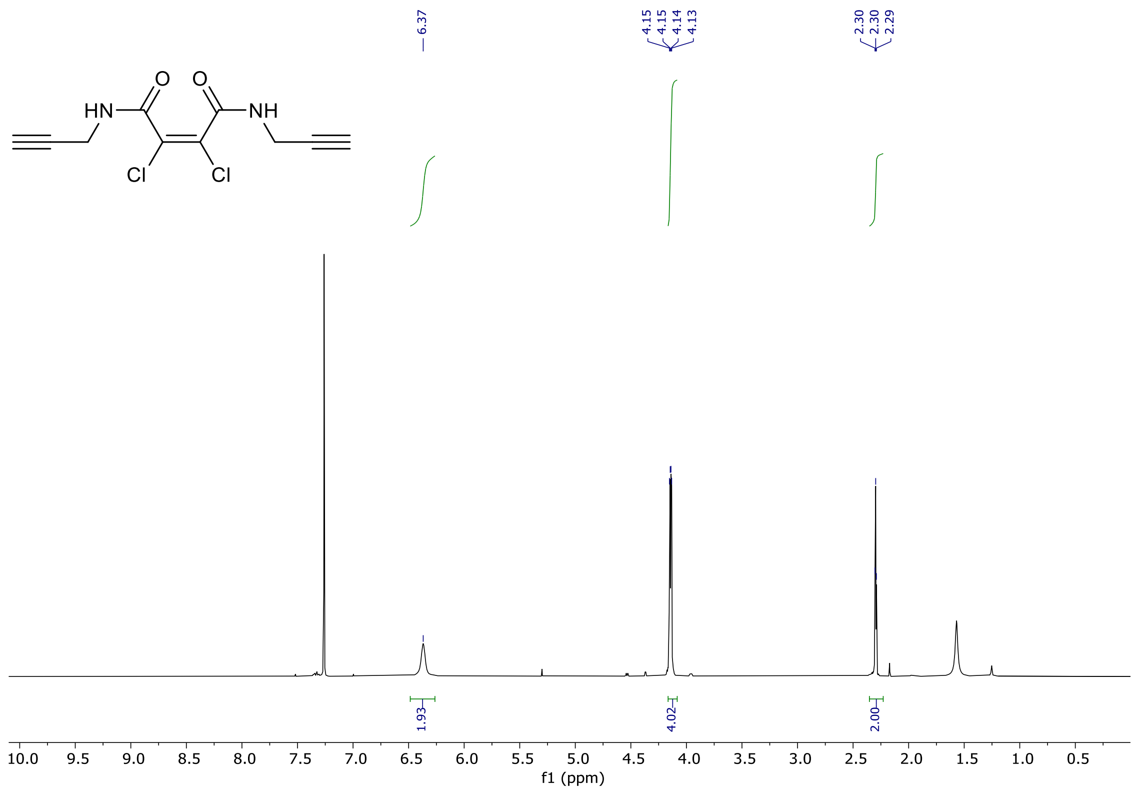

Figure S110. ^{1}H NMR Spectrum of **13**.

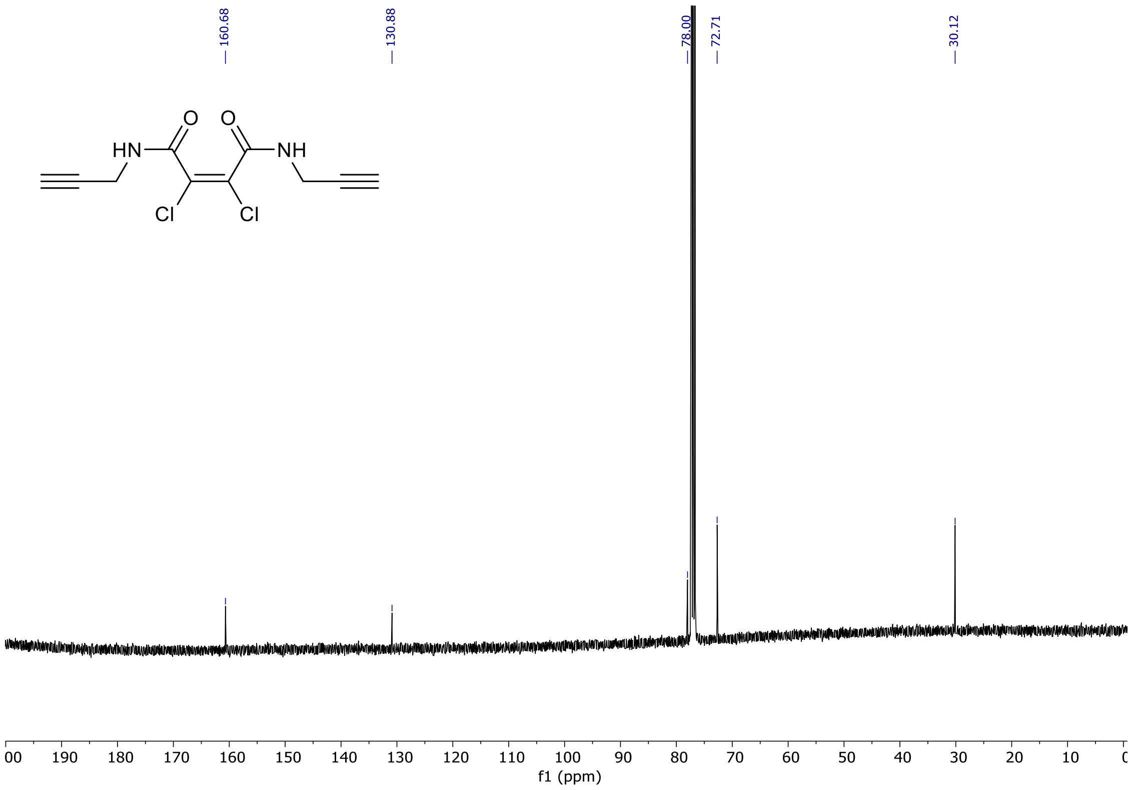

Figure S111. ^{13}C NMR Spectrum of **13**.

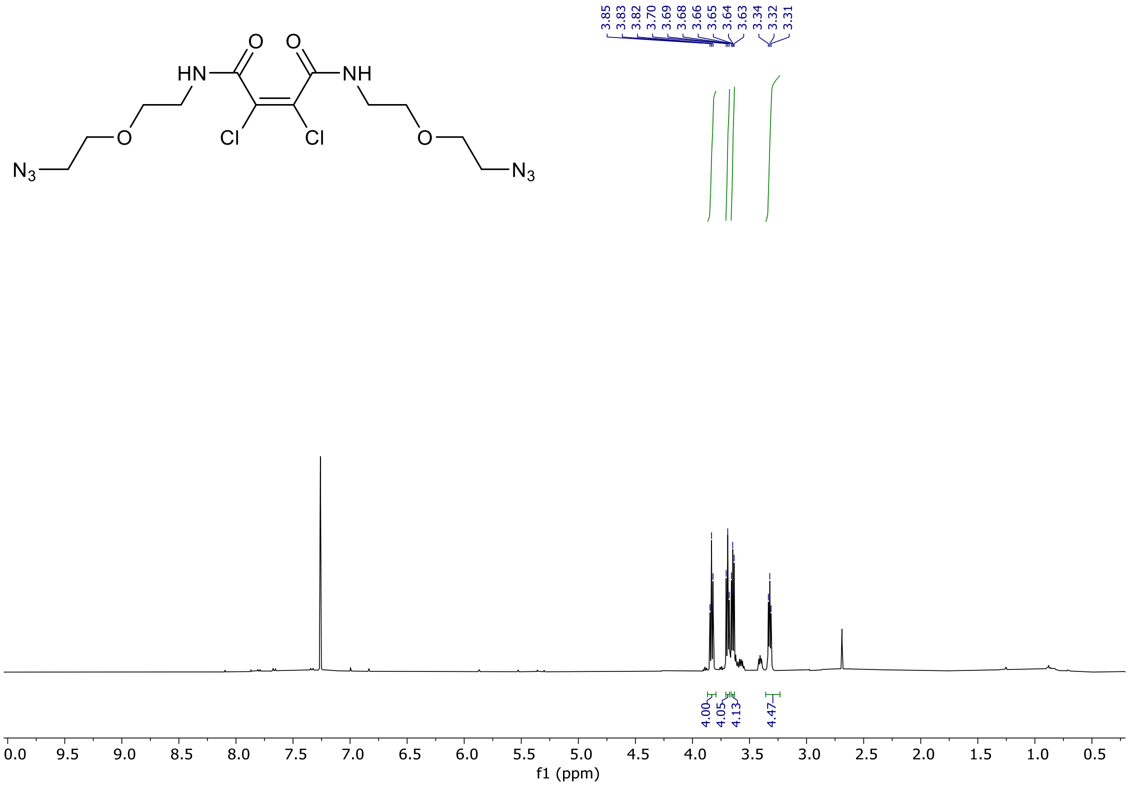

Figure S112. ^{1}H NMR Spectrum of **14**.

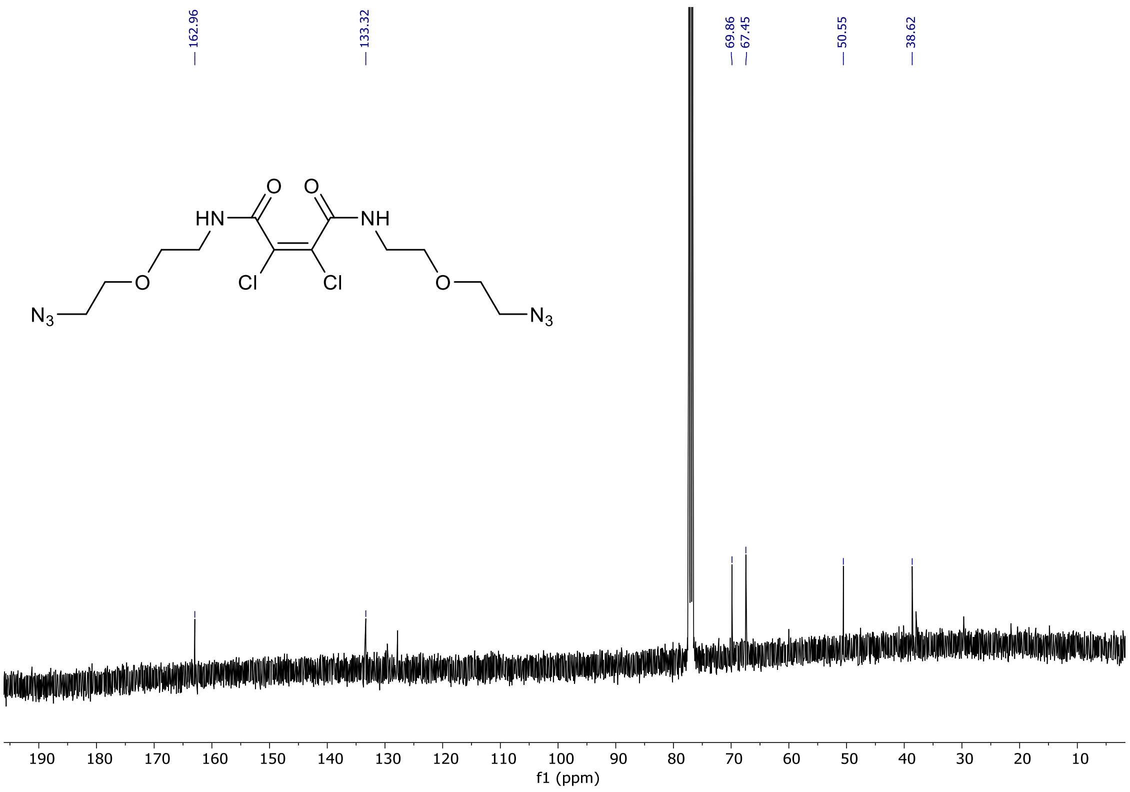

Figure S113. ^{13}C NMR Spectrum of **14**.
